# Enantioenriched α-Vinyl 1,4-Benzodiazepines and 1,4-Benzoxazepines via Enantioselective Rhodium-Catalyzed Hydrofunctionalizations of Alkynes and Allenes

**DOI:** 10.1021/acs.joc.1c01268

**Published:** 2021-07-14

**Authors:** Álvaro Velasco-Rubio, Rodrigo Bernárdez, Jesús A. Varela, Carlos Saá

**Affiliations:** Centro Singular de Investigación en Química Biolóxica e Materiais Moleculares (CiQUS), Departamento de Química Orgánica, Universidade de Santiago de Compostela, 15782 Santiago de Compostela, Spain

## Abstract

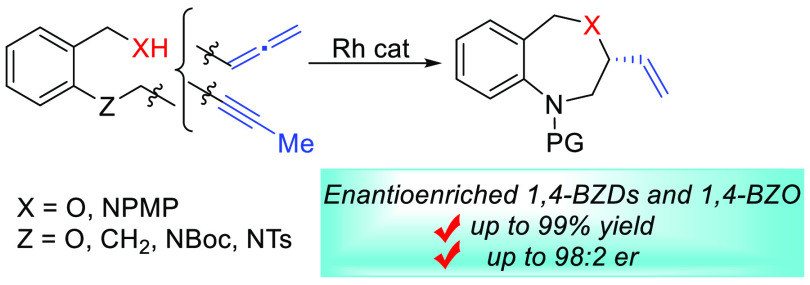

Benzofused seven-membered heterocycles such as 1,4-benzo[*e*]diazepines (1,4-BZDs) and 1,4-benzo[*e*]oxazepines (1,4-BZOs) were efficiently synthesized by Rh-catalyzed hydrofunctionalization of internal alkynes and allenes in good to excellent yields. The asymmetric hydroamination of (aminomethyl)anilines gave rise to 3-vinyl-1,4-BZDs with excellent enantioselectivities. Orthogonal *N*-deprotection of 1,4-BZDs allowed an easy entry to an advanced pyrrolobenzodiazepine metabolite of the V_2_-receptor antagonist Lixivaptan.

Benzofused seven-membered rings containing two heteroatoms (N, O) comprise the structural core of a privileged family of drugs employed to treat several indications.^1^ 1,4-Benzo[*e*]diazepines (1,4-BZDs) are known to interact with a variety of human receptors^2^ and have been extensively used to treat Central Nervous System (CNS) illnesses,^3^ cancer,^4^ or HIV virus.^5^ In addition, several drugs and advanced metabolites possess a stereodefined chiral Csp^3^ in C-3 (1,4 benzodiazepine numbering), which enhances their biological activity,^6^ e.g., the pyrrolobenzodiazepines (PBZDs),^7^ bearing a [7,5] ring fusion with an *N*-bridgehead (Figure 1). On the other hand, 1,4-benzo[*e*]oxazepines (1,4-BZOs) possess recognized pharmacological activity in the treatment of Alzheimer disease^8^ and as tranquilizers.^9^

**Figure 1:**
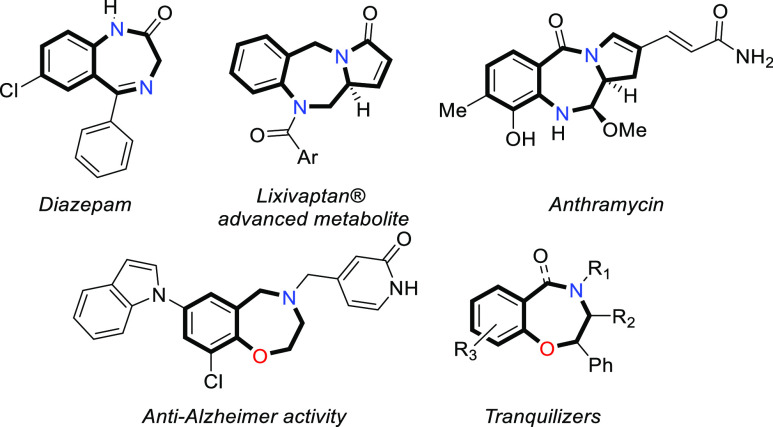
Bioactive 1,4-benzodiazepines and 1,4-benzoxazepines.

These highly relevant biological activities of 1,4-BZDs have inspired chemists over the years to develop a variety of synthetic approaches based on Friedel–Crafts reactions,^10^ ring expansions,^11^ aza-Michael cyclizations,^12^ click chemistry,^13^ heteroannulations,^14^ Ugi condensations,^15^ or 1,5-hydride transfer cyclization reactions.^16^ Although all of these strategies could be considered very useful to build the azaheterocycle skeleton, they lack the capacity to introduce stereodefined Csp^3^ formation in C-3, e.g., a chiral allylic/homoallylic amine (Figure 1)

In this regard, transition metal-catalyzed asymmetric hydrofunctionalization/cyclization of allenes/internal alkynes has been used as an eco-friendly strategy to afford enantioenriched five- and six-membered heterocycles from achiral starting materials.^17^ The combination with a catalytic amount of Brønsted acids allows the π-allyl intermediate formation that can be subsequently trapped with *N*- and *O*-nucleophiles to afford the corresponding chiral allylic amine or allyl ether (Scheme 1).^17^ This methodology was pioneered by Yamamoto (Scheme 1a),^18^ who was able to obtain a racemic five-membered ring in a Pd-catalyzed hydroamidation of allenes,^18a^ and later the enantioenriched five- and six-membered rings in a Pd-catalyzed hydroamidation of internal alkynes.^18b^ The groups of Toste, Liu, and Widenhoefer were working successfully on catalytic Au and Bronsted acid heterocyclizations of allenes (Scheme 1b).^19^ Recently, the group of Breit^20^ has developed an intensive study of the Rh-catalyzed hydrofunctionalizations to afford enantioenriched α-vinylated five- and six-membered azaheterocycles (through NTs nucleophiles)^20e,20f^ and tetrahydropyrans (Scheme 1c).^20g^ However, only a single benzofused seven-membered azaheterocycle, 4-vinyl-tetrahydrobenzo[*b*][1,5]-benzoxazepine, could be synthesized in low chemical yield but good ee using the same protocol.^21^ Herein, we report a Rh-catalyzed hydrofunctionalization of internal alkynes and allenes to benzofused seven-membered heterocycles employing substrates bearing N–Ar groups as nitrogenated nucleophiles.^21g^ The enantioselective hydroamination to 3-vinyl-1,4-BZDs and hydroalkoxylation to 3-vinyl-1,4-BZO is conveniently disclosed (Scheme 1).

**Scheme 1:**
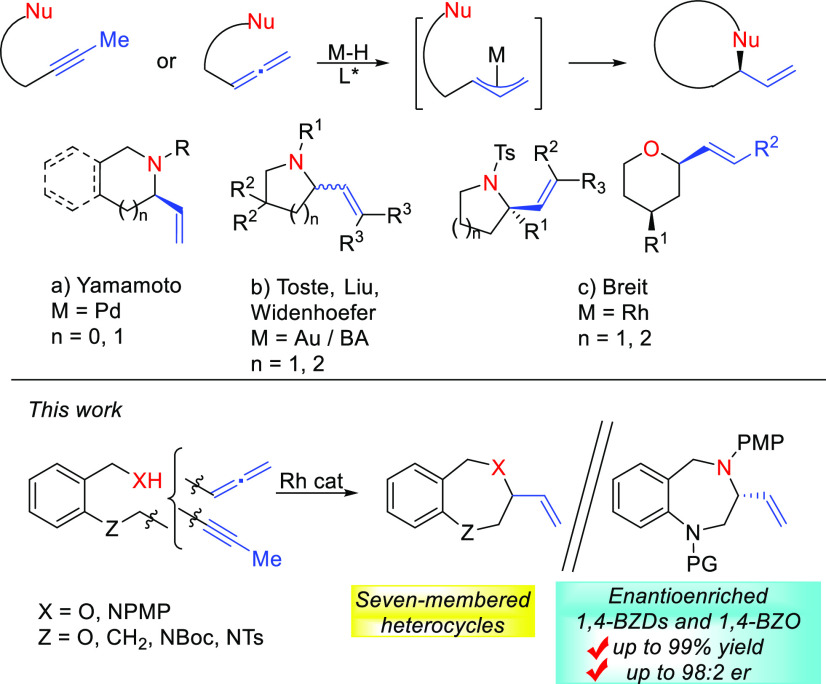
Rh-Catalyzed Hydrofunctionalizations of Alkynes and Allenes to α-Vinylated Heterocycles

We began our study exploring the virtually unknown intramolecular Rh-catalyzed hydrofunctionalizations of internal alkynes to seven-membered heterocycles (Scheme 2). Gratifyingly, benzylic alcohol **1a** (X = O, Z = NTs) smoothly cyclized, under standard conditions,^20a^ to the corresponding 3-vinyl-1,4-benzoxazepine **2a** in very good yield. On changing the nature of the heteroatoms, using oxygen as alkyne tether and PMP-protected amine as a nucleophile,^22^**1b** (X = NPMP, Z = O), the heterocyclization efficiency to **2b** dropped to 41% yield. In this case, partial depropargylation of starting material was detected, whereas when the carbon-tethered alkynylamine **1c** (X = NPMP, Z = CH_2_) was used, the corresponding α-vinyl-2-benzazepine **2c** was isolated in a moderate 57% yield.^23^ To our delight, when both the nucleophile and the alkyne tether were nitrogen atoms, **1d** (X= NPMP, Z = NBoc), the hydroamination smoothly occurred to give the desired 3-vinyl-1,4-BDZ **2d** in fairly good yield.

**Scheme 2:**
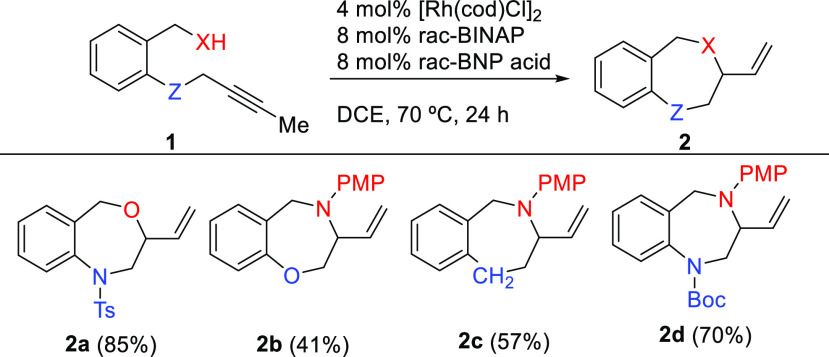
Rh-Catalyzed Hydrofunctionalizations of Internal Alkynes **1a**–**d** to Seven-Membered Heterocycles **2a**–**d**

To accomplish our synthetic goal, we then proceeded to evaluate the Rh-catalyzed asymmetric hydroamination of **1d** (Table 1), with a slight modification of our previous conditions regarding reactants loadings and temperature.

**Table 1:**
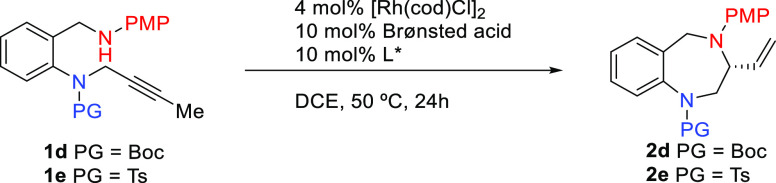
Optimization of Rh-Catalyzed Asymmetric Hydroamination of Internal Alkynes **1d** and **1e**

entry^a^	alkyne **1**	chiral ligand (L*)	Brønsted acid	1,4-BDZ **2** yield (%)	er
1	**1d**	Josiphos-SL-J002–1	*rac*-BNP acid	<5	
2	**1d**	(*R*)-BINAP	PPTS	traces	
3	**1d**	(*R*)-BINAP	*rac*-BNP acid	19	57:43
4^b^	**1d**	(*R*)-BINAP	*rac*-BNP acid	58	57:43
5	**1d**	(*R*)-DTBM-Segphos	*rac*-BNP acid	73	57:43
6	**1d**	(*S*)-DTBM-Garphos	*rac*-BNP acid	81	67:33^c^
7	**1e**	(*R*)-DTBM-Garphos	TFA	50	80:20
8^d^	**1e**	(*R*)-DTBM-Garphos	TFA	60	60:40

a Reaction conditions: 4 mol % [Rh(cod)Cl]_2_, 10 mol % L*, 10 mol % Brønsted acid, DCE (0.4 M).

b 5 days.

c The (*S*)-**2d** was observed as a major enantiomer.

d Reaction performed at 70 °C. PMP = *p*-(methoxyphenyl).

Using Josiphos-SL-J002–1, a member of the typical family of chiral ligands for intramolecular asymmetric hydroaminations,^20e^ only gave traces of **2d** (Table 1, entry 1). When (*R*)-BINAP was used as chiral ligand, 3-vinyl-1,4-benzodiazepine **2d** could only be obtained in a low 19% yield and 57:43 er in the presence of *rac*-BNP as Brønsted acid (Table 1, entries 2 and 3). The yield increased to 58%, without any erosion of enantioselectivity, when the reaction was run for 5 days at the same temperature (Table 1, entry 4). Pleasingly, when chiral biaryl phosphine ligands (*R*)-DTBM-Segphos and (*S*)-DTBM-Garphos were used (Table 1, entries 5 and 6), good yields and promising enantioselectivities of **2d** (73–81%, 14–34% ee) were obtained.^24^ We reasoned that a more rigid *N*-protecting group (e.g., tosyl group) might help to increase the enantioselectivity of the hydroamination. In fact, when **1e** (PG = Ts) was used in the presence of (*R*)-DTBM-Garphos as chiral ligand and TFA as Brønsted acid (p*K*_a_ = 0.52), the corresponding 3-vinyl-1,4-benzodiazepine **2e** was obtained in 50% yield and 80:20 er (Table 1, entry 7).^25^ Unfortunately, reaction at a higher temperature, 70 °C, had limited effect in yield with quite considerable erosion of enantioselectivity (Table 1, entry 8).^24^

The fact that the best result regarding the enantioselectivity was 60% made us wonder about the influence of the Brønsted acid in the isomerization process of the internal alkyne to the terminal allene. So, we decided to directly synthesize allenes **3d** and **3e** to make them react under the optimized conditions (Table 2). Unfortunately, when using chiral biaryl phosphine ligands (*R*)-DTBM-Segphos and (*R*)-DTBM-Garphos, allene **3d** gave rise to 3-vinyl-1,4-benzodiazepine **2d** in moderate to good yields with modest enantioselectivities (Table 2, entries 1–3). Interestingly, hydroaminations occurred more efficiently in terms of chemical yields and enantioselectivities with the more rigid tosylated allene **3e**. Under standard conditions with PPTS as Brønsted acid (p*K*_a_ = 5.21) and (*R*)-DTBM-Segphos as chiral ligand, the 3-vinyl-1,4-benzodiazepine **2e** could be obtained in 80% yield and 90:10 er (Table 2, entry 4).^26^ To our delight, upon changing the nature of the chiral ligand to (*R*)-DTBM-Garphos, the 1,4-BDZ **2e** could be obtained in 70% yield with an excellent 95:5 er (Table 2, entry 5). Curiously, the employment of chloroacetic acid (p*K*_a_ = 2.87) favors the reaction to give an excellent yield (90%) but with slight erosion of enantioselectivity (91:9 er, Table 2, entry 6). Conversely, when the reaction was performed at a higher temperature, 70 °C, a lower chemical yield was obtained (60%) but without loss of enantioselectivity (95:5 er, Table 2, entry 7).^24^ This result contrasts with the drop of ee when using the alkyne **1e** at 70 °C (Table 1, entry 8). We speculate that the nature of the Brønsted acid is crucial (PPTS vs TFA) to favor a cationic intermediate (with PPTS) that would evolve via an “outer sphere” mechanism rather than a more neutral-like intermediate (with TFA) that might favor competitive mechanisms that would erode the enantioselectivity of the process.

**Table 2:**
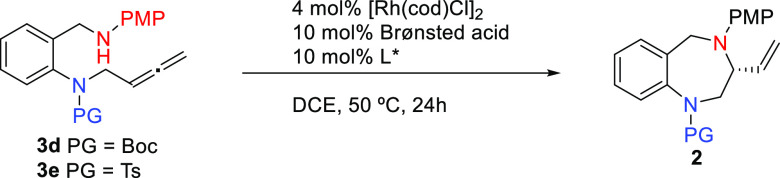
Optimization of Rh-Catalyzed Asymmetric Hydroamination of Allenes **3d** and **3e**

entry^a^	allene **3**	chiral ligand (L*)	Brønsted acid	1,4-BDZ **2** yield (%)	er
1	**3d**	(*R*)-DTBM-Segphos	*rac*-BNP acid	50	74:26
2	**3d**	*(R*)-DTBM-Garphos	*rac*-BNP acid	76	75:25
3^b^	**3d**	*(R*)-DTBM-Garphos	*rac*-BNP acid	70	78:22
4	**3e**	(*R*)-DTBM-Segphos	PPTS	80	90:10
5	**3e**	*(R*)-DTBM-Garphos	PPTS	70	95:5
6	**3e**	*(R*)-DTBM-Garphos	ClCH_2_CO_2_H	90	91:9
7^c^	**3e**	*(R*)-DTBM-Garphos	PPTS	60	95:5

a Reaction conditions: 4 mol % [Rh(cod)Cl]_2_, 10 mol % L*, 10 mol % Brønsted acid, DCE (0.4 M).

b 0.2 M instead of 0.4 M.

c 70 °C. PMP = *p*-(methoxyphenyl).

Having established the optimized conditions, a series of *N*-benzylamino *N*-tosyl allenes **3** bearing different substituents on the benzene ring were screened (Scheme 3).^27^ All of the tested substrates bearing strong EDG and EWG (OMe, CF_3_), halogens (F, Cl, Br), or alkyl (Me) groups in any position of the ring are well tolerated to give the corresponding 3-vinyl-1,4-BDZs **2f**–**2n** in rather good yields and excellent enantiomeric ratios, indicating that the electronic properties of the aromatic moiety have little influence on the reactivity and enantioselectivity. By contrast, the asymmetric reaction was very sensitive to the nature of the nucleophile since the hydroxylated allene **3a** smoothly cyclized to the 3-vinyl-1,4-benzoxazepine **2a** (90% yield) but with a moderate 78:22 er.

**Scheme 3:**
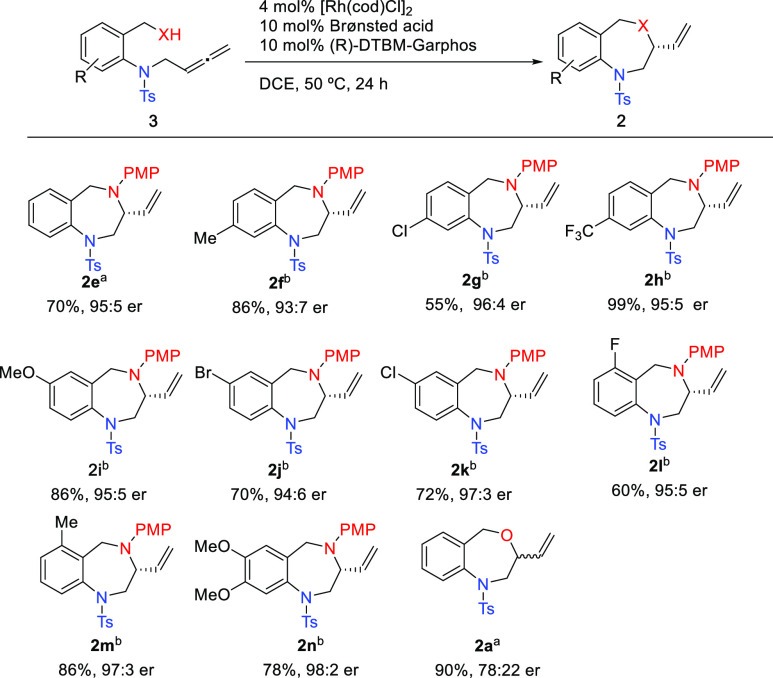
Scope of the Asymmetric Rh-Catalyzed Hydrofunctionalizations of Allenes **3** PPTS. ClCH_2_CO_2_H

From the literature^28^ and our own observations/results during the screening of the reaction conditions, we cannot anticipate which one of the two competing pathways typically proposed for Rh-catalyzed hydrofunctionalizations based on “inner” (reductive elimination) or “outer” (external nucleophile attack) is operating.^19,20^ The nature of the nucleophile plays a crucial role in the last C–X (N, O) bond formation (hydroamination vs hydroalkoxylation). Thus, when NHPMP acts as a nucleophile, an S_N_2 attack over the Rh-π-allyl complex may occur (“outer sphere”).^29^ On the other hand, alcohols typically follow a reductive elimination when they act as a nucleophile (“inner sphere”), and this may cause the low enantioselectivity found in the cyclization of benzylic alcohol **3a**.^30^

We next turned toward derivatization of the enantioenriched 3-vinyl-1,4-benzodiazepine obtained (Scheme 4). Orthogonal *N*-deprotection of the PMP group of **2e** was carried under typical oxidative cleavage conditions (CAN in a mixture of MeCN/H_2_O) to give rise to the desired benzylammonium salt **4** in 85% yield.^31^ On the other hand, removal of the Ts group of **2e** could be achieved using mild reducing conditions (Na, naphthalene, rt) to afford the aniline **5** in 85% yield.^32^ The benzylammonium salt **4** reacted smoothly with acryloyl chloride to afford amide **6** in 65% yield. Finally, an RCM (Hoveyda–Grubbs catalyst second G, 87%) gave rise to the pyrrol-2-one ring **7**, which is an advanced metabolite of Lixivaptan, a vasopressin V_2_-receptor antagonist to treat congestive heart failure and liver cirrhosis.^7a,33^ The derivatization process occurred without erosion of enantioselectivity (94:6 er).

**Scheme 4:**
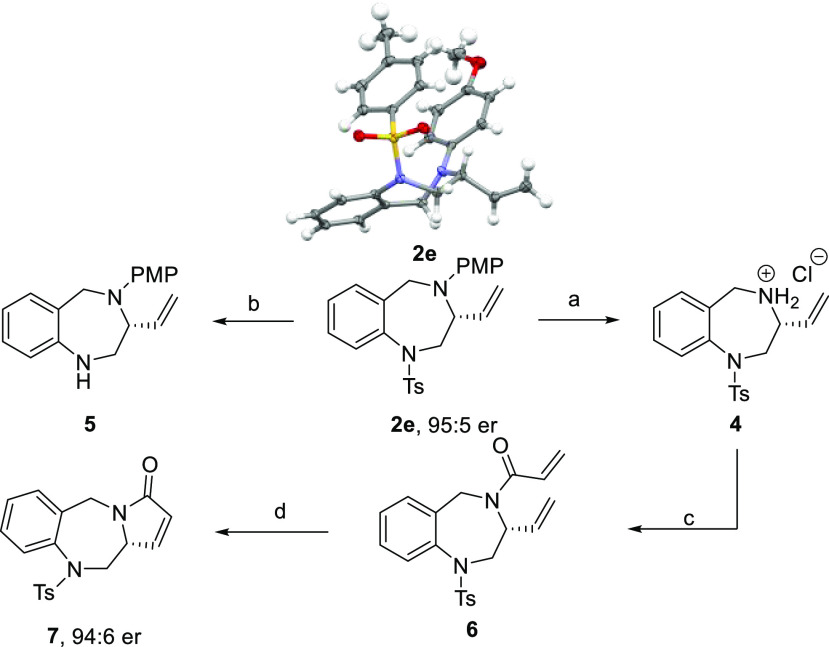
Derivatization of 3-Vinyl-1,4-benzodiazepine **2e** Conditions: (a) 2.5 equiv of CAN, MeCN/H_2_O, then HCl (1 M) in Et_2_O, 85% yield; (b) 6 equiv of Na, 0.2 equiv of naphthalene, THF, rt, 16 h, 85% yield; (c) 2 equiv of acryloyl chloride, 2 equiv of Et_3_N, 0.1 equiv of DMAP, DCM, 0 °C to rt, 2 h, 65% yield; (d) 10 mol % Hoveyda–Grubbs catalyst second G, DCM, reflux, 36 h, 87% yield.

In summary, we have developed an intramolecular Rh-catalyzed hydrofunctionalization of internal alkynes and allenes to benzofused seven-membered heterocycles. The asymmetric hydroamination of (aminomethyl)aniline derivatives afforded chiral 3-vinyl-1,4-benzodiazepines (1,4-BZDs) with good to excellent yields and enantioselectivities. Orthogonal *N*-deprotection of 1,4-BZDs allowed an easy manipulation that led to an enantioenriched advanced metabolite of the V_2_-receptor antagonist Lixivaptan. Mechanistic investigations are currently underway in our laboratory.

## Experimental Section

### General Information

All reactions were performed under an inert atmosphere of argon and with anhydrous solvents in a glassware oven or flame-dried at 80 °C unless otherwise stated. All chemicals were purchased from Acros Organics Ltd., Aldrich Chemical Co. Ltd., Alfa Aesar, Strem Chemicals Inc., Fluorochem Ltd., or TCI Europe N.V. chemical companies and used without further purification, unless otherwise stated. Analytical thin-layer chromatography was carried out on silica-coated aluminum plates (silica gel 60 F_254_ Merck) or on aluminum sheets (aluminum oxide 60 F_254_ neutral Merck) using UV light as a visualizing agent (254 nm) and KMnO_4_ (solution of 1.5 g of potassium permanganate, 10 g of potassium bicarbonate and 1.25 mL of 10% sodium hydroxide in 200 mL of water) with heat as developing agents. Flash column chromatography was performed on silica gel 60 (Merck, 230–400 mesh) with the indicated eluent. All other reagents and solvents (acetonitrile, dichloromethane, dichloroethane, tetrahydrofuran, toluene, and methanol) were used dry, unless otherwise indicated.

Enantiomeric ratio (er) values were determined on an Agilent HPLC 1100 Series or on a Jasco SFC 4000 series using commercially available chiral columns.

^1^H NMR, ^13^C NMR, and DEPT experiments were carried out using a Varian Inova 500, Varian Inova 400 MHz or Varian Mercury 300 MHz. All NMR experiments were recorded at 298 K otherwise stated. All chemical shifts are reported in parts per million (ppm) and referenced to residual solvent peaks. Coupling constants (*J*) are given in hertz (Hz). Multiplicities are reported as follows: s = singlet; d = doublet; t = triplet; q = quartet, m = multiplet or as a combination of them. The proton signals corresponding to NH and OH groups may not appear in the ^1^H NMR spectra due to deuterium exchange.

Reactions were followed using a GC Agilent HP-6890N with a mass spectroscopy HP-5973N using DB-35MS and HP-5MS columns for the GC and a chemical ionization font for the MS. Mass spectrometry analysis was carried out using a Micromass Autospec, a TRACE MS, or a HP-5988-A with chemical ionization and a Bruker Microtof APCI using chemical ionization spectrometers at the CACTUS Facility (Universidade de Santiago de Compostela).

X-ray crystallographic analysis was performed at the CACTUS facility of the University of Santiago de Compostela.

### General Procedure for the Preparation of Alkynes **1a**, **1d**, and **1e**

#### PG-Amine Protection

Boc_2_O (9.3 g, 42 mmol, 1.7 equiv), DMAP (0.92 g, 7.5 mmol, 0.3 equiv), and Et_3_N (3.5 mL, 25 mmol, 1 equiv) were added at rt to a solution of ethyl 2-aminobenzoate (4.13 g, 25 mmol, 1 equiv) in 250 mL of dry THF (0.1 M), and the reaction mixture was then stirred at 60 °C in an oil bath for 24 h. Then the reaction was quenched at rt with H_2_O, and both layers were separated. The aqueous layer was extracted with Et_2_O (3 × 50 mL), and the combination of organic layers was washed with brine, dried over Na_2_SO_4_, and concentrated in vacuo. The compound **S1a** was purified by silica gel column chromatography with hexane/EtOAc (39:1) as the eluent.

The Ts-derivative **S1b** was synthesized according to the literature.^34^

##### Ethyl 2-((*tert*-Butoxycarbonyl)amino)benzoate (**S1a**):

70% yield (5.57 g, 21 mmol); amorphous white solid; ^1^H NMR (300 MHz, CDCl_3_) δ 10.30 (s, 1H), 8.47–8.41 (m, 1H), 8.01 (dd, *J* = 8.1, 1.7 Hz, 1H), 7.55–7.46 (m, 1H), 6.99 (ddd, *J* = 8.3, 7.3, 1.2 Hz, 1H), 4.37 (q, *J* = 7.2 Hz, 2H), 1.53 (d, *J* = 0.9 Hz, 8H), 1.44–1.37 (m, 3H); ^13^C{^1^H} NMR (75 MHz, CDCl_3_) δ 168.0, 152.8, 142.3, 134.3, 130.8, 120.9, 118.6, 114.5, 80.3, 61.1, 28.3, 14.2; MS (CI), *m*/*z* (%) 266 (M^+^ + 1, 100).

#### N-Alkylation.^35^

A round-bottomed flask equipped with a stirring magnetic bar was flamed-dried under a vacuum and backfilled with argon. Then, it was charged with NaH (1.2 equiv), put under a vacuum, and backfilled with argon for three times. Then DMF (0.33 M) was added, and the mixture was cooled to 0 °C. A solution of *N*-protected aniline **S2** (1 equiv) in DMF (2 mL) was then added slowly, and the mixture was stirred at 0 °C for 2 h. Afterward, a propargyl bromide derivative (1.3 equiv) was added, and the reaction was allowed to warm slowly to rt and stirred for 16 h. The reaction was quenched with a saturated solution of NH_4_Cl (aq) and extracted with EtOAc. The aqueous layer was extracted with EtOAc, and the combination of organic layers was washed with a saturated solution of NH_4_Cl (aq) (3 × 100 mL). The combination of organic layers was dried over Na_2_SO_4_ and concentrated in vacuo. The residue was then purified by silica gel column chromatography with hexanes/EtOAc (8:2) as the eluent to give the desired products **S2**.

##### Ethyl 2-(But-2-yn-1-yl(*tert*-butoxycarbonyl)amino)benzoate (**S2d**):

93% yield (2.95 g, 9.3 mmol); colorless oil; ^1^H NMR (300 MHz, CDCl_3_), δ (ppm): 7.91 (d, *J* = 7.7 Hz, 1H), 7.58–7.22 (m, 3H), 4.69 (d, *J* = 17.5 Hz, 1H), 4.41–4.20 (m, 2H), 3.96 (d, *J* = 17.4 Hz, 1H), 1.75 (s, 3H), 1.51 (s, 3H), 1.35 (t, *J* = 7.0 Hz, 3H), 1.28 (s, 6H); ^13^C{^1^H} NMR (75 MHz, CDCl_3_) δ 163.2, 133.0, 132.4, 131.6, 131.0, 130.3, 129.8, 128.8, 127.1, 80.3, 74.9, 61.1, 39.5, 28.0, 14.1, 3.6; MS (CI), *m*/*z* (%) 318 (M^+^ + 1, 100).

##### Ethyl 2-((*N*-(But-2-yn-1-yl)-4-methylphenyl)sulfonamido)benzoate (**S2e**):

90% yield (3.34 g, 9 mmol); amorphous off-white solid; ^1^H NMR (300 MHz, CDCl_3_) δ 7.89–7.81 (m, 1H), 7.64–7.56 (m, 2H), 7.45–7.36 (m, 2H), 7.26–7.19 (m, 2H), 7.17–7.10 (m, 1H), 4.54 (s, 1H), 4.28 (q, *J* = 7.1 Hz, 2H), 2.41 (s, 3H), 1.69 (t, *J* = 2.4 Hz, 3H), 1.37 (t, *J* = 7.1 Hz, 3H); ^13^C{^1^H} NMR (75 MHz, CDCl_3_) δ 166.1, 143.1, 137.9, 137.4, 133.0, 131.8, 131.5, 131.1, 129.1, 128.7, 127.8, 81.5, 74.0, 61.4, 42.0, 21.5, 14.1, 3.4; MS (CI), *m*/*z* (%) 372 (M^+^ + 1, 100).

#### Ester Reduction.^36^

DIBAL-H (1 M in DCM, 2.2 equiv) was added dropwise to a stirred solution of the ester **S2** (1 equiv) in DCM (0.3 M) at −78 °C. The reaction was then stirred at that temperature for 3 h. Afterward, MeOH (5 mL) was added followed by a saturated solution of the Rochelle Salt at −78 °C. The reaction was then warmed up to rt and stirred for 1 h. The mixture was extracted with DCM (3 × 30 mL), and the combination of organic layers was washed with a saturated solution of NaCl (aq), dried over Na_2_SO_4_, and concentrated in vacuo. The residue was then purified by silica gel column chromatography with hexanes/EtOAc (9:1 to 7:3) as the eluent to afford the desired product **S3d**/**1a**.

##### *tert*-Butyl But-2-yn-1-yl(2-(hydroxymethyl)phenyl)carbamate (**S3d**):

99% yield (2.53 g, 9.2 mmol); amorphous white solid. It was used in the next step without further purification.

##### *N*-(But-2-yn-1-yl)-*N*-(2-(hydroxymethyl)phenyl)-4-methylbenzenesulfonamide (**1a**):

91% yield (2.8 g, 8.5 mmol); amorphous off-white solid; ^1^H NMR (300 MHz, CDCl_3_) δ 7.64–7.55 (m, 3H), 7.37 (td, *J* = 7.5, 1.3 Hz, 1H), 7.33–7.23 (m, 2H), 7.15 (td, *J* = 7.7, 1.7 Hz, 1H), 6.65 (dd, *J* = 8.0, 1.3 Hz, 1H), 4.93 (s, 1H), 4.60 (s, 1H), 4.33 (s, 2H), 3.00–2.89 (m, 1H), 2.45 (s, 3H), 1.65 (t, *J* = 2.4 Hz, 3H); ^13^C{^1^H} NMR (75 MHz, CDCl_3_) δ 144.0, 142.2, 137.3, 135.2, 131.0, 129.4, 129.3, 128.3, 128.3, 128.2, 82.0, 72.8, 61.3, 42.6, 21.6, 3.4; HRMS (MM: ESI-APCI+) *m*/*z* calcd for C_18_H_18_N_2_O_2_S [M – H_2_O]^+^ 312.1053, found 312.1059.

#### Alcohol Oxidation

DMP (1.1 equiv) was added to a stirred solution of the alcohol **S3d**/**1a** (1 equiv) in DCM (0.25 M) at rt. The mixture was stirred for 30 min. The reaction was quenched with a 1 M solution of NaOH (aq) and extracted with DCM (2 × 30 mL). The combination of organic layers was washed with brine, dried over Na_2_SO_4_, and concentrated in vacuo. The residue was used in the next step without further purification.

#### Reductive Amination

*p*-Anisidine (1.5 equiv) was added to a stirred solution of the aldehyde previously synthesized in MeOH (0.25 M) or (1:1 MeOH/DCM) under an argon atmosphere. The reaction was stirred at room temperature for 18 h. Then the reaction was cooled to 0 °C, and NaBH_4_ (1.1 equiv) was added portionwise. The reaction was then allowed to warm up to rt and stirred for 2 h. The reaction was quenched with water and extracted with DCM. The combination of organic layers was washed with brine, dried over Na_2_SO_4_, and concentrated in vacuo. The residue was then purified by silica gel column chromatography with hexanes/EtOAc (9:1 to 8:2) as the eluent to give the desired products **1d**/**1e**.

##### *tert*-Butyl But-2-yn-1-yl(2-(((4-methoxyphenyl)amino)methyl)phenyl)carbamate (**1d**):

80% yield; amorphous off-white solid; ^1^H NMR (300 MHz, DMSO-*d*_6_, 80 °C) δ 7.46–7.35 (m, 1H), 7.31–7.18 (m, 3H), 6.7 (d, *J* = 8.9 Hz, 2H), 6.5 (d, *J* = 8.9 Hz, 2H), 5.46 (bs, 1H), 4.30 (bs, 2H), 4.21 (bs, 2H), 3.64 (s, 3H), 1.75 (t, *J* = 2.4 Hz, 3H), 1.39 (s, 9H); ^13^C{^1^H} NMR (75 MHz, DMSO-*d*_6_, 80 °C) δ 153.0, 150.7, 142.7, 139.5, 137.6, 127.5, 127.1, 126.9, 126.8, 114.5, 113.0, 79.5, 79.4, 75.0, 55.2, 43.3, 39.3, 27.6, 2.5; HRMS (MM: ESI-APCI+) *m*/*z* calcd for C_23_H_29_N_2_O_3_ [M + H]^+^ 381.2173, found 381.2171.

##### *N*-(But-2-yn-1-yl)-*N*-(2-(((4-methoxyphenyl)amino)methyl)phenyl)-4-methylbenzenesulfonamide (**1e**):

70% yield (2 g, 4.8 mmol); amorphous off-white solid; ^1^H NMR (300 MHz, CDCl_3_) δ 7.64 (d, *J* = 7.9 Hz, 2H), 7.52 (d, *J* = 7.7 Hz, 1H), 7.29 (d, *J* = 8.1 Hz, 3H), 7.13 (t, *J* = 7.7 Hz, 1H), 6.76 (t, *J* = 7.1 Hz, 3H), 6.62 (d, *J* = 8.4 Hz, 2H), 4.52 (s, 2H), 4.33 (s, 2H), 4.08–4.01 (m, 1H), 3.74 (s, 3H), 2.45 (s, 3H), 1,65 (t, *J* = 2.4 Hz, 3H); ^13^C{^1^H} NMR (75 MHz, CDCl_3_) δ 152.1, 143.8, 142.6, 141.1, 137.7, 135.8, 129.4, 129.3, 129.1, 128.4, 127.4, 114.9, 114.3, 81.8, 73.0, 55.8, 45.4, 42.3, 21.6, 3.5; HRMS (MM: ESI-APCI+) *m*/*z* calcd for C_25_H_27_N_2_O_3_S [M + H]^+^ 435.1737, found 435.1730.

#### Preparation of Alkyne **1b**

##### 2-(But-2-yn-1-yloxy)benzaldehyde (**S4**)

To a suspension of K_2_CO_3_ (1 g, 7.2 mmol, 1.2 equiv) in DMF (3 mL) at rt was added salicylaldehyde (0.63 mL, 6 mmol, 1 equiv) followed by 1-bromo-2-butyne (0.58 mL, 6.6 mmol, 1.1 equiv). The mixture was then stirred at rt for 16 h. The reaction was quenched with water (10 mL). The aqueous layer was extracted with AcOEt (3 × 10 mL), and the combination of organic layers was washed with water (3 × 10 mL), brine (2 × 10 mL), dried over Na_2_SO_4_, and concentrated in vacuo. The residue was purified by silica gel column chromatography with hexanes/EtOAc (19:1) as the eluent to give **S6**: 83% yield (870 mg, 5 mmol); colorless oil; ^1^H NMR (300 MHz, CDCl_3_) δ 10.46 (s, 1H), 7.82 (dd, *J* = 7.6, 1.9 Hz, 1H), 7.53 (td, *J* = 7.3, 1.8 Hz, 1H), 7.09 (d, *J* = 8.5 Hz, 1H), 7.06–7.00 (m, 1H), 4.76 (q, *J* = 2.3 Hz, 2H), 1.83 (t, *J* = 2.3 Hz, 3H); ^13^C{^1^H} NMR (75 MHz, CDCl_3_) δ 189.7, 160.1, 135.7 128.3, 125.4, 121.2, 113.4, 84.7, 73.3, 57.1, 3.6; MS (CI), *m*/*z* (%) 175 (M^+^ + 1, 100).

##### *N*-(2-(But-2-yn-1-yloxy)benzyl)-4-methoxyaniline (**1b**)

used the general procedure for reductive amination, 70% yield (983 mg, 3.5 mmol); yellow oil; ^1^H NMR (300 MHz, CDCl_3_) δ 7.32 (d, *J* = 7.4 Hz, 1H), 7.25 (td, *J* = 7.9, 7.5, 1.5 Hz, 1H), 7.00 (d, *J* = 8.2 Hz, 1H), 6.95 (td, *J* = 7.4, 0.9 Hz, 1H), 6.81–6.73 (m, 2H), 6.68–6.61 (m, 2H), 4.72 (q, *J* = 2.3 Hz, 2H), 4.32 (s, 2H), 3.74 (s, 3H), 1.87 (t, *J* = 2.3 Hz, 3H); ^13^C{^1^H} NMR (75 MHz, CDCl_3_) δ 152.3, 148.6, 139.2, 125.6, 124.8, 124.6, 117.7, 111.3, 111.0, 108.5, 80.1, 70.7, 53.0, 52.2, 40.8, 0.0; HRMS (MM: ESI-APCI+) *m*/*z* calcd for C_18_H_20_NO_2_ [M + H]^+^ 282.1489, found 282.1492.

#### Preparation of Alkyne **1c**

##### 1-(Diethoxymethyl)-2-iodobenzene (**S5**)

Synthesized according to the literature.^37^

##### 3-(2-(Diethoxymethyl)phenyl)propanal (**S6**)

Iodide **S5** (5.73 g, 18.7 mmol, 1 equiv) and the allylic alcohol (3.18 mL, 46.8 mmol, 2.5 equiv) were added to a solution of Pd(OAc)_2_ (168 mg, 0.75 mmol, 4 mol %), NaHCO_3_ (7.54 g, 89.8 mmol, 4.8 equiv), and Bu_4_NCl (5.2 g, 18.7 mmol, 1 equiv) in DMF (35 mL). The mixture was stirred at 50 °C in an oil bath for 4 h, and the reaction was filtered through a short plug of silica gel. Then, H_2_O (30 mL) was added to the filtrate and then extracted with EtOAc (2 × 50 mL). The combination of organic layers was washed with H_2_O (2 × 50 mL) and brine (50 mL), dried over Na_2_SO_4_, and concentrated in vacuo. The product was purified by silica gel column chromatography with hexanes/EtOAc (19:1) as the eluent to give the aldehyde **S6**: 86% yield (3.8 g, 16.1 mmol); yellow oil; ^1^H NMR (300 MHz, CDCl_3_) δ 9.83 (t, *J* = 1.4 Hz, 1H), 7.55 (dd, *J* = 7.3, 2.0 Hz, 1H), 7.30–7.20 (m, 2H), 7.17 (dd, *J* = 7.0, 1.6 Hz, 1H), 5.56 (s, 1H), 3.70–3.42 (m, 6H), 3.07 (t, *J* = 7.7 Hz, 2H), 2.84–2.73 (m, 2H), 1.22 (t, *J* = 7.1 Hz, 6H); ^13^C{^1^H} NMR (75 MHz, CDCl_3_) δ 201.8, 138.7, 136.5, 129.6, 128.6, 127.1, 126.1, 100.6, 61.7, 45.4, 24.5, 15.2; MS (CI), *m*/*z* (%) 237 (M^+^ + 1, 100).

##### 1-(But-3-yn-1-yl)-2-(diethoxymethyl)benzene (**S7**).^38^

*n*BuLi (7.73 mL, 19.32 mmol, 1.2 equiv) was added dropwise to a solution of DIPA (2.71 mL, 19.32 mmol, 1.2 equiv) in THF (130 mL) at −78 °C. The reaction mixture was warmed to 0 °C and stirred for 15 min. Then the reaction was cooled to −78 °C, and trimethylsilyldiazomethane (8.05 mL, 16.1 mmol, 1 equiv) was added. The reaction was stirred at −78 °C for 30 min. Then a solution of aldehyde **S6** (3.8 g, 16.1 mmol, 1 equiv) in THF (33 mL) was added. The mixture was stirred for 1 h, and then the reaction was heated to reflux for 3 h. The reaction was quenched with H_2_O (100 mL), and the aqueous layer was extracted with Et_2_O (2 × 60 mL). The combination of organic layers was washed with H_2_O (100 mL), dried over Na_2_SO_4_, and concentrated in vacuo. The residue was purified by silica gel column chromatography with hexanes/EtOAc (99:1) as the eluent to give **S7**: 51% yield (1.92 g, 8.26 mmol); colorless oil; ^1^H NMR (300 MHz, CDCl_3_) δ 7.57 (d, *J* = 7.6 Hz, 1H), 7.33–7.17 (m, 3H), 5.61 (s, 1H), 3.70–3.43 (m, 4H), 2.98 (t, *J* = 7.7 Hz, 2H), 2.51 (td, *J* = 7.7, 2.7 Hz, 12H), 1.99 (t, *J* = 2.7 Hz, 1H), 1.24 (t, *J* = 7.0 Hz, 3H); ^13^C{^1^H} NMR (75 MHz, CDCl_3_) δ 138.6, 136.5, 129.8, 128.4, 126.8, 126.2, 100.3, 84.1, 68.8, 61.7, 20.2, 15.2; MS (CI), *m*/*z* (%) 233 (M^+^ + 1, 100).

##### 1-(Diethoxymethyl)-2-(pent-3-yn-1-yl)benzene (**S8**)

*n*BuLi (3.2 mL, 8 mmol, 1.1 equiv) was added dropwise at −78 °C to a solution of **S7** (1.7 g, 7.3 mmol, 1 equiv) in THF (0.3 M). The mixture was stirred for 50 min at −78 °C, then MeI (2.27 mL, 36.5 mmol, 5 equiv) was added, and the reaction was stirred at rt for 16 h. The reaction was quenched with a saturated solution of NH_4_Cl (aq). The aqueous layer was extracted with EtOAc (40 mL), and the combination of organic layers was washed with brine (100 mL), dried over Na_2_SO_4_, and concentrated in vacuo. The residue was purified by silica gel column chromatography with hexanes/EtOAc (99:1) as the eluent to give **S8**: 92% yield (1.65 g, 6.7 mmol); colorless oil; ^1^H NMR (300 MHz, CDCl_3_) δ 7.58 (dd, *J* = 7.9, 1.8 Hz, 1H), 7.35–7.16 (m, 3H), 5.63 (s, 1H), 3.67–3.45 (m, 4H), 2.92 (t, *J* = 7.7 Hz, 2H), 2.55–2.33(m, 2H), 1.78 (t, *J* = 2.6 Hz, 3H), 1.23 (t, *J* = 7.1 Hz, 6H); ^13^C{^1^H} NMR (75 MHz, CDCl_3_) δ 139.1, 136.5, 129.6, 128.3, 126.6, 126.0, 100.1, 78.8, 76.0, 61.6, 31.7, 20.6, 15.2, 3.5; MS (CI), *m*/*z* (%) 247 (M^+^ + 1, 100).

##### 2-(Pent-3-yn-1-yl)benzaldehyde (**S9**)

A mixture of **S8** (1.65 g, 6.7 mmol, 1 equiv) and PPTS (505 mg, 2.01 mmol, 0.3 equiv) in acetone (260 mL) and H_2_O (7 mL) was heated to reflux in an oil bath for 15 h. The volatiles were removed under a vacuum, the residue was dissolved in DCM (30 mL), and the solution was washed with H_2_O (30 mL). The aqueous layer was extracted with DCM (3 × 20 mL), and the combination of organic layers was washed with brine, dried over Na_2_SO_4_, and concentrated in vacuo. The residue was purified by silica gel column chromatography with hexanes/EtOAc (98:2) as the eluent to give **S9**: 94% yield (1.08 g, 6.3 mmol); colorless oil; ^1^H NMR (300 MHz, CDCl_3_) δ 10.26 (s, 1H), 7.84 (d, *J* = 7.6 Hz, 1H), 7.51 (t, *J* = 7.1 Hz, 1H), 7.38 (t, *J* = 7.5 Hz, 1H), 7.30 (d, *J* = 7.6 Hz, 1H), 3.19 (t, *J* = 7.2 Hz, 2H), 2.58–2.29 (m, 2H), 1.71 (t, *J* = 2.5 Hz, 3H); ^13^C{^1^H} NMR (75 MHz, CDCl_3_) δ 192.0, 143.3, 134.1, 133.7, 131.6, 131.2, 126.9, 77.8, 77.4, 31.5, 21.0, 3.4; MS (CI), *m*/*z* (%) 173 (M^+^ + 1, 100).

##### 4-Methoxy-*N*-(2-(pent-3-yn-1-yl)benzyl)aniline (**1c**):

used general procedure for reductive amination, 85% yield (1.5 g, 5.4 mmol); amorphous off-white solid; ^1^H NMR (300 MHz, CDCl_3_) δ 7.41 (d, *J* = 7.2 Hz, 1H), 7.32–7.19 (m, 3H), 6.89–6.80 (m, 2H), 6.71–6.60 (m, 2H), 4.31 (s, 2H), 3.79 (s, 3H), 3.69 (bs, 1H), 2.94 (t, *J* = 7.5 Hz, 2H), 2.51 (ddt, *J* = 7.5, 5.1, 2.6 Hz, 2H), 1.82 (t, *J* = 2.6 Hz, 3H); ^13^C{^1^H} NMR (75 MHz, CDCl_3_) δ 152.2, 142.7, 139.3, 137.1, 129.5, 128.9, 127.6, 126.7, 115.0, 114.0, 78.7, 76.5, 55.8, 46.9, 31.7, 20.6, 3.6; HRMS (MM: ESI-APCI+) *m*/*z* calcd for C_19_H_22_N_2_O [M + H]^+^ 280.1696, found 280.1701.

#### General Procedure for the Racemic Cyclization of Alkynes **1**

A 5 mL sealed tube equipped with stirring magnetic bar was flamed-dried under a vacuum, cooled to rt, and backfilled with argon. Then it was charged with [Rh(cod)Cl]_2_ (4 mg, 8 μmol, 0.04 equiv), *rac*-BNP acid (5.6 mg, 16 μmol, 0.08 equiv), and *rac*-BINAP (10 mg, 16 μmol, 0.08 equiv). Afterward, it was put in a vacuum and backfilled with argon three times. Then 0.5 mL of DCE was added, and the mixture was stirred for 10 min at rt. Finally, the alkyne **1** (0.2 mmol, 1 equiv) was added under a flow of argon, and the mixture was stirred at 70 °C in an oil bath for 24 h. After cooling at rt, the solvent was evacuated in vacuo, and the residue was purified by silica gel column chromatography with EtOAc/Hexanes (1:9) as the eluent to give the desired seven-membered heterocycle **2**.

##### 1-Tosyl-3-vinyl-1,2,3,5-tetrahydrobenzo[*e*][1,4]oxazepine (**2a**):

85% yield, colorless oil (amorphous off-white solid at 4 °C); ^1^H NMR (300 MHz, CDCl_3_) δ 7.65–7.58 (m, 2H), 7.40–7.34 (m, 1H), 7.32–7.18 (m, 5H), 5.74 (ddd, *J* = 17.4, 10.7, 5.3 Hz, 1H), 5.37 (t, *J* = 1.5 Hz, 1H), 5.31 (t, *J* = 1.5 Hz, 1H), 5.22 (t, *J* = 1.4 Hz, 1H), 5.19 (t, *J* = 1.5 Hz, 1H), 4.49 (d, *J* = 13.4 Hz, 1H), 4.38 (dd, *J* = 15.1, 1.9 Hz, 1H), 4.27–4.13 (m, 1H), 4.17 (d, *J* = 13.4 Hz, 1H), 2.98 (dd, *J* = 15.1, 10.3 Hz, 1H), 2.43 (s, 3H); ^13^C{^1^H} NMR (75 MHz, CDCl_3_) δ 143.7, 139.7, 138.5, 138.2, 135.1, 129.8, 129.6, 128.9, 128.8, 128.0, 127.1, 117.2, 80.7, 72.2, 55.6, 21.6; HRMS (MM: ESI-APCI+) *m*/*z* calcd for C_18_H_19_NO_3_S [M + H]^+^ 330.1158, found 330.1158.

##### 4-(4-Methoxyphenyl)-3-vinyl-2,3,4,5-tetrahydrobenzo[*f*][1,4]oxazepine (**2b**):

41% yield, colorless oil; ^1^H NMR (300 MHz, CDCl_3_) δ 7.23 (dd, *J* = 7.3, 1.7 Hz, 1H), 7.14 (td, *J* = 8.0, 1.8 Hz, 1H), 6.99 (td, *J* = 7.4, 1.3 Hz, 1H), 6.86 (dd, *J* = 8.1, 1.3 Hz, 1H), 6.75 (d, *J* = 9.2 Hz, 2H), 6.67 (d, *J* = 9.2 Hz, 2H), 5.90 (ddd, *J* = 17.2, 10.4, 4.1 Hz, 1H), 5.44 (dt, *J* = 17.2, 1.8 Hz, 1H), 5.33 (dt, *J* = 10.4, 1.8 Hz, 1H), 4.93 (d, *J* = 17.1 Hz, 1H), 4.61–4.50 (m, 1H), 4.36–4.24 (m, 3H), 3.71 (s, 3H).^13^C{^1^H} NMR (75 MHz, CDCl_3_) δ 158.4, 151.9, 144.6, 133.4, 129.3, 128.1, 127.9, 122.2, 119.4, 116.9, 114.7, 114.3, 72.8, 64.0, 55.8, 48.2; HRMS (MM: ESI-APCI+) *m*/*z* calcd for C_18_H_20_NO_2_ [M + H]^+^ 282.1489, found 282.1493.

##### 2-(4-Methoxyphenyl)-3-vinyl-2,3,4,5-tetrahydro-1*H*-benzo[*c*]azepine (**2c**):

57% yield, colorless oil; ^1^H NMR (300 MHz, CDCl_3_) δ 7.29 (d, *J* = 6.9 Hz, 1H), 7.24–7.12 (m, 2H), 7.09 (d, *J* = 6.9 Hz, 1H), 6.75(d, *J* = 9.2 Hz, 2H), 6.65 (d, *J* = 9.2 Hz, 2H), 5.99 (ddd, *J* = 17.2, 10.4, 3.6 Hz, 1H), 5.28–5.15 (m, 2H), 4.75 (d, *J* = 17.1 Hz, 1H), 4.36–4.25 (m, 1H), 4.24 (d, *J* = 17.0 Hz, 1H) 3.72 (s, 3H), 3.04–2.80 (m, 2H), 2.36–2.21 (m, 1H), 2.15–1.96 (m, 1H); ^13^C{^1^H} NMR (75 MHz, CDCl_3_) δ 151.1, 144.9, 139.8, 139.4, 137.4, 130.2, 127.8, 126.7, 126.0, 114.8, 114.4, 112.9, 62.0, 55.8, 49.3, 32.5, 32.4; HRMS (MM: ESI-APCI+) *m*/*z* calcd for C_19_H_22_NO [M + H]^+^: 280.1696, found 280.1695.

##### 2-(4-Methoxyphenyl)-3-(prop-1-en-1-yl)-1,2,3,4-tetrahydroisoquinoline (**2c′**):

20% yield, colorless oil (mixture of isomers); ^1^H NMR mixture of isomers (300 MHz, CDCl_3_) δ 7.15–7.02 (m, 4H isom 1, 4H isom 2), 6.91–6.82 (m, 2H isom 1, 2H isom 2), 6.82–6.74 (m, 2H isom 1, 2H isom 2), 5.49–5.22 (m, 1H isom 1, 1H isom 2), 4.55 (dt, *J* = 8.8, 4.4 Hz, 1H isom 2), 4.41–4.31 (m, 1H isom 2), 4.27 (d, *J* = 15.5 Hz, 1H isom 1, 1H isom 2), 4.20 (d, *J* = 15.6 Hz, 1H isom 1, 1H isom 2), 3.71 (s, 3H isom 1, 3H isom 2), 3.17 (dd, *J* = 15.8, 5.4 Hz, 1H isom 1, 1H isom 2), 2.77 (dd, *J* = 15.8, 3.1 Hz, 1H isom 1), 2.70 (dd, *J* = 15.9, 4.3 Hz, 1H isom 2), 1.55 (dd, *J* = 6.8, 1.6 Hz, 3H isom 2), 1.46 (dt, *J* = 6.0, 1.1 Hz, 3H isom 1); ^13^C{^1^H} NMR (75 MHz, CDCl_3_) δ 153.7, 152.8, 144.5, 144.1, 134.4, 134.3, 133.6, 133.5, 129.8, 129.3, 128.8, 128.7, 127.4, 126.3, 126.2, 125.9, 125.8, 119.3, 117.5, 114.5, 114.4, 56.9, 55.6, 55.6, 53.0, 49.9, 48.1, 35.3, 34.4, 17.8, 13.4; HRMS (MM: ESI-APCI+) *m*/*z* calcd for C_19_H_22_NO [M + H]^+^ 280.1696, found 280.1695.

##### *tert*-Butyl 4-(4-Methoxyphenyl)-3-vinyl-2,3,4,5-tetrahydro-1*H*-benzo[*e*][1,4]diazepine-1-carboxylate (**2d**)

70% yield, amorphous off-white solid; ^1^H NMR (300 MHz, DMSO-*d*_6_, 80 °C) δ 7.43–7.30 (m, 1H), 7.29–7.09 (m, 3H), 6.70 (d, *J* = 9.1 Hz, 2H), 6.62 (d, *J* = 9.1 Hz, 2H), 6.03 (ddd, *J* = 17.2, 10.5, 4.7 Hz, 1H), 5.42–5.19 (m, 2H), 4.81–4.60 (m, 1H), 4.68 (d, *J* = 17.3 Hz, 1H), 4.39 (d, *J* = 17.3 Hz, 1H), 4.07 (dd, *J* = 14.6, 5.2 Hz, 1H), 3.62 (s, 3H), 3.48 (dd, *J* = 14.6, 9.9 Hz, 1H), 1.22 (s, 9H); ^13^C{^1^H} NMR (75 MHz, DMSO-*d*_6_, 80 °C) δ 152.5, 151.2, 143.8, 140.7, 135.3, 132.8, 127.8, 126.9, 125.7, 124.9, 115.4, 114.3, 113.9, 79.3, 59.4, 55.2, 51.3, 48.1, 27.3; HRMS (MM: ESI-APCI+) *m*/*z* calcd for C_23_H_28_N_2_O_3_Na [M + Na]^+^ 403.1992, found 403.1990.

#### General Procedure for the Asymmetric Cyclization of Alkynes **1**

A 5 mL sealed tube equipped with stirring magnetic bar was flamed-dried under a vacuum, cooled to rt, and backfilled with argon. Then, it was charged with [Rh(cod)Cl]_2_ (4 mg, 8 μmol, 0.04 equiv), Brønsted acid (16 μmol, 0.08 equiv), and chiral ligand (16 μmol, 0.08 equiv). Afterward, it was put in a vacuum and backfilled with argon three times. Then, 0.5 mL of DCE was added, and the mixture was stirred for 10 min at rt. Finally, the alkyne **1** (0.2 mmol, 1 equiv) was added under a flow of argon, and the mixture was stirred at 50 °C in an oil bath for 24 h. After cooling at rt and stripping off the solvent, the resulting residue was purified by silica gel column chromatography with EtOAc/Hexanes (1:9) as the eluent to give the desired seven-membered heterocycle **2**.

#### General Procedure for the Preparation of Allenes **3d**–**3f**, **3h**–**3j**, and **3n**

Tosyl derivatives **S 1f**,^39^**S1h**,^40^**S1i**,^41^**S1j**,^42^ and **S1n**(43) were synthesized according to literature procedures.

See the general procedure for N-alkylation of alkynes.

##### Ethyl 2-((*tert*-Butoxycarbonyl)(prop-2-yn-1-yl)amino)benzoate (**S10d**)

94% yield (2.85 g, 9.4 mmol), colorless oil; ^1^H NMR (300 MHz, CDCl_3_) δ 7.96 (dd, *J* = 7.7, 1.8 Hz, 1H), 7.65–7.33 (m, 3H), 4.82 (dd, *J* = 17.7, 2.5 Hz, 1H), 4.34 (dtd, *J* = 9.7, 7.1, 3.4 Hz, 2H), 4.03 (dd, *J* = 17.7, 2.5 Hz, 1H), 2.33–2.15 (m, 1H), 1.60 (s, 2H), 1.39 (t, *J* = 7.2 Hz, 3H), 1.31 (s, 8H); ^13^C{^1^H} NMR (75 MHz, CDCl_3_) δ 166.0, 154.0, 153.7, 141.6, 141.1, 132.6, 132.5, 131.0, 129.8, 129.5, 129.1, 127.3, 81.0, 80.4, 80.0, 79.8, 72.2, 71.8, 61.1, 60.9, 40.2, 39.1, 28.2, 28.0, 14.1; MS (CI), *m*/*z* (%) 304 (M^+^ + 1, 100).

##### Ethyl 2-((4-Methyl-*N*-(prop-2-yn-1-yl)phenyl)sulfonamido)benzoate (**S10e**)

90% yield (3.2 g, 9 mmol); amorphous yellow solid; ^1^H NMR (300 MHz, CDCl_3_) δ 7.94–7.83 (m, 1H), 7.66–7.54 (m, 2H), 7.51–7.35 (m, 2H), 7.30–7.13 (m, 3H), 5.02–4.35 (m, 2H), 4.28 (s, 2H), 2.42 (s, 3H), 2.21 (t, *J* = 2.5 Hz, 1H), 1.37 (t, *J* = 7.1 Hz, 3H); ^13^C{^1^H} NMR (75 MHz, CDCl_3_) δ 166.0, 143.4, 137.4, 137.1, 132.9, 132.1, 131.9, 131.3, 129.3, 129.0, 127.7, 73.6, 61.5, 41.3, 21.6, 14.1; MS (CI), *m*/*z* (%) 358 (M^+^ + 1, 100).

##### Methyl 4-Methyl-2-((4-methyl-*N*-(prop-2-yn-1-yl)phenyl)sulfonamido)benzoate (**S10f**)

90% yield (3.21 g, 9 mmol); amorphous off-white solid; ^1^H NMR (300 MHz, CDCl_3_) δ 7.73 (d, *J* = 7.9 Hz, 1H), 7.60–7.50 (m, 2H), 7.20 (dd, *J* = 8.1, 4.0 Hz, 3H), 7.01 (d, *J* = 1.7 Hz, 1H), 4.64 (s, 1H), 3.69 (s, 3H), 2.38 (s, 3H), 2.28 (s, 3H); ^13^C{^1^H} NMR (75 MHz, CDCl_3_) δ 166.1, 143.3, 143.2, 137.5, 137.2, 133.0, 131.3, 129.7, 129.3, 129.0, 128.9, 127.9, 127.6, 78.9, 73.4, 52.1, 41.3, 21.5, 21.3; MS (CI), *m*/*z* (%) 358 (M^+^ + 1, 100).

##### Methyl 2-((4-Methyl-*N*-(prop-2-yn-1-yl)phenyl)sulfonamido)-4-(trifluoromethyl)benzoate (**S10h**)

84% yield (1.8 g, 4.5 mmol); amorphous yellow solid; ^1^H NMR (300 MHz, CDCl_3_) δ 8.0 (d, *J* = 8.1 Hz, 1H), 7.7 (d, *J* = 8.1 Hz, 1H), 7.6 (d, *J* = 8.0 Hz, 2H), 7.4 (s, 1H), 7.3–7.1 (m, 2H), 4.6 (s, 2H), 3.9 (s, 3H), 2.4 (s, 3H), 2.2 (d, *J* = 2.5 Hz, 1H); ^13^C{^1^H} NMR (75 MHz, CDCl_3_) δ 165.5, 144.2, 138.3, 136.5 (d, *J* = 8.1 Hz), 133.9 (q, *J* = 33.6 Hz), 131.8, 129.6, 129.1 (d, *J* = 3.8 Hz), 127.9, 125.8 (d, *J* = 3.9 Hz), 124.8, 121.2, 74.5, 52.9, 41.3, 21.7; ^19^F NMR (282 MHz, CDCl_3_) δ −63.2; MS (CI), *m*/*z* (%) 412 (M^+^ + 1, 100).

##### Methyl 5-Methoxy-2-((4-methyl-*N*-(prop-2-yn-1-yl)phenyl)sulfonamido)benzoate (**S10i**)

83% yield (0.62 g, 1.7 mmol); yellow oil; ^1^H NMR (300 MHz, CDCl_3_) δ 7.6 (d, *J* = 7.9 Hz, 2H), 7.3 (d, *J* = 3.0 Hz, 1H), 7.2 (d, *J* = 7.9 Hz, 2H), 7.1 (d, *J* = 8.7 Hz, 1H), 6.9 (dd, *J* = 8.8, 3.1 Hz, 1H), 4.9 (s, 1H), 4.3 (s, 1H), 3.8 (d, *J* = 13.9 Hz, 6H), 2.4 (s, 3H), 2.2 (s, 1H); ^13^C{^1^H} NMR (75 MHz, CDCl_3_) δ 166.3, 159.6, 143.2, 137.3, 133.54, 133.4, 129.9, 129.4, 127.8, 117.8, 116.0, 79.1, 73.6, 55.8, 52.5, 41.5, 21.6; MS (CI), *m*/*z* (%) 423 (M^+^ + 1, 100).

##### Methyl 5-Bromo-2-((4-methyl-*N*-(prop-2-yn-1-yl)phenyl)sulfonamido)benzoate (**S10j**)

70% yield (2.95 g, 7 mmol); amorphous off-white solid; ^1^H NMR (300 MHz, CDCl_3_) δ 7.90 (d, *J* = 2.5 Hz, 1H), 7.48 (dd, *J* = 8.3, 6.3 Hz, 3H), 7.16 (d, *J* = 8.1 Hz, 2H), 6.98 (d, *J* = 8.3 Hz, 1H), 4.49 (s, 1H), 3.71 (s, 3H), 2.33 (s, 3H), 2.16 (t, *J* = 2.3 Hz, 1H); ^13^C{^1^H} NMR (75 MHz, CDCl_3_) δ 164.9, 143.8, 136.8, 136.6, 135.3, 134.2, 134.1, 133.6, 129.5, 127.7, 123.1, 78.5, 74.1, 52.7, 41.2, 21.6.MS (CI), *m*/*z* (%) 374 (M^+^ + 1, 100).

##### Methyl 4,5-Dimethoxy-2-((4-methyl-*N*-(prop-2-yn-1-yl)phenyl)sulfonamido)benzoate (**S10n**)

99% yield (0.8 g, 1.98 mmol); white foam; ^1^H NMR (300 MHz, CDCl_3_) δ 7.59 (d, *J* = 7.9 Hz, 2H), 7.36 (s, 1H), 7.23 (d, *J* = 7.9 Hz, 2H), 6.67 (s, 1H), 4.88 (s, 1H), 4.27 (s, 1H), 3.90 (s, 3H), 3.70 (d, *J* = 2.9 Hz, 6H), 2.40 (s, 3H), 2.23 (t, *J* = 2.5 Hz, 1H); ^13^C{^1^H} NMR (75 MHz, CDCl_3_) δ 165.7, 151.5, 148.8, 143.5, 137.4, 131.5, 129.3, 127.9, 123.9, 115.4, 113.2, 79.3, 73.5, 56.2, 56.0, 52.2, 41.4, 21.6; MS (CI), *m*/*z* (%) 404 (M^+^ + 1, 100).

#### Homologation of Alkynes to Allenes

CuI (0.5 equiv), (CHO)_*n*_ (2.5 equiv), and Cy_2_NH (1.8 equiv) were added to a stirred solution of the alkyne **S10** (1 equiv) in dioxane (0.2 M). The reaction was then heated to reflux in an oil bath for 6 h. Then, the reaction was cooled to rt, and the solvent was removed in vacuo. The residue was dissolved in CHCl_3_ (50 mL) and washed with 10% NH_4_OH (2 × 50 mL) and H_2_O (2 × 50 mL). The combination of organic layers was dried over Na_2_SO_4_ and concentrated in vacuo. The residue was purified by silica gel column chromatography with hexanes/EtOAc (8:2) as the eluent to give the allenes **S11**.

##### Ethyl 2-(Buta-2,3-dien-1-yl(*tert*-butoxycarbonyl)amino)benzoate (**S11d**):

82% yield (2.44 g, 7.7 mmol); yellow oil; ^1^H NMR (300 MHz, CDCl_3_) δ 7.89 (d, *J* = 8.0 Hz, 1H), 7.47 (t, *J* = 7.6 Hz, 1H), 7.35–7.17 (m, 2H), 5.30 (p, *J* = 6.6 Hz, 1H), 4.78–4.60 (m, 2H), 4.58–4.41 (m, 1H), 4.38–4.21 (m, 2H), 3.94–3.79 (m, 1H), 1.50 (s, 3H), 1.36; ^13^C{^1^H} NMR (75 MHz, CDCl_3_) δ 209.1, 166.3, 153.9, 141.9, 132.3, 131.0, 129.2, 126.7, 87.4, 80.0, 75.8, 61.1, 49.1, 28.0, 14.2; MS (CI), *m*/*z* (%) 318 (M^+^ + 1, 100).

##### Ethyl 2-((*N*-(Buta-2,3-dien-1-yl)-4-methylphenyl)sulfonamido)benzoate (**S11e**):

90% yield (3 g, 8.1 mmol); amorphous white solid; ^1^H NMR (300 MHz, CDCl_3_) δ 7.88–7.80 (m, 1H), 7.52–7.44 (m, 2H), 7.36 (hept, *J* = 5.3 Hz, 2H), 7.19 (d, *J* = 8.0 Hz, 2H), 6.97–6.88 (m, 1H), 5.21 (p, *J* = 7.0 Hz, 1H), 4.55 (dt, *J* = 6.6, 2.3 Hz, 2H), 4.38–4.15 (m, 5H), 2.36 (s, 4H), 1.34 (t, *J* = 7.2 Hz, 3H); ^13^C{^1^H} NMR (75 MHz, CDCl_3_) δ 209.6, 166.18, 143.3, 137.7, 136.9, 133.1, 131.8, 131.3, 130.9, 129.4, 128.4, 127.5, 86.6, 75.9, 61.3, 50.9, 21.5, 14.1; MS (CI), *m*/*z* (%) 372 (M^+^ + 1, 100).

##### Methyl 2-((*N*-(Buta-2,3-dien-1-yl)-4-methylphenyl)sulfonamido)-4-methylbenzoate (**S11f**):

85% yield (2.84 g, 7.65 mmol); amorphous white solid; ^1^H NMR (300 MHz, CDCl_3_) δ 7.74 (d, *J* = 7.9 Hz, 1H), 7.52 (d, *J* = 8.0 Hz, 2H), 7.20 (dd, *J* = 14.1, 7.9 Hz, 3H), 6.86 (s, 1H), 5.37–5.08 (m, 1H), 4.58 (dt, *J* = 5.8, 2.4 Hz, 2H), 4.27 (s, 2H), 3.68 (d, *J* = 1.1 Hz, 3H), 2.39 (s, 3H), 2.30 (s, 3H); ^13^C{^1^H} NMR (75 MHz, CDCl_3_) δ 209.6, 166.3, 143.2, 142.9, 137.9, 137.2, 132.5, 131.4, 129.4, 129.1, 129.1, 127.5, 86.8, 75.9, 51.9, 51.0, 21.5, 21.3; MS (CI), *m*/*z* (%) 372 (M^+^ + 1, 100).

##### Methyl 2-((*N*-(Buta-2,3-dien-1-yl)-4-methylphenyl)sulfonamido)-4-(trifluoromethyl)benzoate (**S11h**):

70% yield (1.3 g, 3 mmol); brown oil; ^1^H NMR (500 MHz, CDCl_3_) δ 8.0 (dd, *J* = 8.2, 1.0 Hz, 1H), 7.6 (ddd, *J* = 8.2, 1.8, 0.8 Hz, 1H), 7.5–7.5 (m, 2H), 7.3–7.2 (m, 2H), 7.1 (d, *J* = 1.8 Hz, 1H), 5.2 (ddd, *J* = 7.4, 6.6, 0.9 Hz, 1H), 4.6 (dt, *J* = 6.6, 2.3 Hz, 2H), 4.3 (dt, *J* = 7.4, 2.3 Hz, 3H), 3.9 (s, 4H), 2.4 (s, 4H); ^13^C{^1^H} NMR (126 MHz, CDCl_3_) δ 210.2, 165.7, 144.0, 138.7, 133.7 (q, *J* = 33.2 Hz), 131.8, 129.8, 128.0 (q, *J* = 3.6 Hz), 127.6, 125.1 (q, *J* = 3.7 Hz), 123.1 (q, *J* = 272.8 Hz), 86.1, 76.2, 52.8, 50.9, 21.6; ^19^F NMR (282 MHz, CDCl_3_) δ −63.0; MS (CI), *m*/*z* (%) 426 (M^+^ + 1, 100).

##### Methyl 2-((*N*-(Buta-2,3-dien-1-yl)-4-methylphenyl)sulfonamido)-5-methoxybenzoate (**S11i**):

70% yield (0.45 g, 1.16 mmol); brown oil; ^1^H NMR (500 MHz, CDCl_3_) δ 7.52 (d, *J* = 8.0 Hz, 2H), 7.34 (d, *J* = 2.6 Hz, 1H), 7.23 (d, *J* = 7.9 Hz, 2H), 6.94–6.90 (m, 2H), 5.22 (q, *J* = 6.9 Hz, 1H), 4.60 (d, *J* = 6.5 Hz, 2H), 4.34 (s, 1H), 4.21 (s, 1H), 3.83 (s, 3H), 3.75 (s, 3H), 2.41 (s, 3H); ^13^C{^1^H} NMR (126 MHz, CDCl_3_) δ 209.8, 166.4, 159.1, 143.2, 137.3, 133.5, 132.8, 130.3, 129.5, 127.6, 117.9, 115.9, 86.8, 75.9, 55. 8, 52.4, 51.7, 21.6; MS (CI), *m*/*z* (%) 388 (M^+^ + 1, 100).

##### Methyl 5-Bromo-2-((*N*-(buta-2,3-dien-1-yl)-4-methylphenyl)sulfonamido)benzoate (**S11j**):

80% yield (2.44 g, 5.6 mmol); amorphous white solid; ^1^H NMR (300 MHz, CDCl_3_) δ 7.96 (d, *J* = 2.4 Hz, 1H), 7.50 (dd, *J* = 11.0, 8.3 Hz, 3H), 7.22 (d, *J* = 8.0 Hz, 2H), 6.85 (d, *J* = 8.5 Hz, 1H), 5.19 (p, *J* = 7.0 Hz, 1H), 4.58 (dd, *J* = 6.6, 2.4 Hz, 2H), 4.25 (dd, *J* = 7.4, 2.4 Hz, 2H), 3.75 (s, 3H), 2.38 (s, 3H); ^13^C{^1^H} NMR (75 MHz, CDCl_3_) δ 209.8, 165.1, 143.6, 136.9, 136.6, 135.0, 134.3, 134.2, 132.79, 129.6, 127.5, 122.3, 86.4, 76.1, 52.5, 50.9, 21.5; MS (CI), *m*/*z* (%) 437 (M^+^1, 100).

##### Methyl 2-((*N*-(Buta-2,3-dien-1-yl)-4-methylphenyl)sulfonamido)-4,5-dimethoxybenzoate (**S11n**):

56% yield (0.46 g, 1.1 mmol); brown foam; ^1^H NMR (500 MHz, CDCl_3_) δ 7.55 (d, *J* = 8.3 Hz, 2H), 7.37 (s, 1H), 7.31–7.18 (m, 3H), 6.52 (s, 1H), 5.26 (t, *J* = 6.9 Hz, 1H), 4.61 (s, 2H), 4.40 (s, 1H), 4.15 (s, 1H), 3.92 (s, 4H), 3.75 (s, 4H), 3.66 (s, 3H), 2.40 (s, 3H); ^13^C{^1^H} NMR (126 MHz, CDCl_3_) δ 209.8, 165.9, 151.5, 148.5, 143.2, 137.5, 132.0, 129.4, 127.7, 124.0, 115.0, 113.4, 87.0, 76.0, 56.2, 56.2, 52.1, 51.4, 21.6; MS (CI), *m*/*z* (%) 418 (M^+^ + 1, 100).

See the general procedure for the ester reduction of alkynes.

##### *N*-(Buta-2,3-dien-1-yl)-*N*-(2-(hydroxymethyl)phenyl)-4-methylbenzenesulfonamide (**3a**):

94% yield (2.5 g, 7.6 mmol); amorphous white solid; ^1^H NMR (300 MHz, CDCl_3_) δ 7.63–7.50 (m, 3H), 7.39–7.24 (m, 3H), 7.14 (td, *J* = 7.7, 1.7 Hz, 1H), 6.46 (dd, *J* = 8.0, 1.3 Hz, 1H), 5.10–4.93 (m, 2H), 4.66–4.38 (m, 4H), 3.84 (dd, *J* = 13.8, 8.3 Hz, 1H), 3.09–2.97 (m, 1H), 2.45 (s, 3H); ^13^C{^1^H} NMR (75 MHz, CDCl_3_) δ 209.9, 144.0, 142.4, 136.9, 134.8, 131.0, 129.6, 129.1, 128.3, 128.1, 127.7, 85.3, 76.1, 61.2, 51.4, 21.6; HRMS (MM: ESI-APCI+) *m*/*z* calcd for C_18_H_18_NO_2_S [M – H_2_O]^+^: 312.1053, found 312.1059.

##### *tert*-Butyl Buta-2,3-dien-1-yl(2-(hydroxymethyl)phenyl)carbamate (**S12d**):

88% yield; colorless oil; ^1^H NMR (300 MHz, DMSO-*d*_6_, 80 °C) δ 7.52 (d, *J* = 7.1 Hz, 1H), 7.34–7.17 (m, 2H), 7.12 (dd, *J* = 7.4, 1.7 Hz, 1H), 5.26 (p, *J* = 6.6 Hz, 1H), 4.90–4.69 (m, 3H), 4.45 (d, *J* = 5.3 Hz, 2H), 4.06 (bs, 2H), 1.35 (s, 9H); ^13^C{^1^H} NMR (75 MHz, DMSO-*d*_6_, 80 °C) δ 208.2, 153.2, 139.4, 139.0, 127.5, 127.1, 126.6, 126.5, 86.6, 78.9, 75.7 58.7, 48.3, 27.5; MS (CI), *m*/*z* (%) 276 (M^+^ – [OH], 100).

##### *N*-(Buta-2,3-dien-1-yl)-*N*-(2-(hydroxymethyl)-5-methylphenyl)-4-methylbenzenesulfonamide (**S12f**):

90% yield (2.37 g, 6.9 mmol); amorphous white solid; ^1^H NMR (300 MHz, CDCl_3_) δ 7.56 (d, *J* = 8.0 Hz, 2H), 7.47 (d, *J* = 7.8 Hz, 1H), 7.31 (d, *J* = 7.9 Hz, 2H), 7.16 (d, *J* = 7.8 Hz, 1H), 6.24 (s, 1H), 5.09–4.97 (m, 1H), 4.93 (d, *J* = 11.9 Hz, 1H), 4.61 (d, *J* = 7.1 Hz, 1H), 4.57–4.37 (m, 3H), 3.88–3.73 (m, 1H), 2.46 (s, 3H), 2.16 (s, 3H); ^13^C{^1^H} NMR (75 MHz, CDCl_3_) δ 209.9, 144.1, 139.2, 138.3, 136.9, 134.8, 130.9, 129.9, 129.5, 128.3, 128.2, 85.5, 76.2, 61.1, 51.4, 21.7, 20.9; MS (CI), *m*/*z* (%) 326 M^+^ – [OH], 100).

##### *N*-(Buta-2,3-dien-1-yl)-*N*-(2-(hydroxymethyl)-5-(trifluoromethyl)phenyl)-4-methylbenzenesulfonamide (**S12h**):

64% yield (0.7 g, 1.86 mmol); amorphous yellow solid; ^1^H NMR (500 MHz, CDCl_3_) δ 7.8 (d, *J* = 8.0 Hz, 1H), 7.6 (d, *J* = 8.1 Hz, 1H), 7.5 (dd, *J* = 8.2, 1.7 Hz, 2H), 7.3 (d, *J* = 7.8 Hz, 2H), 6.6 (s, 1H), 5.1–5.0 (m, 2H), 4.7–4.6 (m, 2H), 4.5 (q, *J* = 10.1 Hz, 2H), 3.8 (dd, *J* = 13.6, 8.5 Hz, 1H), 2.5 (s, 3H); ^13^C{^1^H} NMR (126 MHz, CDCl_3_) δ 210.5, 146.8, 144.8, 137.5, 134.0, 130.4 (q, *J* = 33.0 Hz), 129.9, 128.2, 125.8 (q, *J* = 3.7 Hz), 125.0 (q, *J* = 3.7 Hz), 123.4 (1, *J* = 272.3 Hz), 85.0, 76.4, 61.0, 51.4, 21.7; MS (CI), *m*/*z* (%) 380 M^+^ – [OH], 100).

##### *N*-(Buta-2,3-dien-1-yl)-*N*-(2-(hydroxymethyl)-4-methoxyphenyl)-4-methylbenzenesulfonamide (**S12i**):

54% yield (0.23 g, 0.663 mmol), brown oil; ^1^H NMR (500 MHz, CDCl_3_) δ 7.6–7.5 (m, 2H), 7.3–7.3 (m, 2H), 7.1 (d, *J* = 3.0 Hz, 1H), 6.6 (dd, *J* = 8.8, 3.0 Hz, 1H), 6.4 (d, *J* = 8.8 Hz, 1H), 5.1–5.0 (m, 1H), 4.9 (d, *J* = 12.2 Hz, 1H), 4.6 (dddd, *J* = 11.2, 6.6, 2.9, 1.6 Hz, 1H), 4.6–4.4 (m, 3H), 3.8 (s, 4H), 3.0 (s, 1H), 2.4 (s, 3H); ^13^C{^1^H} NMR (126 MHz, CDCl_3_) δ 210.0, 159.8, 144.0, 143.9, 135.1, 129.7, 129.4, 128.9, 128.2, 115.0, 114.6, 85.5, 76.2, 61.6, 55.6, 51.7, 21.7; MS (CI), *m*/*z* (%) 342 (M^+^ – [OH], 100).

##### *N*-(4-Bromo-2-(hydroxymethyl)phenyl)-*N*-(buta-2,3-dien-1-yl)-4-methylbenzenesulfonamide (**S12j**):

90% yield (2 g, 5 mmol); amorphous white solid; ^1^H NMR (300 MHz, CDCl_3_) δ 7.79 (d, *J* = 2.5 Hz, 1H), 7.56 (d, *J* = 8.1 Hz, 2H), 7.39–7.17 (m, 3H), 6.33 (d, *J* = 8.5 Hz, 1H), 5.09–4.91 (m, 2H), 4.70–4.35 (m, 5H), 3.88–3.75 (m, 1H), 2.48 (s, 3H); ^13^C{^1^H} NMR (75 MHz, CDCl_3_) δ 210.0, 144.7, 144.4, 135.8, 134.4, 133.6, 131.2, 129.8, 129.2, 128.0, 123.0, 85.2, 76.41, 60.8, 51.3, 21.7; MS (CI), *m*/*z* (%) 391 (M^+^ – [OH], 100).

##### *N*-(Buta-2,3-dien-1-yl)-*N*-(2-(hydroxymethyl)-4,5-dimethoxyphenyl)-4-methylbenzenesulfonamide (**S12n**):

57% yield (0.24 g, 0.61 mmol), amorphous white solid; ^1^H NMR (500 MHz, CDCl_3_) δ 7.6–7.5 (m, 2H), 7.3 (d, *J* = 7.9 Hz, 2H), 7.0 (s, 1H), 5.8 (s, 1H), 5.0 (dt, *J* = 7.9, 6.6 Hz, 1H), 4.9 (d, *J* = 11.9 Hz, 1H), 4.7–4.6 (m, 1H), 4.5 (ddt, *J* = 9.4, 6.5, 2.3 Hz, 1H), 4.5 (ddt, *J* = 13.5, 5.9, 2.8 Hz, 1H), 4.4 (d, *J* = 12.0 Hz, 1H), 3.9 (s, 3H), 3.8 (ddt, *J* = 13.7, 8.2, 1.7 Hz, 1H), 3.5 (s, 3H), 2.4 (s, 3H); ^13^C{^1^H} NMR (126 MHz, CDCl_3_) δ 210.1, 149.4, 148.4, 144.2, 135.4, 135.1, 129.7, 128.9, 128.4, 112.9, 110.5, 85.6, 76.3, 61.2, 56.1, 55.8, 51.6, 21.7; MS (CI), *m*/*z* (%) 382 (M^+^ – [OH], 100).

See the general procedure for the oxidation and reductive amination of alkynes.

##### *tert*-Butyl Buta-2,3-dien-1-yl(2-(((4-methoxyphenyl)amino)methyl)phenyl)carbamate (**3d**):

70% yield (1.8 g, 4.8 mmol); yellow oil; ^1^H NMR (300 MHz, DMSO-*d*_6_, 80 °C) δ 7.45–7.36 (m, 1H), 7.30–7.14 (m, 3H), 6.68 (d, *J* = 8.9 Hz, 2H), 6.50 (d, *J* = 8.9 Hz, 2H), 5.46 (bs, 1H), 5.33 (p, *J* = 6.6 Hz, 1H), 4.87–4.76 (m, 2H), 4.25 (bs, 1H), 4.15 (s, 2H), 3.97 (bs, 1H), 364 (s, 3H), 1.40 (s, 9H); ^13^C{^1^H} NMR (75 MHz, DMSO-*d*_6_, 80 °C) δ 208.2, 153.2, 150.8, 142.6, 140.0, 137.3, 127.8, 127.3, 126.7, 114.5, 113.0, 86.7, 79.2, 75.9, 55.2, 48.4, 43.4, 27.6; HRMS (MM: ESI-APCI+) *m*/*z* calcd for C_23_H_29_N_2_O_3_ [M + H]^+^: 381.2173, found 381.2176.

##### *N*-(Buta-2,3-dien-1-yl)-*N*-(2-(((4-methoxyphenyl)amino)methyl)phenyl)-4-methylbenzenesulfonamide (**3e**):

70% yield (1.5 g, 3.5 mmol); amorphous white solid; ^1^H NMR (500 MHz, CDCl_3_) δ 7.62–7.57 (m, 2H), 7.53 (dd, *J* = 7.7, 1.6 Hz, 1H), 7.28 (dd, *J* = 10.4, 7.2 Hz, 3H), 7.12 (td, *J* = 7.7, 1.6 Hz, 1H), 6.80–6.74 (m, 2H), 6.64–6.56 (m, 3H), 5.12 (p, *J* = 6.9 Hz, 1H), 4.70–4.32 (m, 5H), 3.90 (dd, *J* = 13.7, 8.4 Hz, 1H), 3.74 (s, 3H), 2.45 (s, 3H); ^13^C{^1^H} NMR (126 MHz, CDCl_3_) δ 210.0, 152.1, 143.8, 142.6, 141.3, 137.3, 135.5, 129.7, 129.6, 129.3, 128.8, 128.2, 127.9, 127.4, 114.9, 114.3, 85.6, 76.0, 55.8, 51.4, 45.3, 21.6; HRMS (MM: ESI-APCI+) *m*/*z* calcd for C_25_H_27_N_2_O_3_S [M + H]^+^: 435.1737, found 435.1738.

##### *N*-(Buta-2,3-dien-1-yl)-*N*-(2-(((4-methoxyphenyl)amino)methyl)-5-methylphenyl)-4-methylbenzenesulfonamide (**3f**):

80% yield (716 mg, 1.6 mmol), amorphous white solid; ^1^H NMR (300 MHz, CDCl_3_) δ 7.60 (d, *J* = 7.9 Hz, 2H), 7.39 (d, *J* = 7.8 Hz, 1H), 7.29 (d, *J* = 8.0 Hz, 2H), 7.09 (d, *J* = 7.8 Hz, 1H), 6.81 (d, *J* = 9 Hz, 2H), 6.62 (d, *J* = 9 Hz, 2H), 6.41 (s, 1H), 5.11 (p, *J* = 6.9 Hz, 1H), 4.69–4.48 (m, 2H), 4.37 (dd, *J* = 16.7, 6.7 Hz, 3H), 3.88 (dd, *J* = 13.6, 8.5 Hz, 1H), 3.74 (s, 3H), 2.45 (s, 3H), 2.18 (s, 3H); ^13^C{^1^H} NMR (75 MHz, CDCl_3_) δ 210.0, 152.2, 143.9, 142.6, 137.9, 137.4, 137.3, 135.6, 129.7, 129.5, 129.4, 128.9, 128.3, 114.9, 114.5, 85.8, 76.1, 55.9, 51.4, 45.2, 21.7, 20.9; HRMS (MM: ESI-APCI+) *m*/*z* calcd for C_26_H_29_N_2_O_3_S [M + H]^+^ 449.1893, found 449.1896.

##### *N*-(Buta-2,3-dien-1-yl)-*N*-(2-(((4-methoxyphenyl)amino)methyl)-5-(trifluoromethyl)phenyl)-4-methylbenzenesulfonamide (**3h**):

73% yield, (0.7 g, 1.4 mmol), brown oil; ^1^H NMR (500 MHz, CDCl_3_) δ 7.7 (d, *J* = 8.0 Hz, 1H), 7.6–7.5 (m, 3H), 7.3 (d, *J* = 8.0 Hz, 2H), 6.8 (d, *J* = 8.6 Hz, 2H), 6.7 (s, 1H), 6.6 (d, *J* = 8.6 Hz, 2H), 5.2–5.1 (m, 1H), 4.7 (d, *J* = 16.3 Hz, 1H), 4.6 (t, *J* = 8.8 Hz, 1H), 4.6–4.4 (m, 3H), 3.9–3.8 (m, 1H), 3.7 (s, 3H), 2.5 (s, 3H); ^13^C{^1^H} NMR (126 MHz, CDCl_3_) δ 210.5, 152.4, 146.3, 144.6, 142.1, 138.0, 134.5, 129.9, 129.6, 129.6 (q, *J* = 32.9 Hz), 125.5 (q, *J* = 3.7 Hz), 125.2 (q, *J* = 3.7 Hz), 123.6 (q, *J* = 272.2 Hz), 114.4, 85.2, 76.3, 55.9, 51.4, 45.4, 21.7; ^19^F NMR (282 MHz, CDCl_3_) δ −62.5; HRMS (MM: ESI-APCI+) *m*/*z* calcd for C_26_H_26_F_3_N_2_O_3_S [M + H]^+^ 503.1649, found 503.1639.

##### *N*-(Buta-2,3-dien-1-yl)-*N*-(4-methoxy-2-(((4-methoxyphenyl)amino)methyl)phenyl)-4-methylbenzenesulfonamide (**3i**):

50% yield, (0.26 g, 0.56 mmol), brown oil; ^1^H NMR (500 MHz, CDCl_3_) δ 7.7–7.5 (m, 2H), 7.3 (dt, *J* = 10.8, 5.3 Hz, 2H), 7.1 (d, *J* = 3.1 Hz, 1H), 6.8–6.7 (m, 2H), 6.7–6.6 (m, 3H), 6.6–6.5 (m, 1H), 5.2–5.0 (m, 1H), 4.7–4.5 (m, 3H), 4.5–4.3 (m, 2H), 4.0–3.8 (m, 1H), 3.7 (d, *J* = 2.7 Hz, 6H), 2.5–2.4 (m, 3H); ^13^C{^1^H} NMR (126 MHz, CDCl_3_) δ 210.0, 159.6, 152.2, 143.8, 142.8, 142.6, 135.6, 129.7, 129.6, 129.3, 128.1, 114.9, 114.5, 114.0, 113.0, 85.8, 77.4, 76.0, 55.8, 55.4, 51.5, 45.6, 21.6; HRMS (MM: ESI-APCI+) *m*/*z* calcd for C_26_H_29_N_2_O_4_S [M + H]^+^ 465.1843, found 465.1840.

##### *N*-(4-Bromo-2-(((4-methoxyphenyl)amino)methyl)phenyl)-*N*-(buta-2,3-dien-1-yl)-4-methylbenzenesulfonamide (**3j**):

65% yield (1.28 g, 2.5 mmol), amorphous white solid; ^1^H NMR δ 7.7 (d, *J* = 2.4 Hz, 1H), 7.6 (d, *J* = 8.3 Hz, 2H), 7.4–7.2 (m, 3H), 6.75 (d, *J* = 8.9, 2H), 6.6 (d, *J* = 8.9, 2H), 6.4 (d, *J* = 8.4 Hz, 1H), 5.1 (q, *J* = 7.0 Hz, 1H), 4.7–4.5 (m, 3H), 4.4 (d, *J* = 15.6 Hz, 2H), 3.8 (dd, *J* = 9.3, 4.4 Hz, 1H), 3.7 (s, 3H), 2.5 (s, 3H); ^13^C{^1^H} NMR (75 MHz, CDCl_3_) δ 210.2, 152.4, 144.2, 144.0, 132.3, 130.6, 129.8, 129.6, 128.2, 115.0, 114.5, 85.9, 76.4, 55.9, 51.4, 45.4, 21.7; HRMS (MM: ESI-APCI+) *m*/*z* calcd for C_25_H_26_BrN_2_O_3_S [M + H]^+^ 513.0842, found 513.0851.

##### *N*-(Buta-2,3-dien-1-yl)-*N*-(4,5-dimethoxy-2-(((4-methoxyphenyl)amino)methyl)phenyl)-4-methylbenzenesulfonamide (**3n**):

64% yield (0.18 g, 0.36 mmol), amorphous white solid; ^1^H NMR (500 MHz, CDCl_3_) 7.6 (dt, *J* = 8.2, 2.0 Hz, 2H), 7.3–7.2 (m, 2H), 7.1–7.0 (m, 1H), 6.8 (dt, *J* = 8.8, 2.0 Hz, 2H), 6.6 (dq, *J* = 6.7, 2.1 Hz, 2H), 6.1–5.9 (m, 1H), 5.2–5.0 (m, 1H), 4.7–4.5 (m, 2H), 4.5–4.3 (m, 3H), 3.9 (dd, *J* = 13.7, 8.6 Hz, 1H), 3.8 (t, *J* = 1.9 Hz, 3H), 3.8–3.7 (m, 3H), 3.6–3.5 (m, 3H), 2.4 (d, *J* = 2.6 Hz, 3H); ^13^C{^1^H} NMR (126 MHz, CDCl_3_) δ 210.0, 152.3, 149.2, 147.7, 143.9, 142.7, 135.7, 133.8, 129.5, 129.1, 128.3, 114.9, 114.8, 111.7, 111.2, 85.8, 76.1, 55.9, 55.8, 55.7, 51.5, 45.4, 21.6; HRMS (MM: ESI-APCI+) *m*/*z* calcd for C_27_H_31_N_2_O_5_S [M + H]^+^ 495.1948, found 495.1951.

### Preparation of Allenes **3**: General Procedure

Tosylamides **S13** were synthesized according to literature.^44^

#### N-Alkylation

A round-bottomed flask equipped with a stirring magnetic bar was flamed-dried under a vacuum and backfilled with argon. Then, it was charged with K_2_CO_3_ (2 equiv), and the corresponding tosylamide **S13** was put under a vacuum and backfilled with argon three times. Then DMF (0.25 M) was added, and the mixture was stirred for 30 min at rt. Afterward, a propargyl bromide derivative (1.5 equiv) was added, and the reaction was warmed to 80 °C in an oil bath for 16 h. The reaction was quenched with a saturated solution of NH_4_Cl (aq) and extracted with EtOAc. The aqueous layer was extracted with EtOAc, and the combination of organic layers was washed with a saturated solution of NH_4_Cl (aq) (3 × 100 mL), dried over Na_2_SO_4_, and concentrated in vacuo. The residue was then purified by silica gel column chromatography with hexanes/EtOAc (7:3) as the eluent to give the propargylated products **S14.**

##### *N*-(5-Chloro-2-(hydroxymethyl)phenyl)-4-methyl-*N*-(prop-2-yn-1-yl)benzenesulfonamide (**S14g**):

60% yield (1 g, 3 mmol); ^1^H NMR (300 MHz, CDCl_3_) δ 7.67–7.53 (m, 3H), 7.31 (s, 2H), 7.11 (dd, *J* = 8.5, 2.5 Hz, 1H), 6.58 (d, *J* = 8.5 Hz, 1H), 4.89 (s, 1H), 4.60 (s, 1H), 4.36 (d, *J* = 2.5 Hz, 2H), 2.86 (d, *J* = 7.7 Hz, 1H), 2.45 (s, 3H), 2.17 (t, *J* = 2.5 Hz, 1H); ^13^C{^1^H} NMR (75 MHz, CDCl_3_) δ 144.6, 144.4, 135.6, 135.3, 134.8, 130.8, 129.8, 129.6, 128.5, 128.4, 128.3, 77.2, 74.5, 60.9, 41.9, 21.7; MS (CI), *m*/*z* (%) 332 (M^+^ – [OH], 100).

##### *N*-(4-Chloro-2-(hydroxymethyl)phenyl)-4-methyl-*N*-(prop-2-yn-1-yl)benzenesulfonamide (**S14k**):

60% yield (1 g, 2.8 mmol); ^1^H NMR (300 MHz, CDCl_3_) δ 7.66–7.50 (m, 3H), 7.34 (t, *J* = 8.8 Hz, 3H), 6.61 (t, *J* = 1.7 Hz, 1H), 4.96–4.48 (m, 2H), 4.40–4.29 (m, 2H), 2.91 (s, 1H), 2.46 (s, 3H), 2.24–2.14 (m, 1H); ^13^C{^1^H} NMR (75 MHz, DMSO-*d*_6_) δ 144.8, 141.1, 137.9, 134.5, 133.4, 131.9, 130.0, 129.9, 129.8, 129.6, 128.5, 128.3, 77.0, 74.6, 60.7, 41.9, 21.7; MS (CI), *m*/*z* (%) 332 (M^+^ – [OH], 100).

##### *N*-(3-Fluoro-2-(hydroxymethyl)phenyl)-4-methyl-*N*-(prop-2-yn-1-yl)benzenesulfonamide (**S14l**):

53% yield (600 mg, 1.8 mmol); ^1^H NMR (300 MHz, CDCl_3_) δ 7.59 (d, *J* = 8.1 Hz, 2H), 7.38–7.24 (m, 3H), 7.22–7.09 (m, 2H), 6.53–6.44 (m, 1H), 4.82 (s, 2H), 4.40 (d, *J* = 2.5 Hz, 2H), 3.07 (t, *J* = 6.9 Hz, 1H), 2.46 (s, 3H), 2.18 (t, *J* = 2.5 Hz, 1H); ^13^C{^1^H} NMR (75 MHz, CDCl_3_) δ 164.12, 160.8, 144.6, 138.9, 134.7, 129.7, 129.3 (d, *J* = 9.8 Hz), 128.5, 124.2, 124.1, 117.2, 116.9, 77.1, 74.5, 54.8, 54.8, 42.3, 21.8; ^19^F NMR (282 MHz, CDCl_3_) δ −113.1; MS (CI), *m*/*z* (%) 316 (M^+^ – [OH], 100).

##### *N*-(2-(Hydroxymethyl)-3-methylphenyl)-4-methyl-*N*-(prop-2-yn-1-yl)benzenesulfonamide (**S14m**):

53% yield (600 mg, 1.82 mmol); ^1^H NMR (300 MHz, CDCl_3_) δ 7.57 (dd, *J* = 8.3, 1.5 Hz, 2H), 7.35–7.18 (m, 3H), 7.02 (t, *J* = 7.8 Hz, 1H), 6.43 (d, *J* = 7.9 Hz, 1H), 4.98 (d, *J* = 11.6 Hz, 1H), 4.59 (t, *J* = 11.3 Hz, 1H), 4.38 (t, *J* = 1.9 Hz, 2H), 2.99 (dd, *J* = 10.8, 3.1 Hz, 1H), 2.52 (s, 3H), 2.43 (d, *J* = 3.6 Hz, 3H), 2.15 (q, *J* = 2.1 Hz, 1H); ^13^C{^1^H} NMR (75 MHz, CDCl_3_) δ 144.3, 140.7, 140.5, 137.5, 135.0, 131.6, 129.6, 128.5, 128.2, 125.6, 74.2, 57.9, 42.3, 21.7, 19.6; MS (CI), *m*/*z* (%) 312 (M^+^ – [OH], 100).

See the general procedure for homologation of alkynes to allenes.

##### *N*-(Buta-2,3-dien-1-yl)-*N*-(5-chloro-2-(hydroxymethyl)phenyl)-4-methylbenzenesulfonamide (**S15g**):

54% yield (450 mg, 1.24 mmol); ^1^H NMR (300 MHz, CDCl_3_) δ 7.55 (dd, *J* = 8.3, 1.8 Hz, 3H), 7.34 (d, *J* = 8.0 Hz, 3H), 6.43 (t, *J* = 1.6 Hz, 1H), 5.10–4.88 (m, 2H), 4.72–4.33 (m, 4H), 3.79 (d, *J* = 10.8 Hz, 1H), 2.87 (t, *J* = 6.5 Hz, 1H), 2.47 (s, 3H); ^13^C{^1^H} NMR (75 MHz, CDCl_3_) δ 210.3, 144.6, 141.3, 138.2, 134.4, 133.4, 132.1, 129.9, 129.5, 128.2, 128.0, 85.2, 76.5, 60.8, 51.4, 21.7; MS (CI), *m*/*z* (%) 346 (M^+^ – [OH], 100).

##### *N*-(Buta-2,3-dien-1-yl)-*N*-(4-chloro-2-(hydroxymethyl)phenyl)-4-methylbenzenesulfonamide (**S15k**):

72% yield (750 mg, 2 mmol); ^1^H NMR (300 MHz, CDCl_3_) δ 7.61 (d, *J* = 2.6 Hz, 1H), 7.54 (d, *J* = 8.1 Hz, 2H), 7.31 (d, *J* = 7.9 Hz, 2H), 7.10 (dd, *J* = 8.5, 2.5 Hz, 1H), 6.40 (d, *J* = 8.5 Hz, 1H), 5.09–4.82 (m, 2H), 4.70–4.36 (m, 4H), 3.87–3.75 (m, 1H), 2.86 (s, 1H), 2.46 (s, 3H); ^13^C{^1^H} NMR (75 MHz, CDCl_3_) δ 210.2, 144.6, 144.4, 135.5, 135.0, 134.7, 130.9, 129.9, 129.1, 128.4, 128.2, 85.3, 76.4, 61.0, 51.5, 21.7; MS (CI), *m*/*z* (%) 346 (M^+^ – [OH], 100).

##### *N*-(Buta-2,3-dien-1-yl)-*N*-(3-fluoro-2-(hydroxymethyl)phenyl)-4-methylbenzenesulfonamide (**S15l**):

50% yield (310 mg, 0.9 mmol); ^1^H NMR (300 MHz, CDCl_3_ δ 7.55 (d, *J* = 8.0 Hz, 2H), 7.31 (d, *J* = 8.0 Hz, 2H), 7.13 (qd, *J* = 8.3, 6.3 Hz, 2H), 6.30 (dd, *J* = 7.0, 2.2 Hz, 1H), 5.04 (q, *J* = 7.0 Hz, 1H), 4.94–4.39 (m, 5H), 3.82 (t, *J* = 10.9 Hz, 1H), 3.25–3.13 (m, 1H), 2.46 (s, 3H); ^13^C{^1^H} NMR (75 MHz, CDCl_3_) δ 210.0, 164.1, 160.8, 144.3, 134.4, 129.7, 129.0 (d, *J* = 9.9 Hz), 128.1, 123.5 (d, J = 3.4 Hz), 116.5, 116.2, 85.2, 76.3, 54.8, 54.8, 51.5, 21.6; ^19^F NMR (282 MHz, CDCl_3_) δ −113.5; MS (CI), *m*/*z* (%) 330 (M^+^ – [OH], 100).

##### *N*-(Buta-2,3-dien-1-yl)-*N*-(2-(hydroxymethyl)-3-methylphenyl)-4-methylbenzenesulfonamide (**S15m**):

70% yield (435 mg, 1.27 mmol); ^1^H NMR (300 MHz, CDCl_3_) δ 7.54 (d, *J* = 7.9 Hz, 2H), 7.37–7.26 (m, 2H), 7.19 (d, *J* = 7.5 Hz, 1H), 7.02 (t, *J* = 7.8 Hz, 1H), 6.27 (d, *J* = 8.0 Hz, 1H), 5.15–4.98 (m, 2H), 4.72–4.36 (m, 4H), 3.89–3.75 (m, 1H), 3.09 (dd, *J* = 11.0, 2.8 Hz, 1H), 2.54 (s, 3H), 2.45 (s, 3H); ^13^C{^1^H} NMR (75 MHz, CDCl_3_) δ 210.0, 144.1, 140.6, 137.6, 134.9, 131.1, 129.7, 128.3, 128.0, 125.1, 85.6, 76.2, 58.0, 51.6, 21.7, 19.6; MS (CI), *m*/*z* (%) 326 (M^+^ – [OH], 100).

See the general procedure for oxidation and reductive amination.

##### *N*-(Buta-2,3-dien-1-yl)-*N*-(5-chloro-2-(((4-methoxyphenyl)amino)methyl)phenyl)-4-methylbenzenesulfonamide (**3g**):

57% yield (330 mg, 0.71 mmol); ^1^H NMR (300 MHz, CDCl_3_) δ 7.5 (d, *J* = 7.9 Hz, 2H), 7.4 (d, *J* = 8.3 Hz, 1H), 7.2 (d, *J* = 8.0 Hz, 2H), 7.1 (dd, *J* = 8.4, 2.2 Hz, 1H), 6.7 (d, *J* = 9 Hz, 2H), 6.5–6.4 (m, 3H), 5.0 (p, *J* = 7.0 Hz, 1H), 4.6–4.2 (m, 5H), 3.8–3.7 (m, 1H), 3.6 (s, 3H), 2.4 (s, 3H); ^13^C{^1^H} NMR (75 MHz, CDCl_3_) δ 210.2, 152.3, 144.3, 142.3, 140.3, 138.5, 134.9, 132.3, 130.3, 129.8, 129.0, 128.3, 128.2, 115.0, 114.4, 85.4, 76.3, 55.9, 51.3, 45.0, 21.7; HRMS (MM: ESI-APCI+) *m*/*z* calcd for C_25_H_26_ClN_2_O_3_S [M + H]^+^ 469.1347, found 469.1351.

##### *N*-(Buta-2,3-dien-1-yl)-*N*-(4-chloro-2-(((4-methoxyphenyl)amino)methyl)phenyl)-4-methylbenzenesulfonamide (**3k**):

56% yield (530 mg, 1.13 mmol); ^1^H NMR (300 MHz, CDCl_3_) δ 7.62–7.50 (m, 3H), 7.31 (d, *J* = 8.0 Hz, 2H), 7.09 (dd, *J* = 8.5, 2.6 Hz, 1H), 6.77 (d, *J* = 8.7 Hz, 2H), 6.58 (d, *J* = 8.7 Hz, 2H), 6.51 (d, *J* = 8.5 Hz, 1H), 5.11 (p, *J* = 7.0 Hz, 1H), 4.72–4.48 (m, 3H), 4.41 (d, *J* = 15.5 Hz, 2H), 3.83 (dd, *J* = 13.9, 8.3 Hz, 1H), 3.74 (s, 3H), 2.45 (s, 3H); ^13^C{^1^H} NMR (75 MHz, CDCl_3_) δ 210.2, 152.4, 144.2, 143.8, 142.3, 135.9, 135.3, 134.9, 129.8, 129.4, 129.3, 128.2, 127.5, 115.0, 11447.5, 85.5, 76.3, 55.9, 51.4, 45.4, 21.7; HRMS (MM: ESI-APCI+) *m*/*z* calcd for C_25_H_26_ClN_2_O_3_S [M + H]^+^ 469.1347, found 469.1348.

##### *N*-(Buta-2,3-dien-1-yl)-*N*-(3-fluoro-2-(((4-methoxyphenyl)amino)methyl)phenyl)-4-methylbenzenesulfonamide (**3l**):

52% yield (210 mg, 0.47 mmol); ^1^H NMR (300 MHz, CDCl_3_) δ 7.59 (d, *J* = 8.0 Hz, 2H), 7.29 (d, *J* = 7.9 Hz, 2H), 7.21–7.04 (m, 2H), 6.81 (d, *J* = 9.0 Hz, 2H), 6.77–6.67 (m, 2H), 6.47 (d, *J* = 7.7 Hz, 1H), 5.03 (p, *J* = 6.9 Hz, 1H), 4.67–4.45 (m, 3H), 4.41–4.21 (m, 2H), 3.97–3.84 (m, 1H), 3.76 (s, 3H), 2.45 (s, 3H); ^13^C{^1^H} NMR (75 MHz, CDCl_3_) δ 210.0, 162.3 (d, *J* = 248.7 Hz), 152.6, 144.2, 142.8, 139.8 (d, *J* = 6.3 Hz), 135.2, 129.7, 128.9, 128.6 (d, *J* = 9.9 Hz), 128.4, 128.2, 124.5 (d, *J* = 3.4 Hz), 116.2 (d, *J* = 22.8 Hz), 115.2, 114.9, 85.5, 76.2, 55.9, 51.7, 39.8, 21.7; ^19^F NMR (282 MHz, CDCl_3_) δ −113.1 (t, *J* = 7.8 Hz); HRMS (MM: ESI-APCI+) *m*/*z* calcd for C_25_H_26_FN_2_O_3_S [M + H]^+^ 453.1643, found 453.1645.

##### *N*-(Buta-2,3-dien-1-yl)-*N*-(2-(((4-methoxyphenyl)amino)methyl)-3-methylphenyl)-4-methylbenzenesulfonamide (**3m**):

58% yield (330 mg, 0.74 mmol); ^1^H NMR (300 MHz, CDCl_3_) δ 7.60 (d, *J* = 8.1 Hz, 2H), 7.27 (d, *J* = 8.0 Hz, 2H), 7.20 (d, *J* = 7.6 Hz, 1H), 7.08 (t, *J* = 7.7 Hz, 1H), 6.82 (d, *J* = 8.9 Hz, 2H), 6.70 (d, *J* = 8.9 Hz, 2H), 6.48 (d, *J* = 7.8 Hz, 1H), 5.05 (dq, *J* = 8.0, 6.6 Hz, 1H), 4.68–4.46 (m, 2H), 4.46–4.20 (m, 3H), 3.90 (ddt, *J* = 13.8, 8.2, 1.8 Hz, 1H), 3.77 (s, 3H), 2.47 (s, 3H), 2.44 (s, 3H); ^13^C{^1^H} NMR (75 MHz, CDCl_3_) δ 209.8, 152.3, 143.8, 143.4, 140.2, 138.8, 138.2, 135.6, 131.0, 129.6, 128.2, 127.6, 125.9, 114.9, 114.6, 85.6, 76.0, 55.9, 51.6, 42.6, 21.6, 19.5; HRMS (MM: ESI-APCI+) *m*/*z* calcd for C_26_H_29_N_2_O_3_S [M + H]^+^: 449.1893, found 449.1896.

#### General Procedure for the Asymmetric Cyclization of Allenes **3**

A 5 mL sealed tube equipped with a stirring magnetic bar was flamed-dried under a vacuum, cooled to rt, and backfilled with argon. Then it was charged with [Rh(cod)Cl]_2_ (3 mg, 6 μmol, 0.04 equiv), PPTS (4 mg, 15 μmol, 0.1 equiv) or ClCH_2_CO_2_H (1.4 mg, 15 μmol, 0.1 equiv), and (*R*)-DTBM-Garphos (19 mg, 15 μmol, 0.1 equiv). Afterward, it was put in a vacuum and backfilled with argon for three times. Then 0.4 mL of DCE was added, and the mixture was stirred for 10 min at rt. Finally, the allene **3** (0.15 mmol, 1 equiv) was added under a flow of argon, and the mixture was stirred at 50 °C in an oil bath for 24 h. After the mixture was cooled at rt and the solvent was stripped off, the resulting residue was purified by silica gel column chromatography with hexanes/EtOAc (9:1) as the eluent to give the desired seven-membered heterocycle **2**.

##### 1-Tosyl-3-vinyl-1,2,3,5-tetrahydrobenzo[*e*][1,4]oxazepine (**2a**):

used the general procedure with PPTS as Bronsted acid, 90%, 56% ee, [α]_D_^25^ −7.54 (*c* 1, CHCl_3_). SFC conditions: 30% MeOH, Phenomenex Amylose 1 at 40 °C, (CO_2_/MeOH = 70:30, 1 mL/min), λ = 210 nm, *t*_R_ (min): major = 5.98, minor = 6.96). See other spectroscopic data of **2a** in the racemic cyclization of alkyne **1a**.

##### (*R*)-4-(4-Methoxyphenyl)-1-tosyl-3-vinyl-2,3,4,5-tetrahydro-1*H*-benzo[*e*][1,4]diazepine (**2e**):

PPTS as Brønsted acid, 70% yield, amorphous off-white solid, 90% ee, [α]_D_^25^ −22.4 (*c* 0.5, CHCl_3_); ^1^H NMR (500 MHz, CDCl_3_) δ 7.78 (d, *J* = 8.1 Hz, 1H), 7.22–7.03 (m, 5H), 6.64 (d, *J* = 8 Hz, 2H), 6.51 (d, *J* = 9 Hz, 2H), 6.25 (d, *J* = 9 Hz, 2H), 5.75 (ddd, *J* = 17.3, 10.6, 3.9 Hz, 1H), 5.16 (dd, *J* = 28.1, 13.9 Hz, 2H), 4.57–4.36 (m, 3H), 4.04 (d, *J* = 17.2 Hz, 1H), 3.65 (s, 3H), 3.47 (q, *J* = 13.4, 12.7 Hz, 1H), 2.08 (s, 3H); ^13^C{^1^H} NMR (126 MHz, CDCl_3_) δ 152.1, 144.0, 143.0, 139.5, 136.2, 133.6, 129.9, 129.1, 128.5, 127.7, 126.8, 126.5, 126.4, 117.2, 114.8, 114.4, 60.3, 55.7, 54.3, 51.3, 21.4; HRMS (MM: ESI-APCI+) *m*/*z* calcd for C_25_H_27_N_2_O_3_S [M + H]^+^ 435.1737, found 435.1738. SFC conditions: 30% MeOH, Phenomenex Amylose-1 at 40 °C (CO_2_/MeOH = 70:30, 1 mL/min), λ = 210 nm, *t*_R_ (min): major = 19.26, minor = 22.02).

##### (*R*)-4-(4-Methoxyphenyl)-8-methyl-1-tosyl-3-vinyl-2,3,4,5-tetrahydro-1*H*-benzo[*e*][1,4]diazepine (**2f**):

ClCH_2_CO_2_H as a Brønsted acid, 86% yield, amorphous off-white solid, 86% ee, [α]_D_^25^ −70.17 (*c* 1, CHCl_3_); ^1^H NMR (300 MHz, CDCl_3_) δ 7.68 (s, 1H), 7.20 (d, *J* = 8.3 Hz, 2H), 7.08–6.91 (m, 2H), 6.72 (d, *J* = 8.1 Hz, 2H), 6.60 (d, *J* = 9 Hz, 2H), 6.30 (d, *J* = 9 Hz, 2H), 5.82 (ddd, *J* = 16.9, 10.9, 3.5 Hz, 1H), 5.31–5.14 (m, 2H), 4.61–4.37 (m, 3H), 4.07 (d, *J* = 17.1 Hz, 1H), 3.73 (d, *J* = 2.1 Hz, 3H), 3.61–3.43 (m, 1H), 2.34 (s, 3H), 2.16 (s, 3H); ^13^C{^1^H} NMR (75 MHz, CDCl_3_) δ 152.05, 144.0, 143.0, 139.4, 137.6, 136.2, 133.7, 130.6, 129.1, 128.3, 127.2, 126.9, 126.8, 117.1, 114.9, 114.4, 60.3, 55.7, 54.3, 51.1, 21.5, 21.3; HRMS (MM: ESI-APCI+) *m*/*z* calcd for C_26_H_29_N_2_O_3_S [M + H]^+^ 449.1893, found 449.1896. SFC conditions: 30% MeOH, Phenomenex Amylose-1 at 40 °C (CO_2_/MeOH = 70:30, 1 mL/min), λ = 210 nm, *t*_R_ (min): major = 33.15, minor = 30.60).

##### (*R*)-8-Chloro-4-(4-methoxyphenyl)-1-tosyl-3-vinyl-2,3,4,5-tetrahydro-1*H*-benzo[*e*][1,4]diazepine (**2g**):

ClCH_2_CO_2_H as a Brønsted acid, 55% yield, amorphous off-white solid, 92% ee, [α]_D_^25^ −17.20 (*c* 1, CHCl_3_); ^1^H NMR (500 MHz, CDCl_3_) δ 7.8 (d, *J* = 2.2 Hz, 1H), 7.16 (d, *J* = 7.9 Hz, 1H), 7.07 (dd, *J* = 8.2, 2.1 Hz, 1H), 7.00 (d, *J* = 8.1 Hz, 1H), 6.68 (d, *J* = 8.0 Hz, 2H), 6.58–6.48 (m, 2H), 6.23 (d, *J* = 8.5 Hz, 2H), 5.75 (ddd, *J* = 17.2, 10.5, 3.8 Hz, 1H), 5.27–5.11 (m, 2H), 4.57–4.38 (m, 3H), 4.03 (d, *J* = 17.4 Hz, 1H), 3.66 (s, 4H), 3.52–3.38 (m, 1H), 2.10 (s, 3H); ^13^C{^1^H} NMR (126 MHz, CDCl_3_) δ 152.3, 143.9, 143.5, 140.7, 135.4, 133.1, 130.1, 129.5, 129.3, 127.0, 126.4, 126.1, 117.4, 114.9, 114.5, 60.5, 55.8, 54.1, 50.9, 21.5; HRMS (MM: ESI-APCI+) *m*/*z* calcd for C_25_H_26_ClN_2_O_3_S [M + H]^+^ 469.1347, found 469.1348. SFC conditions: 30% MeOH, Phenomenex Amylose-1 at 40 °C (CO_2_/MeOH = 70:30, 1 mL/min), λ = 210 nm, *t*_R_ (min): major = 19.78, minor = 16.24).

##### (*R*)-4-(4-Methoxyphenyl)-1-tosyl-8-(trifluoromethyl)-3-vinyl-2,3,4,5-tetrahydro-1*H*-benzo[*e*][1,4]diazepine (**2h**):

ClCH_2_CO_2_H as a Brønsted acid, 99% yield, amorphous off-yellow solid, 90% ee, [α]_D_^25^ −37.40 (*c* 1, CHCl_3_); ^1^H NMR (500 MHz, CDCl_3_) δ 8.1 (s, 1H), 7.4 (d, *J* = 7.9 Hz, 1H), 7.3 (d, *J* = 8.0 Hz, 1H), 7.2 (d, *J* = 7.9 Hz, 2H), 6.7 (d, *J* = 8.0 Hz, 2H), 6.6 (d, *J* = 8.5 Hz, 2H), 6.3 (d, *J* = 8.4 Hz, 2H), 5.8 (ddd, *J* = 17.2, 10.3, 3.5 Hz, 1H), 5.3–5.2 (m, 2H), 4.6 (d, *J* = 17.5 Hz, 1H), 4.6–4.5 (m, 2H), 4.2 (d, *J* = 17.5 Hz, 1H), 3.7 (d, *J* = 1.3 Hz, 3H), 3.5 (s, 1H), 2.2 (s, 3H); ^13^C{^1^H} NMR (126 MHz, CDCl_3_) δ 152.4, 143.8, 143.6, 140.2, 137.3, 135.6, 133.3, 129.3, 127.2, 123.8 (q, *J* = 272.5 Hz), 123.0–122.9 (m), 117.4, 115.0, 114.9, 114.6, 60.5, 55.8, 54.0, 51.1, 21.5; ^19^F NMR (282 MHz, CDCl_3_) δ −62.4; HRMS (MM: ESI-APCI+) *m*/*z* calcd for C_26_H_26_F_3_N_2_O_3_S [M + H]^+^ 504.1689, found 504.1683. SFC conditions: 20% MeOH, Phenomenex Amylose-1 at 40 °C (CO_2_/MeOH = 80:20, 1 mL/min), λ = 210 nm, *t*_R_ (min): major = 15.05, minor = 12.15).

##### (*R*)-7-Methoxy-4-(4-methoxyphenyl)-1-tosyl-3-vinyl-2,3,4,5-tetrahydro-1*H*-benzo[*e*][1,4]diazepine (**2i**):

ClCH_2_CO_2_H as a Brønsted acid, 86% yield, amorphous off-yellow solid, 90% ee, [α]_D_^25^ −66.28 (*c* 1, CHCl_3_); ^1^H NMR (500 MHz, CDCl_3_) δ 7.8 (d, *J* = 8.9 Hz, 1H), 7.2 (d, *J* = 7.9 Hz, 2H), 6.8–6.7 (m, 3H), 6.7 (d, *J* = 3.0 Hz, 1H), 6.55 (d, *J* = 8.5 Hz, 2H), 6.3 (d, *J* = 8.5 Hz, 2H), 5.8 (ddd, *J* = 17.3, 10.6, 4.0 Hz, 1H), 5.3–5.1 (m, 2H), 4.6–4.4 (m, 3H), 4.0 (d, *J* = 17.1 Hz, 1H), 3.8 (s, 3H), 3.7 (s, 3H), 3.5 (s, 1H), 2.2 (s, 3H); ^13^C{^1^H} NMR (126 MHz, CDCl_3_) δ 157.9, 152.3, 144.0, 142.9, 136.4, 135.8, 133.8, 132.4, 129.1, 128.2, 126.7, 117.2, 115.4, 114.3, 113.9, 112.2, 60.3, 55.7, 55.6, 54.5, 51.8, 21.5; HRMS (MM: ESI-APCI+) *m*/*z* calcd for C_26_H_29_N_2_O_4_S [M + H]^+^ 465.1843, found 465.1858. SFC conditions: 20% MeOH, Phenomenex Amylose-1 at 40 °C (CO_2_/MeOH = 80:20, 1 mL/min), λ = 210 nm, *t*_R_ (min): major = 15.71, minor = 16.97).

##### (*R*)-7-Bromo-4-(4-methoxyphenyl)-1-tosyl-3-vinyl-2,3,4,5-tetrahydro-1*H*-benzo[*e*][1,4]diazepine (**2j**):

ClCH_2_CO_2_H as Brønsted acid, 70% yield, amorphous off-white solid, 88% ee, [α]_D_^25^ −11.69 (*c* 1, CHCl_3_); ^1^H NMR (300 MHz, CDCl_3_) ^1^H NMR (300 MHz, CDCl_3_) δ 7.8 (d, *J* = 8.7 Hz, 1H), 7.3 (dd, *J* = 8.7, 2.4 Hz, 1H), 7.3 (d, *J* = 2.4 Hz, 1H), 7.2 (d, *J* = 8.0 Hz, 2H), 6.7 (d, *J* = 8.0 Hz, 2H), 6.60 (d, *J* = 9.0 Hz, 2H), 6.25 (d, *J* = 9.0 Hz, 2H), 5.8 (ddd, *J* = 17.2, 10.5, 3.7 Hz, 1H), 5.3–5.1 (m, 2H), 4.7–4.4 (m, 3H), 4.1 (d, *J* = 17.5 Hz, 1H), 3.74 (s, 3H), 3.5 (dd, *J* = 14.8, 10.6 Hz, 1H), 2.2 (s, 3H); ^13^C{^1^H} NMR (75 MHz, CDCl_3_) δ 152.3, 143.7, 143.4, 138.8, 135.8, 133.2 131.2, 130.6, 130.0, 129.3, 128.0, 126.9, 119.6, 117.43, 114.8, 114.5, 60.2, 55.7, 54.2, 50.9, 21.5; HRMS (MM: ESI-APCI+) *m*/*z* calcd for C_25_H_26_BrN_2_O_3_S [M + H]^+^ 513.0842, found 513.0851. SFC conditions: 30% MeOH, Phenomenex Amylose-1 at 40 °C (CO_2_/MeOH = 70:30, 1 mL/min), λ = 210 nm, *t*_R_ (min): major = 24.37, minor = 21.15).

##### (*R*)-7-Chloro-4-(4-methoxyphenyl)-1-tosyl-3-vinyl-2,3,4,5-tetrahydro-1*H*-benzo[*e*][1,4]diazepine (**2k**):

ClCH_2_CO_2_H as a Brønsted acid, 72% yield, amorphous off-white solid, 94% ee, [α]_D_^25^ −17.20 (*c* 1, CHCl_3_); ^1^H NMR (500 MHz, CDCl_3_) δ 7.74 (d, *J* = 8.7 Hz, 1H), 7.18–7.00 (m, 4H), 6.65 (d, *J* = 7.9 Hz, 2H), 6.55 (d, *J* = 8.8 Hz, 2H), 6.23 (d, *J* = 8.8 Hz, 2H), 5.74 (ddd, *J* = 17.3, 10.5, 4.0 Hz, 1H), 5.25–5.07 (m, 2H), 4.55–4.36 (m, 3H), 3.98 (d, *J* = 17.4 Hz, 1H), 3.66 (s, 3H), 3.40 (d, *J* = 15.6 Hz, 1H), 2.09 (s, 3H); ^13^C{^1^H} NMR (126 MHz, CDCl_3_) δ 152.4, 143.8, 143.3, 138.3, 135.9, 135.6, 133.3, 131.7, 129.3, 128.3, 127.7, 127.6, 126.9, 117.4, 114.9, 114.5, 60.3, 55.7, 54.3, 51.1, 21.5; HRMS (MM: ESI-APCI+) *m*/*z* calcd for C_25_H_26_ClN_2_O_3_S [M + H]^+^ 469.1347, found 469.1348. SFC conditions: 30% MeOH, Phenomenex Amylose-1 at 40 °C (CO_2_/MeOH = 70:30, 1 mL/min), λ = 210 nm, *t*_R_ (min): major = 20.09, minor = 17.33).

##### (*R*)-6-Fluoro-4-(4-methoxyphenyl)-1-tosyl-3-vinyl-2,3,4,5-tetrahydro-1*H*-benzo[*e*][1,4]diazepine (**2l**):

ClCH_2_CO_2_H as a Brønsted acid, 60% yield, amorphous off-white foam, 90% ee, [α]_D_^25^ −6.50 (*c* 1, CHCl_3_); ^1^H NMR (500 MHz, CDCl_3_) δ 7.59 (d, *J* = 8.3 Hz, 1H), 7.23–7.05 (m, 3H), 6.84 (t, *J* = 8.8 Hz, 1H), 6.66 (d, *J* = 7.8 Hz, 2H), 6.55 (d, *J* = 8.4 Hz, 2H), 6.26 (d, *J* = 8.4 Hz, 2H), 5.77 (ddd, *J* = 17.3, 10.5, 3.7 Hz, 1H), 5.27–5.12 (m, 2H), 4.56 (d, *J* = 17.8 Hz, 1H), 4.46 (dd, *J* = 10.7, 5.4 Hz, 2H), 4.29 (d, *J* = 17.8 Hz, 1H), 3.66 (s, 3H), 3.55–3.41 (m, 1H), 2.09 (s, 3H); ^13^C{^1^H} NMR (126 MHz, CDCl_3_) δ 159.9 (d, *J* = 244.3 Hz), 152.1, 143.7, 143.2, 141.4 (d, *J* = 5.0 Hz), 135.8, 133.2, 129.1, 127.8 (d, *J* = 10.0 Hz), 126.8, 121.5, 121.4, 117.3, 114.6, 114.4, 112.8 (d, *J* = 23.0 Hz), 60.1, 55.6, 54.10 42.7, 21.4; ^19^F NMR (282 MHz, CDCl_3_) δ −116.32 (t, *J* = 8.3 Hz); HRMS (MM: ESI-APCI+) *m*/*z* calcd for C_25_H_26_FN_2_O_3_S [M + H]^+^ 453.1643, found 453.1643. SFC conditions: 20% MeOH, Phenomenex Amylose-1 at 40 °C (CO_2_/MeOH = 80:20, 1 mL/min), λ = 210 nm, *t*_R_ (min): major = 19.08, minor = 17.66).

##### (*R*)-4-(4-Methoxyphenyl)-6-methyl-1-tosyl-3-vinyl-2,3,4,5-tetrahydro-1*H*-benzo[*e*][1,4]diazepine (**2m**):

ClCH_2_CO_2_H as a Brønsted acid, 86% yield, brown oil, 94% ee, [α]_D_^25^ −20.65 (*c* 1, CHCl_3_); ^1^H NMR (500 MHz, CDCl_3_) δ 7.60 (d, *J* = 7.8 Hz, 1H), 7.18 (s, 2H), 7.03 (t, *J* = 7.8 Hz, 1H), 6.95 (d, *J* = 7.5 Hz, 1H), 6.70 (d, *J* = 7.9 Hz, 2H), 6.58–6.50 (m, 2H), 6.25 (d, *J* = 8.6 Hz, 2H), 5.71 (ddd, *J* = 17.2, 10.6, 4.5 Hz, 1H), 5.21–5.11 (m, 2H), 4.49–4.36 (m, 2H), 4.25 (q, *J* = 17.3 Hz, 2H), 3.66 (s, 3H), 3.36 (dd, *J* = 14.7, 10.8 Hz, 1H), 2.19 (s, 3H), 2.12 (s, 3H); ^13^C{^1^H} NMR (126 MHz, CDCl_3_) δ 152.6, 144.7, 142.9, 140.0, 136.5, 136.1, 134.1, 131.7, 129.2, 128.7, 126.9, 124.6, 119.4, 117.4, 116.3, 114.4, 60.4, 55.7, 54.6, 48.5, 21.5, 20.3; HRMS (MM: ESI-APCI+) *m*/*z* calcd for C_26_H_29_N_2_O_3_S [M + H]^+^ 449.1893, found 449.1896. SFC conditions: 20% MeOH, Phenomenex Amylose-1 at 40 °C (CO_2_/MeOH = 80:20, 1 mL/min), λ = 210 nm, *t*_R_ (min): major = 20.98, minor = 19.36).

##### (*R*)-7,8-Dimethoxy-4-(4-methoxyphenyl)-1-tosyl-3-vinyl-2,3,4,5-tetrahydro-1*H*-benzo[*e*][1,4]diazepine (**2n**):

ClCH_2_CO_2_H as a Brønsted acid, 78% yield, amorphous off-white foam, 96% ee, [α]_D_^25^ −76.38 (*c* 1, CHCl_3_); ^1^H NMR (500 MHz, CDCl_3_) δ 7.5–7.4 (m, 1H), 7.2 (d, *J* = 7.9 Hz, 2H), 6.7 (d, *J* = 7.9 Hz, 2H), 6.6–6.5 (m, 3H), 6.3 (d, *J* = 8.4 Hz, 2H), 5.8 (ddd, *J* = 17.1, 10.5, 4.1 Hz, 1H), 5.3–5.1 (m, 2H), 4.5 (d, *J* = 17.0 Hz, 2H), 4.4 (dd, *J* = 10.6, 4.8 Hz, 1H), 4.0 (d, *J* = 17.1 Hz, 1H), 3.9 (d, *J* = 2.6 Hz, 6H), 3.7 (s, 3H), 3.5 (d, *J* = 14.6 Hz, 1H), 2.2 (s, 3H); ^13^C{^1^H} NMR (126 MHz, CDCl_3_) δ 152.3, 147.7, 147.2, 144.0, 143.0, 136.2, 133.8, 132.3, 129.2, 126.8, 126.0, 122.2, 117.2, 115.3, 114.3, 110.7, 60.5, 56.2, 56.1, 55.7, 54.6, 51.4, 21.5; HRMS (MM: ESI-APCI+) *m*/*z* calcd for C_27_H_31_N_2_O_5_S [M + H]^+^ 495.1948, found 495.1958. SFC conditions: 20% MeOH, Phenomenex Amylose-1 at 40 °C (CO_2_/MeOH = 80:20, 2 mL/min), λ = 210 nm, *t*_R_ (min): major = 11.50, minor = 12.90).

#### Derivatization of (*R*)-4-(4-Methoxyphenyl)-1-tosyl-3-vinyl-2,3,4,5-tetrahydro-1*H*-benzo[*e*][1,4]diazepine (**2e**)

##### (*R*)-1-Tosyl-3-vinyl-2,3,4,5-tetrahydro-1*H*-benzo[*e*][1,4]diazepin-4-ium chloride (**4**).^31^

A solution of PMP-protected amine **2e** (110 mg, 0.25 mmol) in 8.0 mL of MeCN was cooled in an ice bath and treated with a solution of CAN (275 mg, 0.5 mmol, 2.5 equiv) in water (8 mL) dropwise. The reaction allowed to warm to 25 °C and stirred for 3 h. The crude reaction was diluted with water and washed with Et_2_O, and the organic layer was discarded. The aqueous layer was basified to pH 10 with a saturated Na_2_CO_3_ solution and extracted with Et_2_O. The combined organic layers were dried with MgSO_4_ and treated with a 1 M HCl solution in Et_2_O and concentrated to afford the hydrochloride salt of the product as an amorphous white solid: 77 mg, 85% yield, [α]_D_^25^ 122.7 (*c* 0.70, MeOH); ^1^H NMR (300 MHz, methanol-*d*_4_) δ 7.77 (d, *J* = 7.8 Hz, 2H), 7.56 (dd, *J* = 5.8, 3.2 Hz, 1H), 7.45 (dd, *J* = 8.5, 4.5 Hz, 4H), 7.31–7.17 (m, 1H), 5.86 (ddd, *J* = 17.4, 10.5, 7.0 Hz, 1H), 5.73–5.54 (m, 2H), 4.51 (dd, *J* = 15.7, 2.6 Hz, 1H), 4.31 (d, *J* = 14.1 Hz, 1H), 4.25–4.15 (m, 1H), 4.09 (d, *J* = 14.0 Hz, 1H), 3.44–3.29 (m, 2H), 2.48 (s, 3H); ^13^C{^1^H} NMR (75 MHz, methanol-*d*_4_) δ 148.8, 144.4, 141.2, 135.5, 135.3, 134.6, 133.9, 133.7, 132.8, 132.2, 131.1, 126.3, 66.2, 54.8, 52.8, 24.1; HRMS (MM: ESI-APCI+) *m*/*z* calcd for C_18_H_21_ClN_2_O_2_S [M – Cl + H]^+^ 329.1324, found 329.1320.

##### (*R*)-4-(4-Methoxyphenyl)-3-vinyl-2,3,4,5-tetrahydro-1*H*-benzo[*e*][1,4]diazepine (**5**).^32^

To a solution of naphthalene (4 mg, 0.02 mmol, 0.2 equiv) in anhydrous THF (1 mL) in an oven-dried Schlenk flask under a stream of argon were added hexane-rinsed sheets of sodium metal (21.2 mg, 0.885 mmol, 6 equiv). The mixture was then sonicated at rt until a green color persisted when a solution of **2e** (62 mg, 0.143 mmol) in THF (4 mL) was added, resulting in a rapid loss of the green color. The turbid yellow reaction mixture was removed from the sonicator and stirred at rt for 15 h. Afterward, the reaction was cooled to 0 °C, and 10 mL of MeOH was slowly added to quench the Na followed by the addition of a saturated solution of NaHCO_3_ (only after consumption). The reaction was diluted with Et_2_O and washed with NaHCO_3_ and H_2_O. The organic layer was dried over Na_2_SO_4_ and concentrated in vacuo. The product was purified by silica gel column chromatography with hexanes/EtOAc (8:2) as the eluent to give the desired product **5**: 85% yield (34 mg), colorless oil, [α]_D_^25^ −50.1 (*c* 1, CHCl_3_); ^1^H NMR (300 MHz, CDCl_3_) δ 7.16 (d, *J* = 7.4 Hz, 1H), 7.01 (t, *J* = 7.6 Hz, 1H), 6.88–6.55 (m, 5H), 6.50 (d, *J* = 7.9 Hz, 1H), 5.90 (ddd, *J* = 17.0, 10.3, 3.9 Hz, 1H), 5.29 (dd, *J* = 25.6, 13.9 Hz, 2H), 5.01 (d, *J* = 17.1 Hz, 1H), 4.50–4.37 (m, 1H), 4.19 (d, *J* = 17.1 Hz, 1H), 3.9 (bs, 1H), 3.70 (s, 3H), 3.67–3.58 (m, 1H), 3.40 (dd, *J* = 14.3, 5.4 Hz, 1H); ^13^C{^1^H} NMR (75 MHz, CDCl_3_) δ 151.3, 147.8, 145.0, 135.4, 129.4, 127.4, 124.8, 119.0, 116.6, 115.5, 114.7, 113.1, 64.8, 55.8, 50.2, 48.5; HRMS (MM: ESI-APCI+) *m*/*z* calcd for C_18_H_21_ClN_2_O_2_S [M + H]^+^ 281.1648, found 281.1647.

##### (*R*)-1-(1-Tosyl-3-vinyl-1,2,3,5-tetrahydro-4*H*-benzo[*e*][1,4] diazepin-4-yl) prop-2-en-1-one (**6**)

To a suspension of **4** (45 mg, 0.123 mmol) and DMAP (1.5 mg, 0.012 mmol, 0.1 equiv) in DCM (0.1 M) cooled in an ice-bath was added Et_3_N (60 μL, 0.5 mmol, 4 equiv). After the mixture was stirred for 5 min, acryloyl chloride (20 μL, 0.247 mmol, 2 equiv) was added dropwise. The reaction was allowed to warm to rt and stirred for 2 h. The crude reaction was diluted with H_2_O and extracted with DCM. The organic layer was washed with an aqueous solution of 5% HCl, brine, dried over Na_2_SO_4_, and concentrated in vacuo. The resulting product was purified by silica gel column chromatography with hexanes/EtOAc (7:3) as the eluent to give the desired product **6**: 65% yield (30 mg), amorphous off-white solid, [α]_D_^25^ – 8.90 (*c* 0.7, CHCl_3_); ^1^H NMR (300 MHz, DMSO-*d*_6_, 80 °C) δ 7.62 (d, *J* = 7.9 Hz, 2H), 7.40–7.09 (m, 6H), 6.54 (dd, *J* = 16.7, 10.5 Hz, 1H), 5.99 (ddd, *J* = 16.4, 13.8, 3.5 Hz, 2H), 5.63 (dd, *J* = 10.4, 2.3 Hz, 1H), 5.24 (dd, *J* = 22.5, 14.0 Hz, 2H), 4.60 (d, *J* = 132.9 Hz, 3H), 4.11–4.00 (m, 2H), 2.35 (s, 3H); ^13^C{^1^H} NMR (75 MHz, DMSO-*d*_6_, 80 °C) δ 165.4, 143.3, 139.3, 137.1, 133.7, 132.1, 129.5, 129.0, 128.0, 127.2, 127.1, 126.5, 125.7, 124.1, 116.6, 52.0, 44.7, 39.8, 20.6; HRMS (MM: ESI-APCI+) *m*/*z* calcd for C_21_H_24_N_2_O_3_S [M + H]^+^ 383.1424, found 383.1422.

##### (*R*)-10-Tosyl-5,10,11,11a-tetrahydro-3*H*-benzo[*e*]pyrrolo[1,2-*a*][1,4]diazepin-3-one (**7**).^45^

A flame-dried Schlenk was charged with the Hoveyda–Grubbs second generation catalyst (2.5 mg, 0.004 mmol, 0.1 equiv), and it was put under a vacuum and backfilled with argon. Afterward, a solution of **5** (15 mg, 0.04 mmol, 1 equiv) in dry DCM (1.5 mL) was added, and the reaction was refluxed in an oil bath for 36 h. Then, the reaction crude was purified by silica gel column chromatography with a gradient of hexanes/EtOAc (60:40) to 100% EtOAc as the eluent to give the desired product **7**: 87% yield (12 mg), white foam, 88% ee, [α]_D_^25^ −8.10 (*c* 0.5, CHCl_3_); ^1^H NMR (500 MHz, CDCl_3_) δ 7.70–7.58 (m, 2H), 7.39 (dd, *J* = 7.3, 1.8 Hz, 1H), 7.32–7.21 (m, 4H), 7.16 (dd, *J* = 7.6, 1.6 Hz, 1H), 6.93 (dd, *J* = 6.0, 1.7 Hz, 1H), 6.19 (dd, *J* = 6.0, 1.7 Hz, 1H), 4.84 (d, *J* = 14.9 Hz, 1H), 4.72 (dd, *J* = 14.5, 3.4 Hz, 1H), 4.67–4.55 (m, 1H), 3.83 (d, *J* = 14.9 Hz, 1H), 2.73 (dd, *J* = 14.5, 11.3 Hz, 1H), 2.44 (s, 3H); ^13^C{^1^H} NMR (126 MHz, CDCl_3_) δ 169.1, 144.3, 142.8, 139.8, 138.3, 137.1, 130.2, 130.1, 129.6, 129.5, 129.1, 129.0, 127.3, 64.7, 53.8, 44.0, 21.7; HRMS (MM: ESI-APCI+) *m*/*z* calcd for C_19_H_19_N_2_O_3_S [M + H]^+^ 355.1111, found 355.1111. SFC conditions: 40% MeOH, Phenomenex Amylose-1 at 40 °C (CO_2_/MeOH = 60:40, 1 mL/min), λ = 210 nm, *t*_R_ (min): major = 9.12, minor = 11.06).

## Supporting Information Available

Optimization procedures, X-ray crystallographic data, chiral HPL, and NMR spectra for all new compounds (PDF)

## Accession Codes

CCDC 1983304 contains the supplementary crystallographic data for this paper. These data can be obtained free of charge via www.ccdc.cam.ac.uk/data_request/cif, or by emailing data_request@ccdc.cam.ac.uk, or by contacting The Cambridge Crystallographic Data Centre, 12 Union Road, Cambridge CB2 1EZ, UK; fax: +44 1223 336033.
