# Supplementary material for: Enantioenriched α-Vinyl 1,4-Benzodiazepines and 1,4-Benzoxazepines via Enantioselective Rhodium-Catalyzed Hydrofunctionalizations of Alkynes and Allenes
Source: J Org Chem. 2021 Jul 14;86(15):10889–902. doi: 10.1021/acs.joc.1c01268 (PMC8499028; doi:10.1021/acs.joc.1c01268)

Enantioenriched α-Vinyl 1,4-Benzodiazepines and 1,4-Benzoxazepines via Enantioselective Rhodium-Catalyzed Hydrofunctionalizations of Alkynes and Allenes

Álvaro Velasco-Rubio, Rodrigo Bernárdez, Jesús A. Varela and Carlos Saá*
Centro Singular de Investigación en Química Biolóxica e Materiais Moleculares (CiQUS), Dpto. Química Orgánica, Universidade de Santiago de Compostela 15782 Santiago de Compostela, Spain

Email: carlos.saa@usc.es

Table of Contents

Preparation of Alkynes 1a-1e and Allenes 3a-3n S2

Preparation of Alkynes 1a,1d-1e S2

Preparation of Alkyne 1b S2

Preparation of Alkyne 1c 2

Preparation of Allenes 3d-f,3h-3j, 3n S3

Preparation of Allenes 3g, 3k, 3l, 3m........ S3

Optimization of the Asymmetric Cyclization of Alkynes 1 .... S4

Optimization of cyclization of 1d S4

Optimization of cyclization of 1e S5

Optimization of the Asymmetric Cyclization of Allenes 3 .... S5

Optimization of cyclization of 3d S5

Optimization of cyclization of 3e S6

Unsuccessful allenes in the Rh-H catalyzed hydroamination S7

X-ray Crystallographic Data S8

HPLC and SFC Chromatograms of Enantioenriched Isolated Products S15

NMR spectra S27

Preparation of Alkynes 1a-1e and Allenes 3a-3n

Preparation of Alkynes 1a,1d-1e

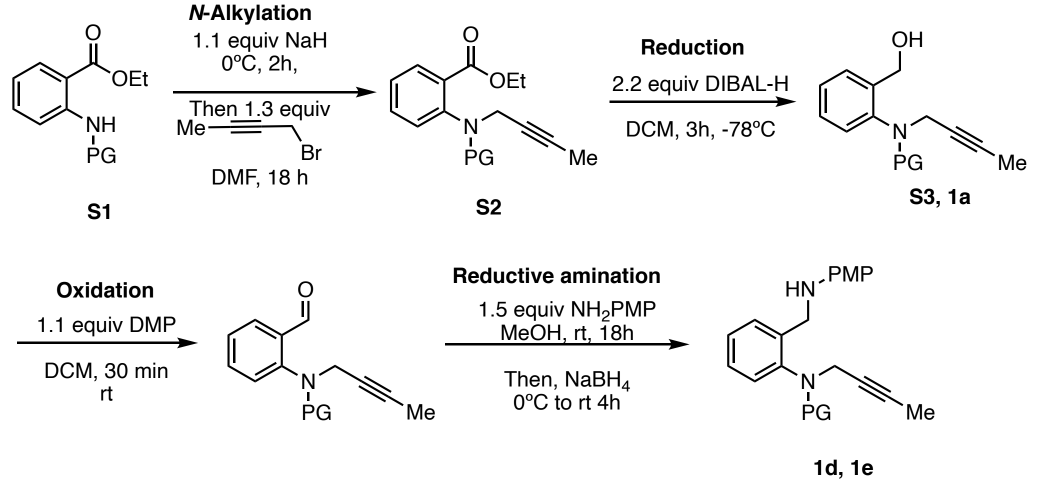

Preparation of Alkyne 1b

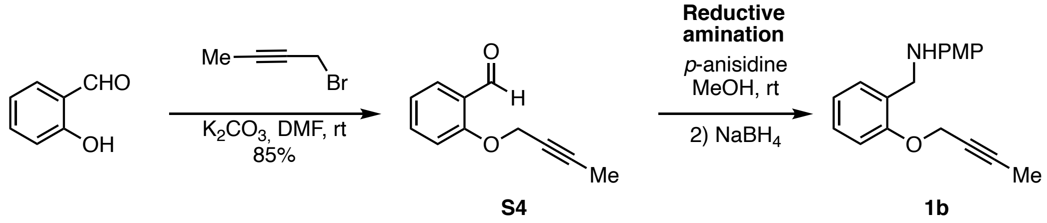

Preparation of Alkyne 1c

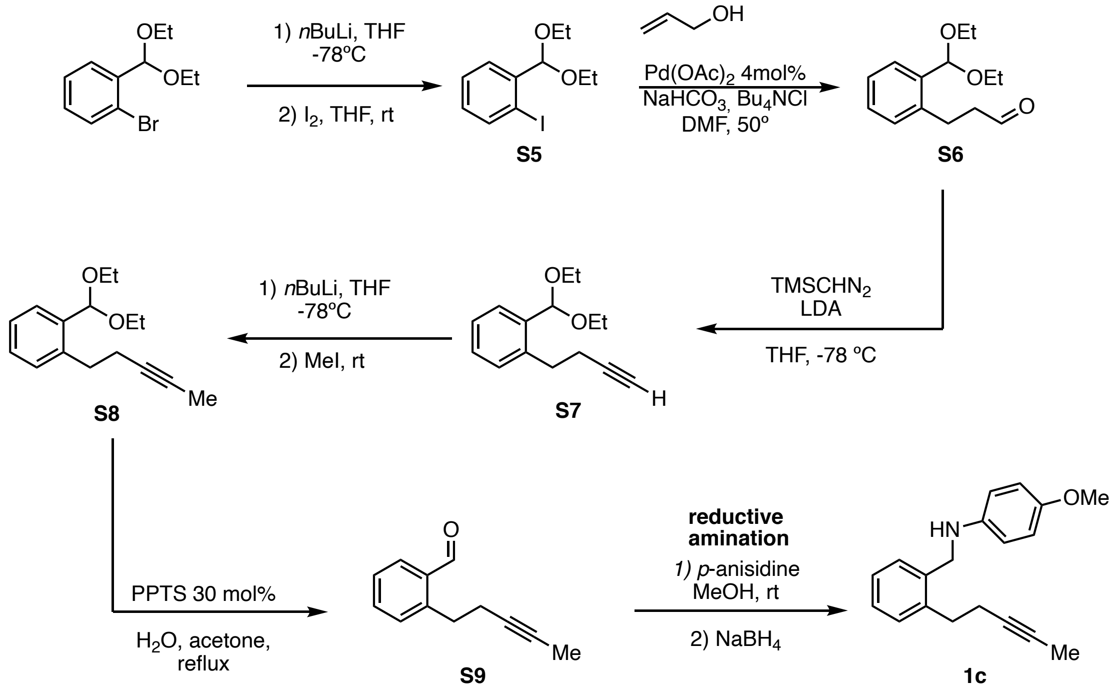

Preparation of Allenes 3d-f,3h-3j, 3n

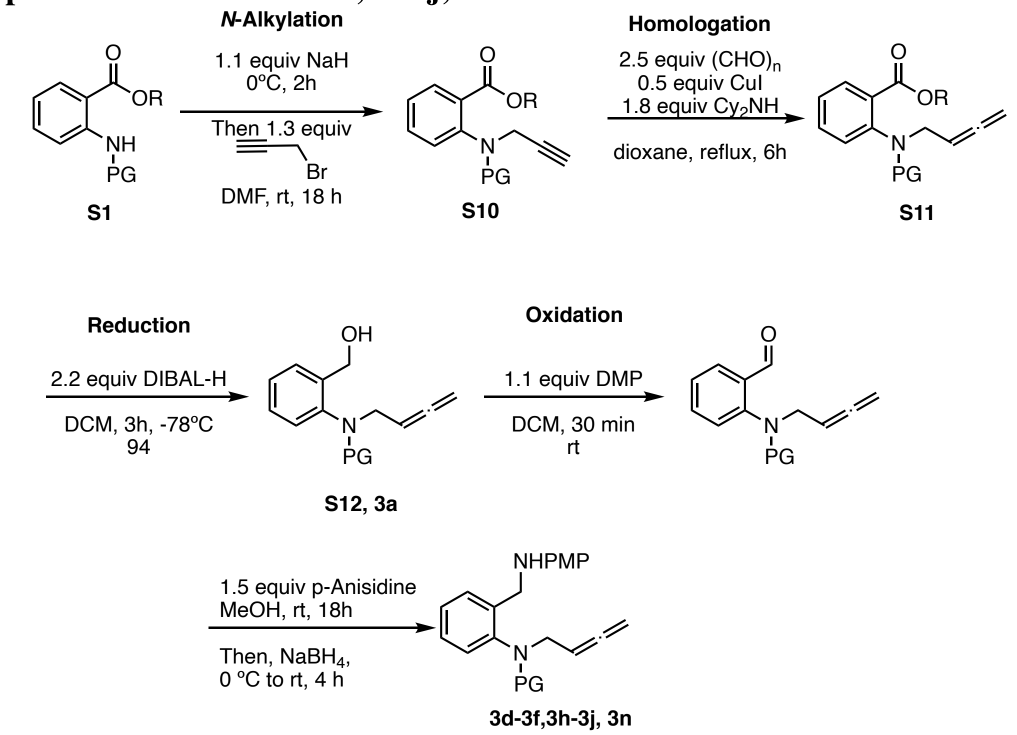

Preparation of Allenes 3g, 3k, 3l, 3m

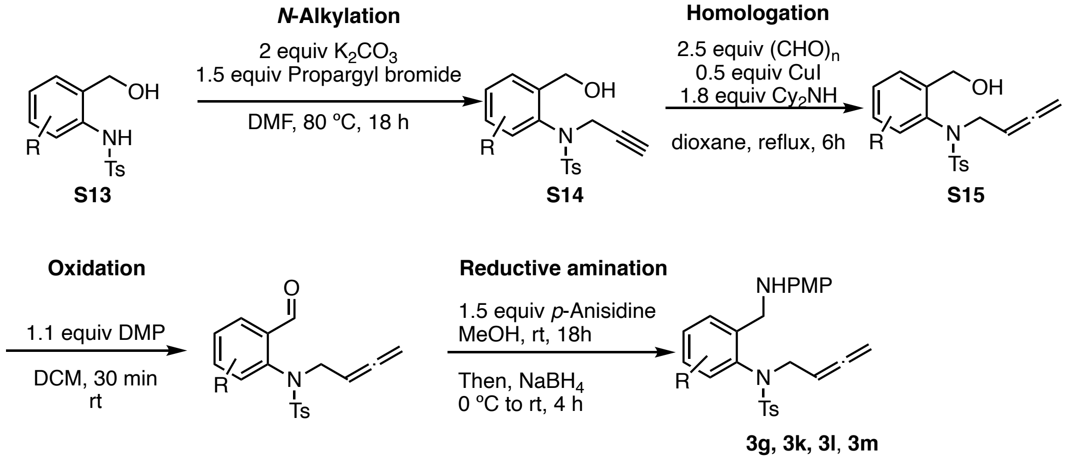

Optimization of the Asymmetric Cyclization of Alkynes 1

Optimization of cyclization of 1d

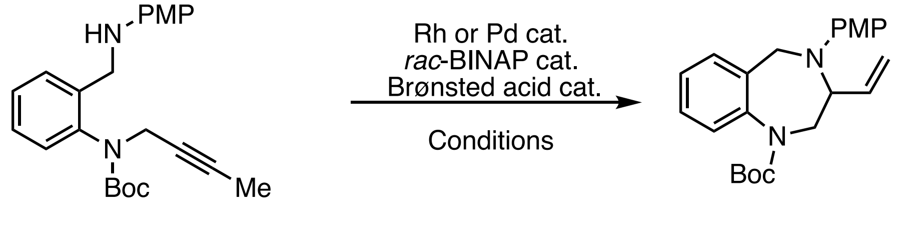

1d **2d**

Table S1. Screening of conditions

Entry[a]	Catalyst	*Rac*-BINAP	Brønsted Acid	% yield[c]
1	4 mol% $[Rh(cod)Cl]_2$	8 mol%	8 mol% *rac*-BNP acid	61
2	4 mol% $[Rh(cod)Cl]_2$	8 mol%	8 mol% $(PhO)_2PO_2H$	52
3	4 mol% $[Rh(cod)Cl]_2$	8 mol%	8 mol% $PhCO_2H$	-[d]
4[b]	15 mol% $Pd(PPh_3)_4$	-	8 mol% $PhCO_2H$	traces
5	4 mol% $[Rh(cod)Cl]_2$	8 mol%	8 mol% *R*-Trip	69 (0)[e]

a) Conditions: **1d** (0.2 mmol), 0.4 M in DCE, 70 ºC, 21 h; b) **1d** (0.2 mmol), 0.4 M in 1,4-Dioxane, 100 ºC, 21h; c) Isolated yields; d) All **1d** was recovered; e) %ee was determined by HPLC.

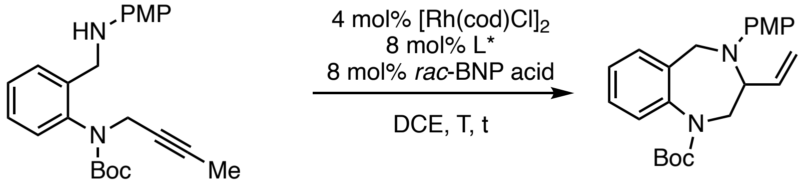

1d **2d**

Table S2. Screening of chiral ligands

Entry[a]	Ligand	T/ºC	t/h	% Yield[b]	%ee[c]
1	(*R*)-BINAP	70	20	65	14
2	(*R*)-*tol*-BINAP	70	20	77	-2
3	(*R*)-DTBM-Segphos	70	20	78	6
4	Josiphos-SL-J002-1	70	20	26	22
5	(*R*)-BINAP	50	24	19	24
6	(*R*)-BINAP	50	5d	58	24
7	(*R*)-*xyl*-BINAP	50	24	69	-2
8	(*R*)-DTBM-Segphos	50	24	73	24
9	(*S*)-DTBM-Garphos	50	24	81	-34
10	(*S*, *S*)-BDPP	50	24	0[d]	-
11	(*R*, *R*)-DIOP	50	24	>5	-
12	(*R*, *R*)-^{i}Pr-Ferrocelane	50	24	0[d]	-
13	Josiphos-SL-J002-1	50	24	>5	-
14	(*R*)-DTBM-Segphos	30	24	0[d]	-
15	(*S*)-DTBM-Garphos	30	24	0[d]	-
16	(*R*)-DTBM-Segphos	40	24	0[d]	-

a) Conditions: **1d** (0.2 mmol), 0.4 M in DCE; b) Isolated yields; c) %ee was determined by HPLC; d) SM was recovered.

Optimization of cyclization of 1e

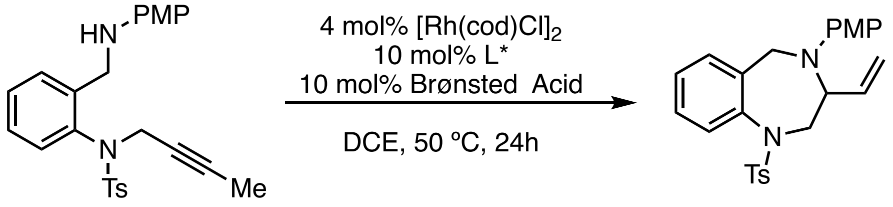

1e **2e**

Table S3. Optimization of conditions

Entry[a]	Ligand	Bronsted acid	Yield %[b]	%ee[c]
1	*rac*-BINAP	PPTS	traces	-
2[d]	*rac*-BINAP	PPTS	traces	-
3	*rac*-BINAP	*rac*-BNP acid	-	-
4[d]	*rac*-BINAP	*rac*-BNP acid	30	-
5	*rac*-BINAP	TFA	62	-
6	*rac*-BINAP	TfOH	40	
7	(*R*)-DTBM-Garphos	TFA	50	60
8[d]	(R)-DTBM-Garphos	TFA	60	20

a) Conditions: **1e** (0.15 mmol), 0.4 M in DCE; b) Isolated yields; c) %ee was determined by chiral SFC; d) 70 ºC instead of 50 ºC.

Optimization of the Asymmetric Cyclization of Allenes 3

Optimization of cyclization of 3d

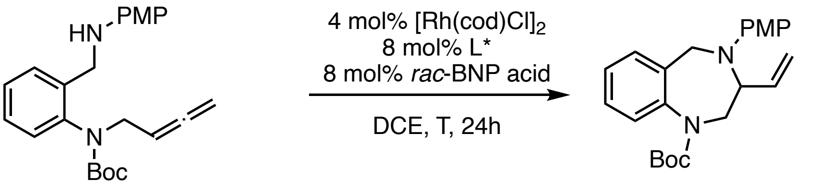

3d **2d**

Table S4. Optimization of cyclization of **3d**

Entry[a]	Ligand	T/ºC	[M]	%Yield[b]	%ee[c]
1	(*R*)-BINAP	50	0.4	36	18
2	(*R*)-*tol*-BINAP	50	0.4	51	8
3	(*R*)-*xyl*-BINAP	50	0.4	72	2
4	(*R*)- Segphos	50	0.4	47	4
5	(*R*)-DTBM-Segphos	50	0.4	50	48
6	(*R*)-DTBM-Garphos	50	0.4	76	48
7[d]	(*R*)-DTBM-Garphos	50	0.4	14	50
8	(*R*, *R*)-^{i}Pr-Ferrocelane	50	0.4	48	-6
9	Josiphos-SL-J002-1	50	0.4	14	50
10	JoSPOphos-SL-J688-2	50	0.4	45	10
11	(*R*)-DTBM-Segphos	40	0.4	9	38
12	(*R*)-DTBM-Garphos	50	0.2	70	54

a) Conditions: **3d** (0.2 mmol), 0.4 M in DCE; b) Isolated yields; c) %ee was determined by HPLC; d) (*R*)-TRIP was used instead of *rac*-BNP acid.

Optimization of cyclization of 3e

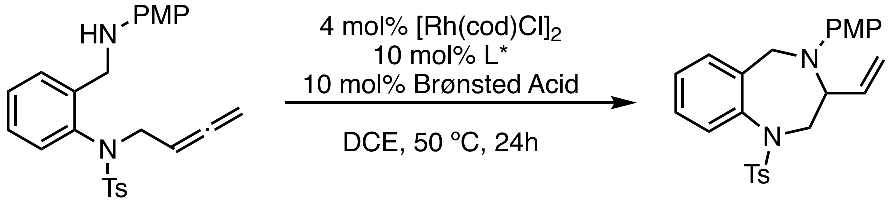

3e **2e**

Table S5. Optimization of cyclization of **3e**

Entry[a]	Ligand	Brønsted acid	[M]	%yield[b]	%ee[c]
1	(*S*)-^{i}Pr-Phox	*rac*-BNP acid	0.4	-	-
2	(*S*)-Ferrocenyl-Phox	*rac*-BNP acid	0.4	-	-
3	Davephos	*rac*-BNP acid	0.4	-	-
4	(*R*)-Phanephos	PPTS	0.4	-	-
5	(*R*)-*xyl*-P-phos	PPTS	0.4	-	-
6	(*R*)-P-phos	PPTS	0.4	-	-
7	JoSPOphos-SL-J688-2	*rac*-BNP acid	0.4	25	40
8	(*R*)-DTBM-Segphos	*rac*-BNP acid	0.2	25	72
9	(*R*)-DTBM-Segphos	PPTS	0.4	80	80
10	(*R*)-DTBM-Segphos	PPTS	0.2	70	72
11	(*R*)-Segphos	PPTS	0.2	60	56
12	(*R*)-Segphos	*rac*-BNP acid	0.2	20	56
13	(*S*)-DM-Segphos	PPTS	0.4	65	50
14	(*S*)-DM-Segphos	*rac*-BNP acid	0.4	50	16
15	(*S*)-BTFM-Garphos	PPTS	0.4	-	-
16	(*S*)-BTFM-Garphos	*rac*-BNP acid	0.4	-	-
17	(*R*)-Ph-Garphos	PPTS	0.4	-	-
18	(*R*)-*xyl*-Garphos	PPTS	0.4	15	80
19	(*R*)-*tol*-Garphos	PPTS	0.4	70	60
20	(*R*)-DTBM-Garphos	*rac*-BNP acid	0.4	25	70
21	(*R*)-DTBM-Garphos	4-Chloropyiridinium chloride	0.4	95	80
22	(*R*)-DTBM-Garphos	$ClCH_2CO_2H$	0.4	90	82
23	(*R*)-DTBM-Garphos	Acetic acid	0.4	50	90
**24**	**(*R*)-DTBM-Garphos**	**PPTS**	**0.4**	**70**	**90**
25[d]	(*R*)-DTBM-Garphos	PPTS	0.4	15	90
26[e]	(*R*)-DTBM-Garphos	PPTS	0.4	60	90

a) Conditions: **3e** (0.15 mmol), 0.4 M in DCE; b) Isolated yields; c) %ee was determined by chiral SFC; d) 40 ºC instead of 50 ºC, 1 week instead of 24 h; e) 70 ºC instead of 50 ºC.

Unsuccessful allenes in the Rh-H catalyzed hydroamination

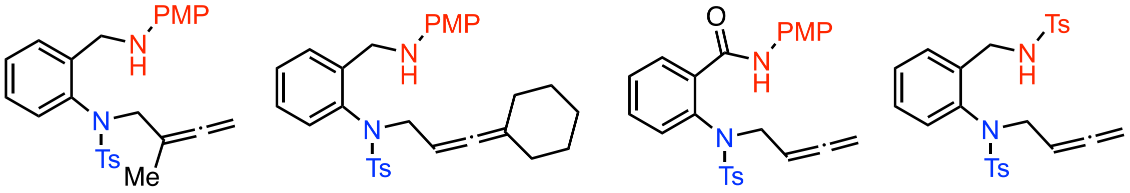

X-ray Crystallographic Data

Single crystals of compound **2e** (CCDC-1983304) suitable for X-Ray diffraction analysis were grown from solution in DCM/hexanes by slow evaporation of the solvents.

Figure S1. ORTEP drawing of **2e** showing ellipsoids at the 30% contour probability level

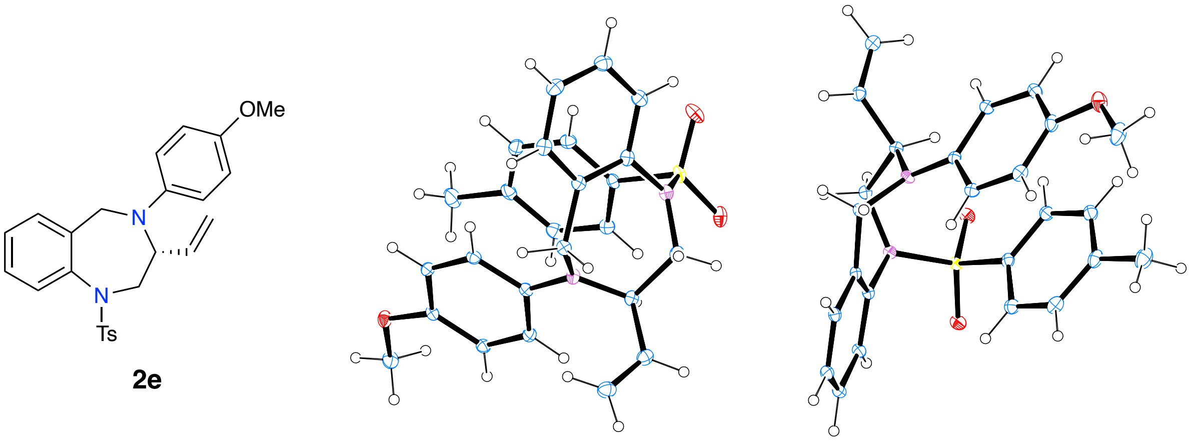

Table S6. Sample and crystal data for **2e** (19SRA011).

Identification code	19SRA011
Chemical formula	$C_{25}H_{26}N_2O_3S$
Formula weight	434.54 g/mol
Temperature	100(2) K
Wavelength	1.54178 Å
Crystal size	0.094 x 0.149 x 0.248 mm
Crystal habit	colorless prism
Crystal system	monoclinic
Space group	P 1 21 1
Unit cell dimensions	a = 12.9168(3) Å α = 90°
	b = 8.5270(2) Å β = 104.2520(10)°
	c = 20.0414(5) Å γ = 90°
Volume	2139.45(9) $Å^3$
Z	4
Density (calculated)	1.349 g/cm^3
Absorption coefficient	1.588 mm^{-1}
F(000)	920

Table S7. Data collection and structure refinement for **2e**.

Diffractometer	Bruker D8 VENTURE PHOTON-III 14 ϰ-geometry diffractometer

Radiation source	Incoatec IμS 3.0 microfocus sealed tube (Cu Kα, λ = 1.54178 Å)	
Theta range for data collection	2.27 to 80.55°	
Index ranges	-16<=h<=16, -10<=k<=10, -25<=l<=25	
Reflections collected	74898	
Independent reflections	8980 [R(int) = 0.0294]	
Coverage of independent reflections	99.3%	
Absorption correction	Multi-Scan	
Max. and min. transmission	0.8650 and 0.6940	
Structure solution technique	direct methods	
Structure solution program	SHELXT 2014/5 (Sheldrick, 2014)	
Refinement method	Full-matrix least-squares on F^2	
Refinement program	SHELXL-2018/3 (Sheldrick, 2018)	
Function minimized	$\Sigma w(F_o^2 - F_c^2)^2$	
Data / restraints / parameters	8980 / 1 / 563	
Goodness-of-fit on F^2	1.035	
Δ/σ_{max}	0.001	
Final R indices	8886 data; I>2σ(I)	R1 = 0.0233, wR2 = 0.0611
	all data	R1 = 0.0236, wR2 = 0.0614
Weighting scheme	$w=1/[\sigma^2(F_o^2)+(0.0349P)^2+0.4866P]$ where $P=(F_o^2+2F_c^2)/3$	
Absolute structure parameter	0.000(4)	
Largest diff. peak and hole	0.287 and -0.263 eÅ^{-3}	
R.M.S. deviation from mean	0.032 eÅ^{-3}	

Table S8. Atomic coordinates and equivalent isotropic atomic displacement parameters (Å^2) for **2e**

	x/a	y/b	z/c	U(eq)

N1	0.40791(11)	0.74141(18)	0.18175(8)	0.0194(3)
C2	0.41383(13)	0.7735(2)	0.25486(9)	0.0201(3)
C3	0.30531(13)	0.7896(2)	0.27294(8)	0.0186(3)
N4	0.23633(11)	0.89231(18)	0.22400(7)	0.0190(3)
C5	0.28618(13)	0.0304(2)	0.20309(9)	0.0189(3)
C6	0.33794(13)	0.0063(2)	0.14349(9)	0.0180(3)
C7	0.33306(14)	0.1300(2)	0.09710(9)	0.0210(3)
C8	0.38133(15)	0.1227(2)	0.04247(9)	0.0245(4)
C9	0.43850(14)	0.9897(3)	0.03437(9)	0.0251(4)
C10	0.44715(14)	0.8662(2)	0.08063(9)	0.0222(4)
C11	0.39611(13)	0.8716(2)	0.13446(9)	0.0189(3)
S12	0.37256(3)	0.56301(5)	0.15257(2)	0.01973(9)
O13	0.42110(11)	0.53184(16)	0.09691(8)	0.0285(3)
O14	0.39646(10)	0.46281(16)	0.21194(7)	0.0263(3)
C15	0.23317(13)	0.5628(2)	0.11784(8)	0.0175(3)
C16	0.19079(15)	0.6422(2)	0.05629(9)	0.0213(3)
C17	0.08108(15)	0.6426(2)	0.02956(9)	0.0230(4)
C18	0.01309(14)	0.5632(2)	0.06236(9)	0.0233(4)
C19	0.05779(14)	0.4835(2)	0.12359(9)	0.0235(4)
C20	0.16725(14)	0.4843(2)	0.15199(9)	0.0208(3)
C21	0.89414(15)	0.5634(3)	0.03271(11)	0.0335(5)
C22	0.32568(14)	0.8411(2)	0.34755(9)	0.0231(4)
C23	0.28015(15)	0.9635(3)	0.36927(10)	0.0280(4)
C24	0.12551(13)	0.8728(2)	0.20588(8)	0.0164(3)
C25	0.07310(14)	0.7780(2)	0.24419(8)	0.0190(3)
C26	0.96299(13)	0.7527(2)	0.22399(8)	0.0185(3)
C27	0.90236(13)	0.8250(2)	0.16523(9)	0.0175(3)
C28	0.95296(14)	0.9236(2)	0.12746(8)	0.0183(3)
C29	0.06244(14)	0.9461(2)	0.14685(8)	0.0183(3)
O30	0.79429(10)	0.80635(16)	0.13923(6)	0.0225(3)
C31	0.74240(14)	0.6954(2)	0.17326(10)	0.0246(4)
N51	0.59661(11)	0.13008(17)	0.32215(7)	0.0151(3)
C52	0.58418(13)	0.1543(2)	0.24746(8)	0.0159(3)
C53	0.68720(13)	0.2010(2)	0.22670(8)	0.0152(3)
N54	0.74395(11)	0.31732(17)	0.27521(7)	0.0160(3)
C55	0.67944(13)	0.44393(19)	0.29219(8)	0.0157(3)
C56	0.62764(12)	0.4108(2)	0.35095(8)	0.0145(3)
C57	0.61532(13)	0.5366(2)	0.39306(8)	0.0169(3)
C58	0.56469(14)	0.5192(2)	0.44617(9)	0.0192(3)
C59	0.52322(14)	0.3744(2)	0.45759(9)	0.0194(3)
C60	0.53289(13)	0.2479(2)	0.41583(8)	0.0177(3)
C61	0.58644(12)	0.2645(2)	0.36348(8)	0.0152(3)
S62	0.66754(3)	0.98020(4)	0.36046(2)	0.01504(8)
O63	0.62523(10)	0.93380(14)	0.41736(6)	0.0206(3)
O64	0.66867(9)	0.86874(14)	0.30672(6)	0.0191(2)
C65	0.80000(13)	0.0415(2)	0.39650(8)	0.0171(3)
C66	0.82287(15)	0.1329(2)	0.45575(9)	0.0207(3)
C67	0.92830(15)	0.1719(2)	0.48581(9)	0.0237(4)

C68	0.01144(15)	0.1182(2)	0.45837(9)	0.0235(4)
C69	0.98657(14)	0.0271(2)	0.39896(9)	0.0221(4)
C70	0.88112(13)	0.9888(2)	0.36726(9)	0.0192(3)
C71	0.12618(16)	0.1560(3)	0.49261(11)	0.0339(5)
C72	0.65802(13)	0.2546(2)	0.15251(9)	0.0190(3)
C73	0.67781(15)	0.1729(2)	0.10109(9)	0.0244(4)
C74	0.85496(13)	0.33307(19)	0.28714(8)	0.0152(3)
C75	0.91301(13)	0.2508(2)	0.24737(8)	0.0181(3)
C76	0.02337(14)	0.2602(2)	0.26252(9)	0.0204(3)
C77	0.07964(13)	0.3542(2)	0.31564(9)	0.0214(3)
C78	0.02406(14)	0.4370(2)	0.35535(9)	0.0207(3)
C79	0.91310(14)	0.4258(2)	0.34123(8)	0.0182(3)
O80	0.18884(10)	0.35648(18)	0.32440(7)	0.0301(3)
C81	0.24971(15)	0.4501(3)	0.37885(11)	0.0331(5)

Table S9. Bond lengths (Å) for **2e**

N1-C11	1.443(2)	N1-C2	1.474(2)
N1-S12	1.6533(15)	C2-C3	1.538(2)
C2-H2A	0.99	C2-H2B	0.99
C3-N4	1.447(2)	C3-C22	1.518(2)
C3-H3	1.0	N4-C24	1.397(2)
N4-C5	1.452(2)	C5-C6	1.519(2)
C5-H5A	0.99	C5-H5B	0.99
C6-C7	1.397(2)	C6-C11	1.408(2)
C7-C8	1.388(3)	C7-H7	0.95
C8-C9	1.384(3)	C8-H8	0.95
C9-C10	1.389(3)	C9-H9	0.95
C10-C11	1.397(2)	C10-H10	0.95
S12-O13	1.4326(14)	S12-O14	1.4354(14)
S12-C15	1.7633(16)	C15-C20	1.389(2)
C15-C16	1.396(2)	C16-C17	1.386(3)
C16-H16	0.95	C17-C18	1.395(3)
C17-H17	0.95	C18-C19	1.397(3)
C18-C21	1.505(2)	C19-C20	1.388(2)
C19-H19	0.95	C20-H20	0.95
C21-H21A	0.98	C21-H21B	0.98
C21-H21C	0.98	C22-C23	1.323(3)
C22-H22	0.95	C23-H23A	0.95
C23-H23B	0.95	C24-C25	1.399(2)
C24-C29	1.407(2)	C25-C26	1.396(2)
C25-H25	0.95	C26-C27	1.388(2)
C26-H26	0.95	C27-O30	1.374(2)
C27-C28	1.397(2)	C28-C29	1.385(2)
C28-H28	0.95	C29-H29	0.95
O30-C31	1.426(2)	C31-H31A	0.98
C31-H31B	0.98	C31-H31C	0.98
N51-C61	1.439(2)	N51-C52	1.480(2)
N51-S62	1.6481(14)	C52-C53	1.541(2)
C52-H52A	0.99	C52-H52B	0.99

C53-N54	1.454(2)	C53-C72	1.512(2)
C53-H53	1.0	N54-C74	1.401(2)
N54-C55	1.454(2)	C55-C56	1.517(2)
C55-H55A	0.99	C55-H55B	0.99
C56-C57	1.398(2)	C56-C61	1.403(2)
C57-C58	1.388(2)	C57-H57	0.95
C58-C59	1.387(3)	C58-H58	0.95
C59-C60	1.390(2)	C59-H59	0.95
C60-C61	1.399(2)	C60-H60	0.95
S62-O63	1.4360(12)	S62-O64	1.4392(12)
S62-C65	1.7645(17)	C65-C66	1.390(2)
C65-C70	1.395(2)	C66-C67	1.387(3)
C66-H66	0.95	C67-C68	1.399(3)
C67-H67	0.95	C68-C69	1.392(3)
C68-C71	1.507(3)	C69-C70	1.393(2)
C69-H69	0.95	C70-H70	0.95
C71-H71A	0.98	C71-H71B	0.98
C71-H71C	0.98	C72-C73	1.320(3)
C72-H72	0.95	C73-H73A	0.95
C73-H73B	0.95	C74-C79	1.401(2)
C74-C75	1.408(2)	C75-C76	1.385(2)
C75-H75	0.95	C76-C77	1.387(3)
C76-H76	0.95	C77-O80	1.378(2)
C77-C78	1.389(3)	C78-C79	1.394(2)
C78-H78	0.95	C79-H79	0.95
O80-C81	1.423(3)	C81-H81A	0.98
C81-H81B	0.98	C81-H81C	0.98

Table S10. Bond angles (°) for **2e**

C11-N1-C2	118.76(14)	C11-N1-S12	120.02(12)
C2-N1-S12	117.70(12)	N1-C2-C3	115.07(13)
N1-C2-H2A	108.5	C3-C2-H2A	108.5
N1-C2-H2B	108.5	C3-C2-H2B	108.5
H2A-C2-H2B	107.5	N4-C3-C22	114.92(15)
N4-C3-C2	109.82(14)	C22-C3-C2	108.12(14)
N4-C3-H3	107.9	C22-C3-H3	107.9
C2-C3-H3	107.9	C24-N4-C3	121.64(14)
C24-N4-C5	121.35(14)	C3-N4-C5	116.34(14)
N4-C5-C6	115.44(14)	N4-C5-H5A	108.4
C6-C5-H5A	108.4	N4-C5-H5B	108.4
C6-C5-H5B	108.4	H5A-C5-H5B	107.5
C7-C6-C11	117.92(16)	C7-C6-C5	117.69(16)
C11-C6-C5	124.26(16)	C8-C7-C6	122.35(17)
C8-C7-H7	118.8	C6-C7-H7	118.8
C9-C8-C7	119.06(17)	C9-C8-H8	120.5
C7-C8-H8	120.5	C8-C9-C10	120.02(17)
C8-C9-H9	120.0	C10-C9-H9	120.0
C9-C10-C11	120.94(17)	C9-C10-H10	119.5

C11-C10-H10	119.5	C10-C11-C6	119.66(16)
C10-C11-N1	119.20(16)	C6-C11-N1	121.09(15)
O13-S12-O14	119.28(8)	O13-S12-N1	108.09(8)
O14-S12-N1	105.67(8)	O13-S12-C15	107.01(8)
O14-S12-C15	108.48(8)	N1-S12-C15	107.87(8)
C20-C15-C16	120.99(16)	C20-C15-S12	119.76(13)
C16-C15-S12	119.25(13)	C17-C16-C15	118.95(17)
C17-C16-H16	120.5	C15-C16-H16	120.5
C16-C17-C18	121.26(17)	C16-C17-H17	119.4
C18-C17-H17	119.4	C17-C18-C19	118.56(16)
C17-C18-C21	120.89(18)	C19-C18-C21	120.55(18)
C20-C19-C18	121.11(17)	C20-C19-H19	119.4
C18-C19-H19	119.4	C19-C20-C15	119.12(16)
C19-C20-H20	120.4	C15-C20-H20	120.4
C18-C21-H21A	109.5	C18-C21-H21B	109.5
H21A-C21-H21B	109.5	C18-C21-H21C	109.5
H21A-C21-H21C	109.5	H21B-C21-H21C	109.5
C23-C22-C3	124.68(17)	C23-C22-H22	117.7
C3-C22-H22	117.7	C22-C23-H23A	120.0
C22-C23-H23B	120.0	H23A-C23-H23B	120.0
N4-C24-C25	121.96(15)	N4-C24-C29	120.63(15)
C25-C24-C29	117.39(15)	C26-C25-C24	121.73(15)
C26-C25-H25	119.1	C24-C25-H25	119.1
C27-C26-C25	119.95(15)	C27-C26-H26	120.0
C25-C26-H26	120.0	O30-C27-C26	125.38(15)
O30-C27-C28	115.55(14)	C26-C27-C28	119.06(15)
C29-C28-C27	120.87(15)	C29-C28-H28	119.6
C27-C28-H28	119.6	C28-C29-C24	120.95(15)
C28-C29-H29	119.5	C24-C29-H29	119.5
C27-O30-C31	116.65(13)	O30-C31-H31A	109.5
O30-C31-H31B	109.5	H31A-C31-H31B	109.5
O30-C31-H31C	109.5	H31A-C31-H31C	109.5
H31B-C31-H31C	109.5	C61-N51-C52	117.91(13)
C61-N51-S62	117.95(11)	C52-N51-S62	119.27(11)
N51-C52-C53	115.04(13)	N51-C52-H52A	108.5
C53-C52-H52A	108.5	N51-C52-H52B	108.5
C53-C52-H52B	108.5	H52A-C52-H52B	107.5
N54-C53-C72	114.46(14)	N54-C53-C52	108.79(13)
C72-C53-C52	108.72(13)	N54-C53-H53	108.2
C72-C53-H53	108.2	C52-C53-H53	108.2
C74-N54-C53	120.38(13)	C74-N54-C55	120.68(13)
C53-N54-C55	116.04(13)	N54-C55-C56	115.52(13)
N54-C55-H55A	108.4	C56-C55-H55A	108.4
N54-C55-H55B	108.4	C56-C55-H55B	108.4
H55A-C55-H55B	107.5	C57-C56-C61	118.01(14)
C57-C56-C55	117.58(15)	C61-C56-C55	124.32(14)
C58-C57-C56	121.74(16)	C58-C57-H57	119.1
C56-C57-H57	119.1	C59-C58-C57	119.74(16)

C59-C58-H58	120.1	C57-C58-H58	120.1
C58-C59-C60	119.73(15)	C58-C59-H59	120.1
C60-C59-H59	120.1	C59-C60-C61	120.47(16)
C59-C60-H60	119.8	C61-C60-H60	119.8
C60-C61-C56	120.27(15)	C60-C61-N51	118.86(14)
C56-C61-N51	120.86(14)	O63-S62-O64	119.23(7)
O63-S62-N51	107.87(7)	O64-S62-N51	105.68(7)
O63-S62-C65	106.07(8)	O64-S62-C65	108.32(8)
N51-S62-C65	109.45(8)	C66-C65-C70	121.05(16)
C66-C65-S62	119.76(13)	C70-C65-S62	119.07(13)
C67-C66-C65	119.02(17)	C67-C66-H66	120.5
C65-C66-H66	120.5	C66-C67-C68	121.15(17)
C66-C67-H67	119.4	C68-C67-H67	119.4
C69-C68-C67	118.78(17)	C69-C68-C71	120.21(18)
C67-C68-C71	121.01(18)	C68-C69-C70	120.99(16)
C68-C69-H69	119.5	C70-C69-H69	119.5
C69-C70-C65	118.99(17)	C69-C70-H70	120.5
C65-C70-H70	120.5	C68-C71-H71A	109.5
C68-C71-H71B	109.5	H71A-C71-H71B	109.5
C68-C71-H71C	109.5	H71A-C71-H71C	109.5
H71B-C71-H71C	109.5	C73-C72-C53	123.98(16)
C73-C72-H72	118.0	C53-C72-H72	118.0
C72-C73-H73A	120.0	C72-C73-H73B	120.0
H73A-C73-H73B	120.0	N54-C74-C79	121.14(15)
N54-C74-C75	121.39(15)	C79-C74-C75	117.41(15)
C76-C75-C74	120.64(16)	C76-C75-H75	119.7
C74-C75-H75	119.7	C75-C76-C77	121.24(16)
C75-C76-H76	119.4	C77-C76-H76	119.4
O80-C77-C76	115.62(16)	O80-C77-C78	125.26(16)
C76-C77-C78	119.12(15)	C77-C78-C79	119.97(16)
C77-C78-H78	120.0	C79-C78-H78	120.0
C78-C79-C74	121.60(16)	C78-C79-H79	119.2
C74-C79-H79	119.2	C77-O80-C81	117.43(15)
O80-C81-H81A	109.5	O80-C81-H81B	109.5
H81A-C81-H81B	109.5	O80-C81-H81C	109.5
H81A-C81-H81C	109.5	H81B-C81-H81C	109.5

HPLC and SFC Chromatograms of Enantioenriched Isolated Products

*1-Tosyl-3-vinyl-1,2,3,5-tetrahydrobenzo[e][1,4]oxazepine (**2a**)*

SFC conditions: 30% MeOH, *Phenomenex Amylose-1 at 40ºC (CO_2 : MeOH= 70:30, 1mL/min)*, λ=210 nm, t_R (min): major = 5.98, minor = 6.96).

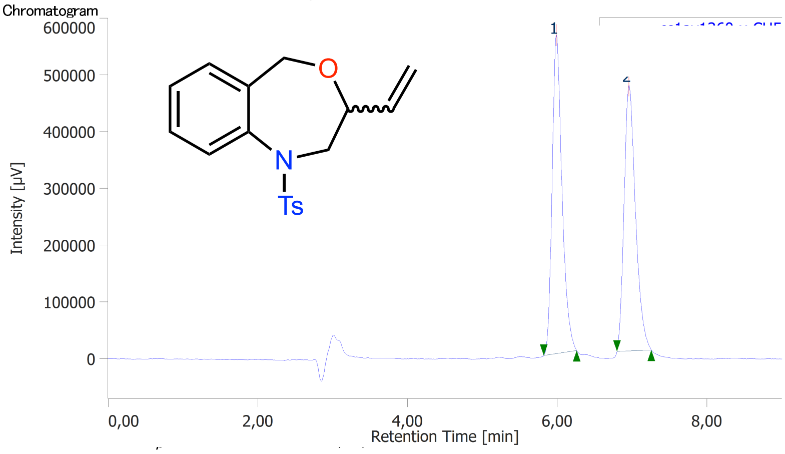

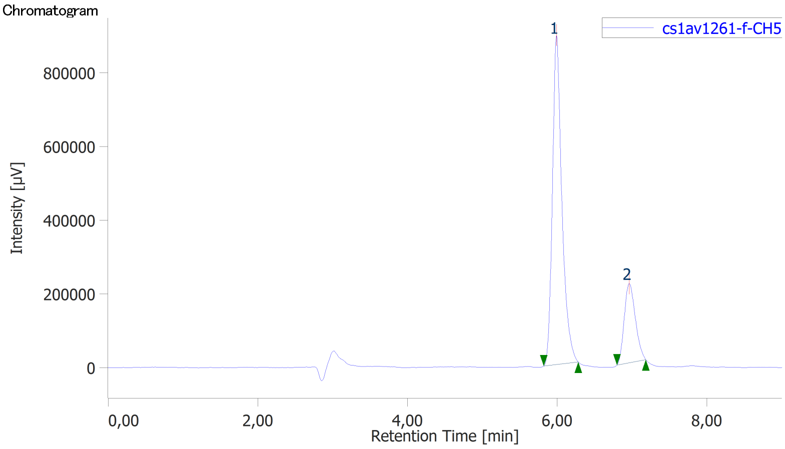

Peak Information

#	Peak Name	CH	tR [min]	Area [µV·sec]	Height [µV]	Area%	Height%	Quantity	NTP	Resolution	Symmetry Factor	Warning
1	Unknown	5	5,987	7826242	893059	77,966	80,616	N/A	12201	3,958	1,345	
2	Unknown	5	6,960	2211838	214730	22,034	19,384	N/A	10152	N/A	1,182	

Signals in the range around 2-4 min are due to the front arriving to the detector.

(R)-4-(4-Methoxyphenyl)-1-tosyl-3-vinyl-2,3,4,5-tetrahydro-1H-benzo[e][1,4]diazepine ***(2e)***

SFC conditions: 30% MeOH, *Phenomenex Amylose-1 at 40ºC* (CO_2 : *MeOH= 70:30, 1mL/min)*, λ=210 nm, t_R (min): major = 19.26, minor = 22.02).

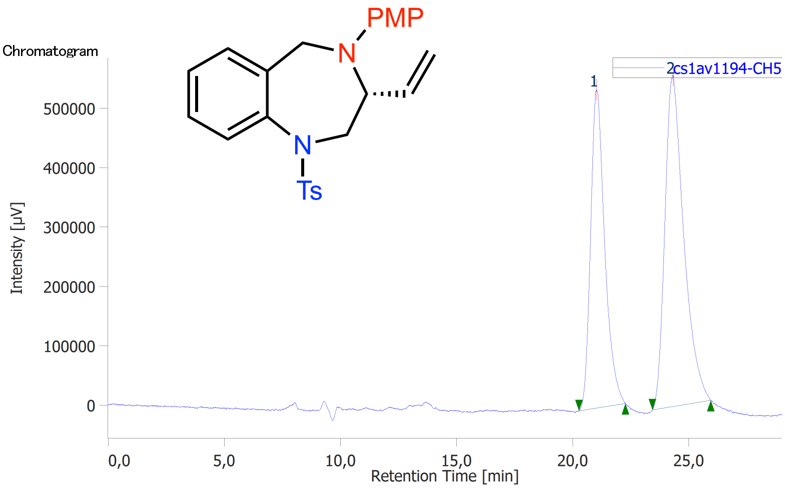

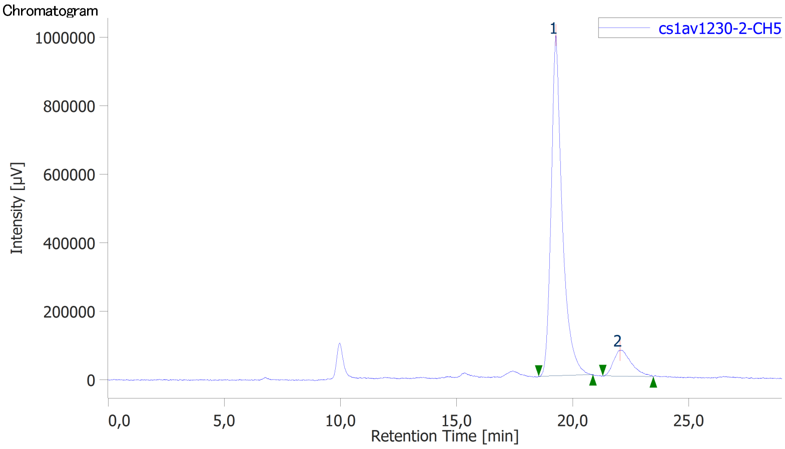

Peak Information

#	Peak Name	CH	tR [min]	Area [µV·sec]	Height [µV]	Area%	Height%	Quantity	NTP	Resolution	Symmetry Factor	Warning
1	Unknown	5	19,267	32337694	991169	94,914	95,117	N/A	9650	3,096	1,383	
2	Unknown	5	22,023	1732934	50881	5,086	4,883	N/A	7743	N/A	1,011	

Signals in the range around 10 min are due to the front arriving to the detector.

*(R)-4-(4-Methoxyphenyl)-8-methyl-1-tosyl-3-vinyl-2,3,4,5-tetrahydro-1H-benzo[e][1,4]diazepine (**2f**)*

SFC conditions: 30% MeOH, *Phenomenex Amylose-1 at 40ºC (CO_2 : MeOH= 70:30, 1mL/min)*, λ=210 nm, t_R (min): major = 33.15, minor = 30.60).

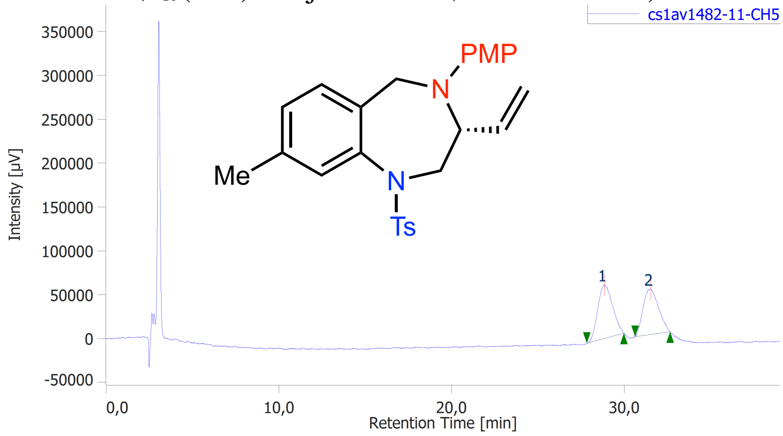

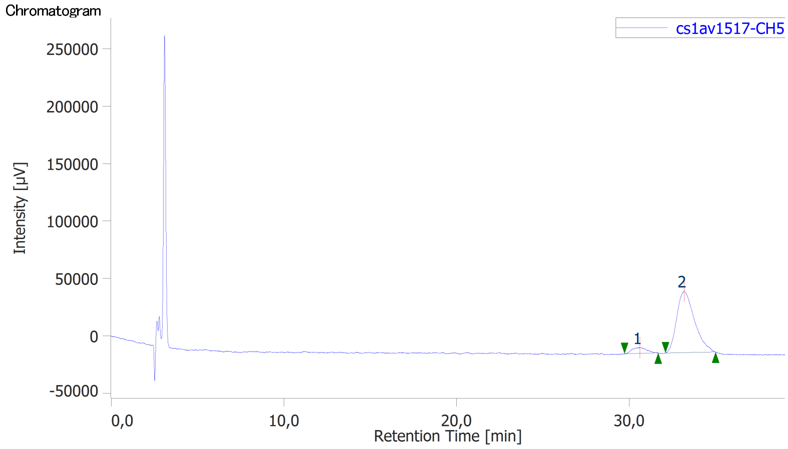

Peak Information

#	Peak Name	CH	tR [min]	Area [µV·sec]	Height [µV]	Area%	Height%	Quantity	NTP	Resolution	Symmetry Factor	Warning
1	Unknown	5	30,600	300312	5056	7,318	8,626	N/A	5657	1,475	1,100	
2	Unknown	5	33,157	3803265	53554	92,682	91,374	N/A	5150	N/A	1,365	

Signals in the range around 1-3 min are due to the front arriving to the detector.

*(R)-8-chloro-4-(4-methoxyphenyl)-1-tosyl-3-vinyl-2,3,4,5-tetrahydro-1H-benzo[e][1,4]diazepine (**2g**).*

SFC conditions: 30% MeOH, *Phenomenex Amylose-1 at 40ºC (CO_2 : MeOH= 70:30, 1mL/min)*, λ=210 nm, t_R (min): major =19.78, minor =16.24).

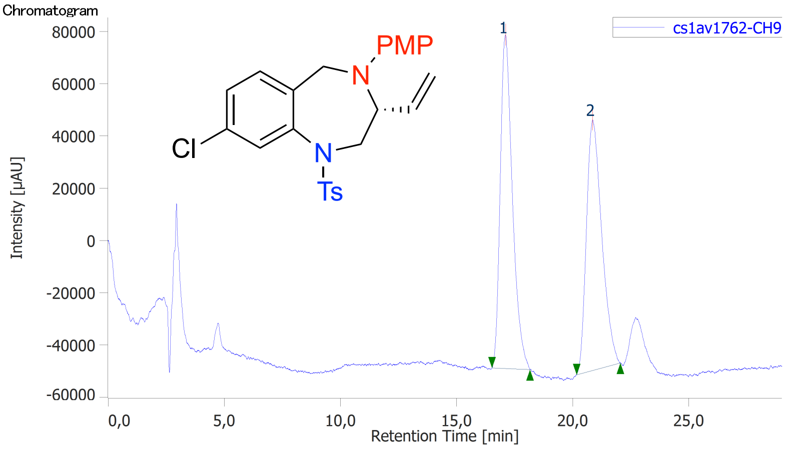

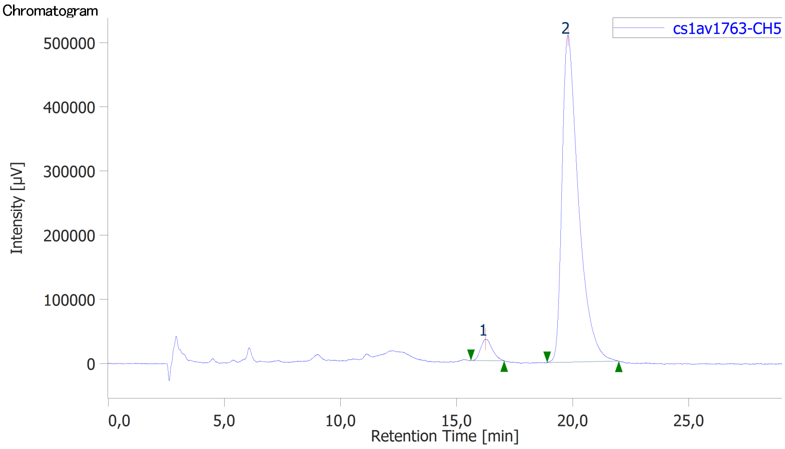

Peak Information

#	Peak Name	CH	tR [min]	Area [µV·sec]	Height [µV]	Area%	Height%	Quantity	NTP	Resolution	Symmetry Factor	Warning
1	Unknown	5	16,240	1031106	31914	4,063	5,938	N/A	5366	3,367	1,173	
2	Unknown	5	19,783	24348177	505505	95,937	94,062	N/A	4183	N/A	1,690	

Signals in the range around 2-4 min are due to the front arriving to the detector.

*(R)-4-(4-methoxyphenyl)-1-tosyl-8-(trifluoromethyl)-3-vinyl-2,3,4,5-tetrahydro-1H-benzo[e][1,4]diazepine (**2h**).*

SFC conditions: 20% MeOH, *Phenomenex Amylose-1 at 40ºC (CO_2 : MeOH= 80:20, 1mL/min)*, λ=210 nm, t_R (min): major =15.05, minor =12.15).

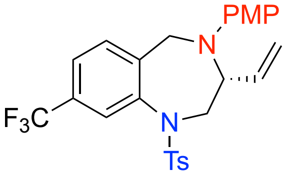

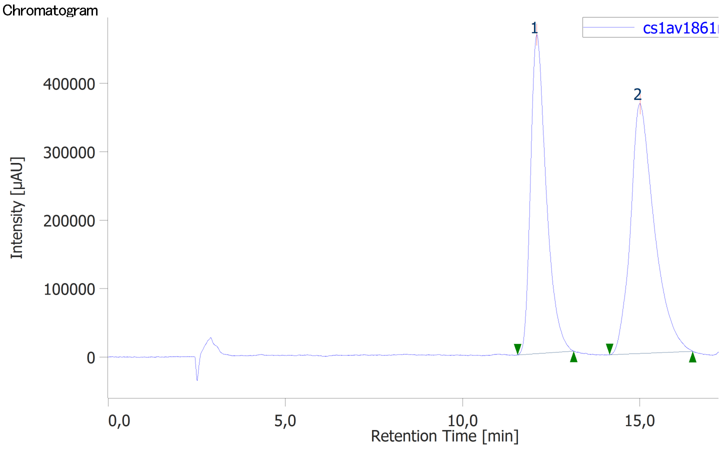

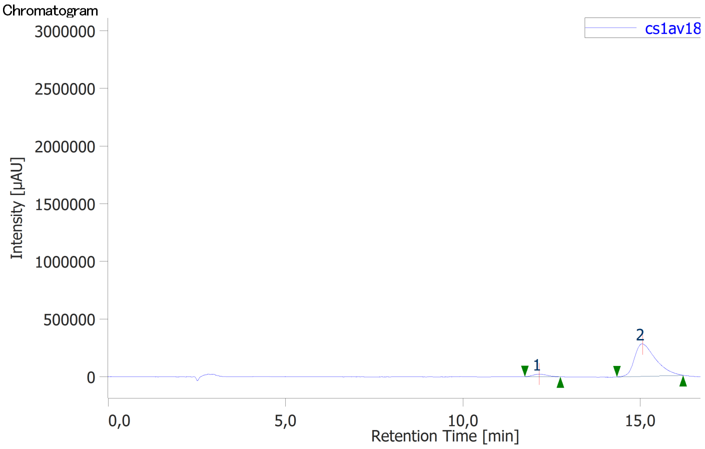

Peak Information

#	Peak Name	CH	tR [min]	Area [µV·sec]	Height [µV]	Area%	Height%	Quantity	NTP	Resolution	Symmetry Factor	Warning
1	Unknown	9	12,147	604593	22313	4,925	7,347	N/A	4284	3,216	1,190	
2	Unknown	9	15,053	11671016	281376	95,075	92,653	N/A	3164	N/A	1,508	

Signals in the range around 2-4 min are due to the front arriving to the detector.

*(R)-7-methoxy-4-(4-methoxyphenyl)-1-tosyl-3-vinyl-2,3,4,5-tetrahydro-1H-benzo[e][1,4]diazepine (**2i**).*

SFC conditions: 20% MeOH, *Phenomenex Amylose-1 at 40ºC (CO_2 : MeOH= 80:20, 1mL/min)*, λ=210 nm, t_R (min): major = 15.71), minor =16.87.

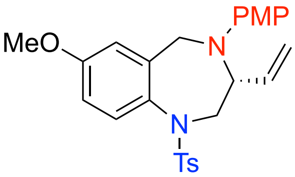

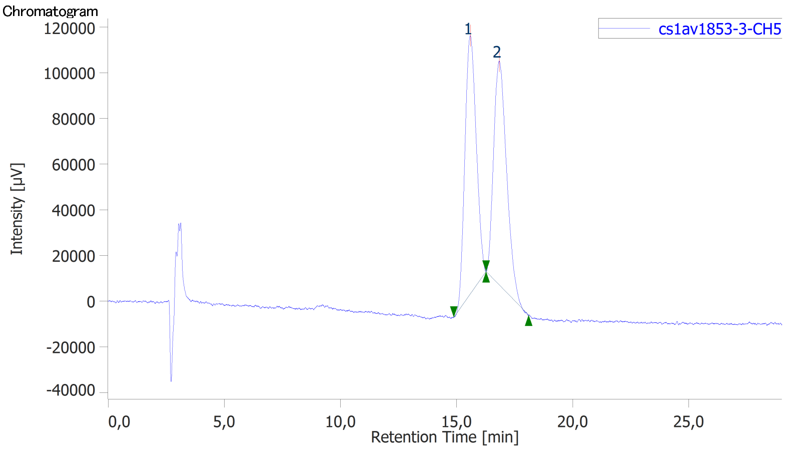

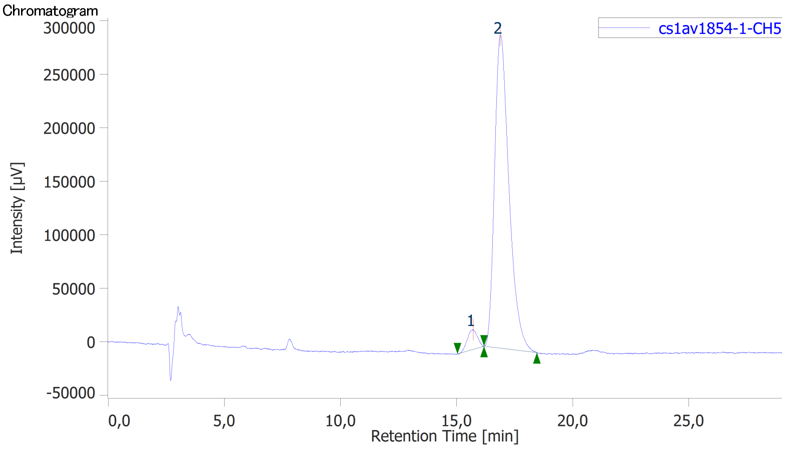

Peak Information

#	Peak Name	CH	tR [min]	Area [µV·sec]	Height [µV]	Area%	Height%	Quantity	NTP	Resolution	Symmetry Factor	Warning
1	Unknown	5	15,713	556418	18342	4,226	5,904	N/A	5386	1,185	0,892	
2	Unknown	5	16,877	12609985	292344	95,774	94,096	N/A	3683	N/A	1,426	

Signals in the range around 2-4 min are due to the front arriving to the detector.

*(R)-7-Bromo-4-(4-methoxyphenyl)-1-tosyl-3-vinyl-2,3,4,5-tetrahydro-1H-benzo[e][1,4]diazepine (**2j**)*

SFC conditions: 30% MeOH, *Phenomenex Amylose-1 at 40ºC (CO_2 : MeOH= 70:30, 1mL/min)*, λ=210 nm, t_R (min): major = 24.37, minor = 21.15).

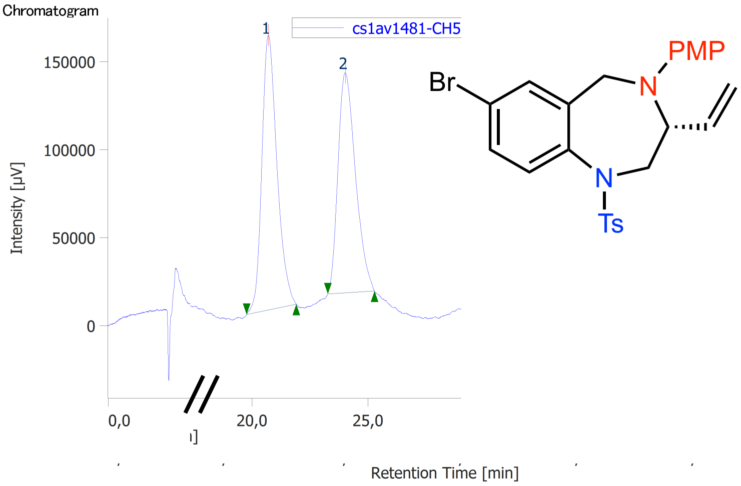

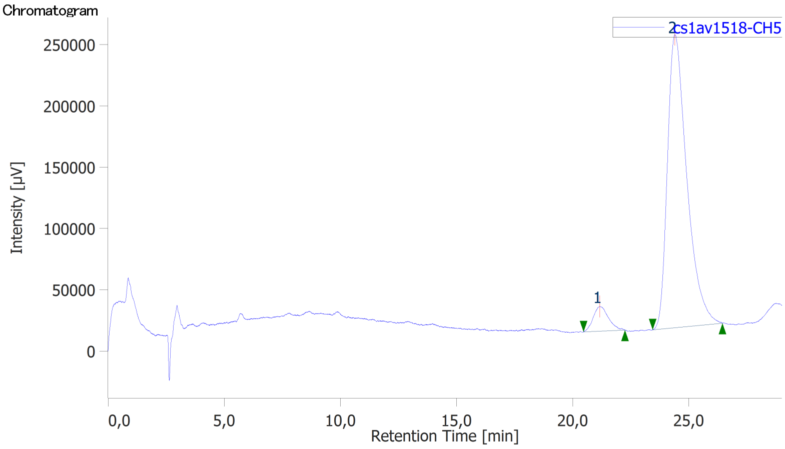

Peak Information

#	Peak Name	CH	tR [min]	Area [µV·sec]	Height [µV]	Area%	Height%	Quantity	NTP	Resolution	Symmetry Factor	Warning
1	Unknown	5	21,150	918860	20164	6,200	7,783	N/A	4968	2,416	1,286	
2	Unknown	5	24,387	13900989	238916	93,800	92,217	N/A	4310	N/A	1,532	

Signals in the range around 2-4 min are due to the front arriving to the detector.

R)-7-chloro-4-(4-methoxyphenyl)-1-tosyl-3-vinyl-2,3,4,5-tetrahydro-1H-benzo[e][1,4]diazepine (***2k***).

SFC conditions: 30% MeOH, *Phenomenex Amylose-1 at 40ºC (*CO_2 *: MeOH= 70:30, 1mL/min)*, λ=210 nm, t_R (min): major =20.09, minor =17.33).

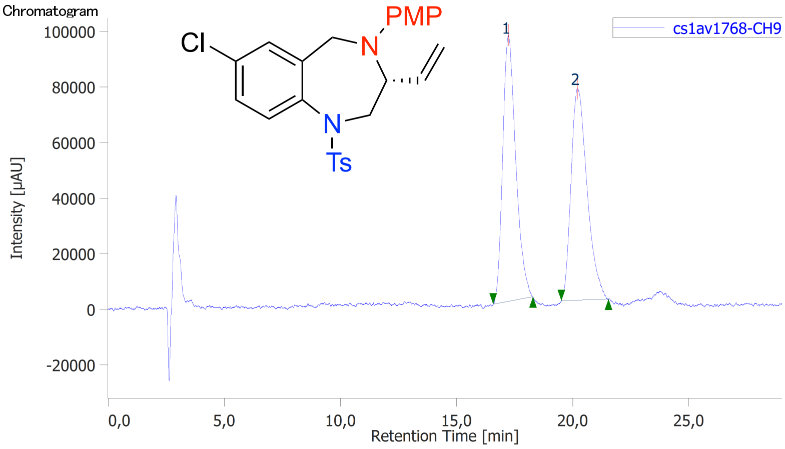

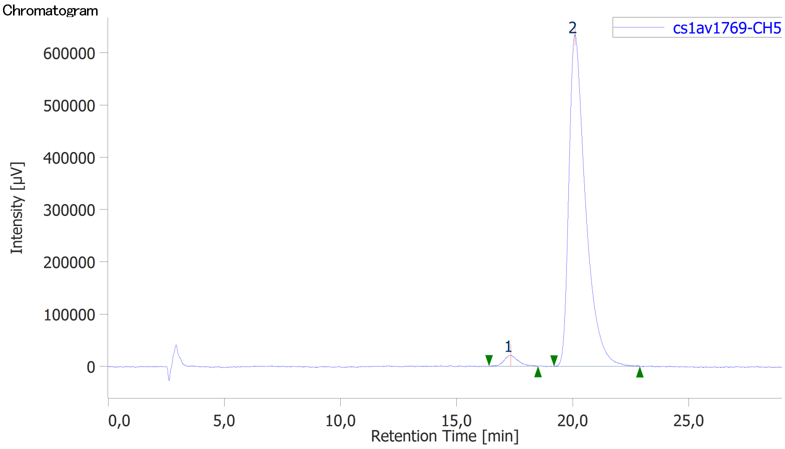

Peak Information

#	Peak Name	CH	tR [min]	Area [μV·sec]	Height [μV]	Area%	Height%	Quantity	NTP	Resolution	Symmetry Factor	Warning
1	Unknown	5	17,330	876466	20679	2,700	3,158	N/A	4235	2,405	1,205	
2	Unknown	5	20,093	31588334	634079	97,300	96,842	N/A	4206	N/A	1,678	

Signals in the range around 2-4 min are due to the front arriving to the detector.

*(R)-6-fluoro-4-(4-methoxyphenyl)-1-tosyl-3-vinyl-2,3,4,5-tetrahydro-1H-benzo[e][1,4]diazepine (**2l**).*

SFC conditions: 20% MeOH, *Phenomenex Amylose-1 at 40ºC (CO_2 : MeOH= 80:20, 1mL/min)*, λ=210 nm, t_R (min): major =19.08, minor = 17.66).

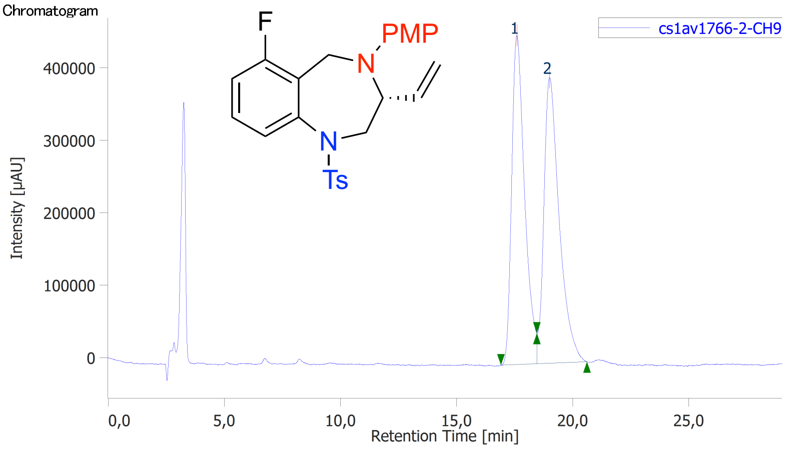

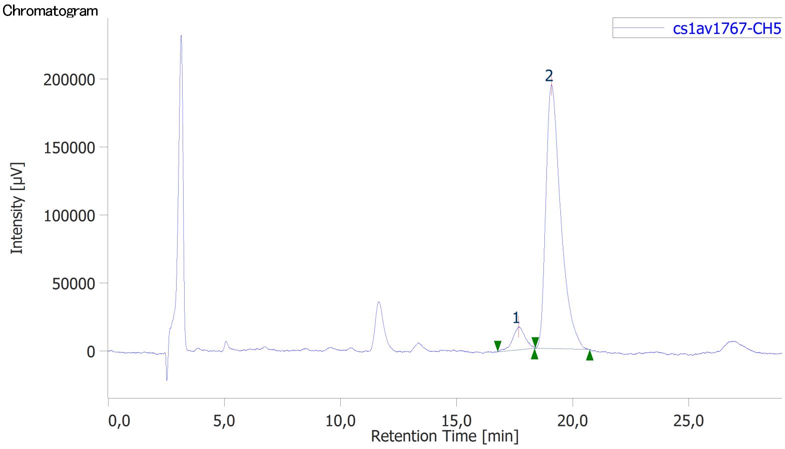

Peak Information

#	Peak Name	CH	tR [min]	Area [µV·sec]	Height [µV]	Area%	Height%	Quantity	NTP	Resolution	Symmetry Factor	Warning
1	Unknown	5	17,663	447665	14915	5,130	7,231	N/A	7215	1,456	1,101	
2	Unknown	5	19,080	8279094	191356	94,870	92,769	N/A	4646	N/A	1,546	

Signals in the range around 2-4 min are due to the front arriving to the detector.

*(R)-4-(4-methoxyphenyl)-6-methyl-1-tosyl-3-vinyl-2,3,4,5-tetrahydro-1H-benzo[e][1,4]diazepine (**2m**).*

SFC conditions: 20% MeOH, *Phenomenex Amylose-1 at 40ºC (CO_2 : MeOH= 80:20, 1mL/min)*, λ=210 nm, t_R (min): major =20.98, minor =19.36).

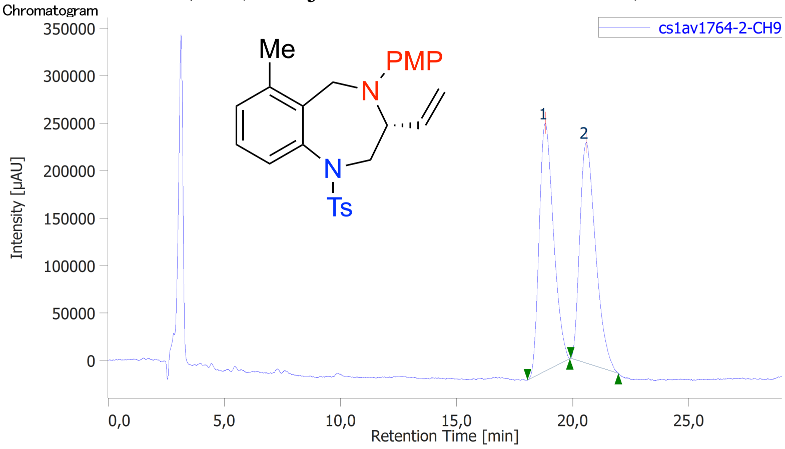

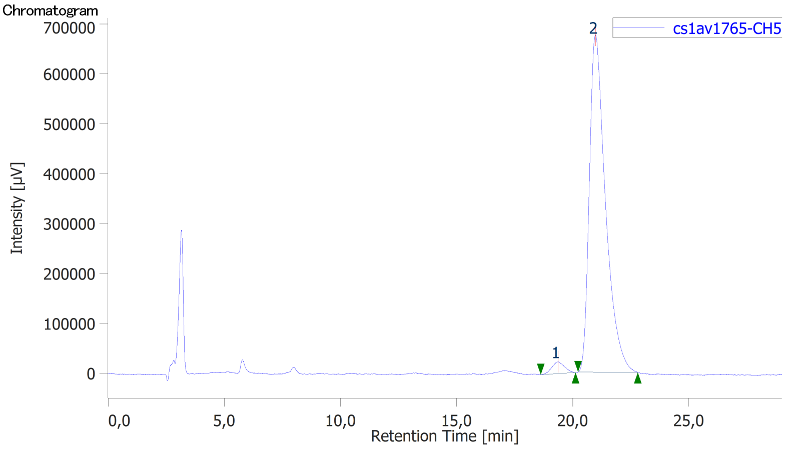

Peak Information

#	Peak Name	CH	tR [min]	Area [µV·sec]	Height [µV]	Area%	Height%	Quantity	NTP	Resolution	Symmetry Factor	Warning
1	Unknown	5	19,367	896769	23679	2,670	3,388	N/A	5687	1,439	0,987	
2	Unknown	5	20,980	32685719	675169	97,330	96,612	N/A	4722	N/A	1,565	

Signals in the range around 2-4 min are due to the front arriving to the detector.

*(R)-7,8-dimethoxy-4-(4-methoxyphenyl)-1-tosyl-3-vinyl-2,3,4,5-tetrahydro-1H-benzo[e][1,4]diazepine (**2n**).*

SFC conditions:20 % MeOH, *Phenomenex Amylose-1 at 40ºC (CO_2 : MeOH=80:20, 2mL/min)*, λ=210 nm, t_R (min): major =11.50 , minor = 12.90).

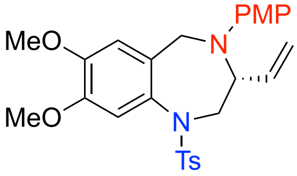

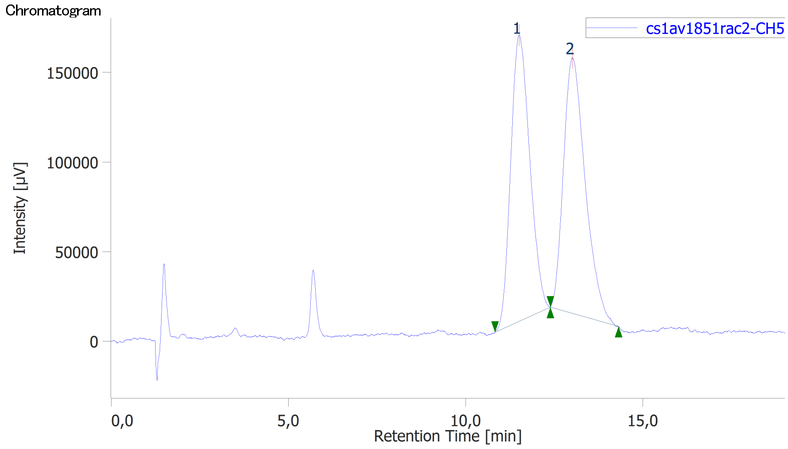

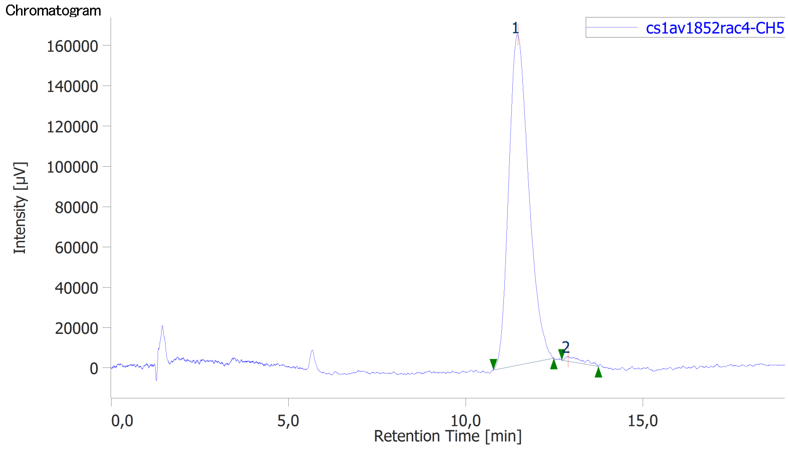

Peak Information

#	Peak Name	CH	tR [min]	Area [µV·sec]	Height [µV]	Area%	Height%	Quantity	NTP	Resolution	Symmetry Factor	Warning
1	Unknown	5	11,470	6414490	163916	98,592	98,569	N/A	1994	1,136	1,242	
2	Unknown	5	12,883	91607	2380	1,408	1,431	N/A	1232	N/A	3,552	

Signals in the range around 1-3 min are due to the front arriving to the detector.

(R)-10-Tosyl-5,10,11,11a-tetrahydro-3H-benzo[e]pyrrolo[1,2-a][1,4]diazepin-3-one (**7**)

SFC conditions: 40% MeOH, *Phenomenex Amylose-1 at 40ºC (CO_2 : MeOH= 60:40, 1mL/min)*, λ=210 nm, t_R (min): major = 9.12, minor = 11.06).

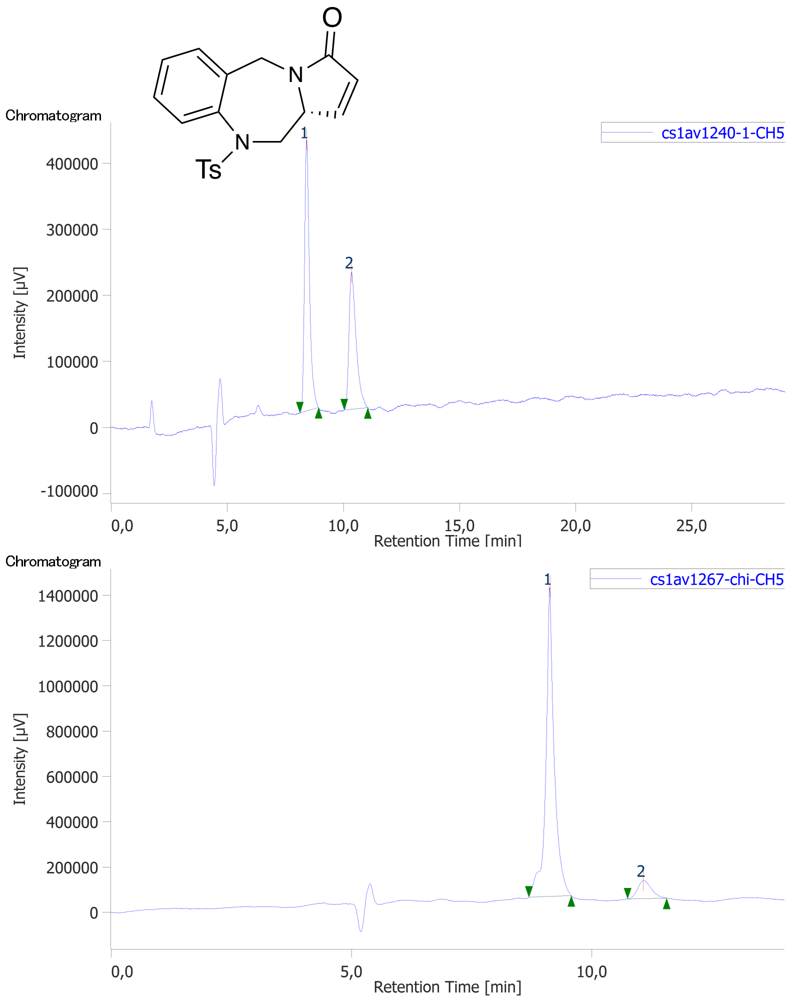

Peak Information

#	Peak Name	CH	tR [min]	Area [µV·sec]	Height [µV]	Area%	Height%	Quantity	NTP	Resolution	Symmetry Factor	Warning
1	Unknown	5	9,123	16461841	1369051	93,556	95,192	N/A	21000	5,464	0,943	
2	Unknown	5	11,063	1133955	69145	6,444	4,808	N/A	9246	N/A	1,104	

Signals in the range around 5 min are due to the front arriving to the detector.

NMR spectra

NOTE: The proton signals corresponding to NH and OH groups may not appear in the ^{1}H NMR spectra due to deuterium exchange.

^{1}H-NMR (300 MHz). Solvent $CDCl_3$

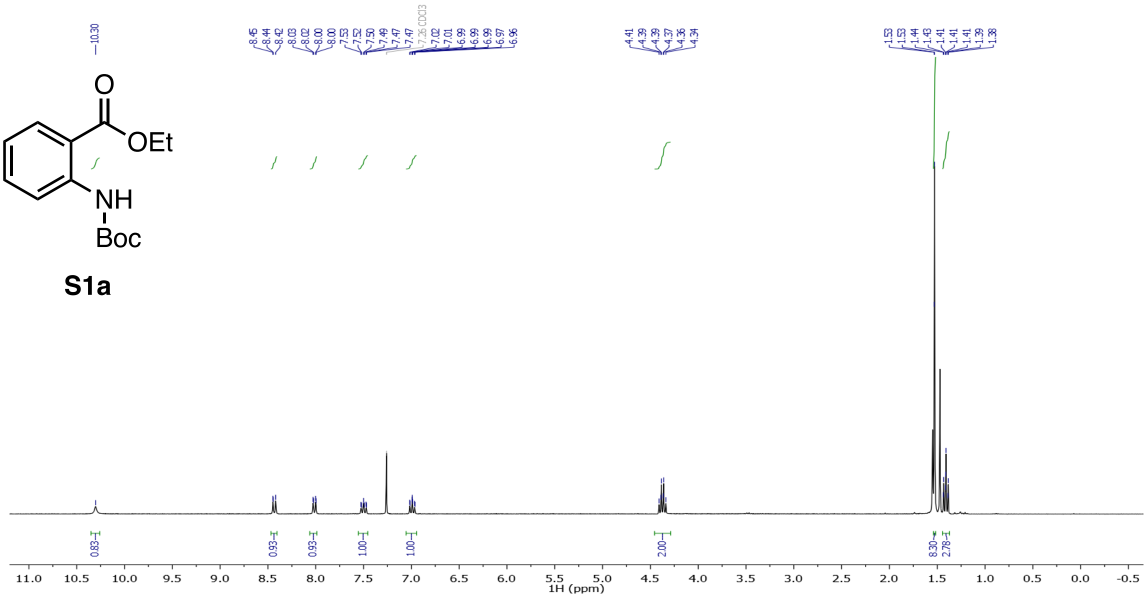

^{13}C-NMR (75 MHz). Solvent $CDCl_3$

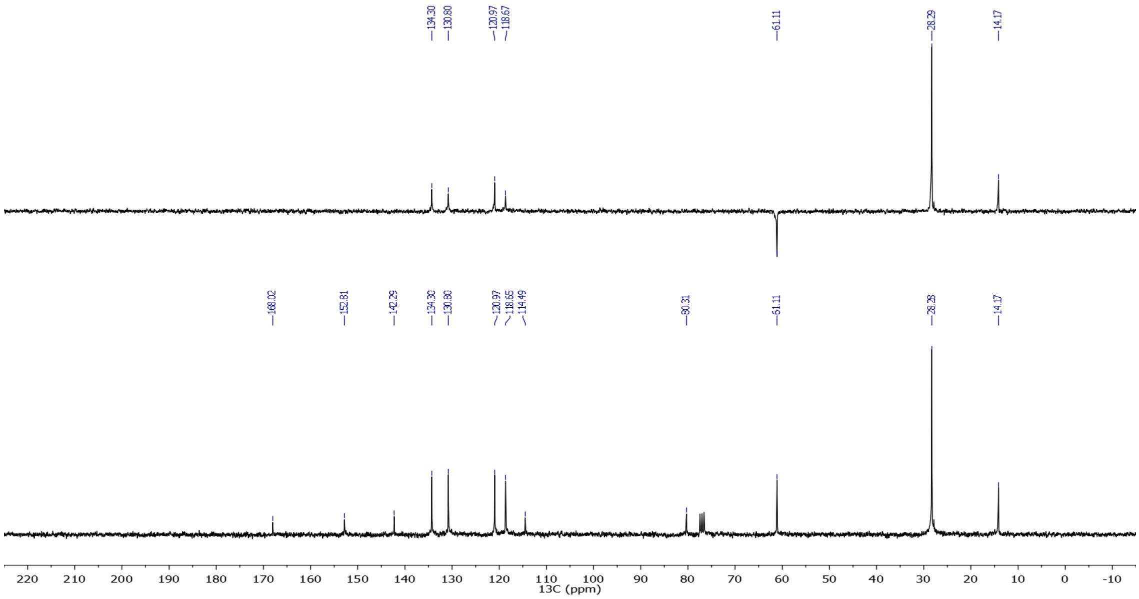

^{1}H-NMR (300 MHz). Solvent $CDCl_3$

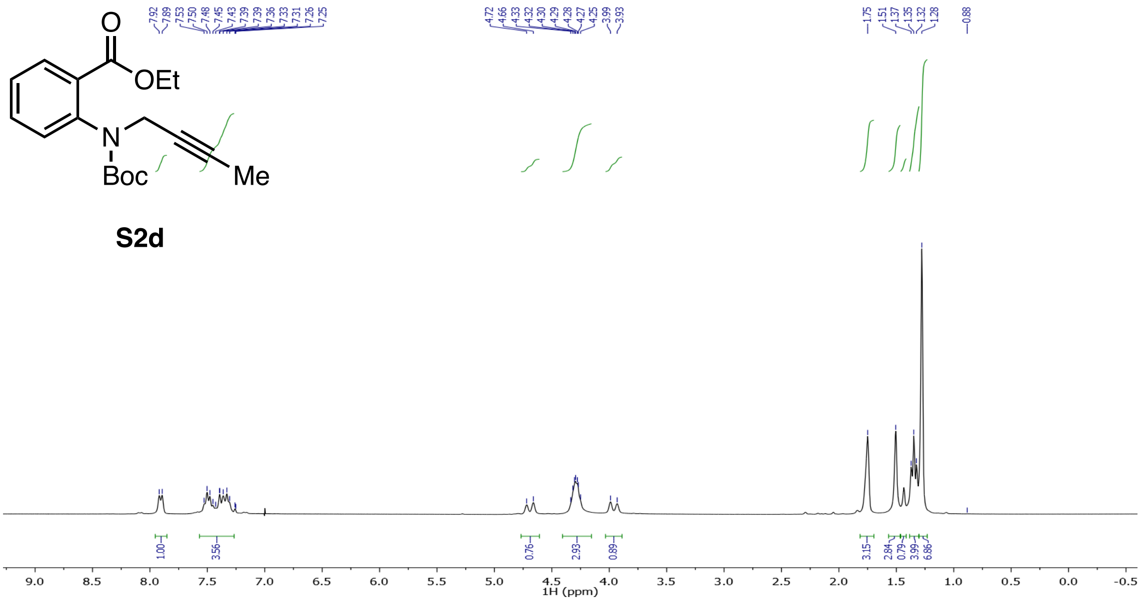

^{113}C-NMR (75 MHz). Solvent $CDCl_3$

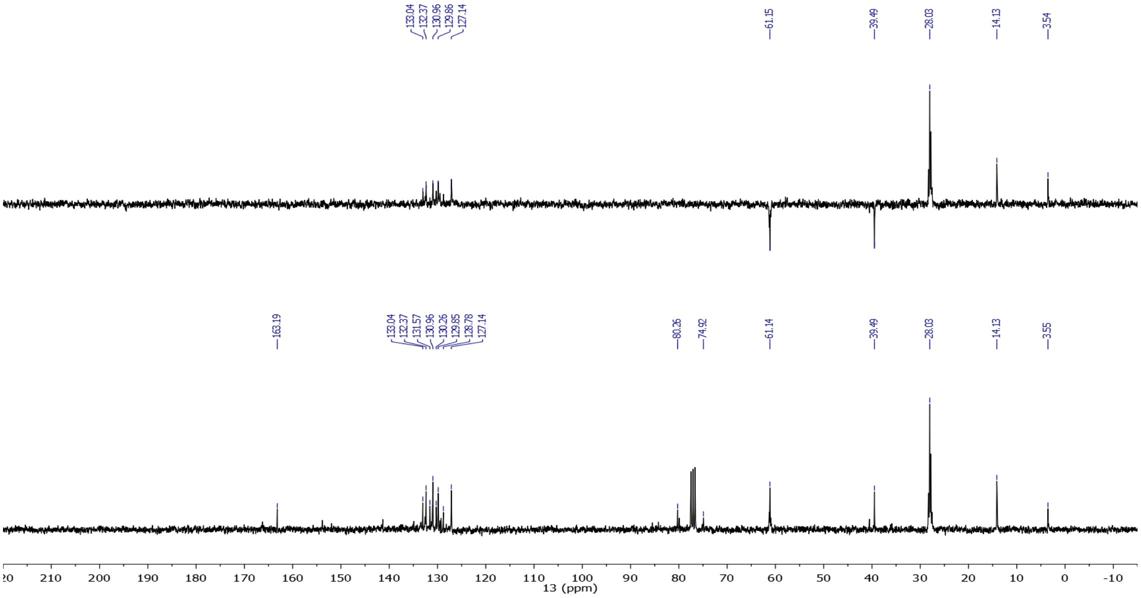

^{1}H-NMR (300 MHz). Solvent $CDCl_3$

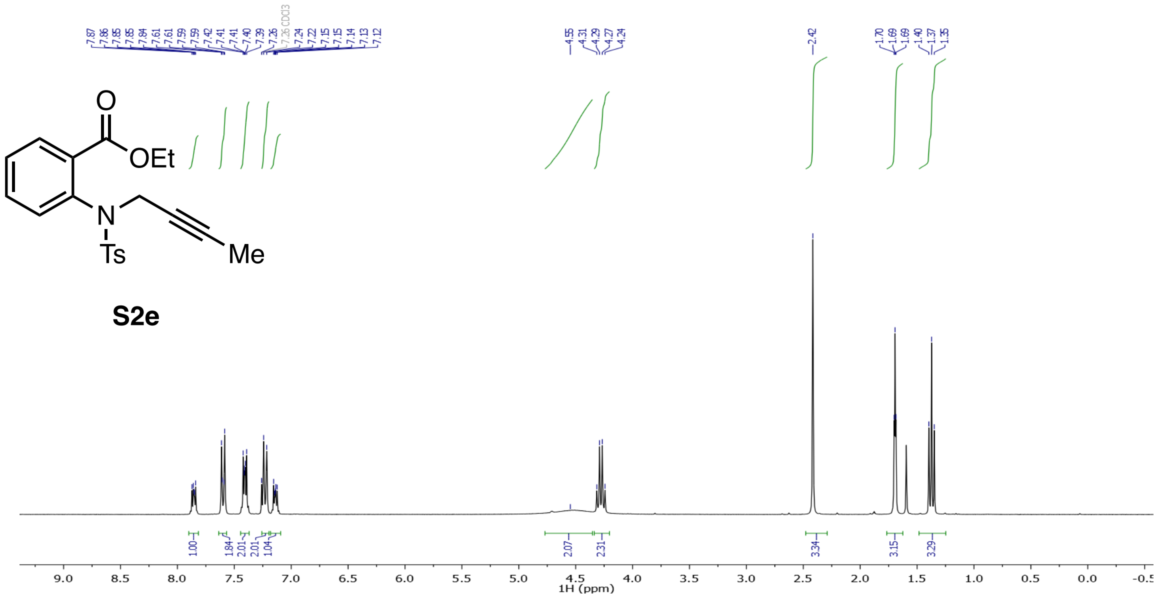

S2e

^{13}C-NMR (75 MHz). Solvent $CDCl_3$

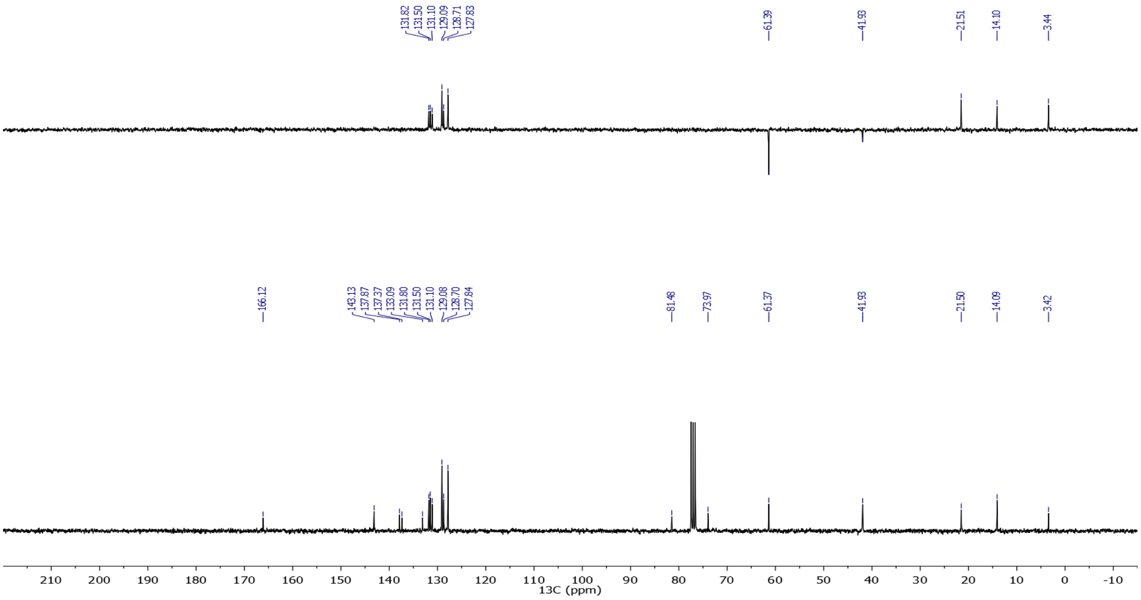

^{1}H-NMR (300 MHz). Solvent $CDCl_3$

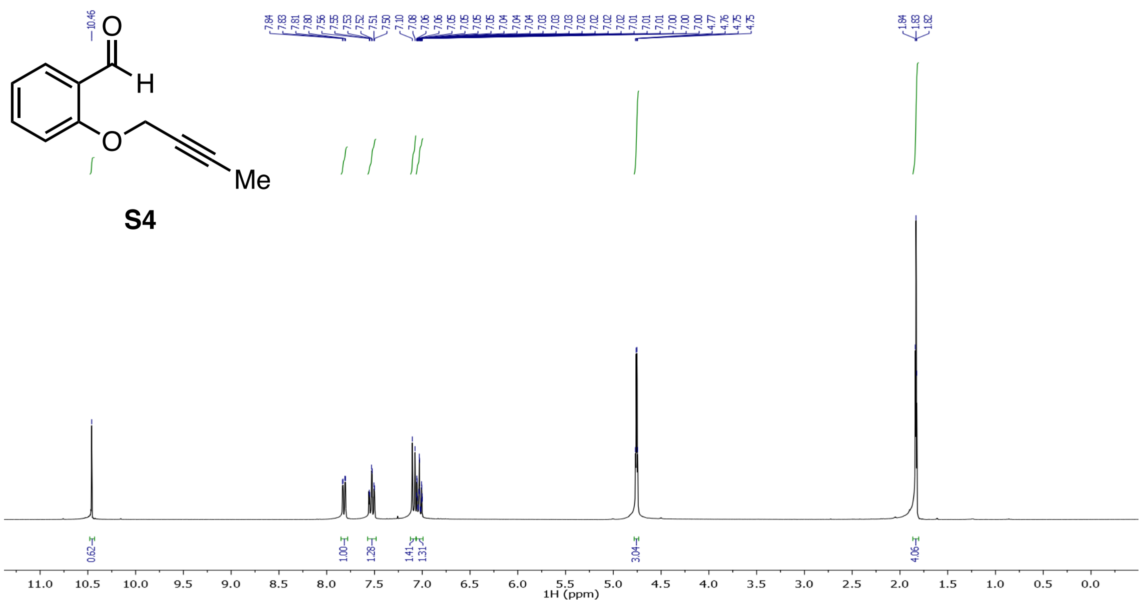

^{13}C-NMR (75 MHz). Solvent $CDCl_3$

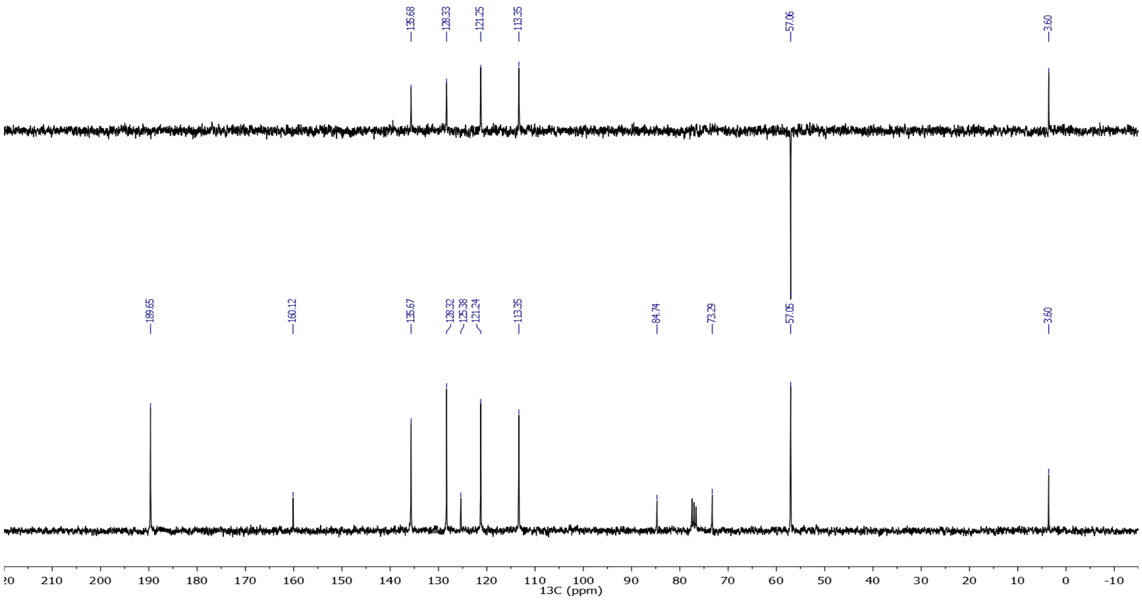

^{1}H-NMR (300 MHz). Solvent $CDCl_3$

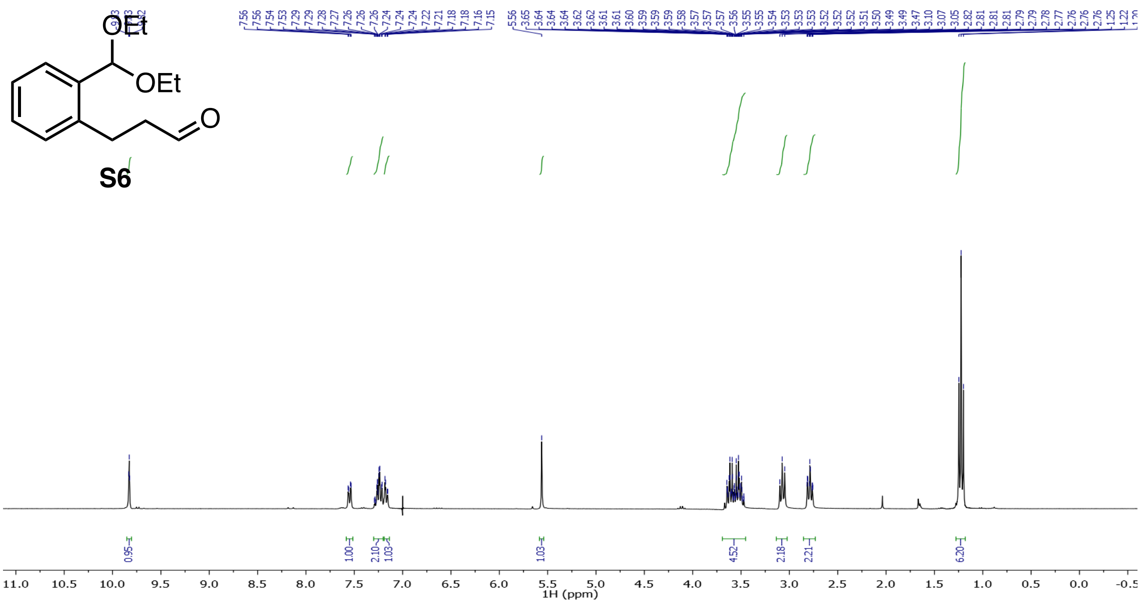

^{13}C-NMR (75 MHz). Solvent $CDCl_3$

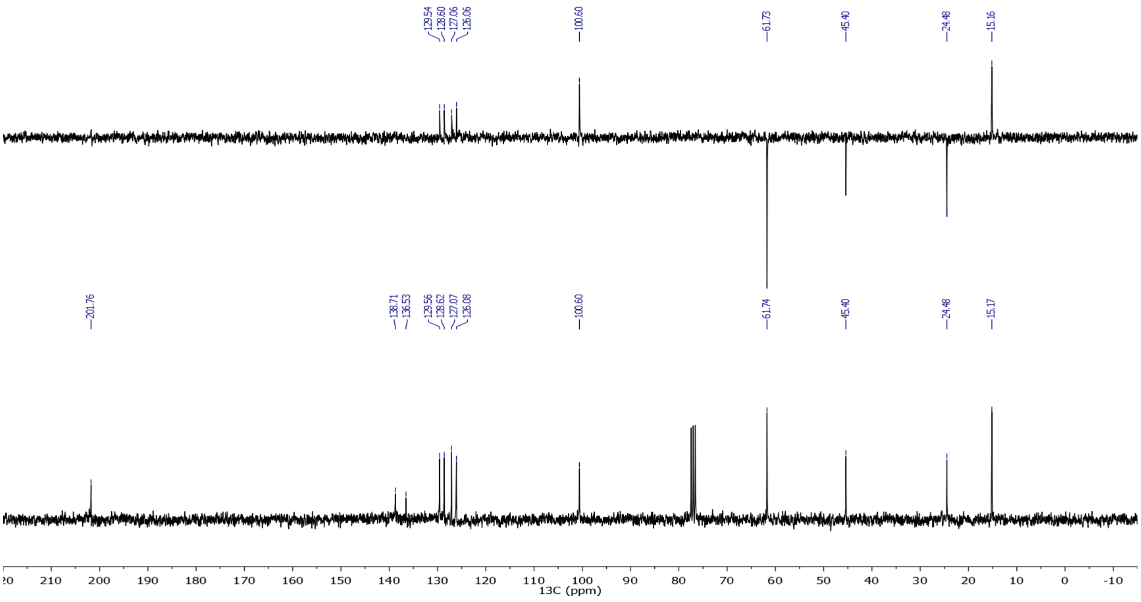

^{1}H-NMR (300 MHz). Solvent $CDCl_3$

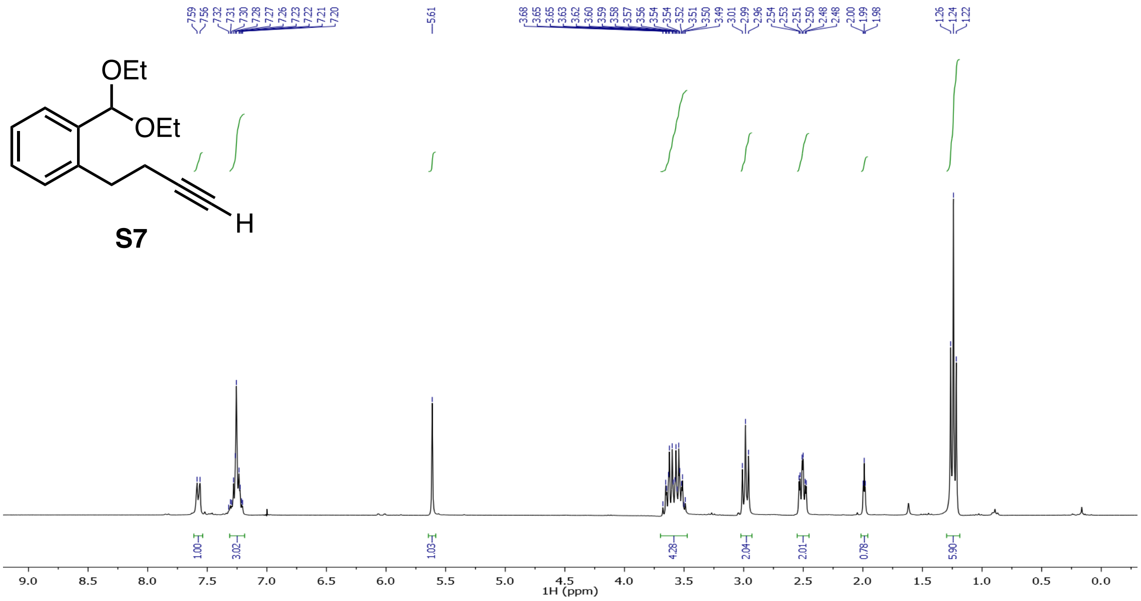

^{13}C-NMR (75MHz). Solvent $CDCl_3$

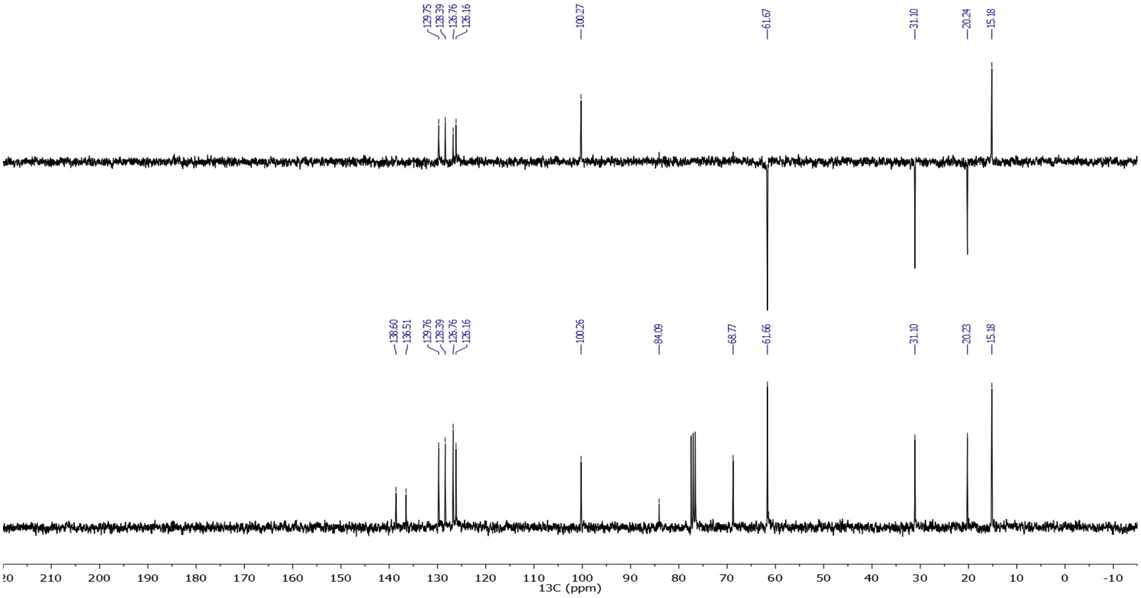

^{1}H-NMR (300 MHz). Solvent $CDCl_3$

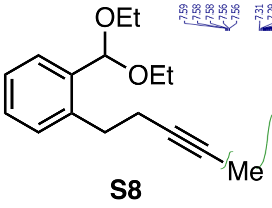

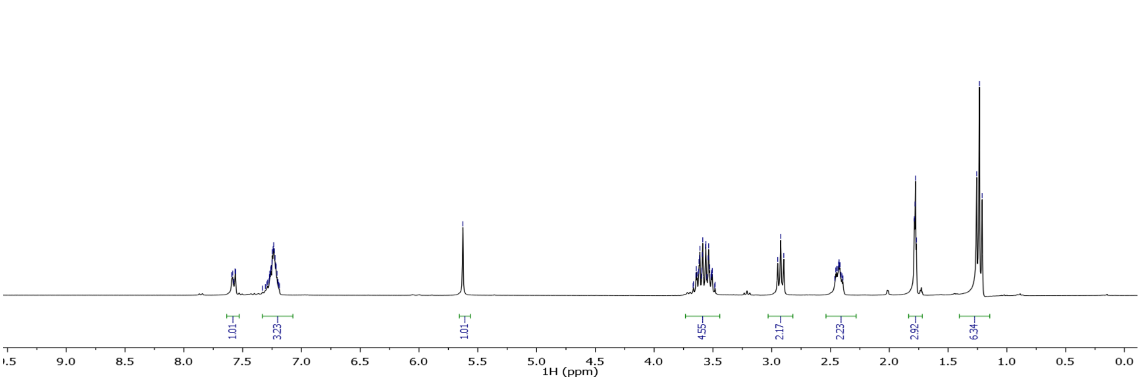

^{13}C-NMR (75 MHz). Solvent $CDCl_3$

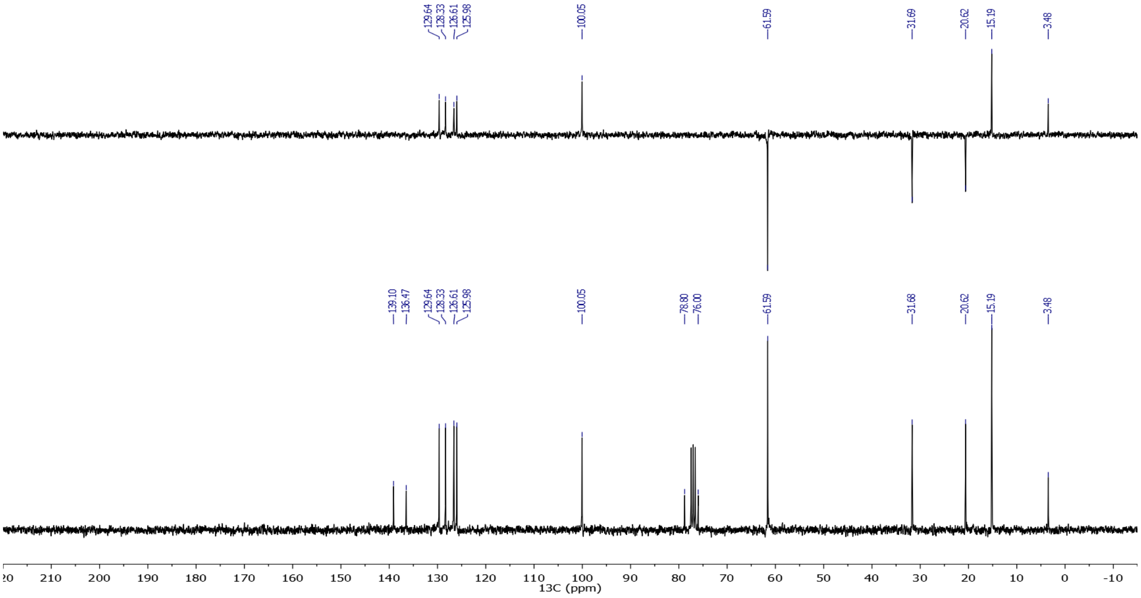

^{1}H-NMR (300 MHz). Solvent $CDCl_3$

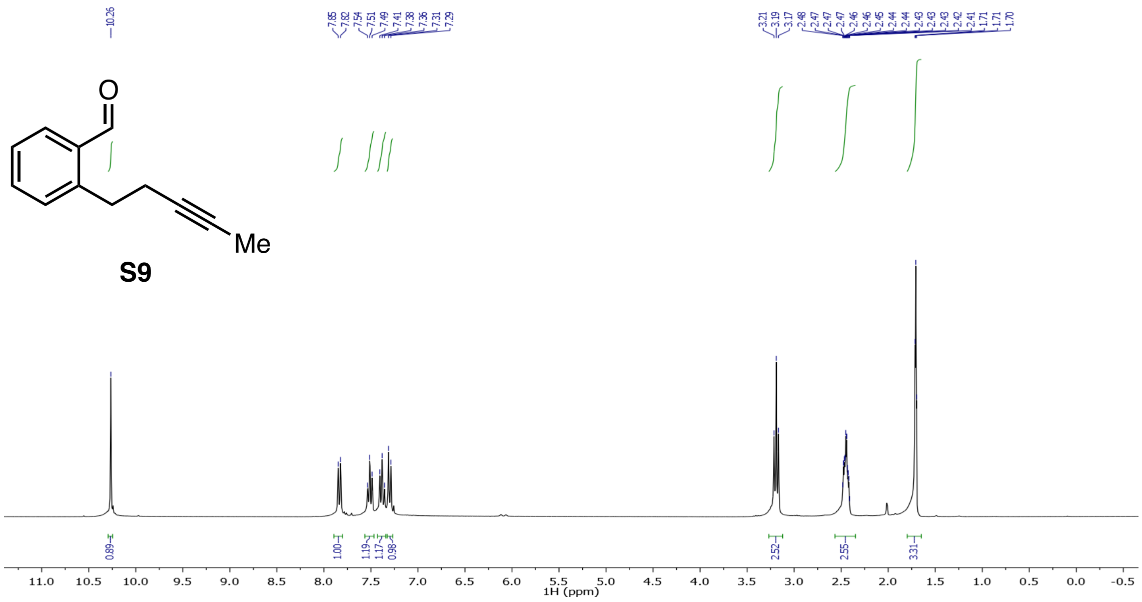

S9

^{13}C-NMR (75 MHz). Solvent $CDCl_3$

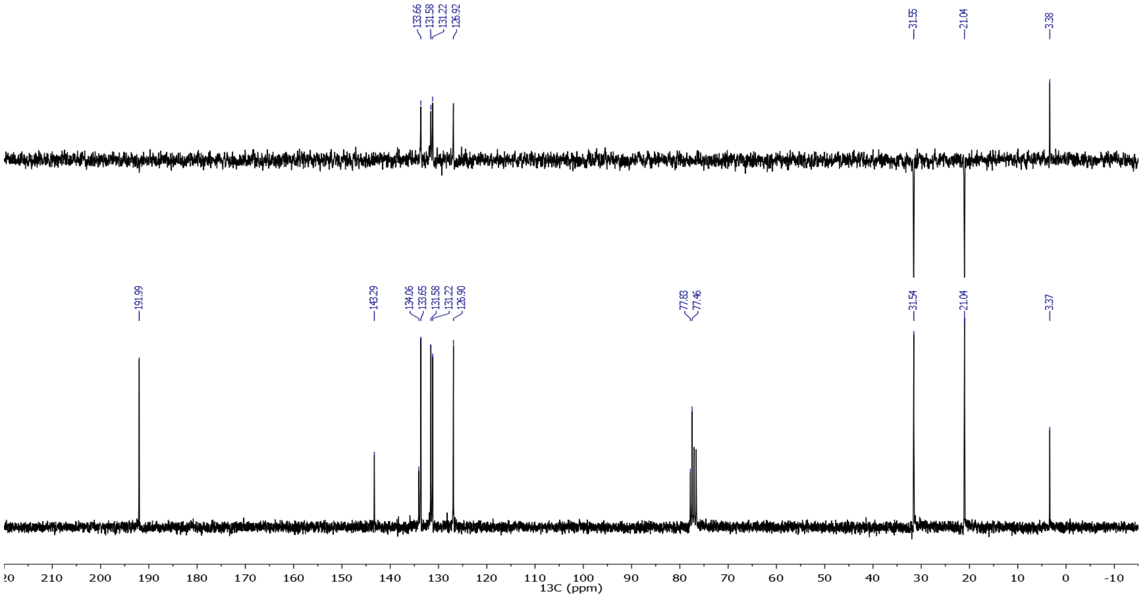

^{1}H-NMR (300 MHz). Solvent $CDCl_3$

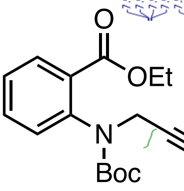

S10d

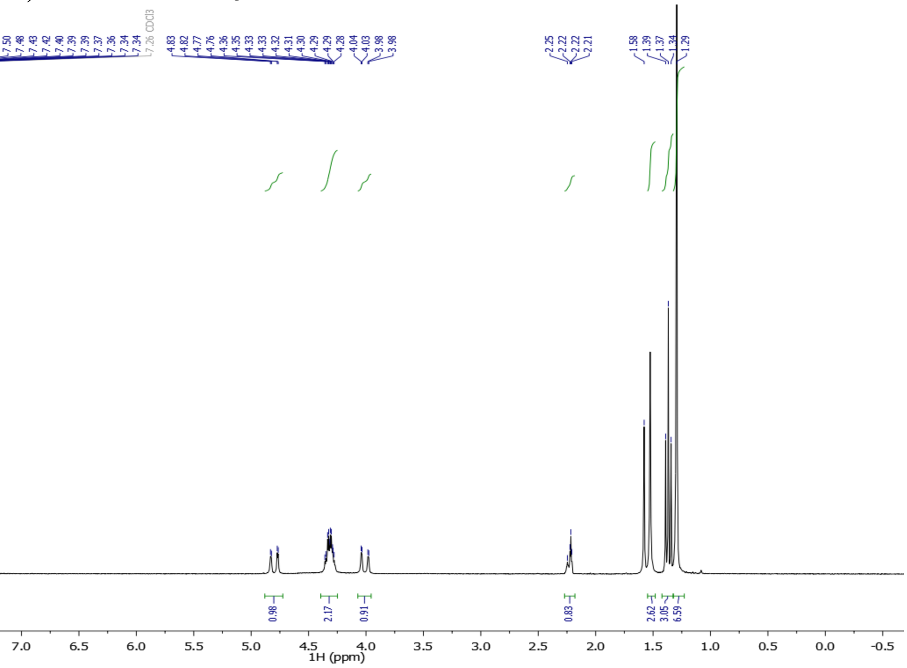

^{13}C-NMR (75 MHz). Solvent $CDCl_3$

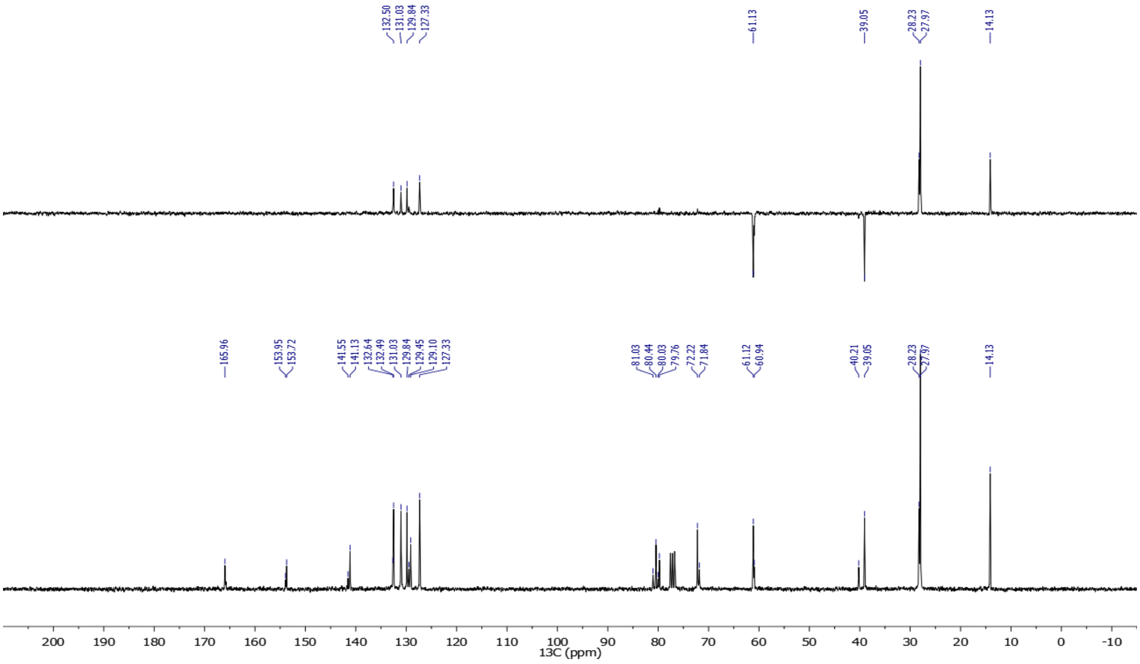

^{1}H-NMR (300 MHz). Solvent $CDCl_3$

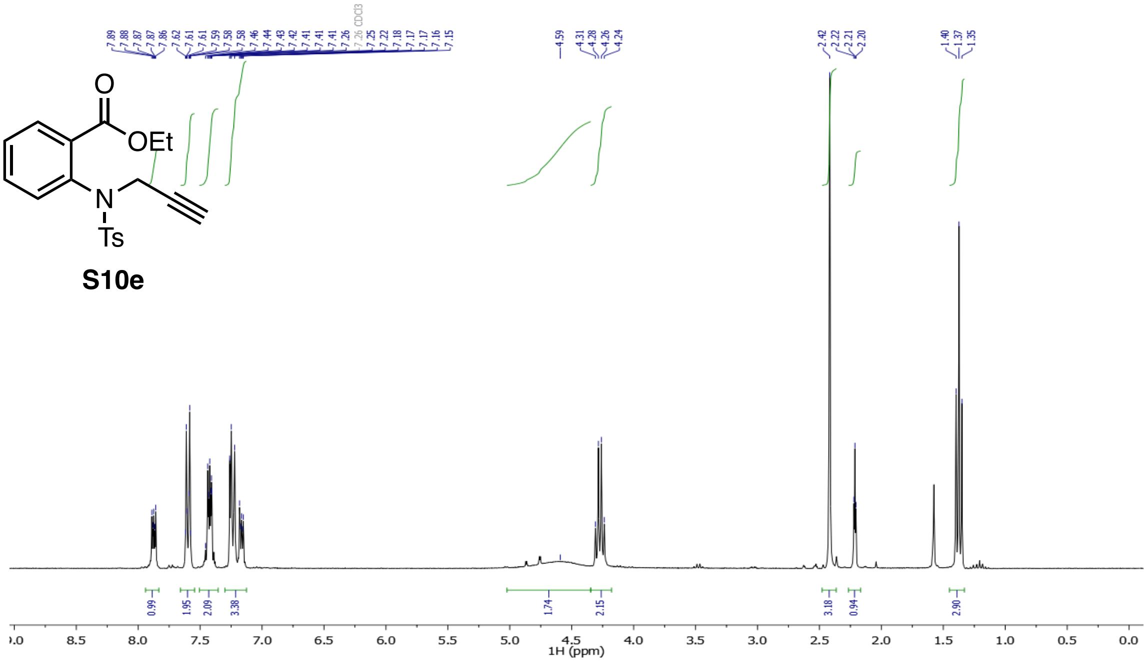

S10e

^{13}C-NMR (75 MHz). Solvent $CDCl_3$

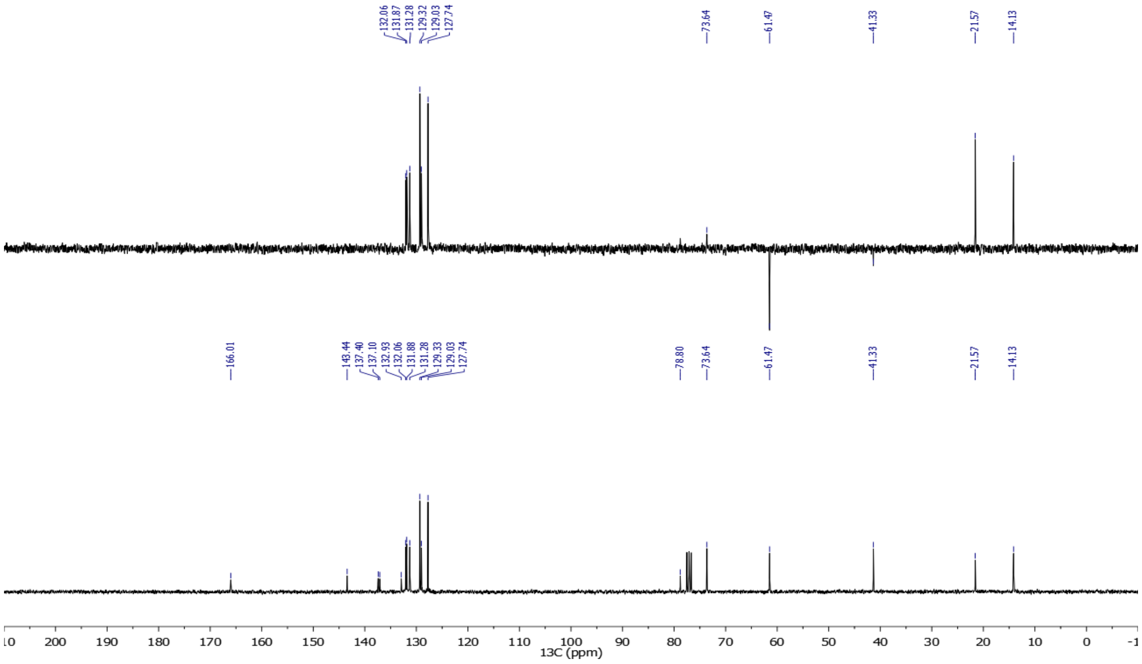

^{1}H-NMR (300 MHz). Solvent $CDCl_3$

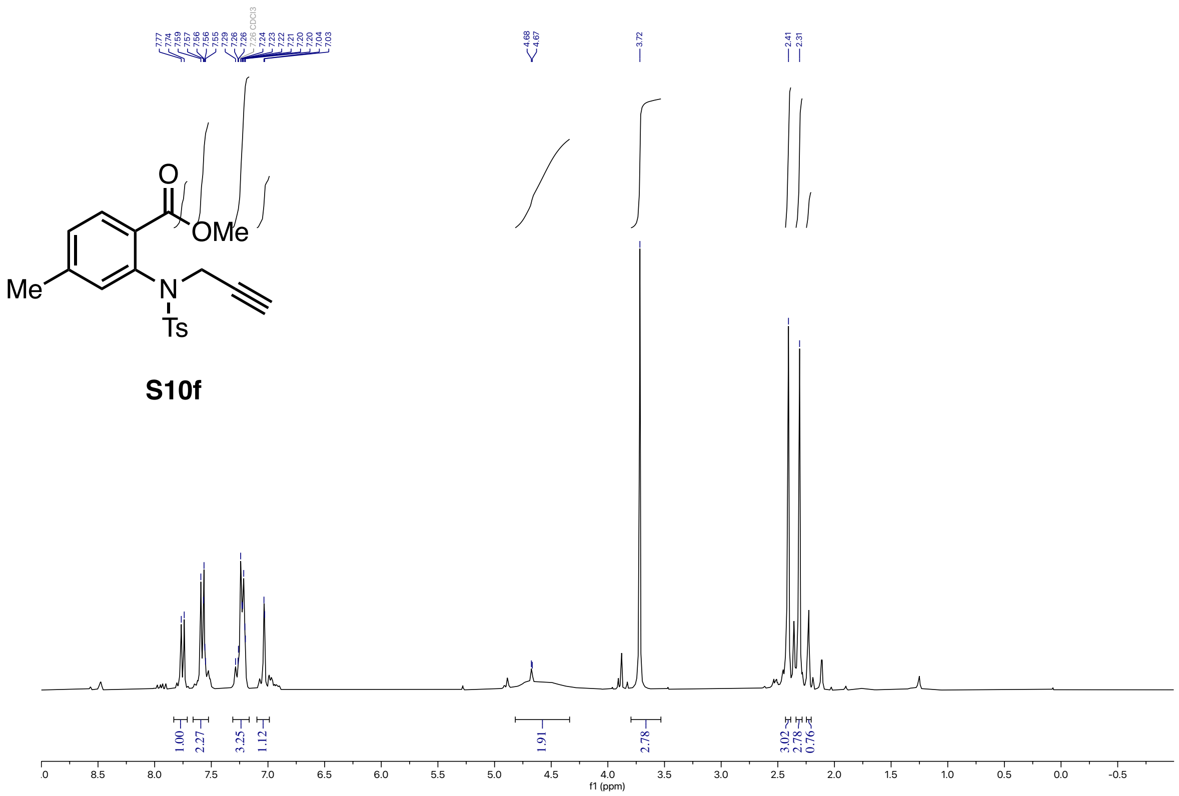

^{13}C-NMR (75 MHz). Solvent $CDCl_3$

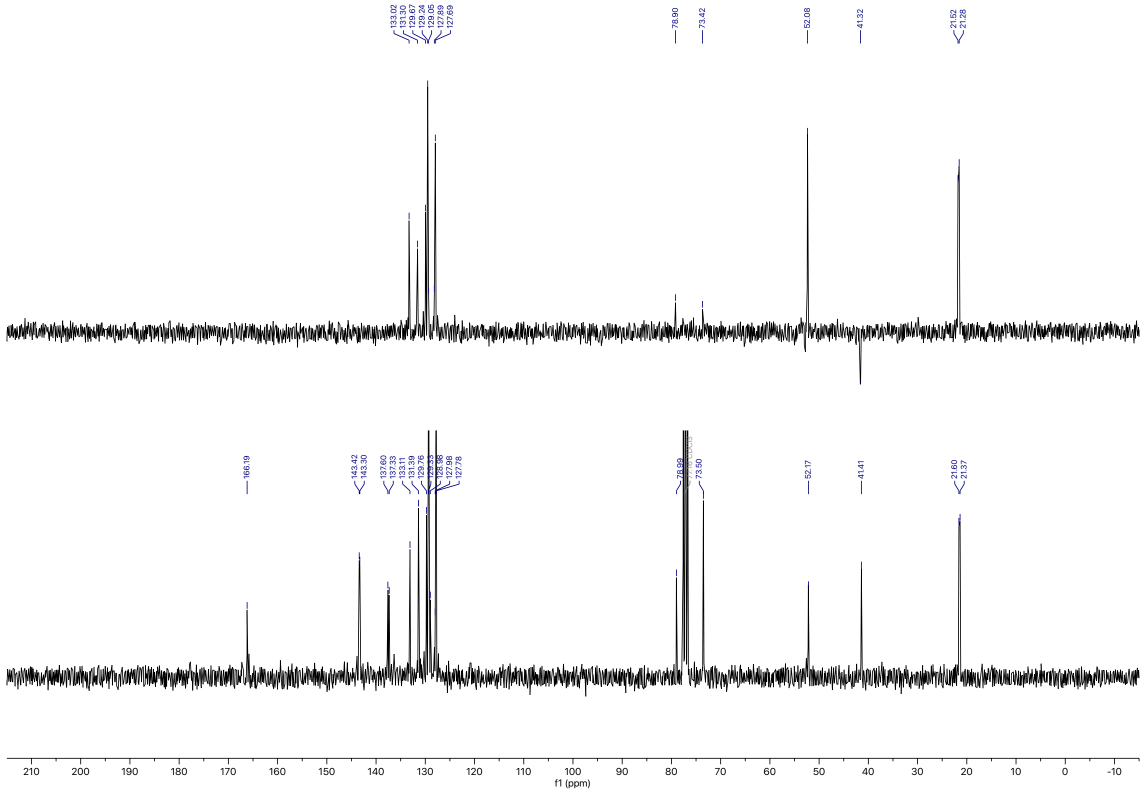

^{1}H-NMR (300 MHz). Solvent $CDCl_3$

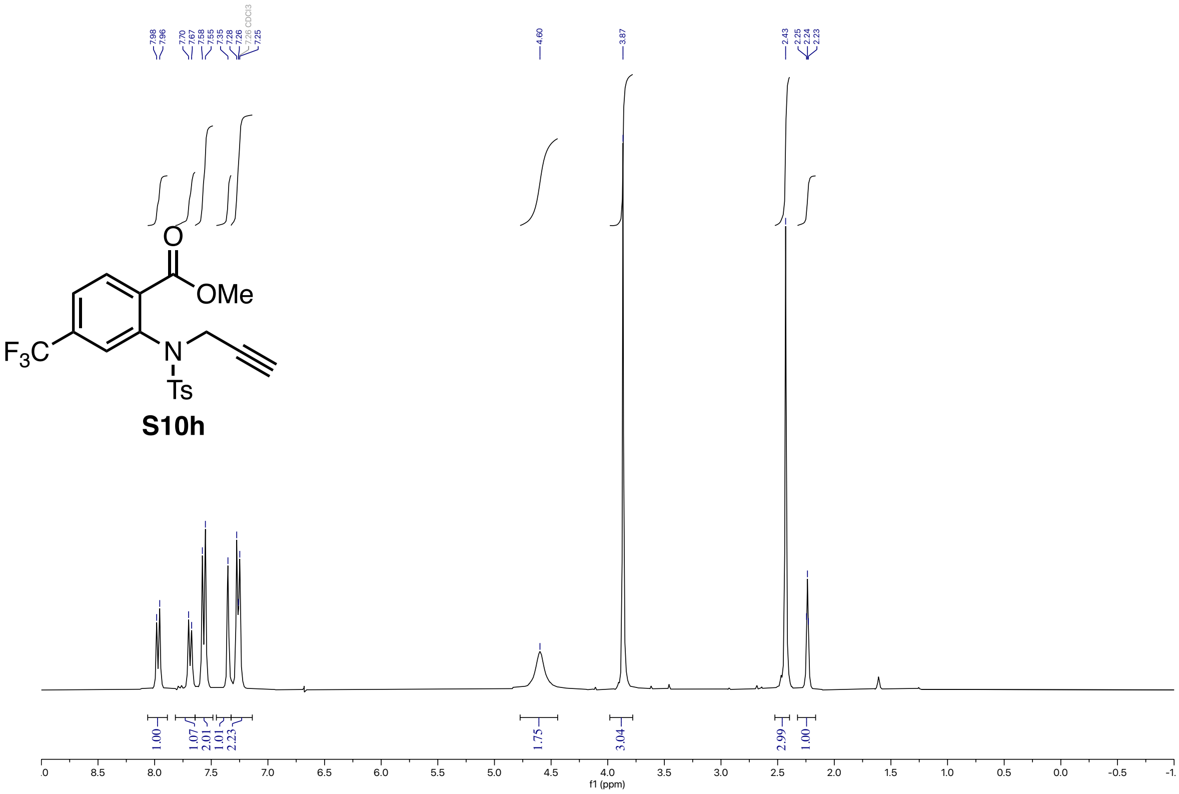

^{13}C-NMR (75 MHz). Solvent $CDCl_3$

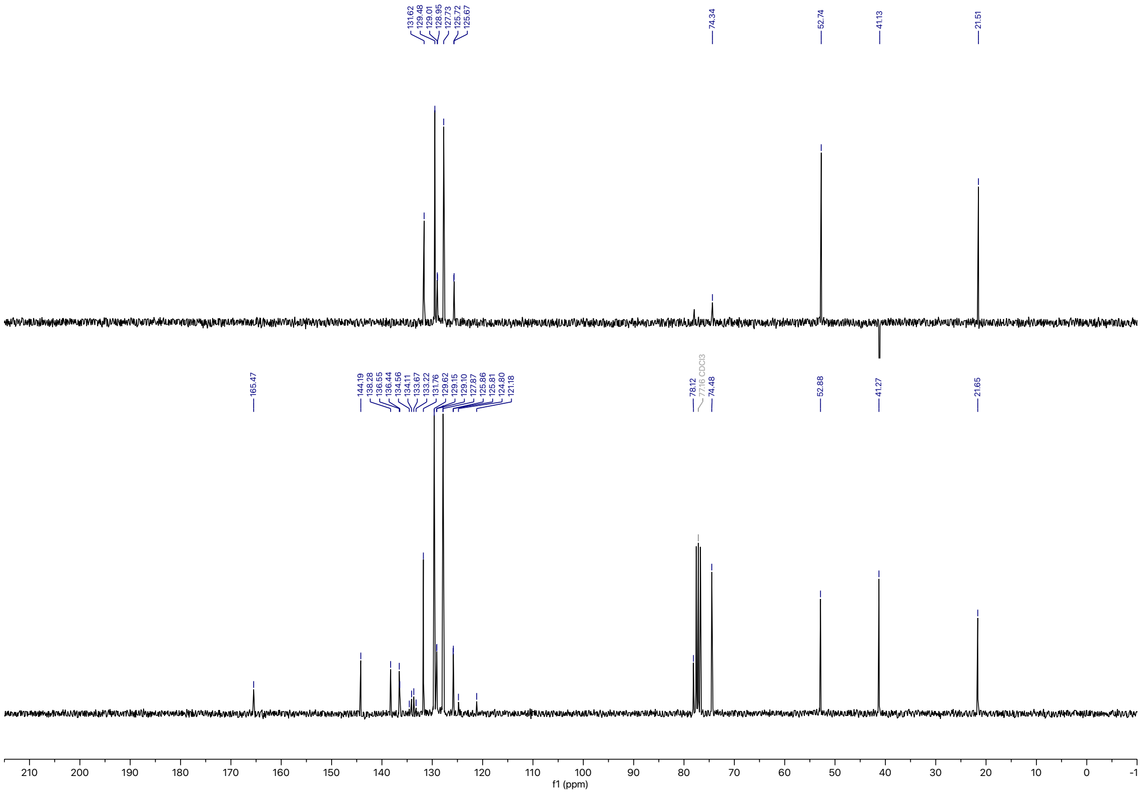

^{19}F-NMR (282 MHz). Solvent $CDCl_3$

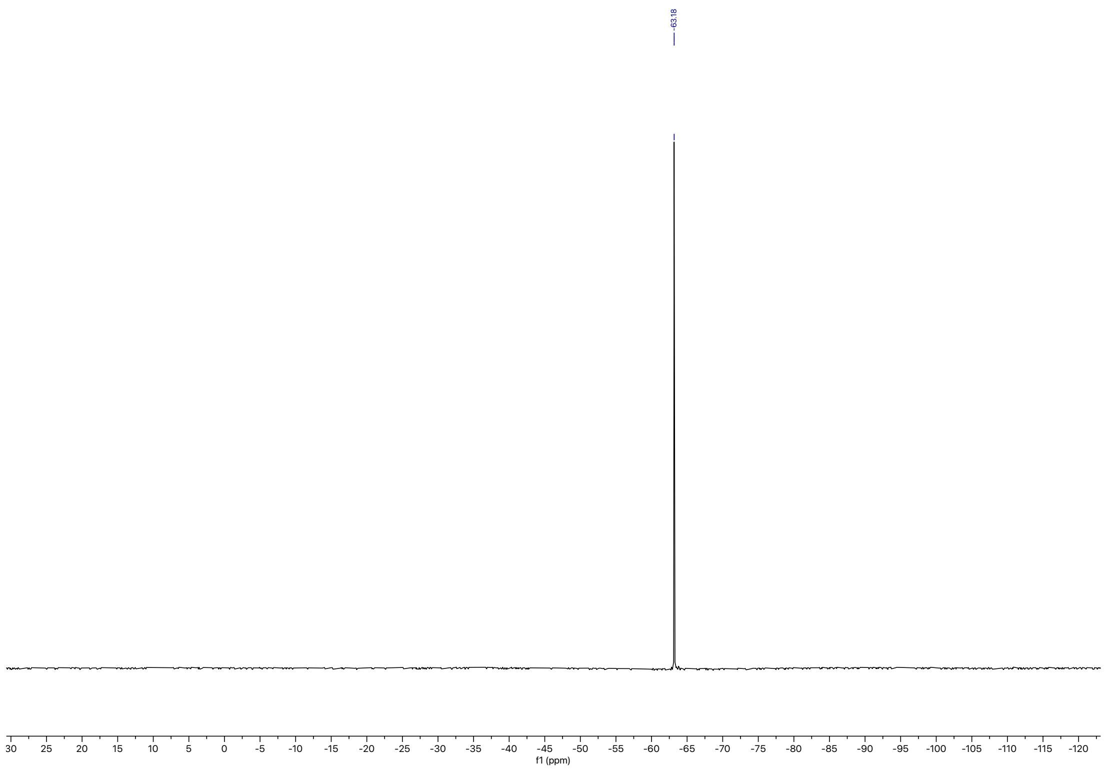

^{1}H-NMR (300 MHz). Solvent $CDCl_3$

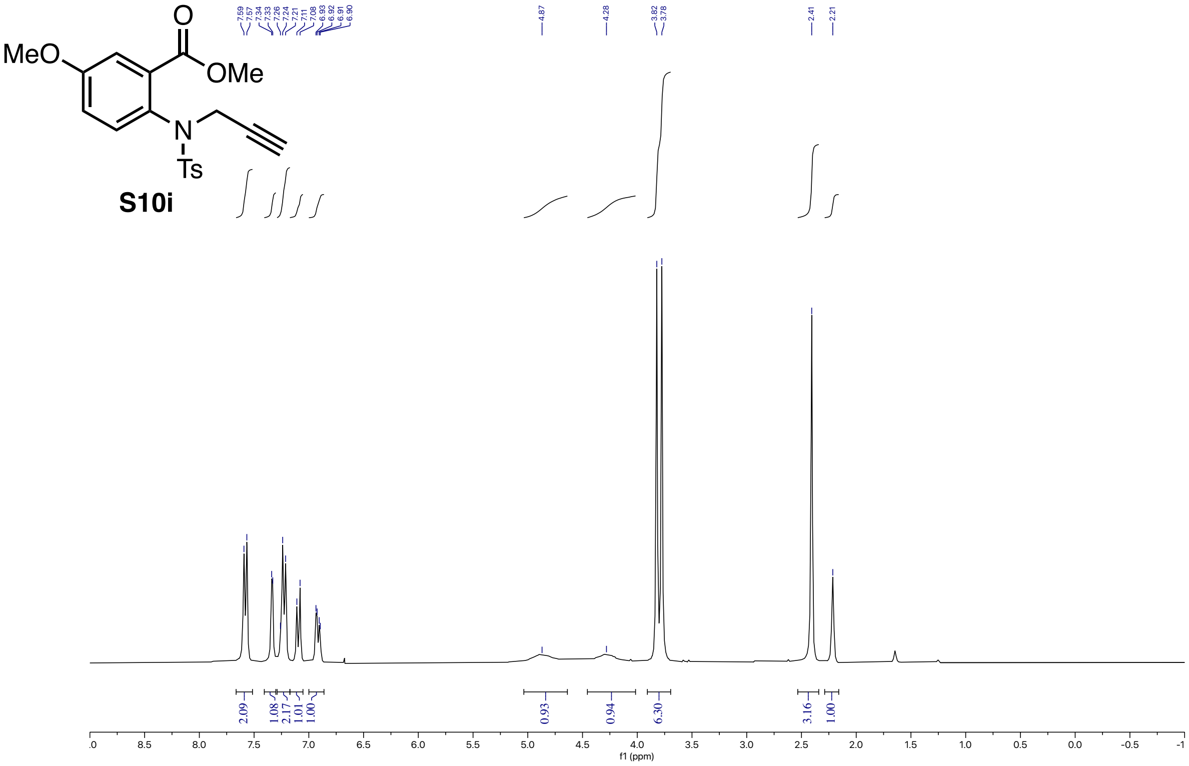

S10i

^{13}C-NMR (75 MHz). Solvent $CDCl_3$

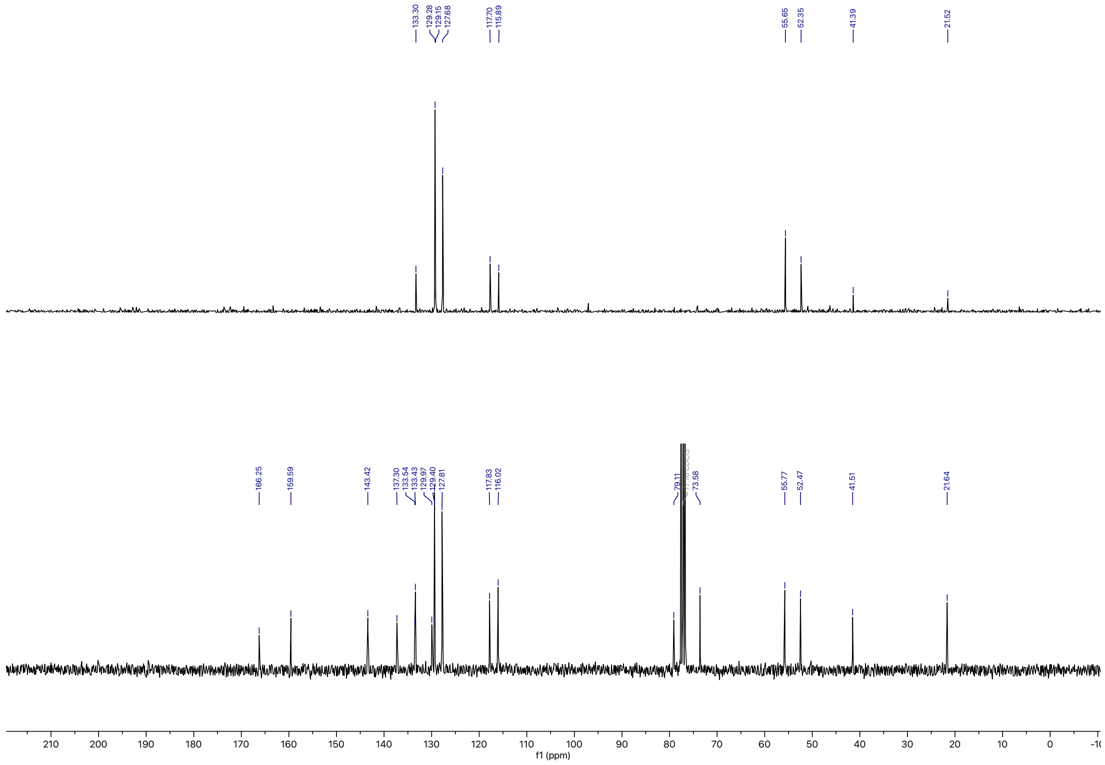

^{1}H-NMR (300 MHz). Solvent $CDCl_3$

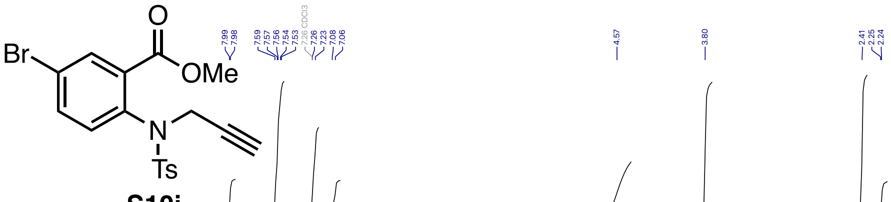

S10j

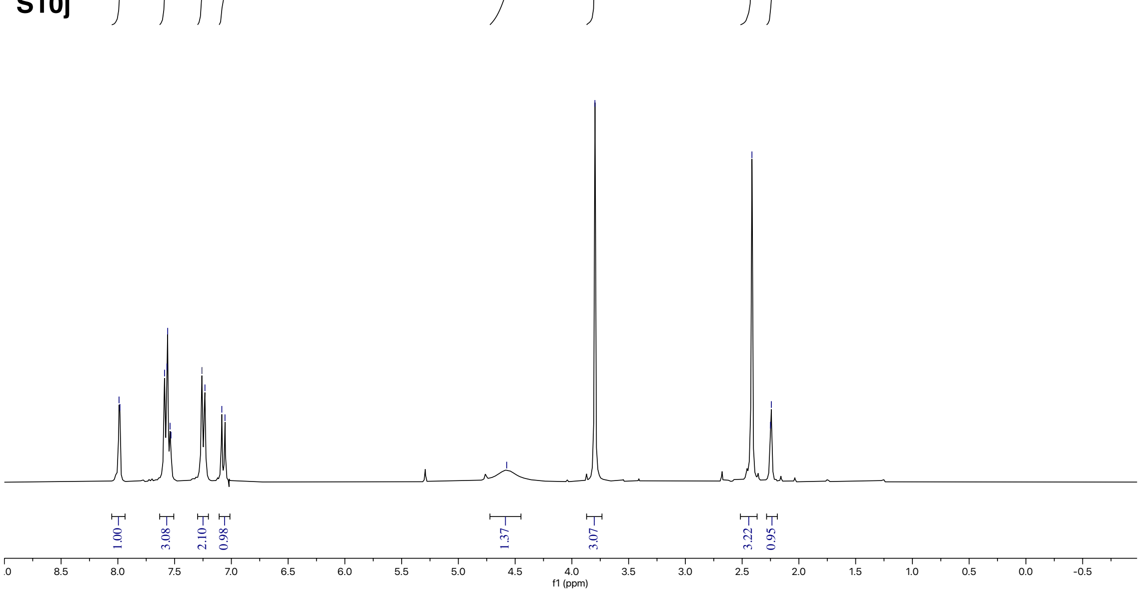

^{13}C-NMR (75 MHz). Solvent $CDCl_3$

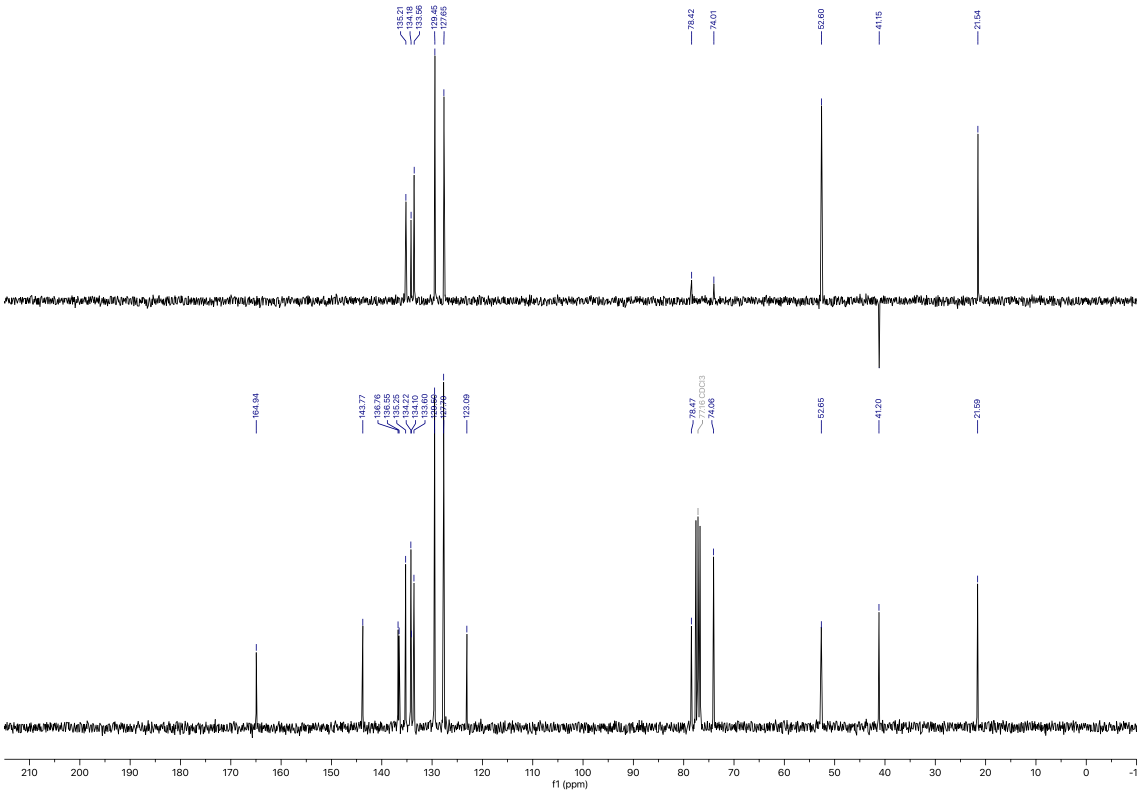

^{1}H-NMR (300 MHz). Solvent $CDCl_3$

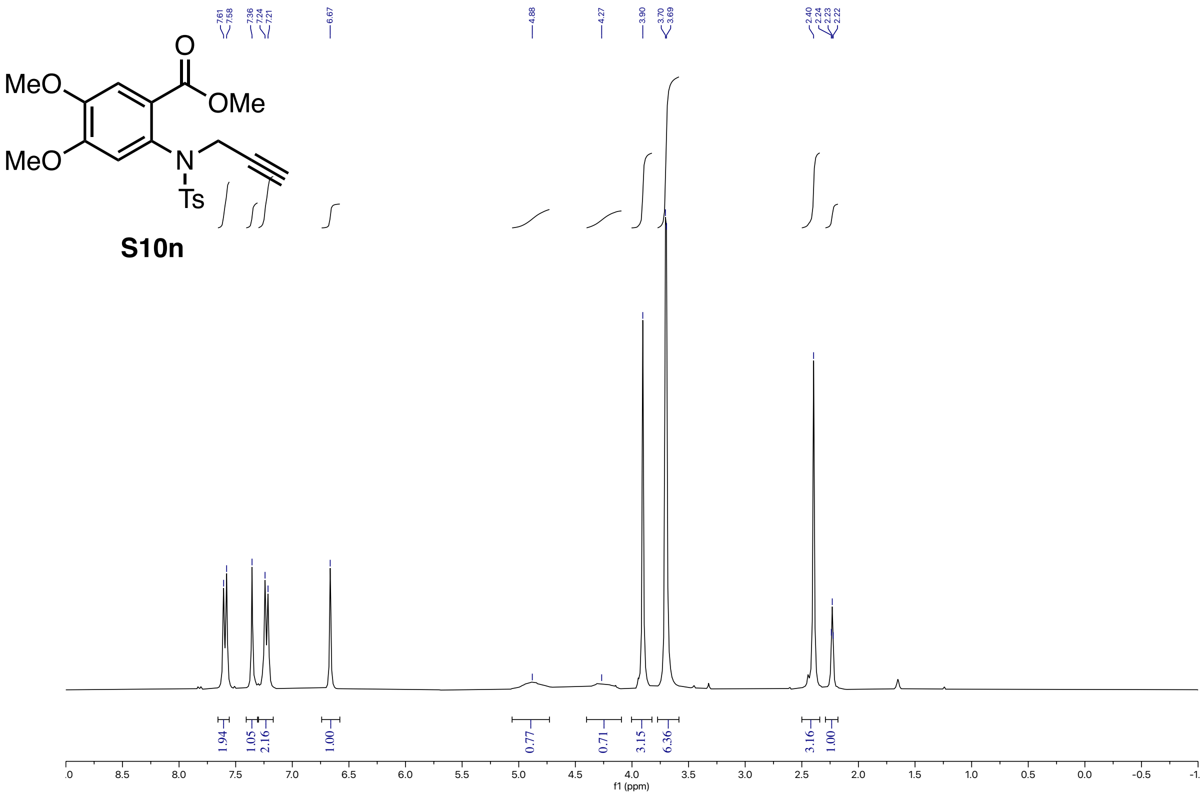

^{13}C-NMR (75 MHz). Solvent $CDCl_3$

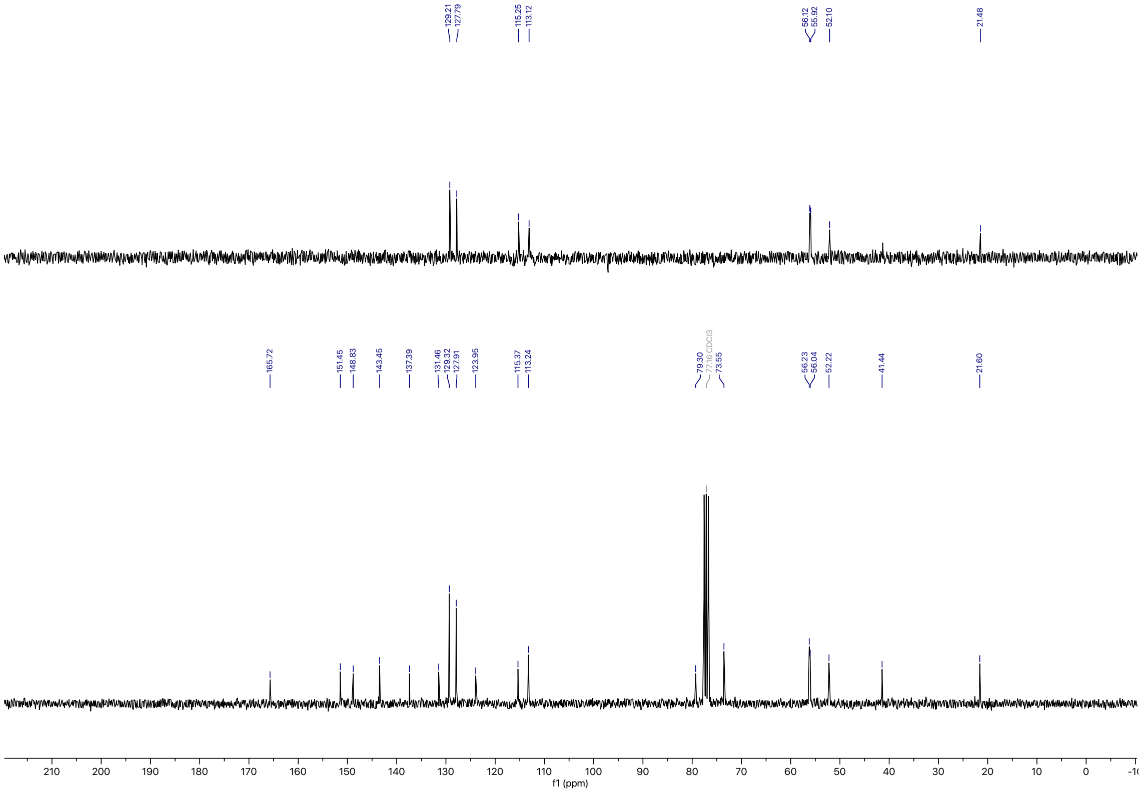

^{1}H-NMR (300 MHz). Solvent $CDCl_3$

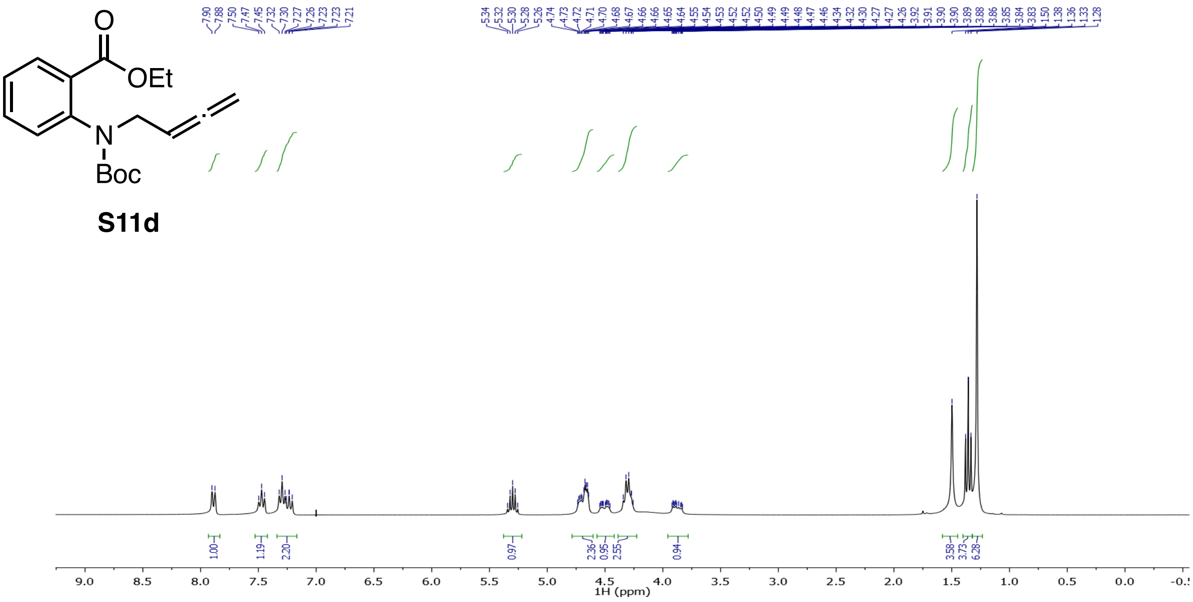

S11d

^{13}C-NMR (75 MHz). Solvent $CDCl_3$

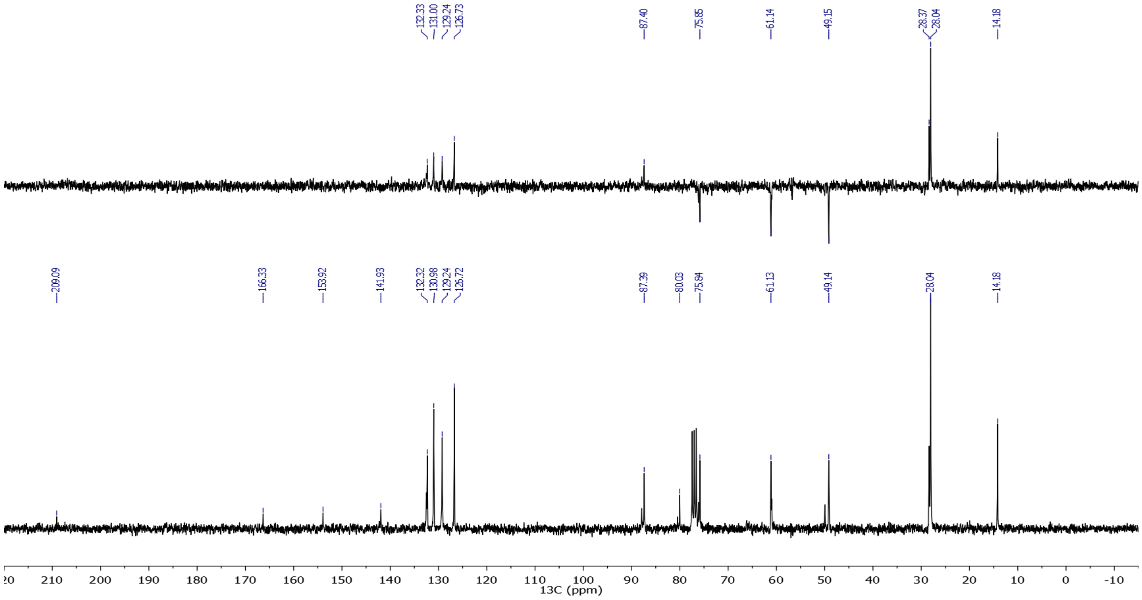

^{1}H-NMR (500 MHz). Solvent $CDCl_3$

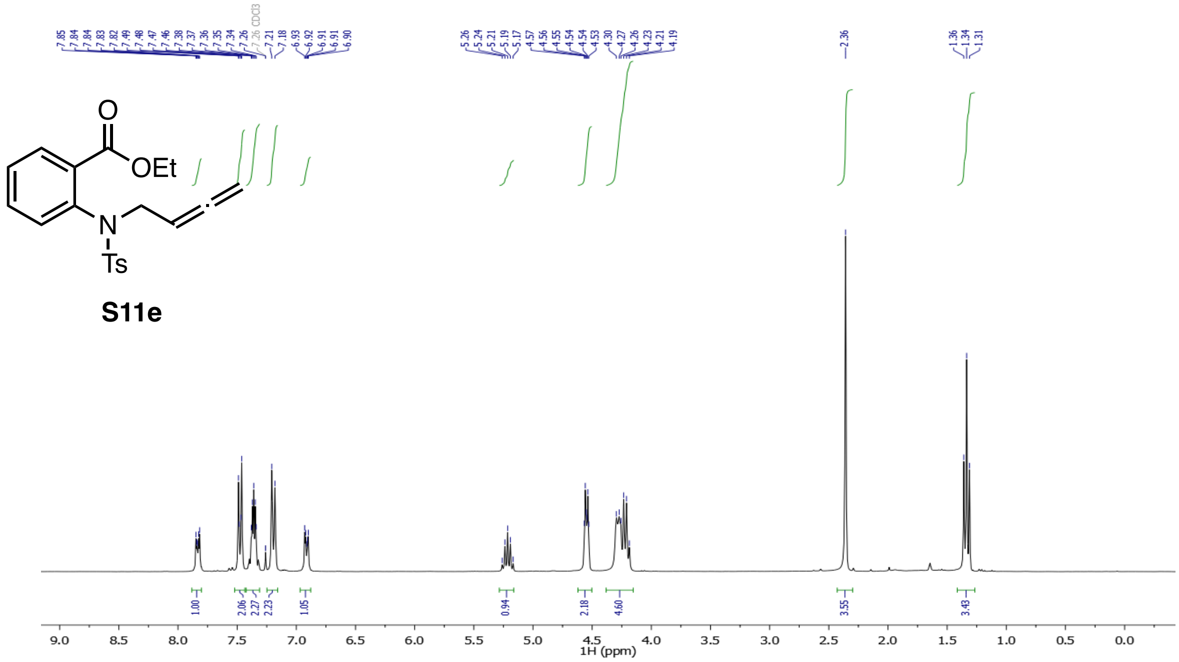

S11e

^{13}C-NMR (126 MHz). Solvent $CDCl_3$

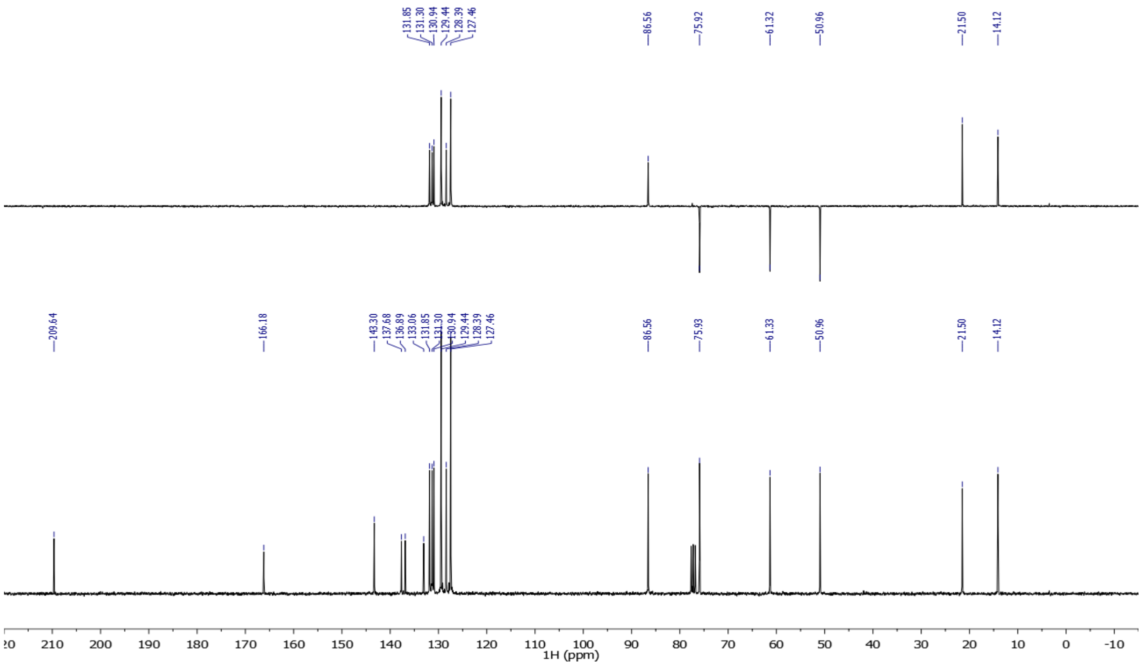

^{1}H-NMR (300 MHz). Solvent $CDCl_3$

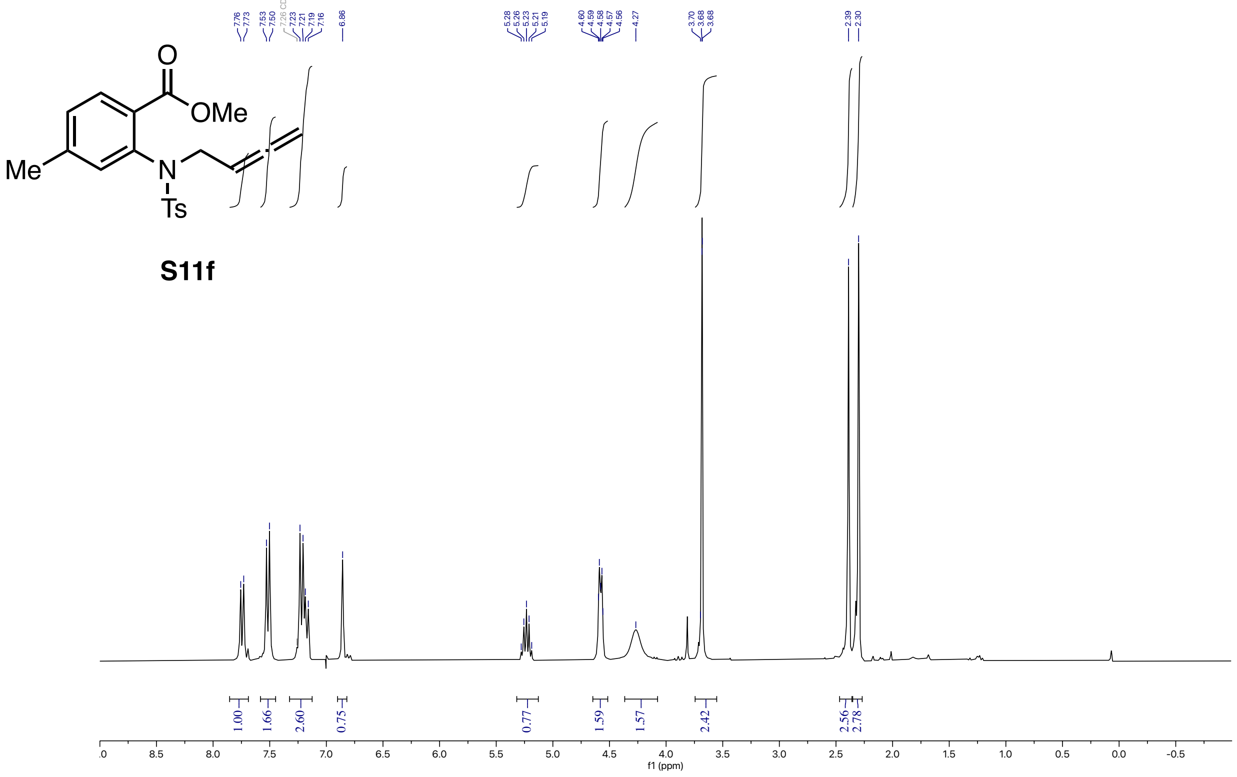

S11f

^{13}C-NMR (75 MHz). Solvent $CDCl_3$

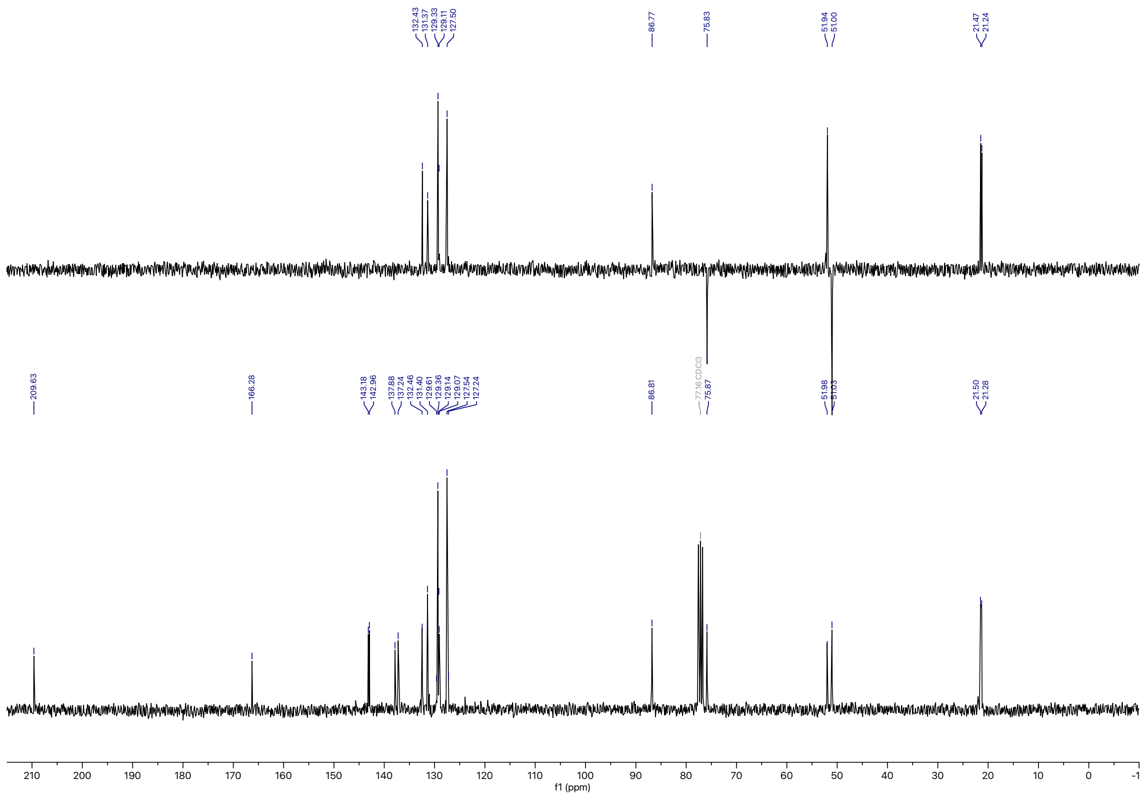

^{1}H-NMR (500 MHz). Solvent $CDCl_3$

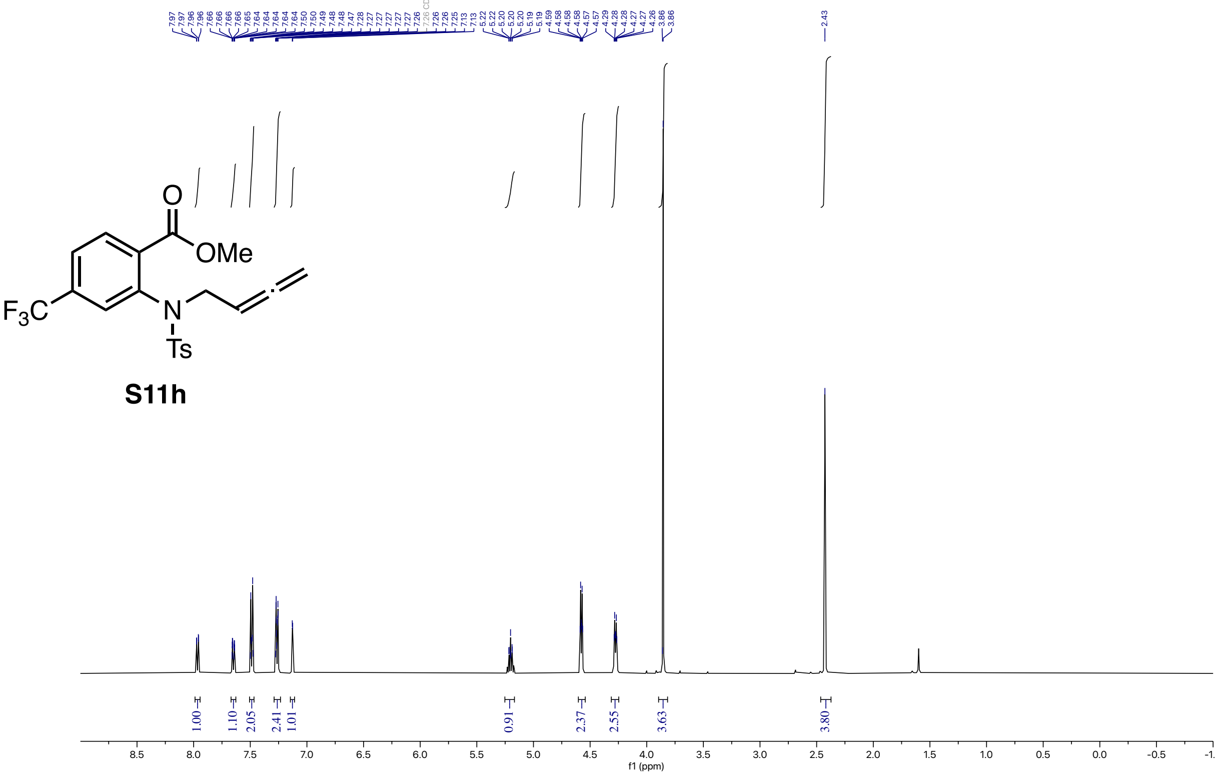

^{13}C-NMR (126 MHz). Solvent $CDCl_3$

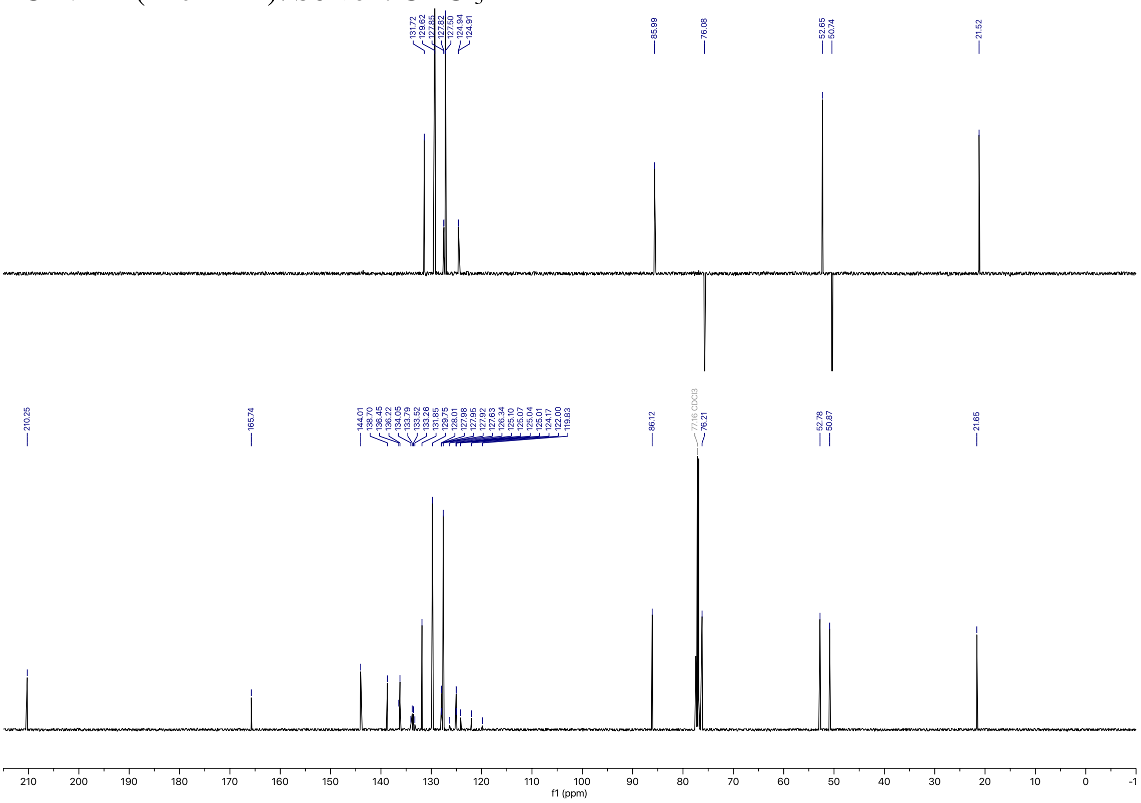

^{19}F-NMR (282 MHz). Solvent $CDCl_3$

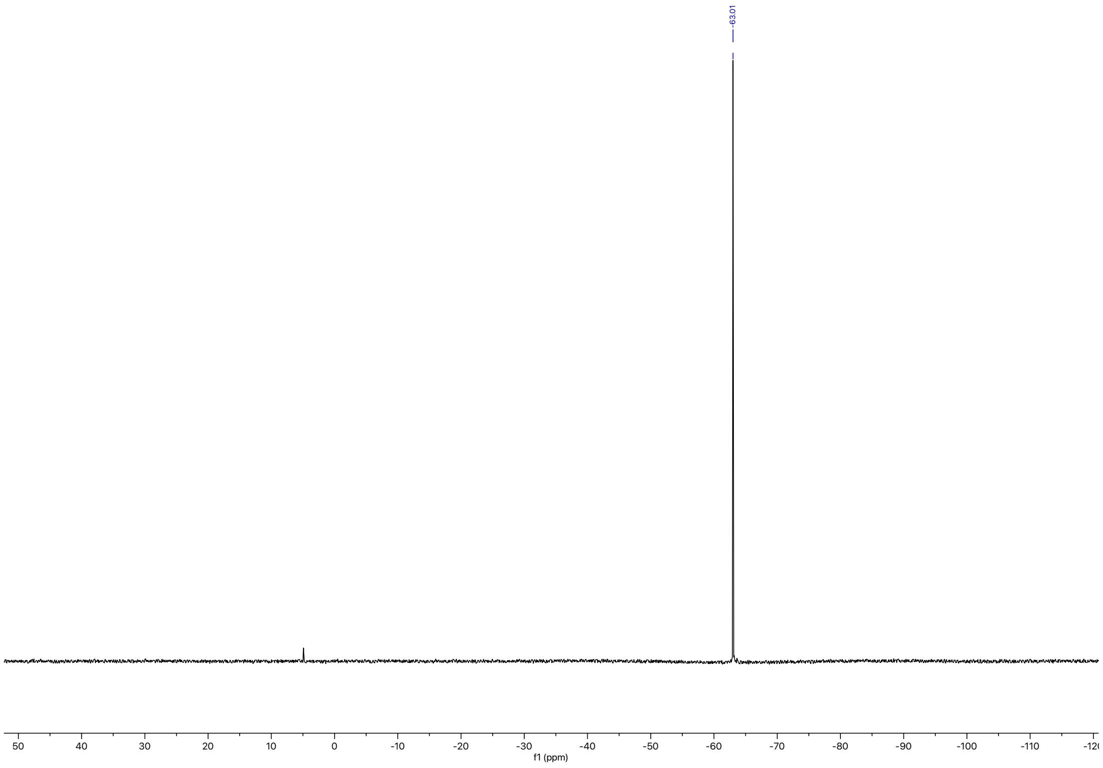

^{1}H-NMR (500 MHz). Solvent $CDCl_3$

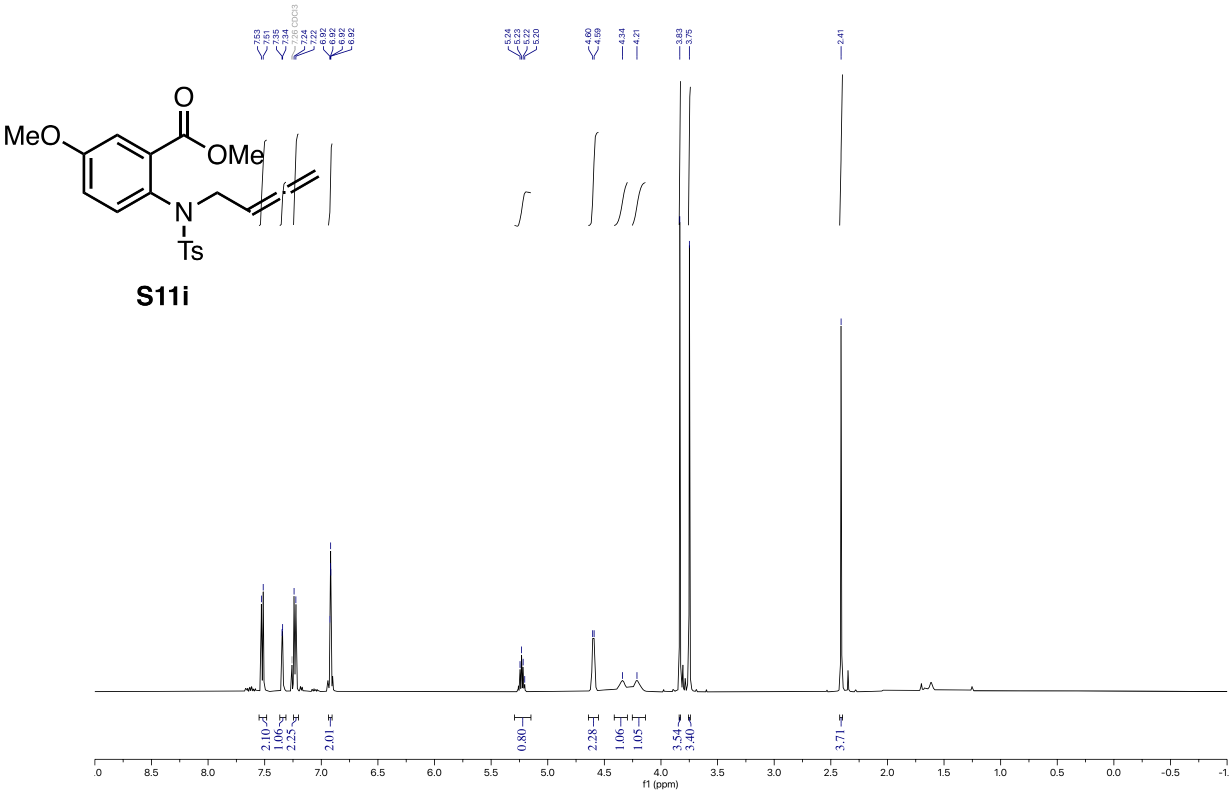

S11i

^{13}C-NMR (126 MHz). Solvent $CDCl_3$

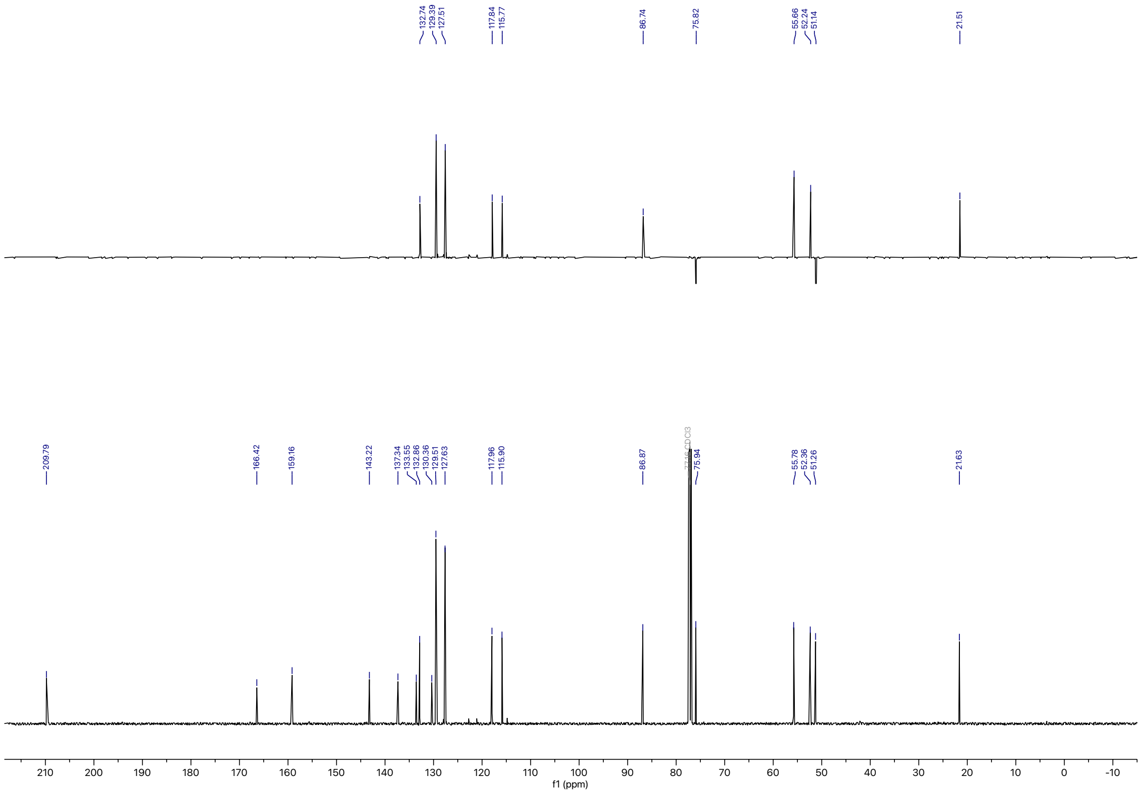

^{1}H-NMR (300 MHz). Solvent $CDCl_3$

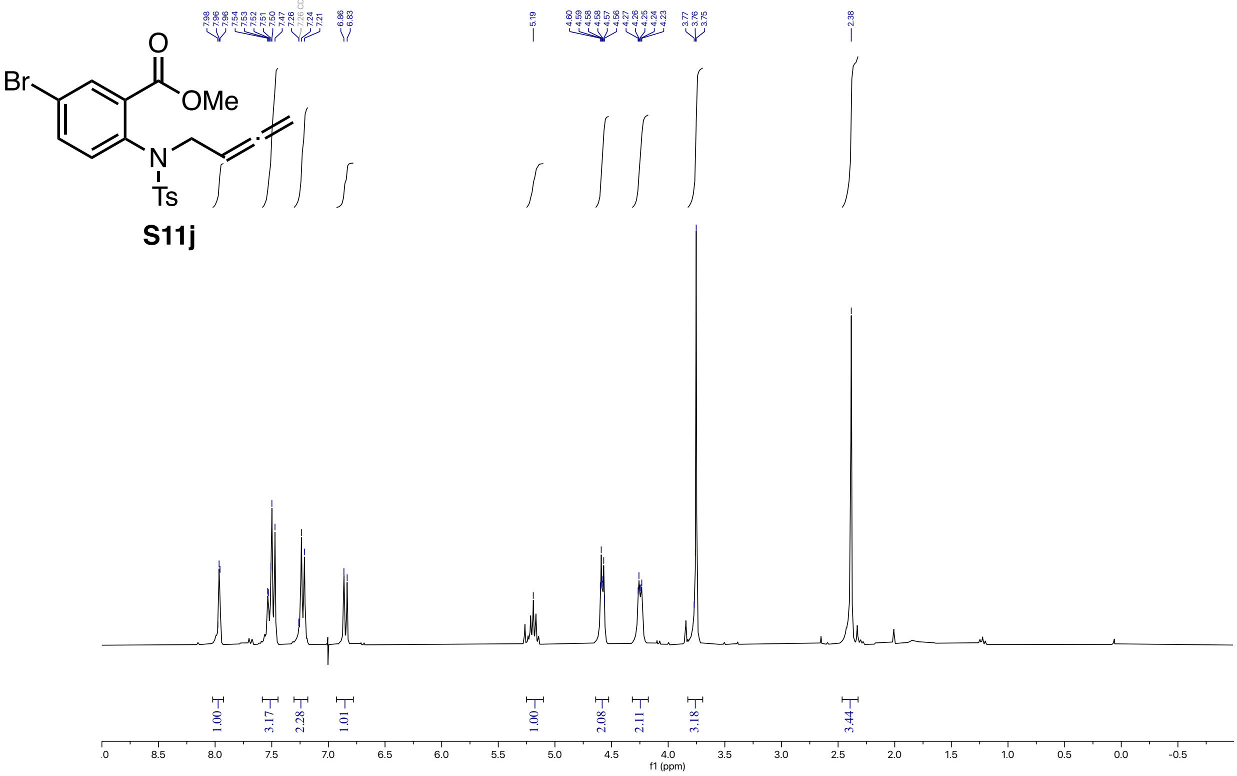

S11j

^{13}C-NMR (75 MHz). Solvent $CDCl_3$

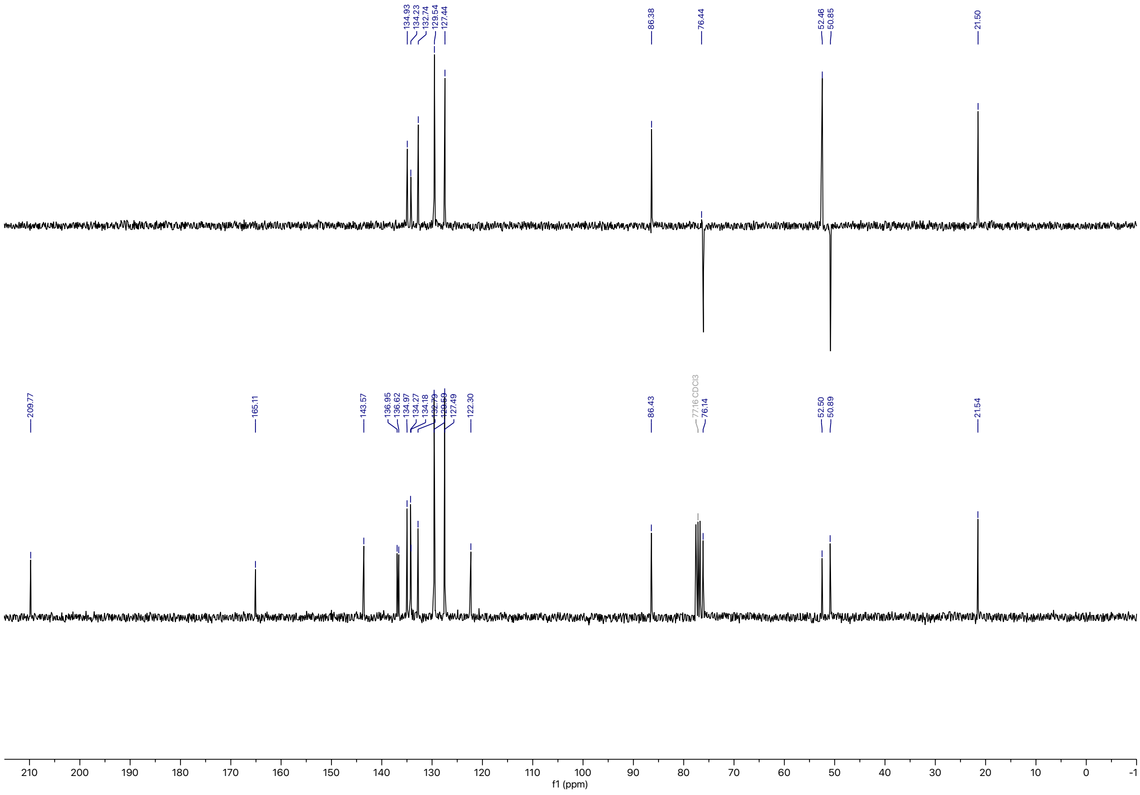

^{1}H-NMR (500 MHz). Solvent $CDCl_3$

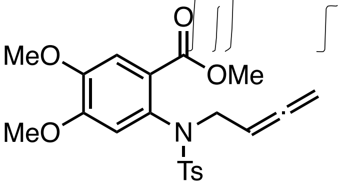

S11n

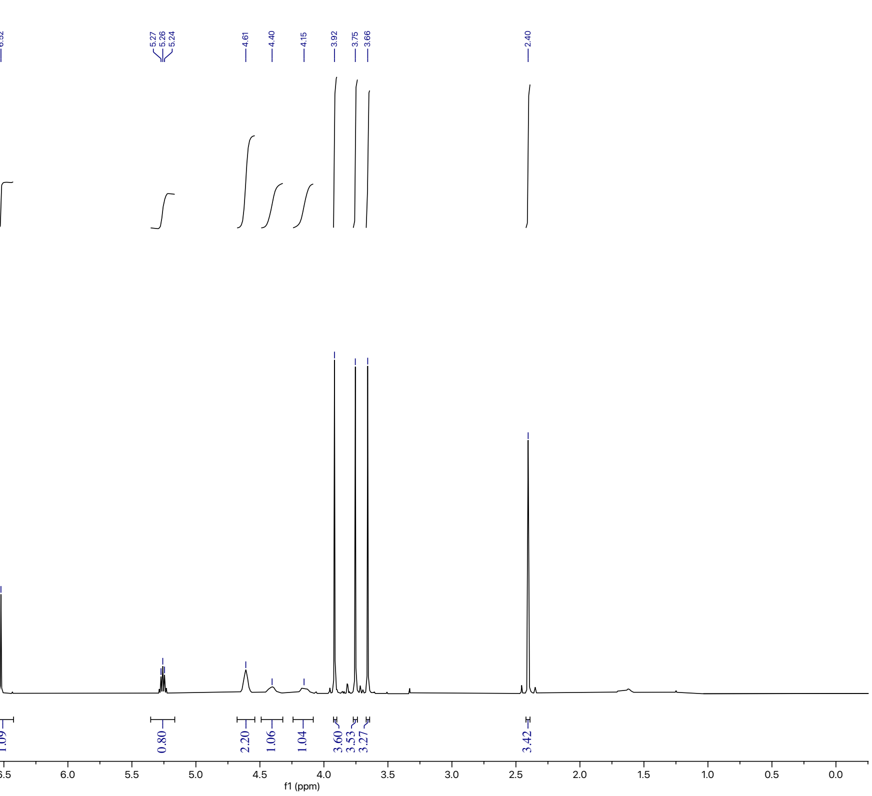

^{13}C-NMR (126 MHz). Solvent $CDCl_3$

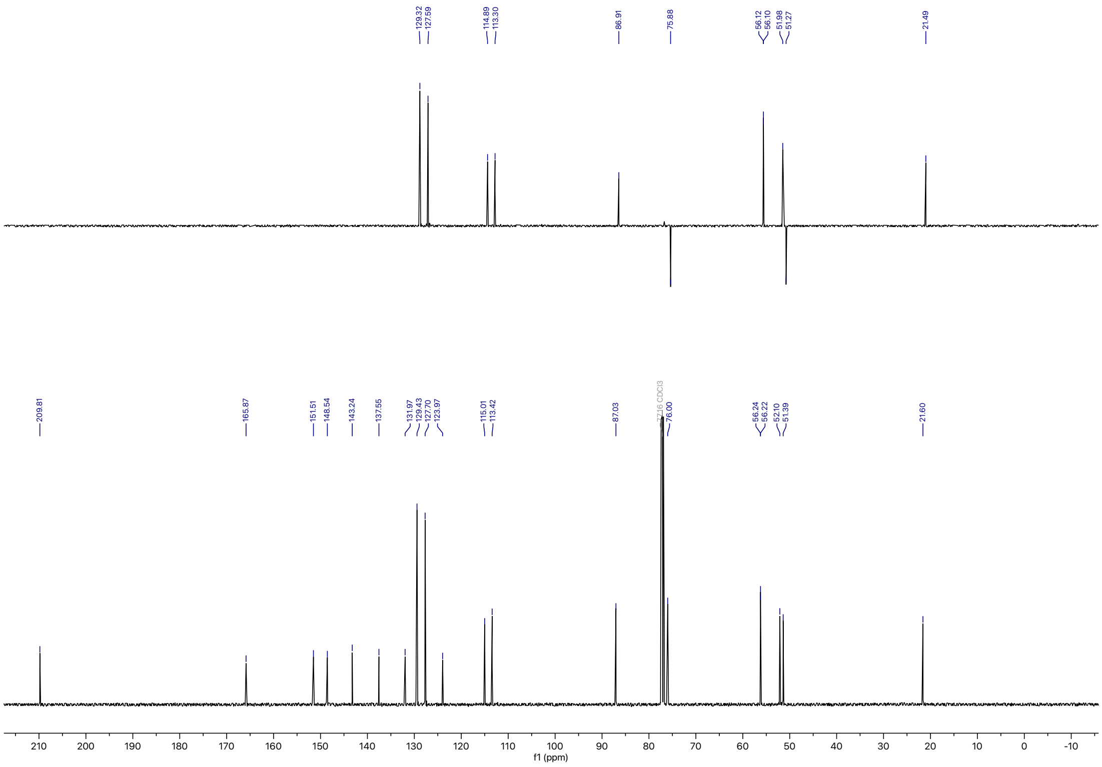

^{1}H-NMR (300 MHz). Solvent $CDCl_3$

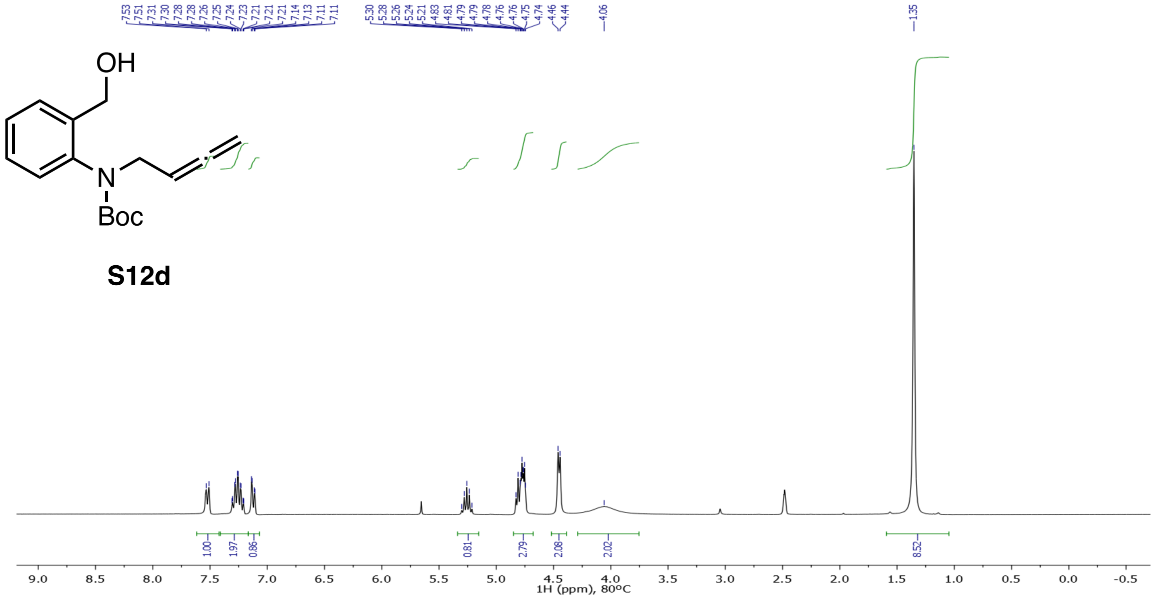

^{13}C-NMR (75 MHz). Solvent $CDCl_3$

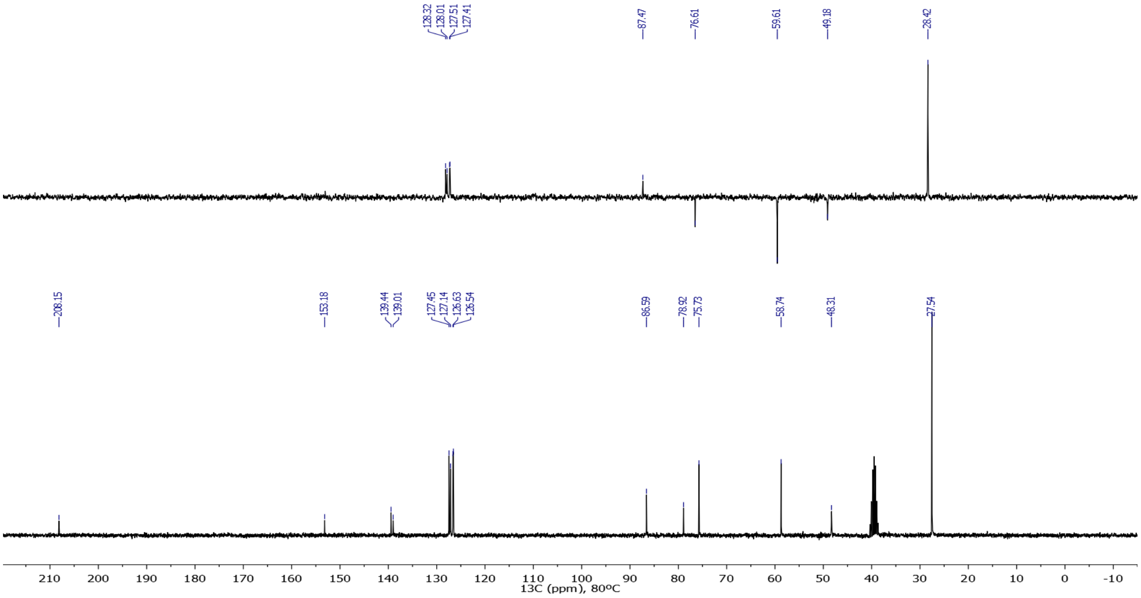

^{1}H-NMR (300 MHz). Solvent $CDCl_3$

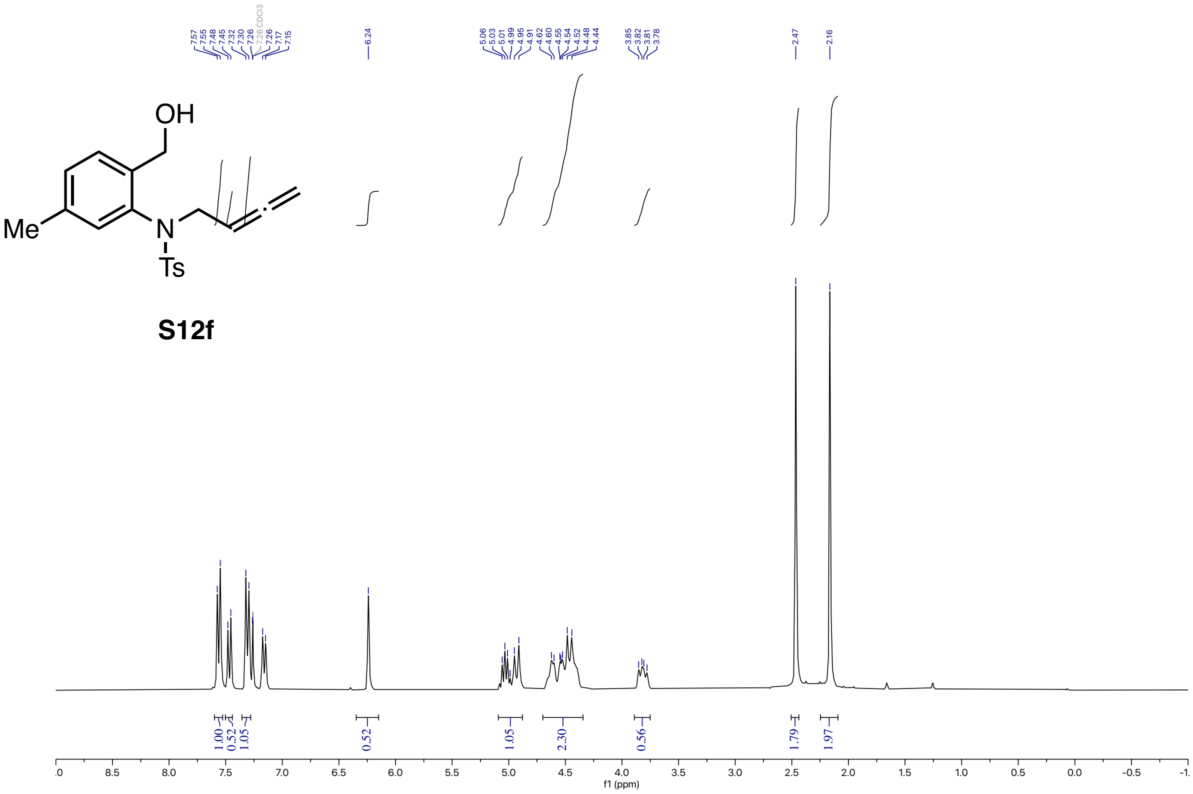

S12f

^{13}C-NMR (75 MHz). Solvent $CDCl_3$

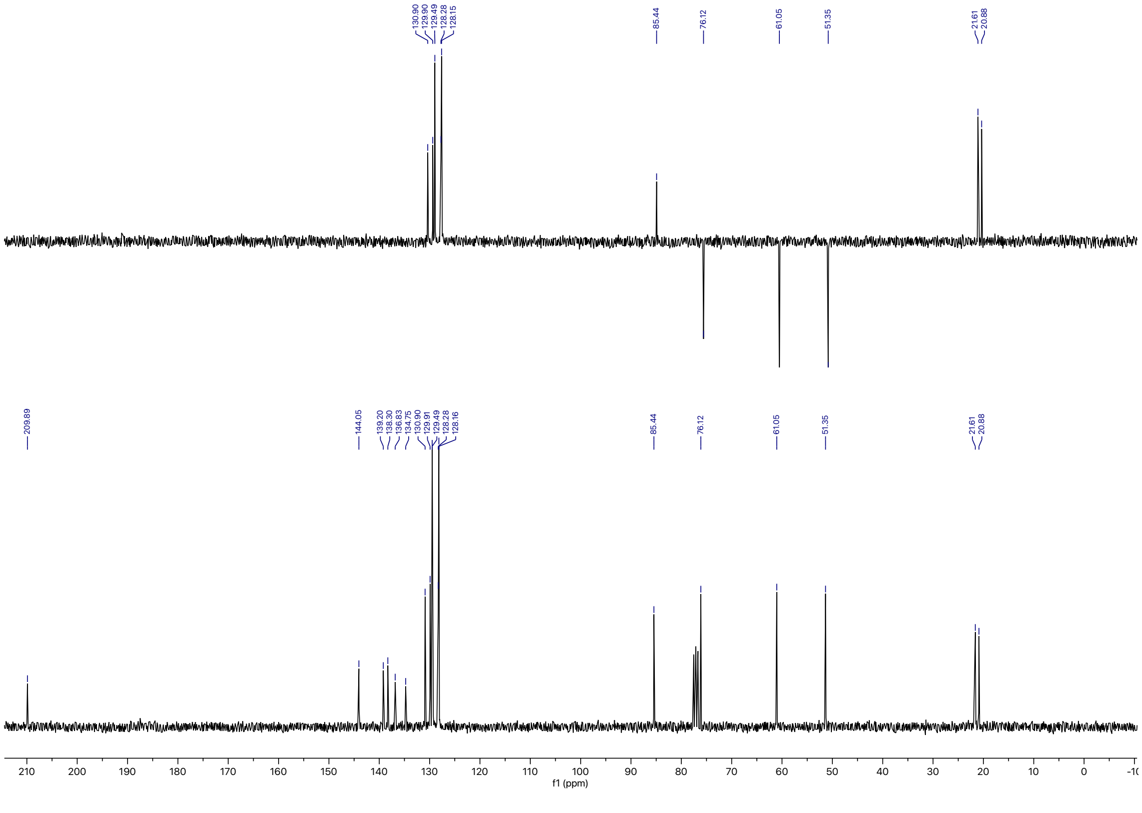

^{1}H-NMR (500 MHz). Solvent $CDCl_3$

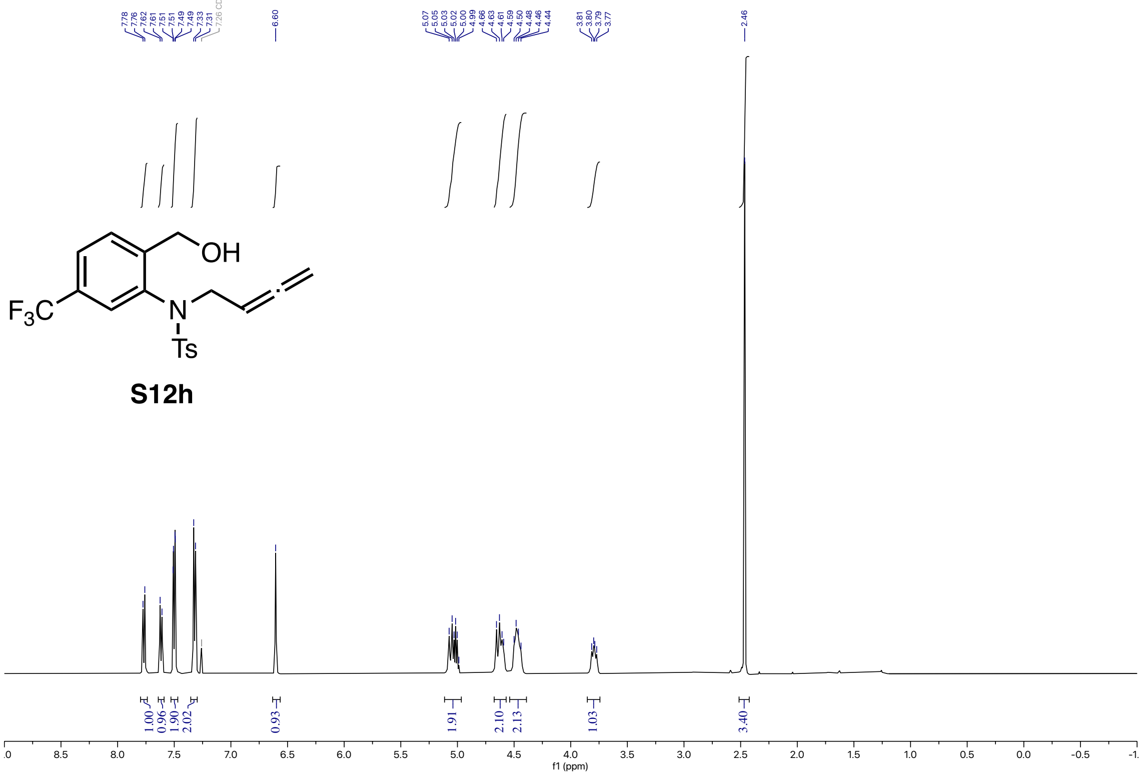

S12h

^{13}C-NMR (126 MHz). Solvent $CDCl_3$

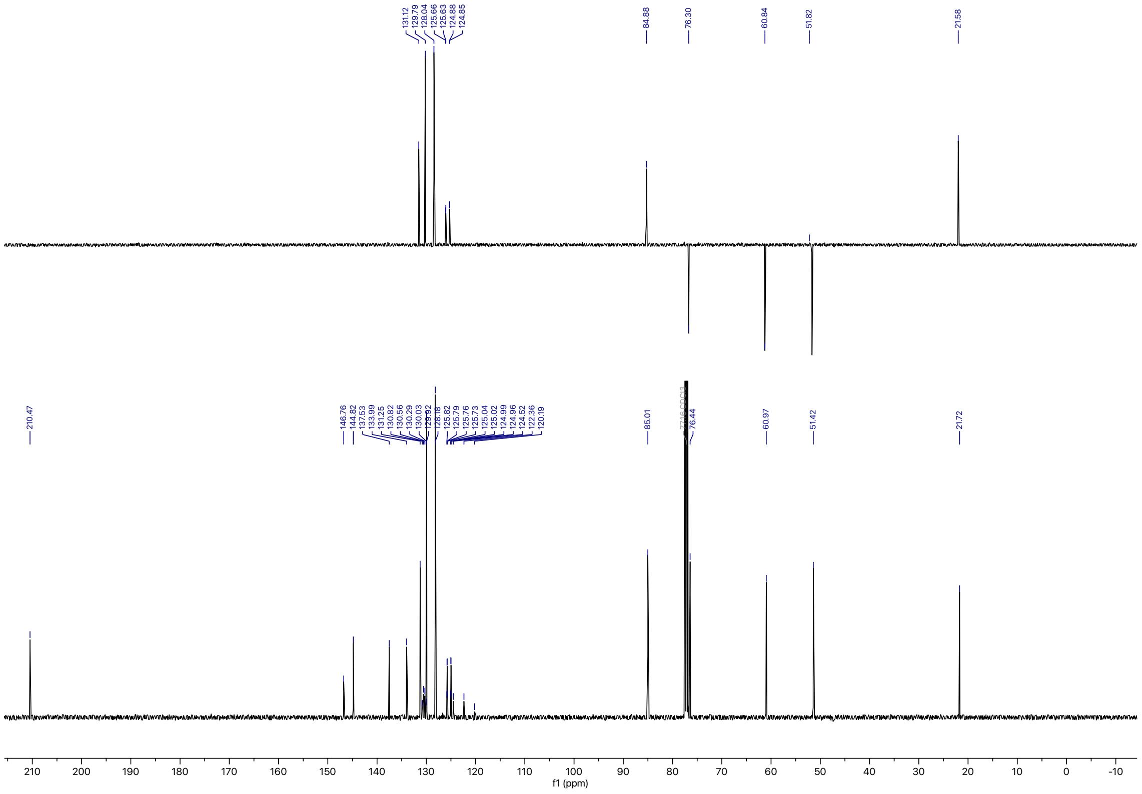

^{19}F-NMR (282 MHz). Solvent $CDCl_3$

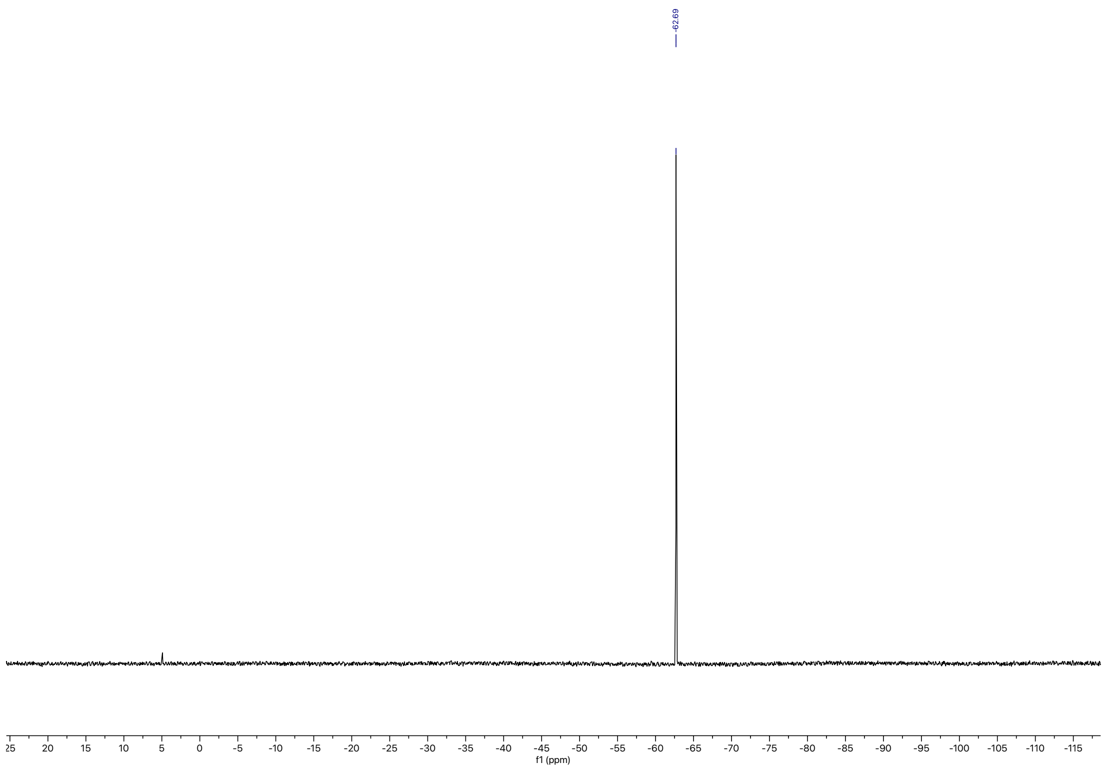

^{1}H-NMR (500 MHz). Solvent $CDCl_3$

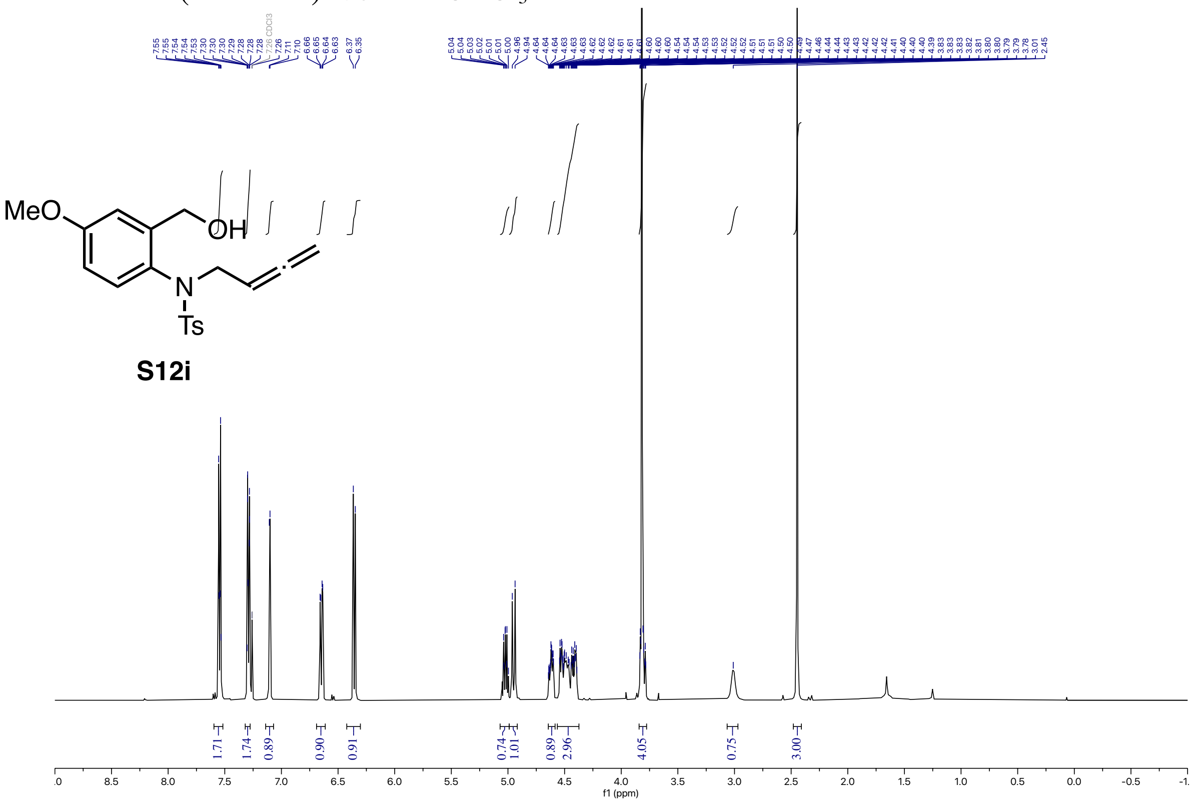

S12i

^{13}C-NMR (126 MHz). Solvent $CDCl_3$

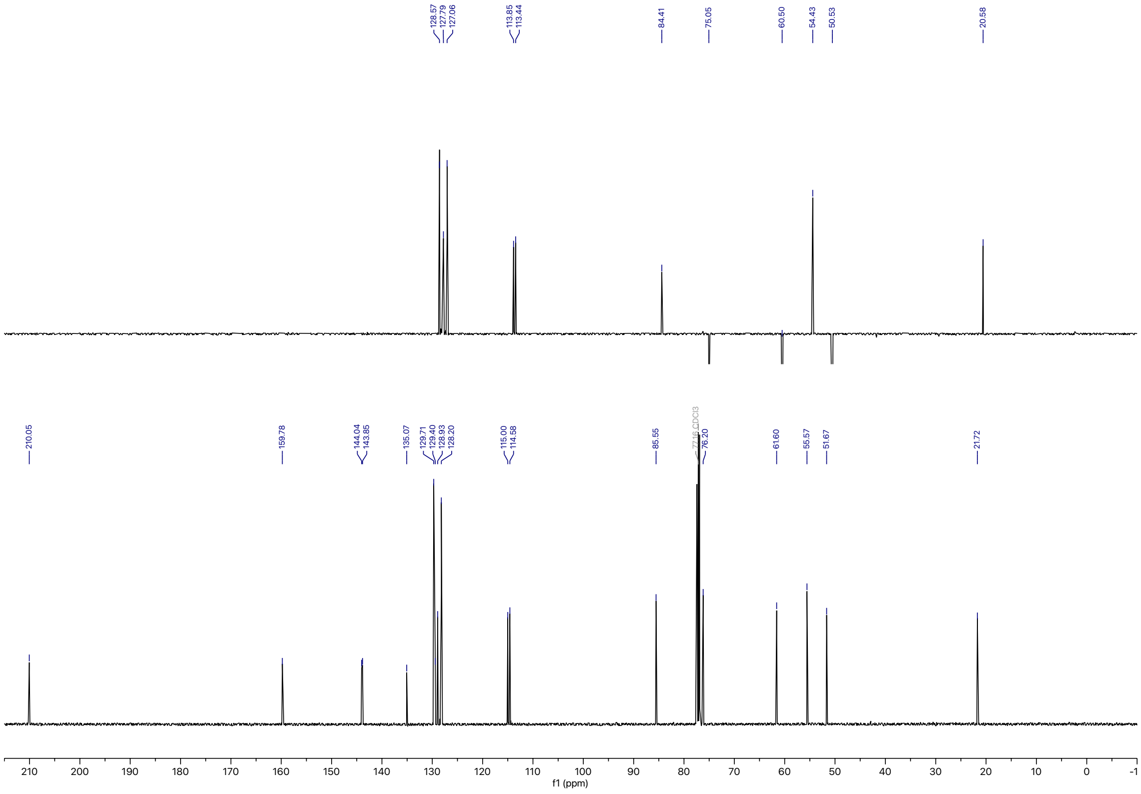

^{1}H-NMR (500 MHz). Solvent $CDCl_3$

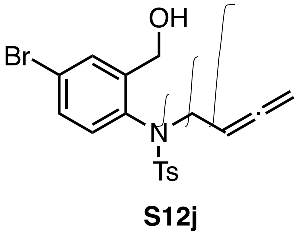

S12j

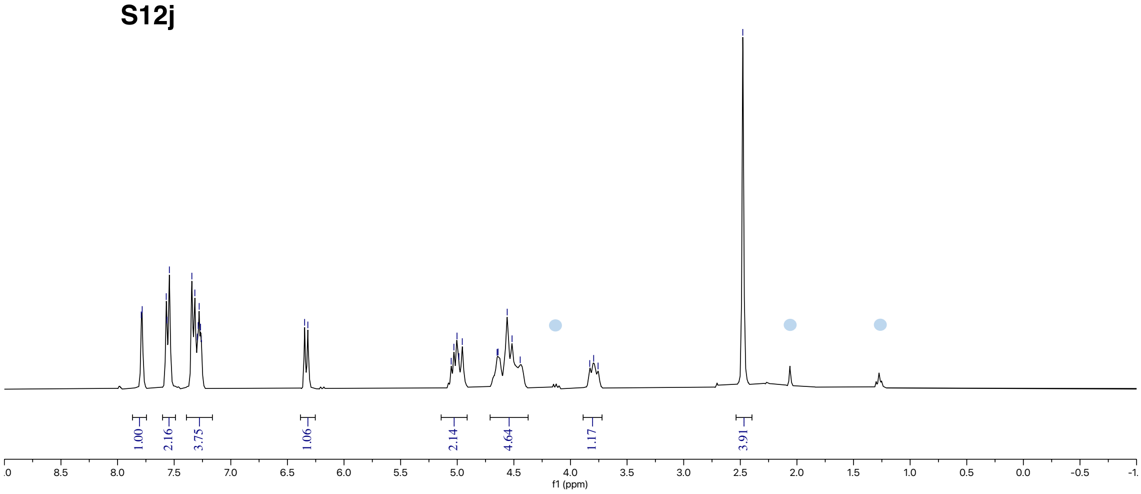

^{13}C-NMR (126 MHz). Solvent $CDCl_3$

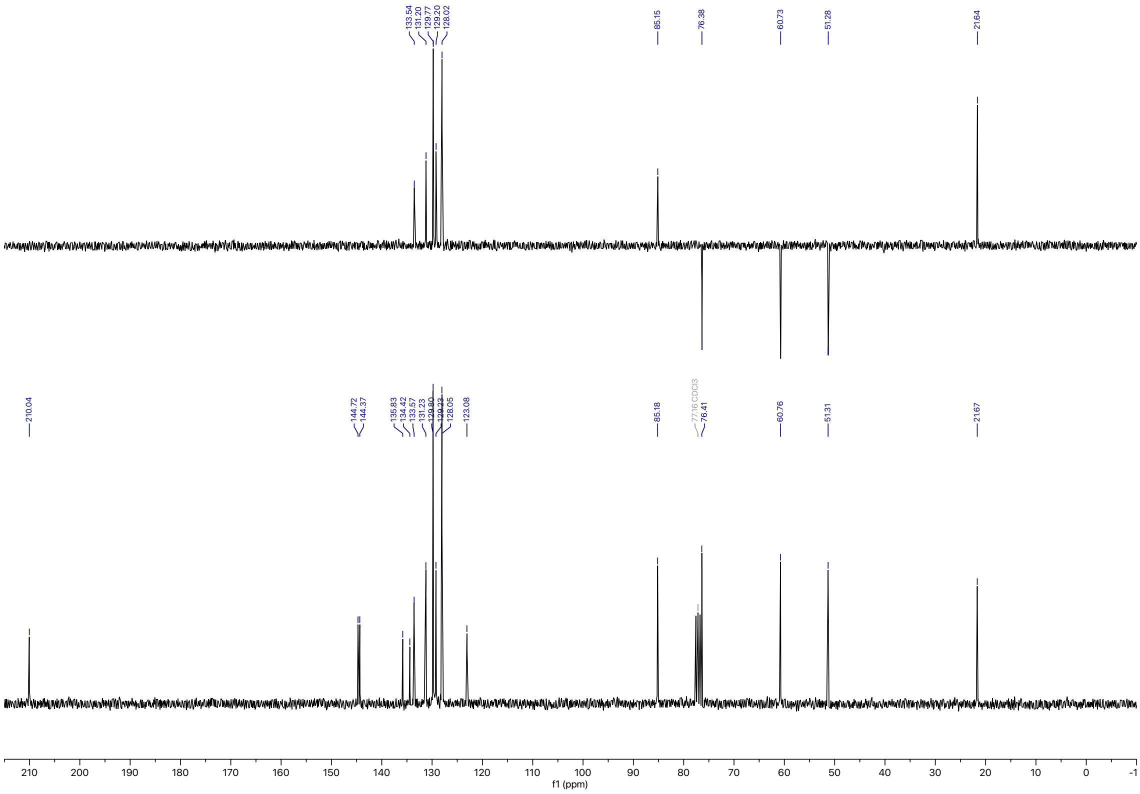

^{1}H-NMR (500 MHz). Solvent $CDCl_3$

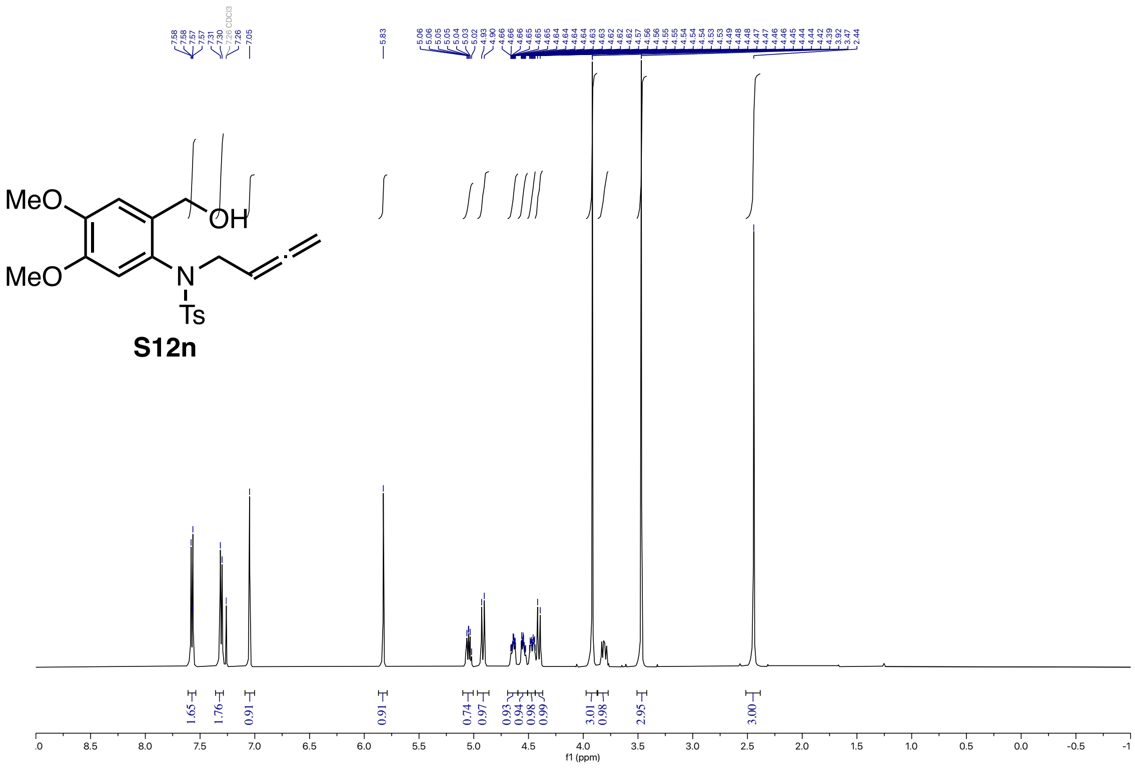

^{13}C-NMR (126 MHz). Solvent $CDCl_3$

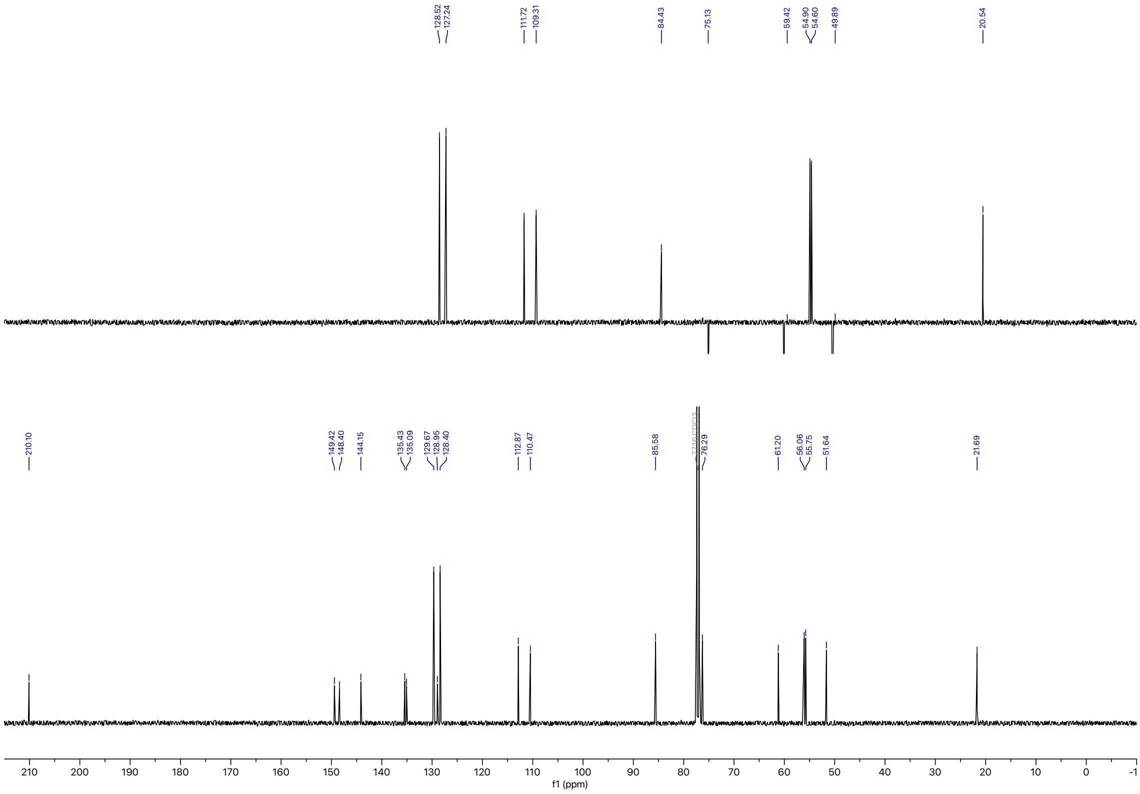

^{1}H-NMR (300 MHz). Solvent $CDCl_3$

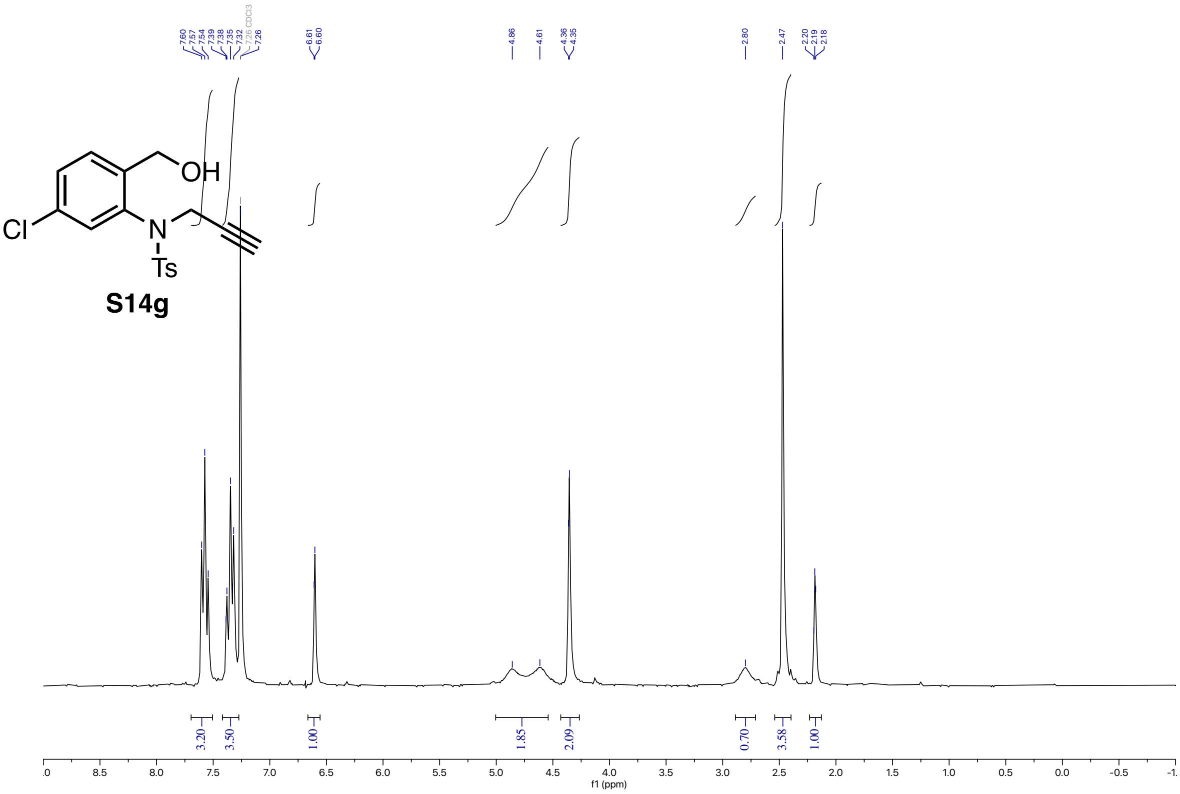

^{13}C-NMR (75 MHz). Solvent $CDCl_3$

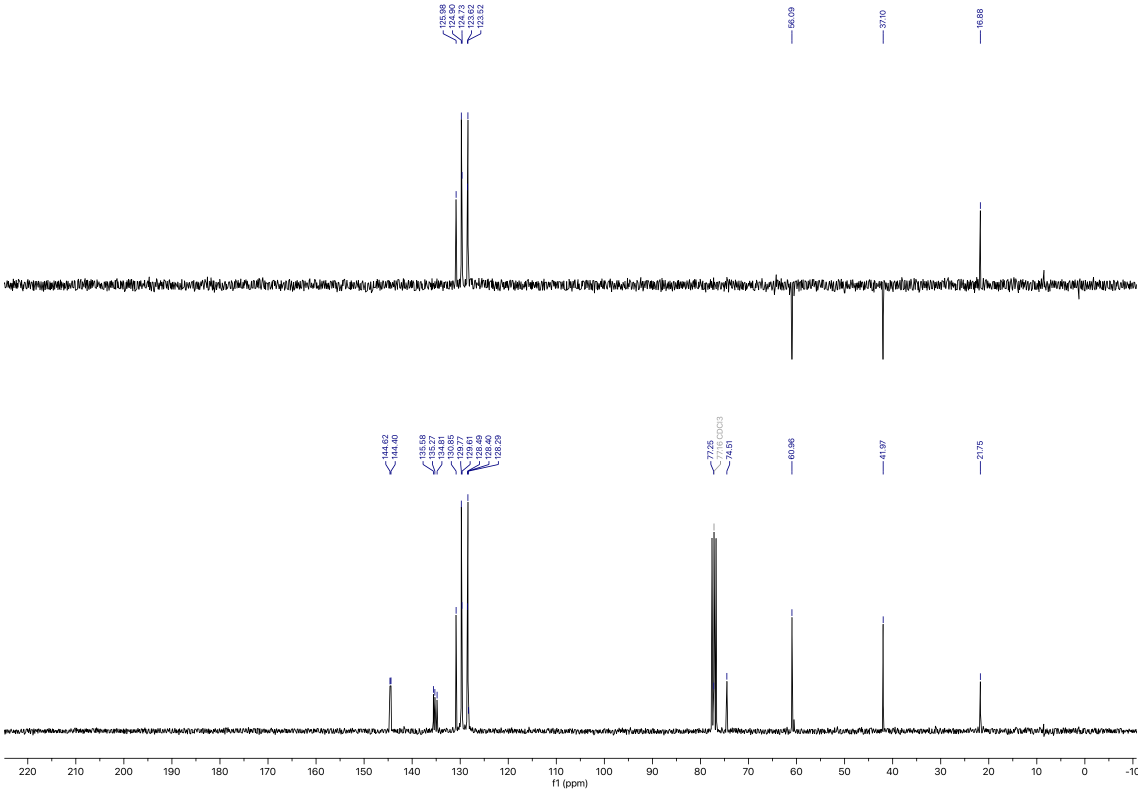

^{1}H-NMR (300 MHz). Solvent $CDCl_3$

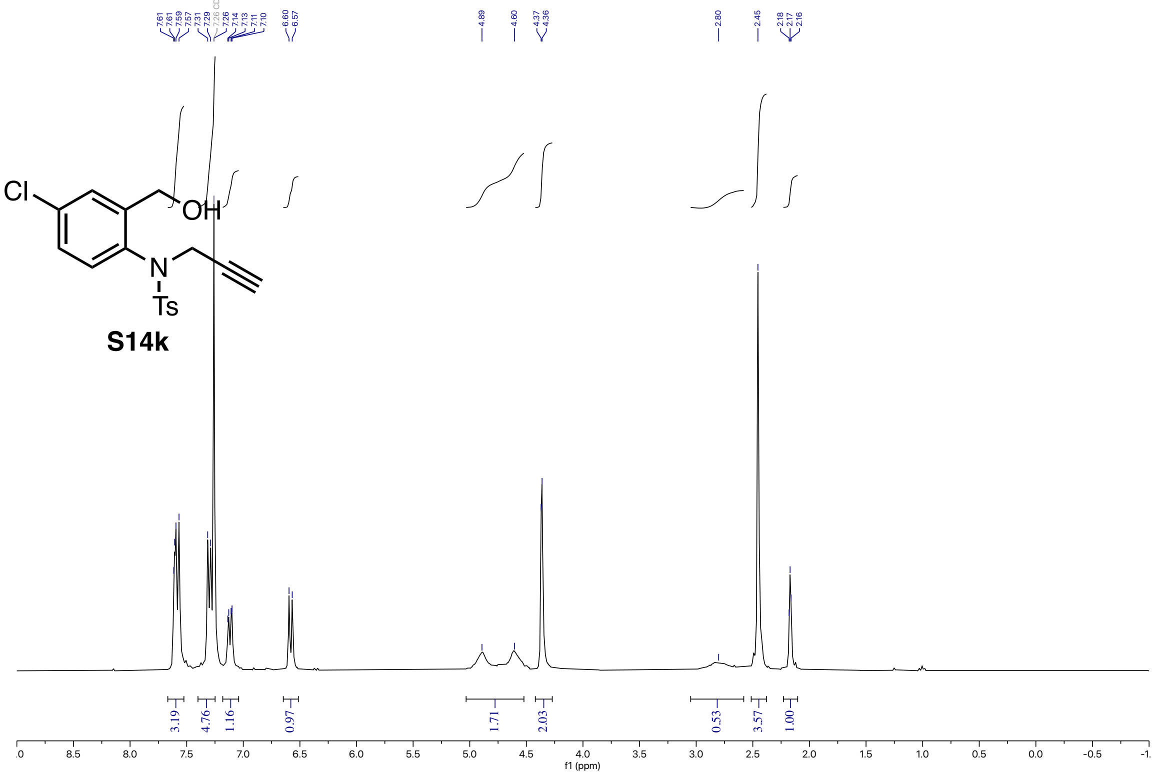

S14k

^{13}C-NMR (75 MHz). Solvent $CDCl_3$

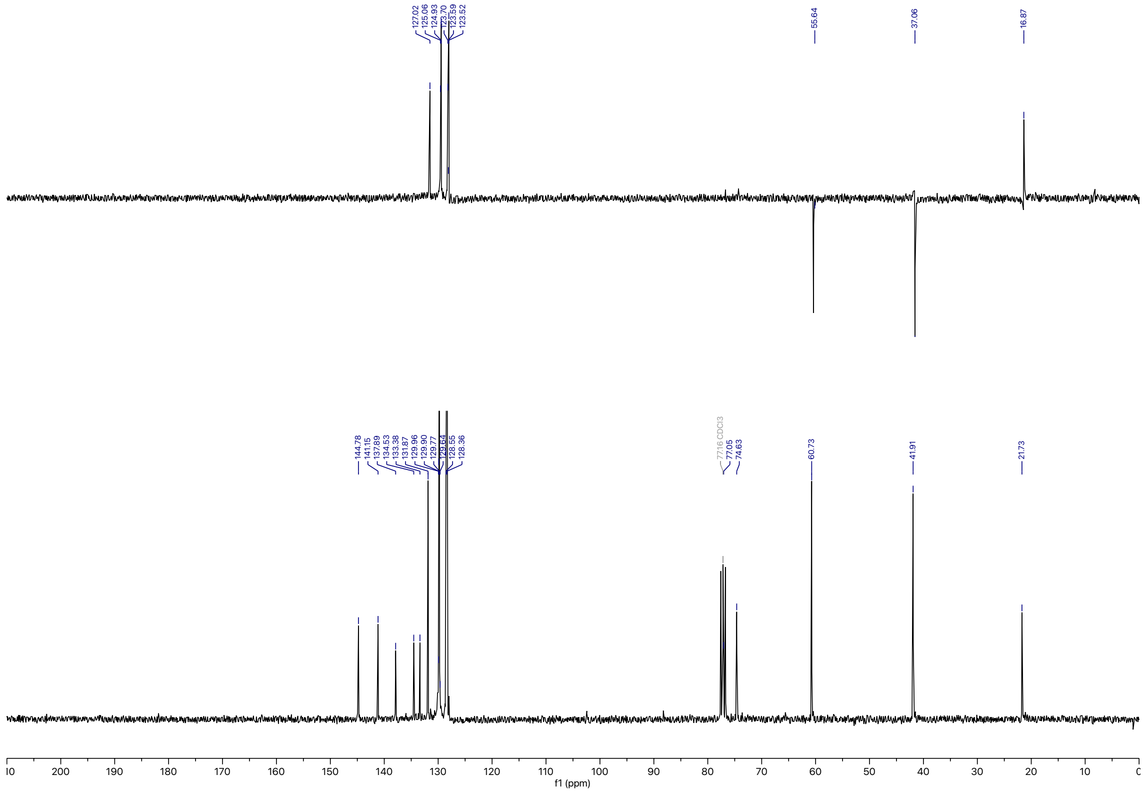

^{1}H-NMR (300 MHz). Solvent $CDCl_3$

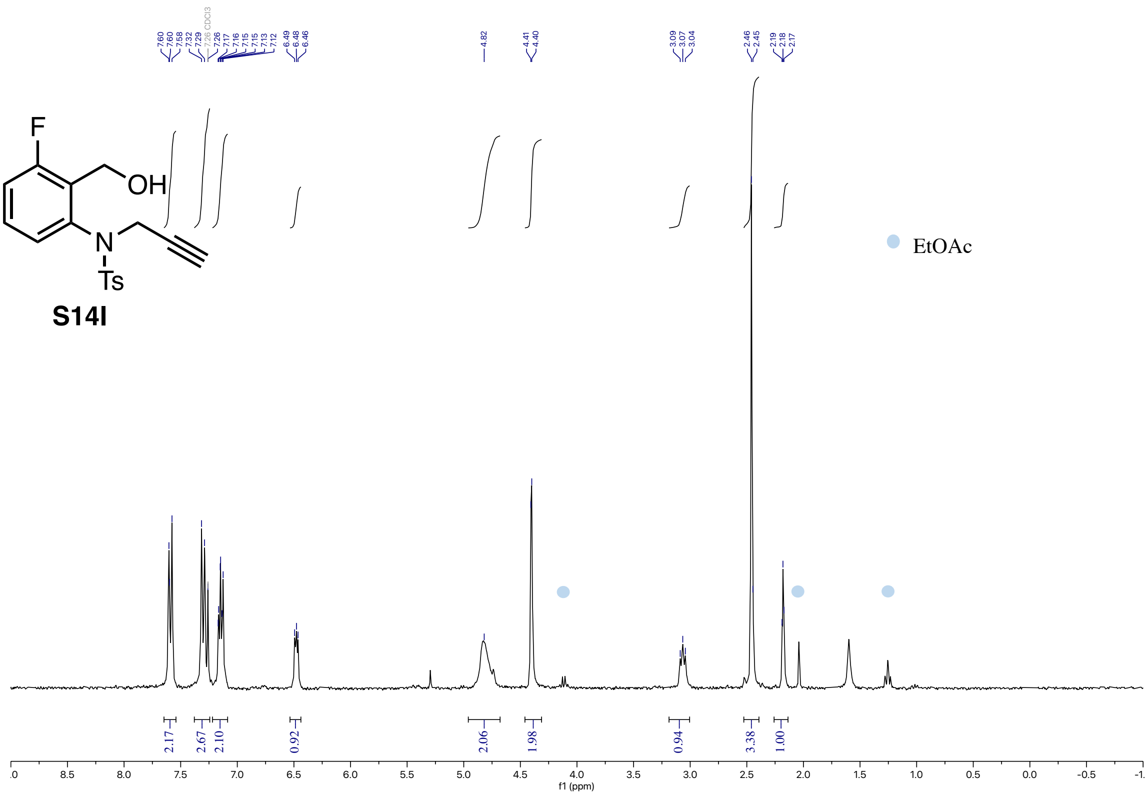

^{13}C-NMR (75 MHz). Solvent $CDCl_3$

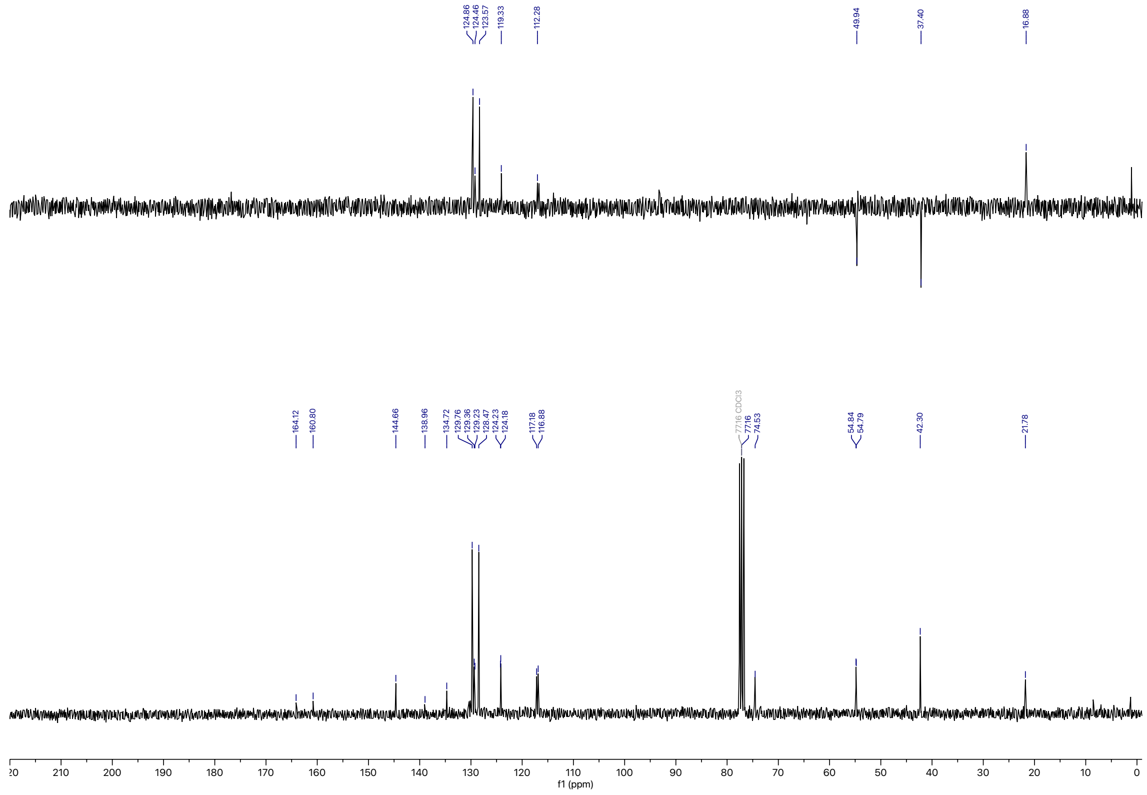

^{19}F-NMR (282 MHz). Solvent $CDCl_3$

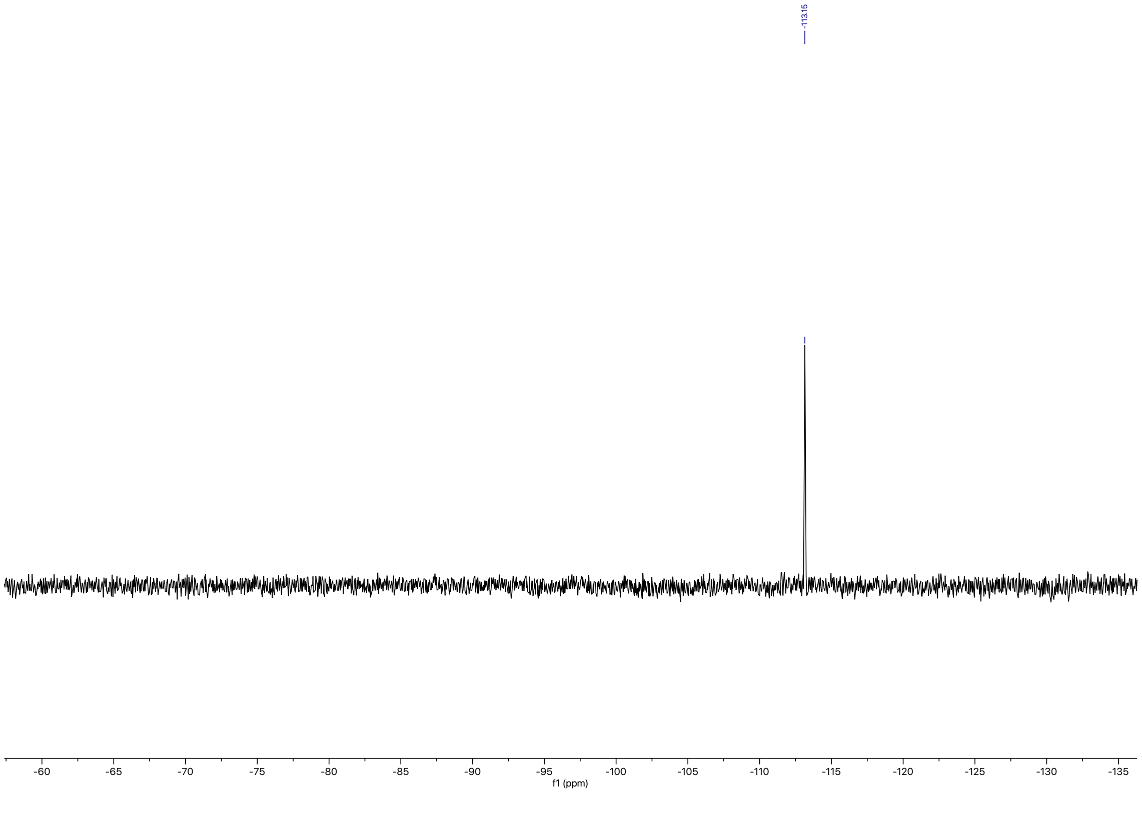

^{1}H-NMR (300 MHz). Solvent $CDCl_3$

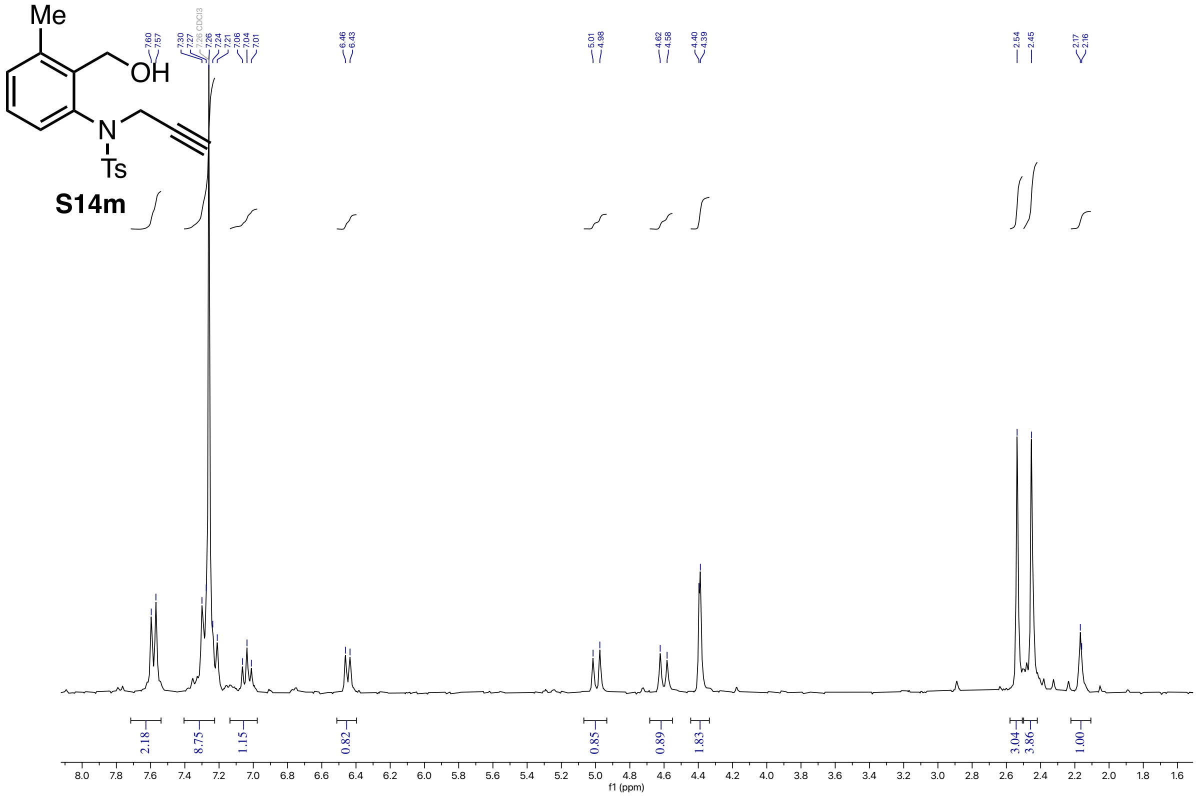

^{13}C-NMR (75 MHz). Solvent $CDCl_3$

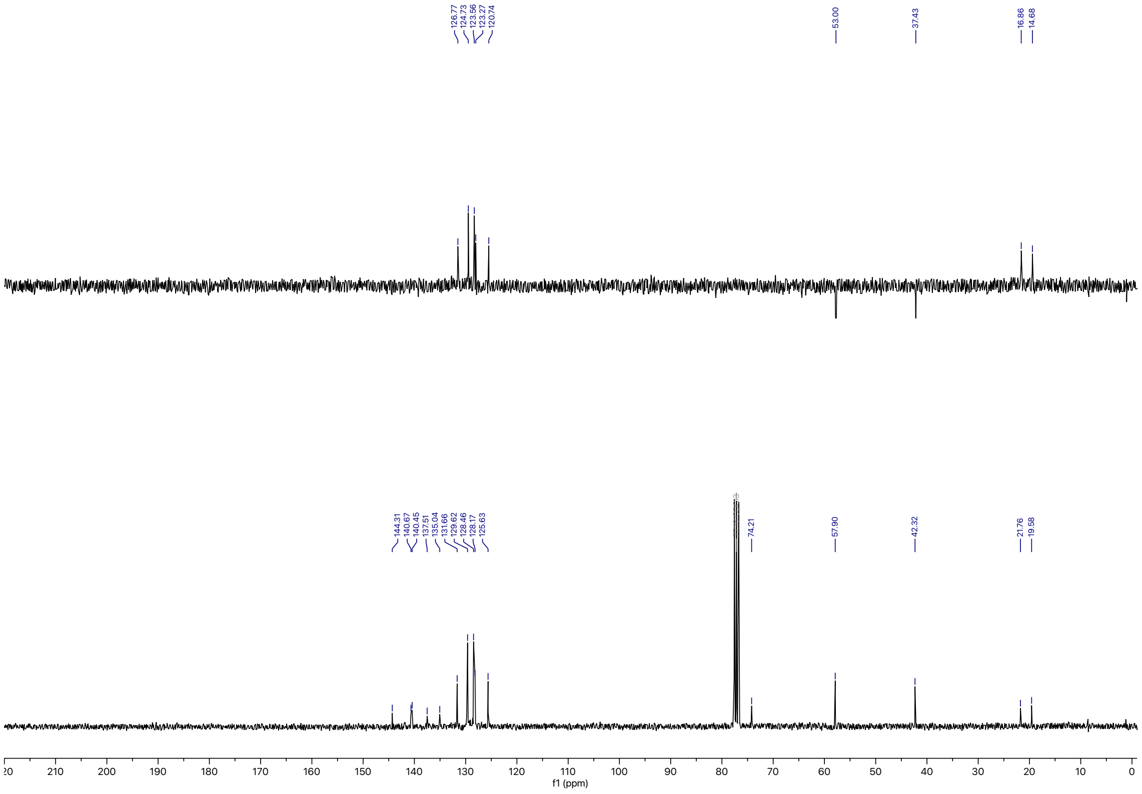

^{1}H-NMR (300 MHz). Solvent $CDCl_3$

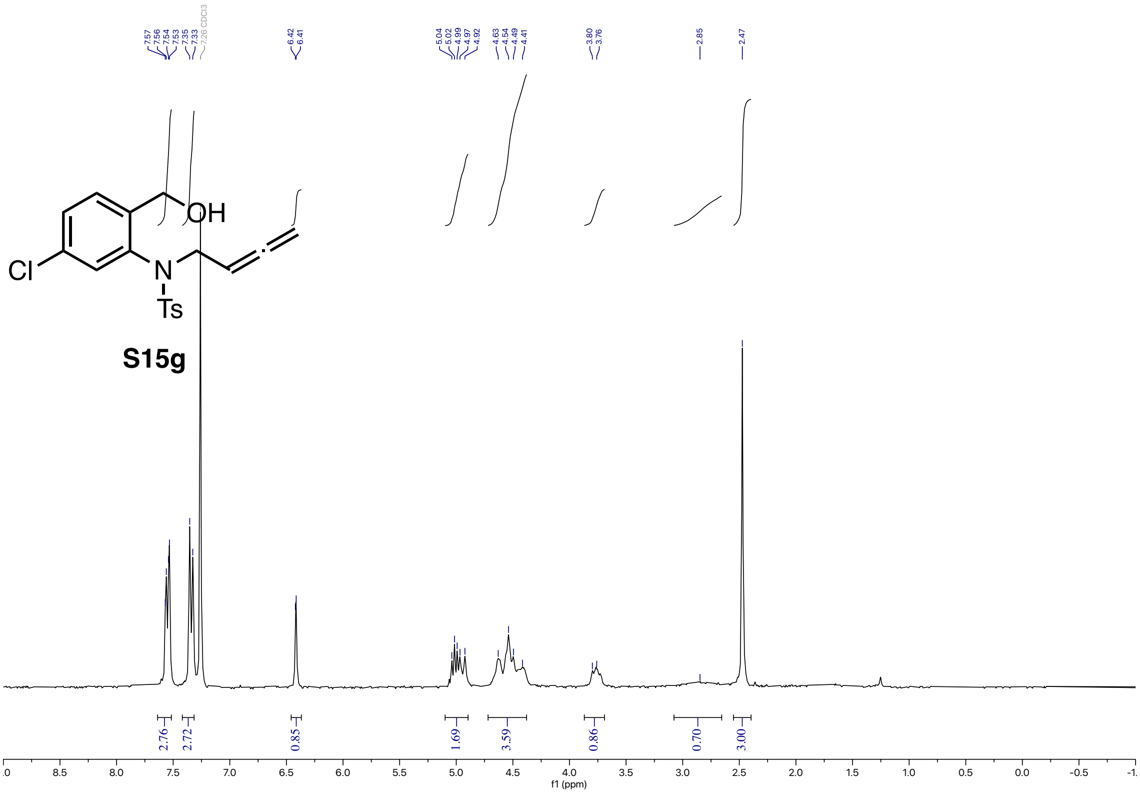

S15g

^{13}C-NMR (75 MHz). Solvent $CDCl_3$

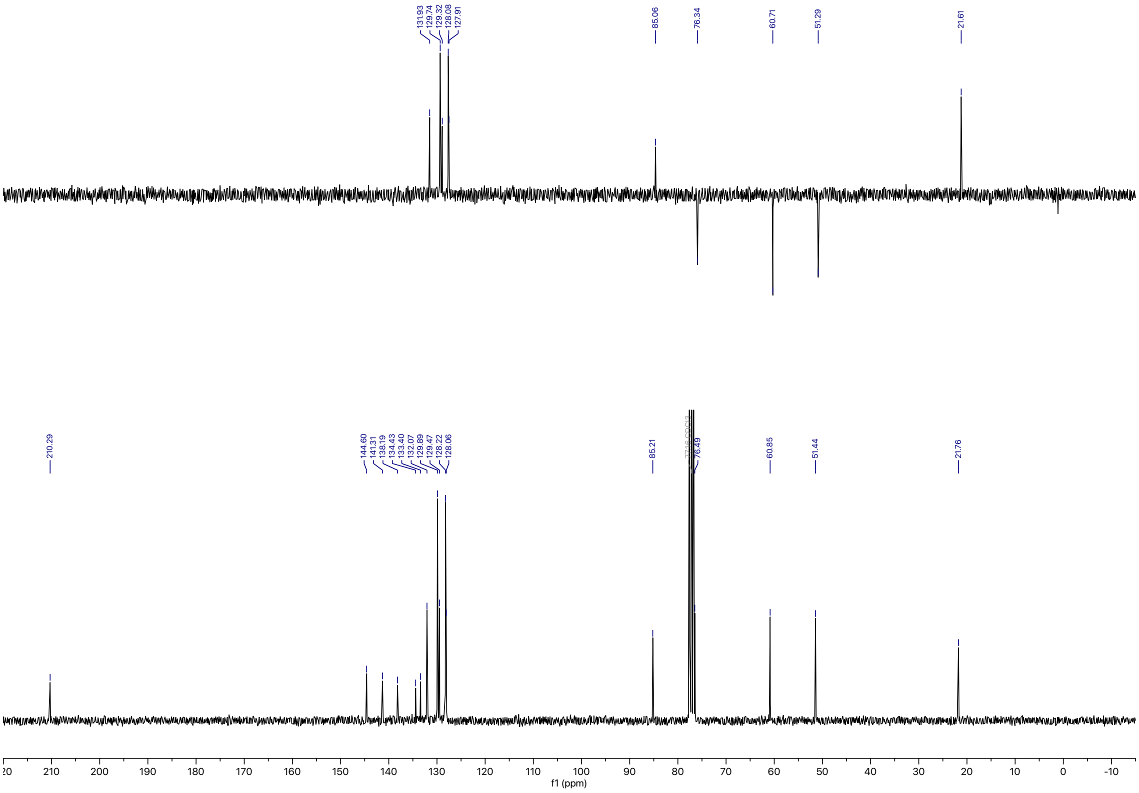

^{1}H-NMR (300 MHz). Solvent $CDCl_3$

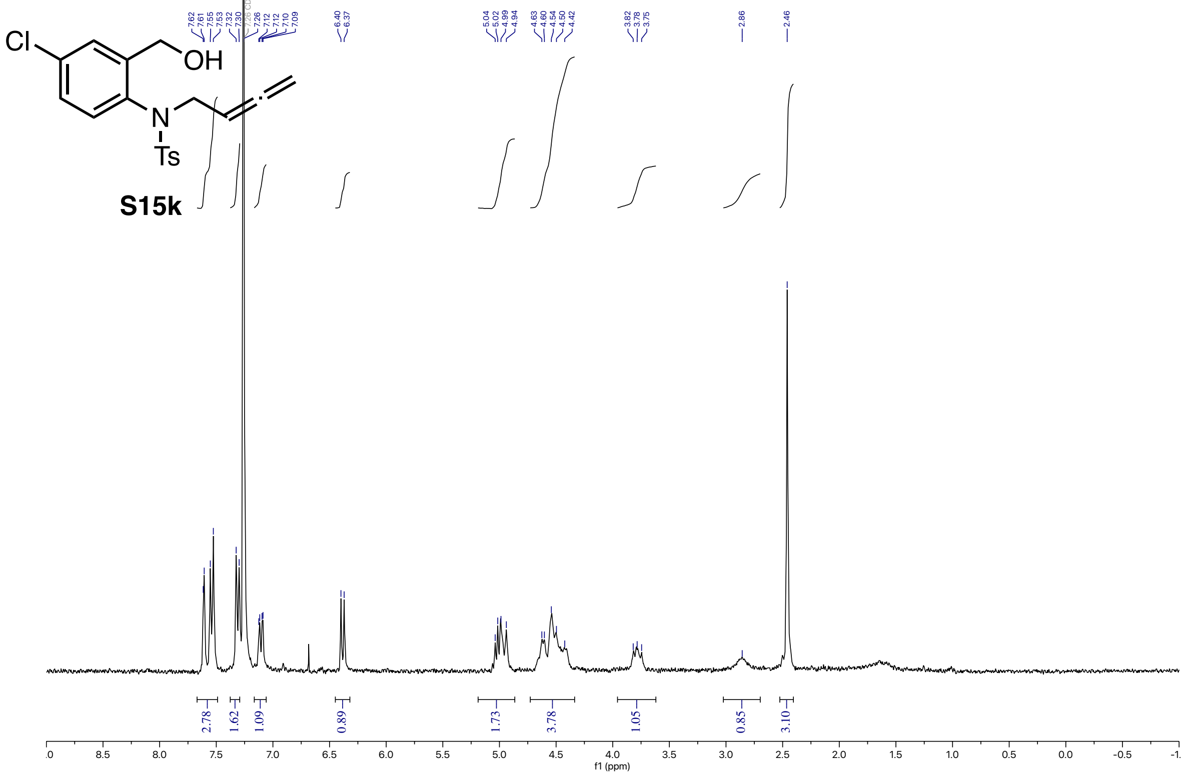

S15k

^{13}C-NMR (75 MHz). Solvent $CDCl_3$

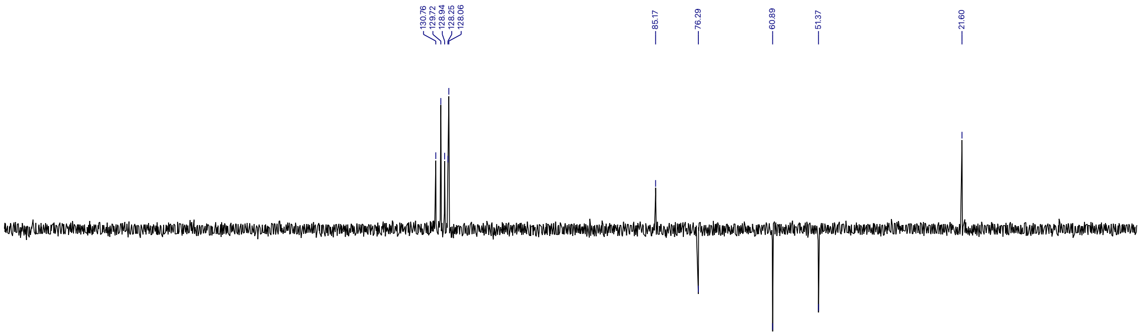

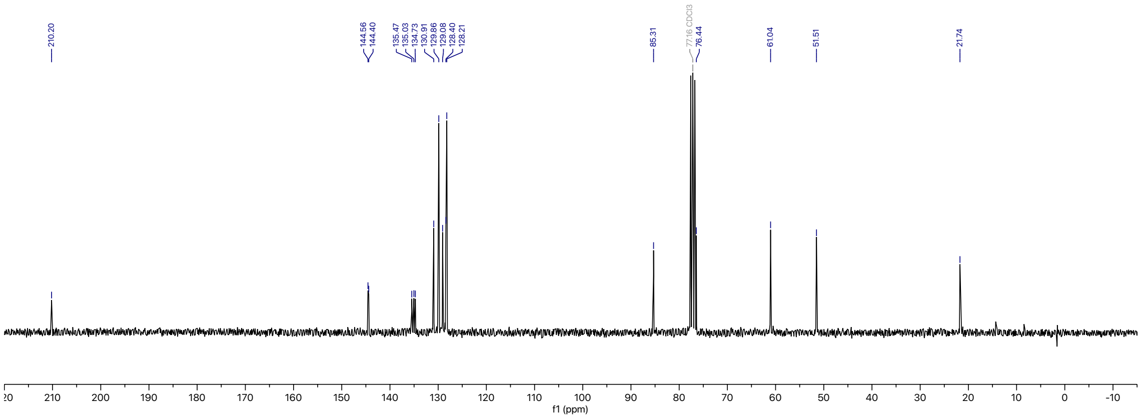

^{1}H-NMR (300 MHz). Solvent $CDCl_3$

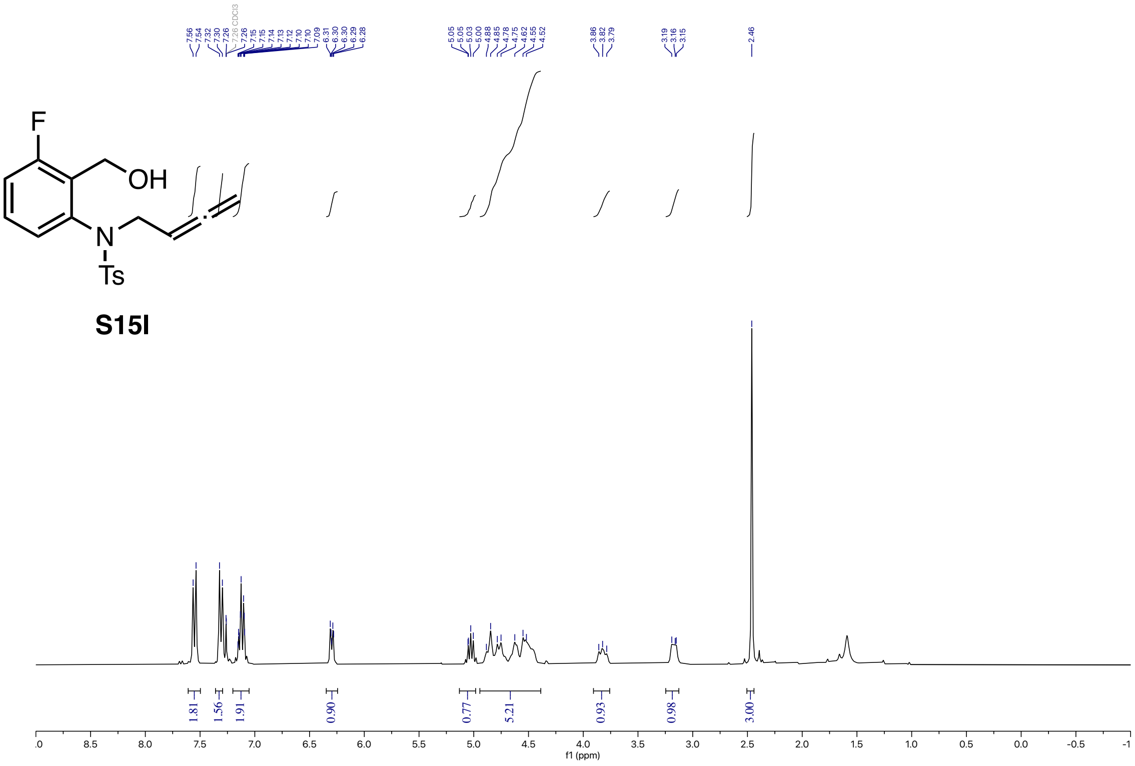

S15l

^{13}C-NMR (75MHz). Solvent $CDCl_3$

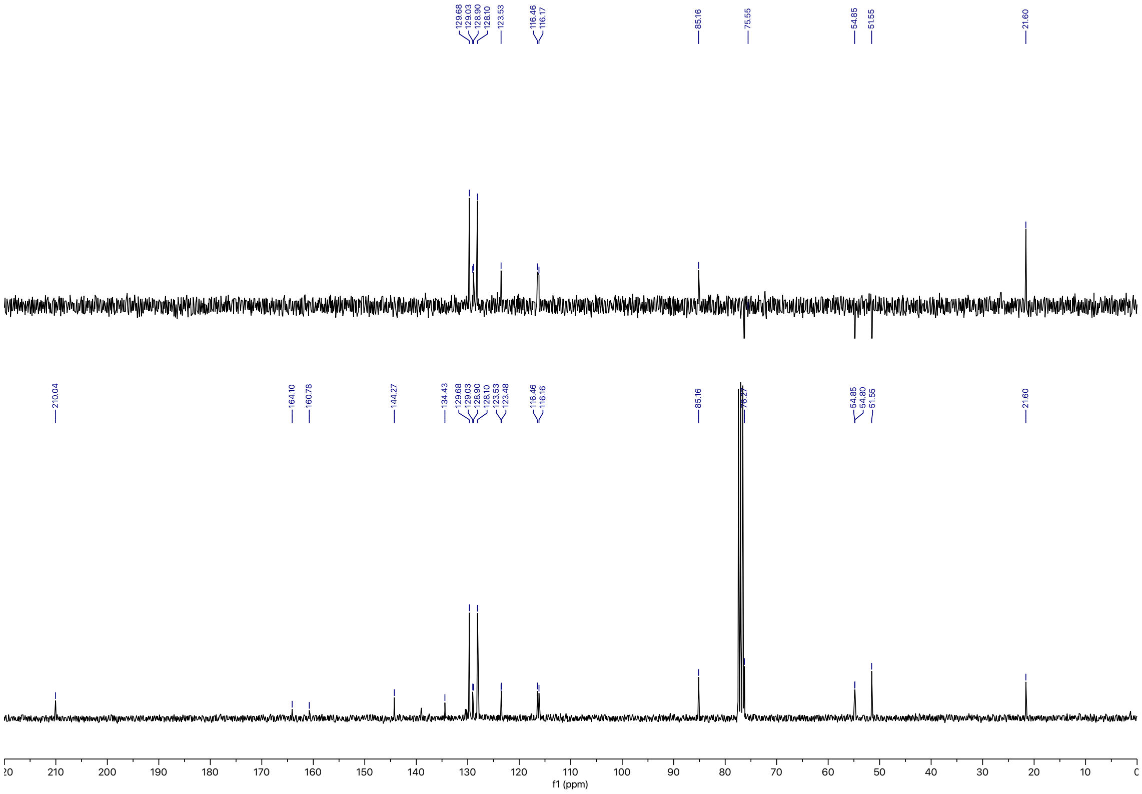

^{19}F-NMR (282 MHz). Solvent $CDCl_3$

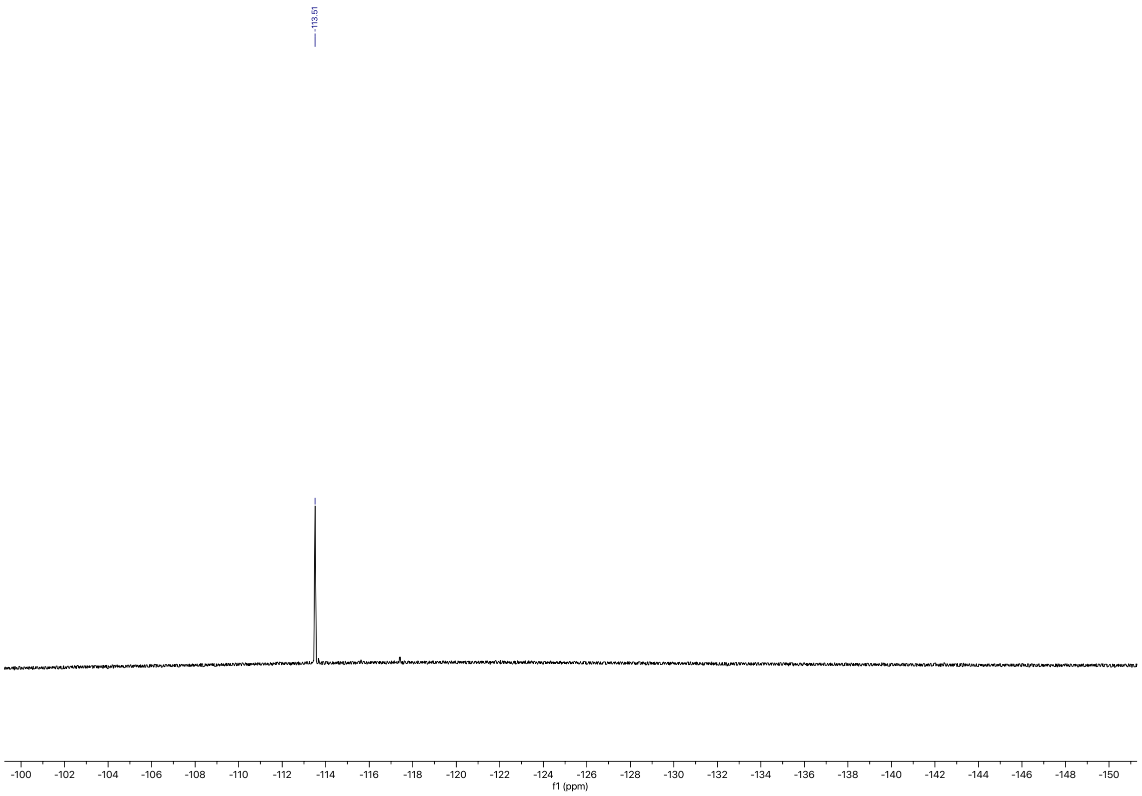

^{1}H-NMR (300 MHz). Solvent $CDCl_3$

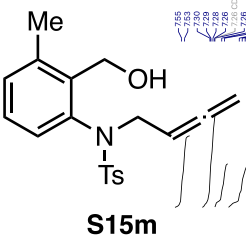

S15m

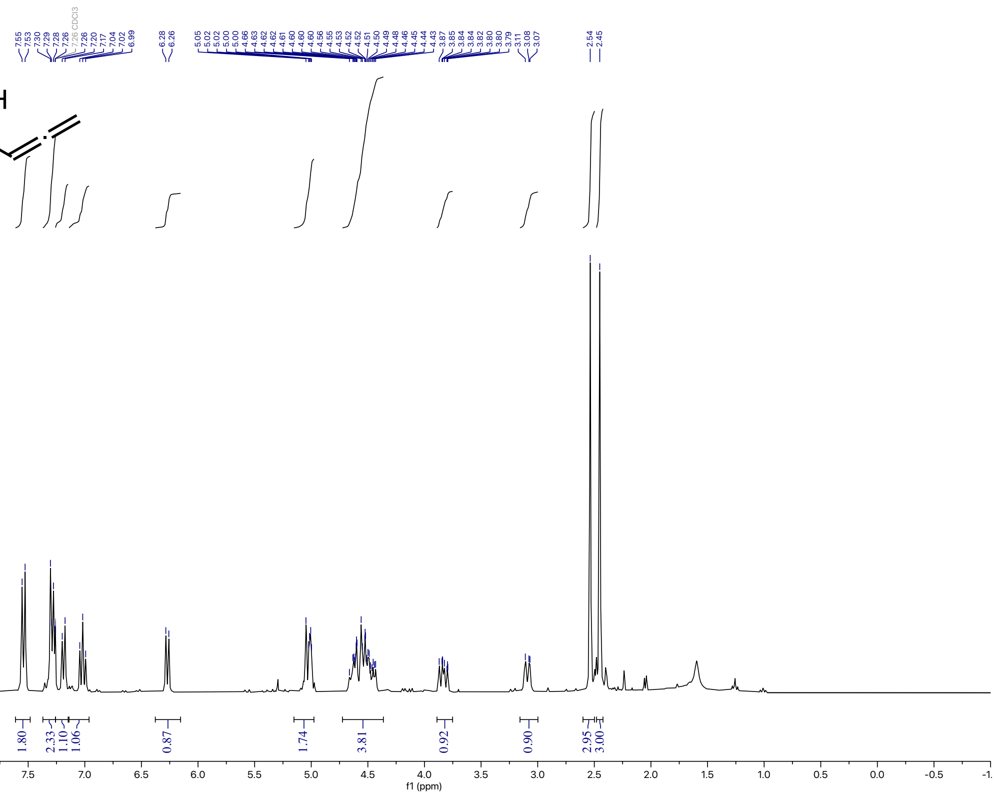

^{13}C-NMR (75 MHz). Solvent $CDCl_3$

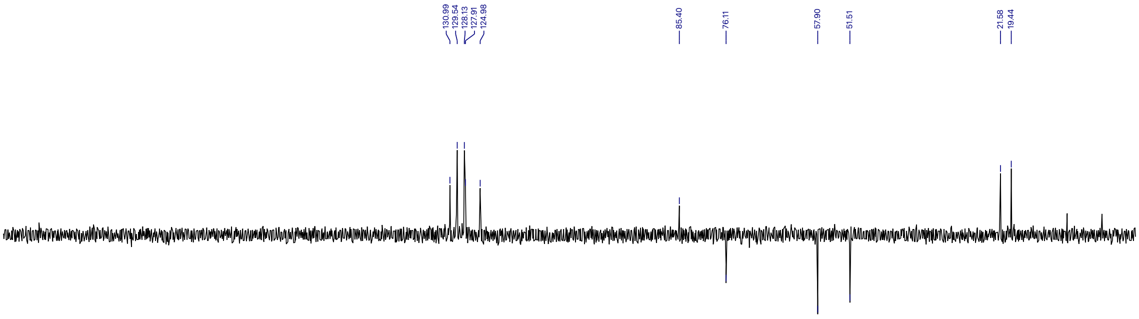

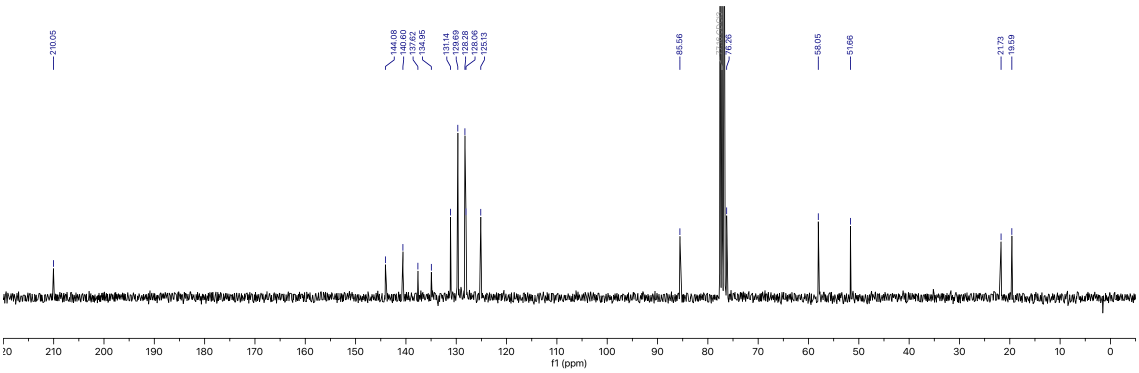

^{1}H-NMR (300 MHz). Solvent $CDCl_3$

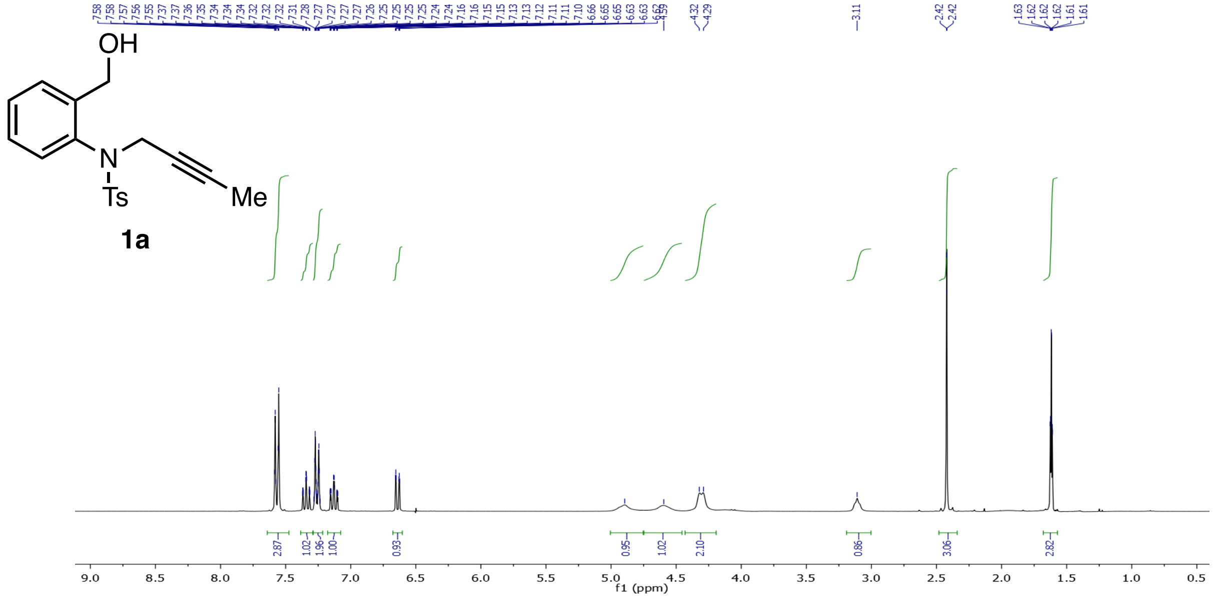

^{13}C-NMR (75 MHz). Solvent $CDCl_3$

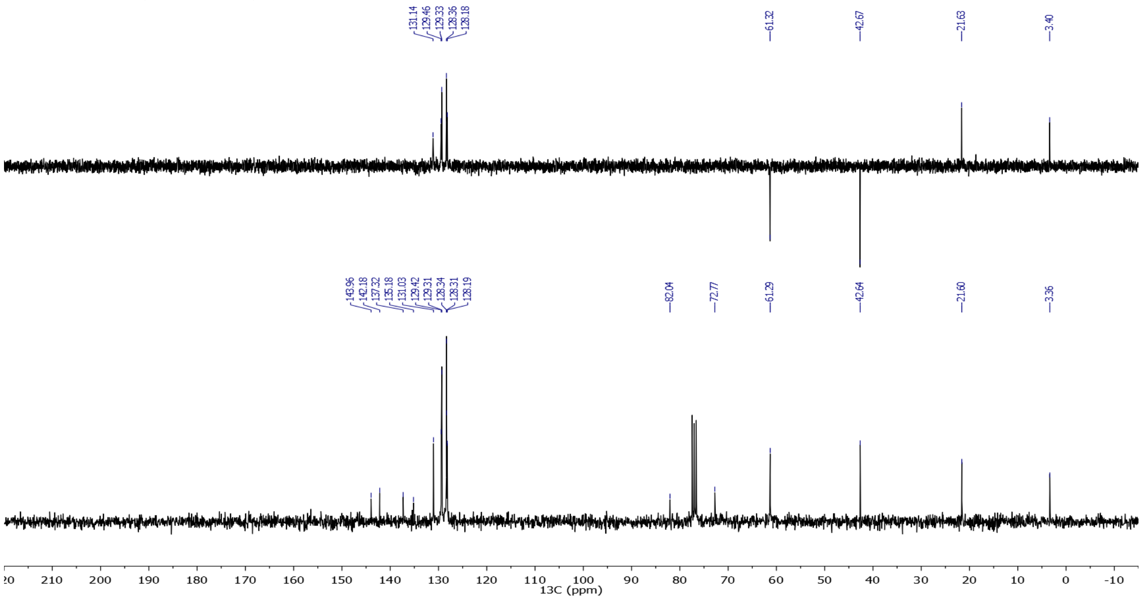

^{1}H-NMR (300 MHz). Solvent $CDCl_3$

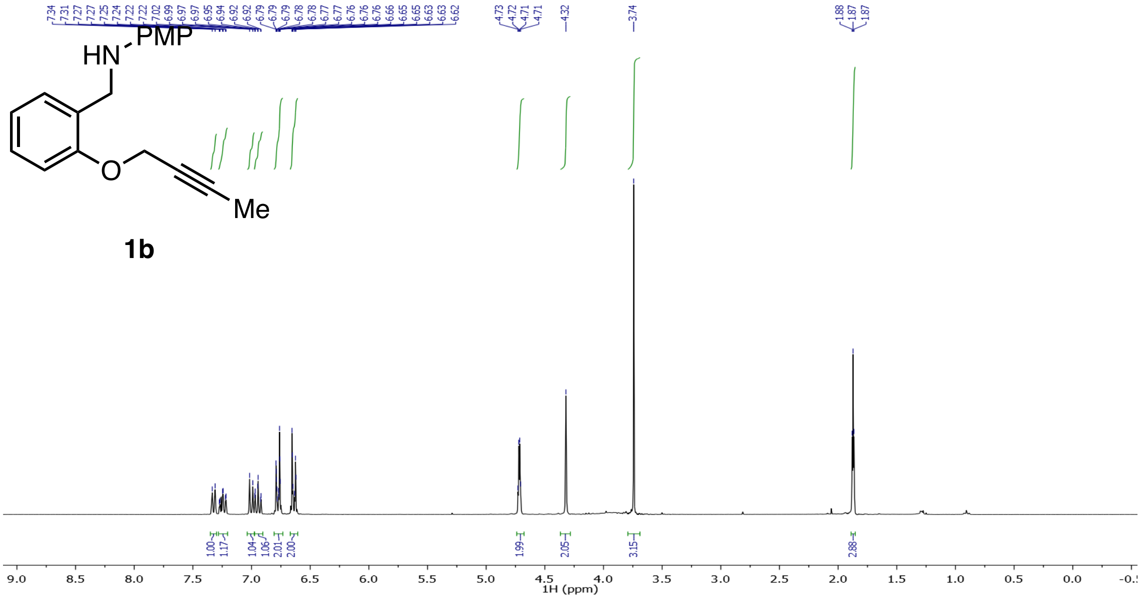

^{13}C-NMR(75 MHz). Solvent $CDCl_3$

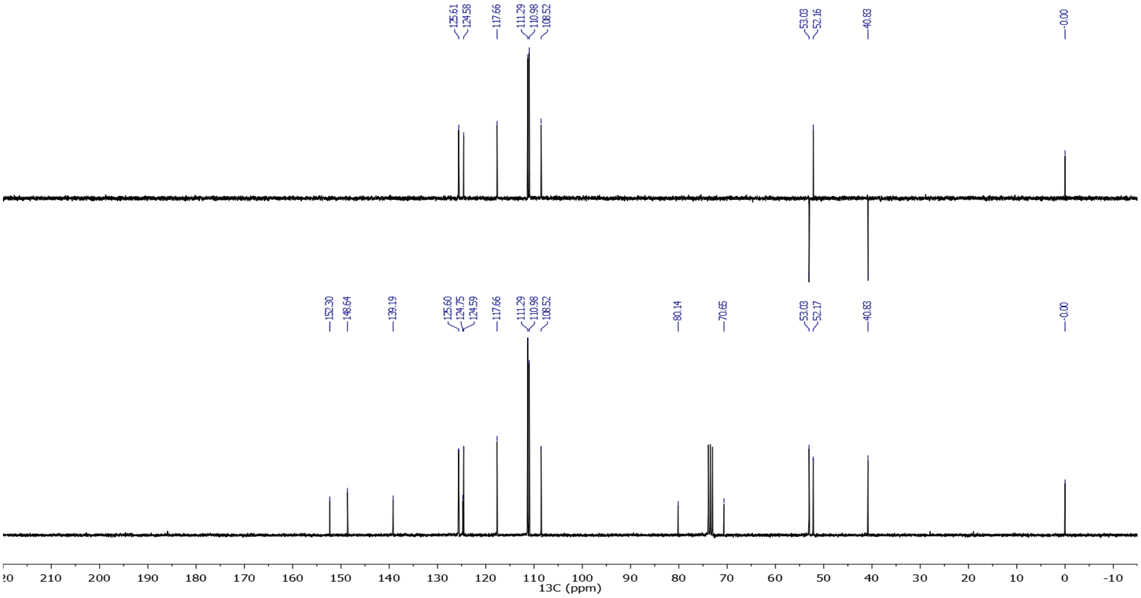

^{1}H-NMR (300 MHz). Solvent $CDCl_3$

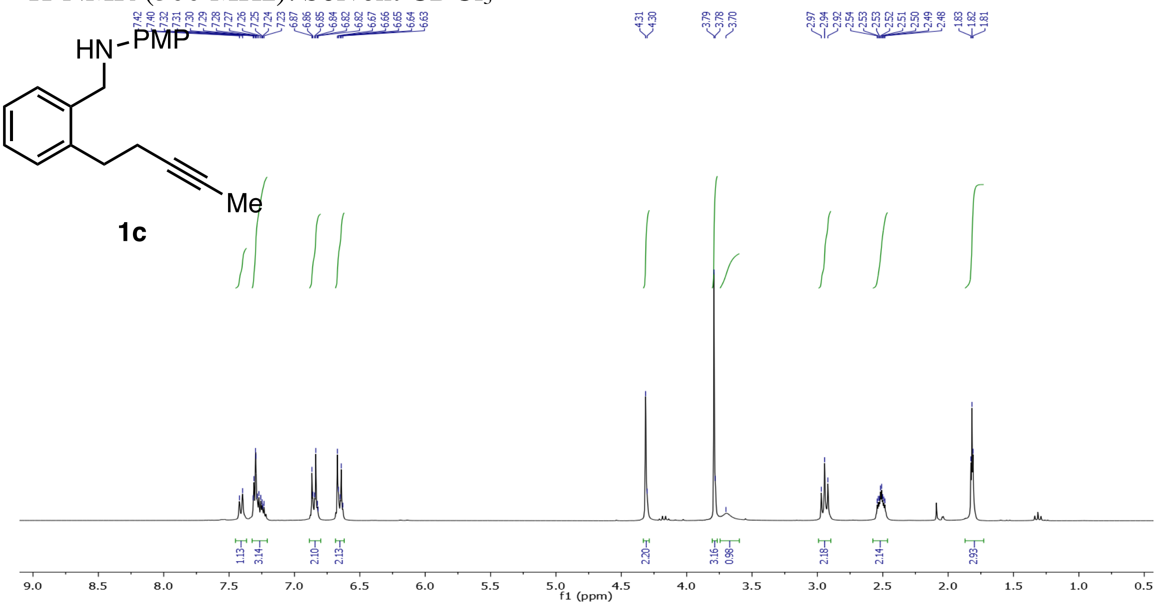

^{13}C-NMR(75 MHz). Solvent $CDCl_3$

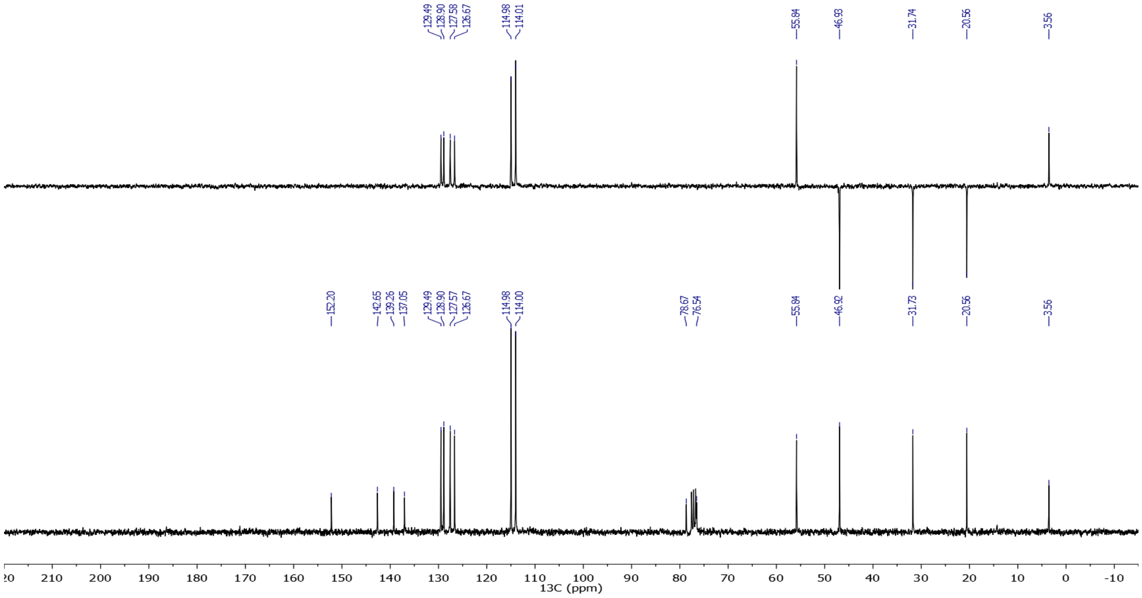

^{1}H-NMR (300 MHz). Solvent DMSO-d_6. 80º C

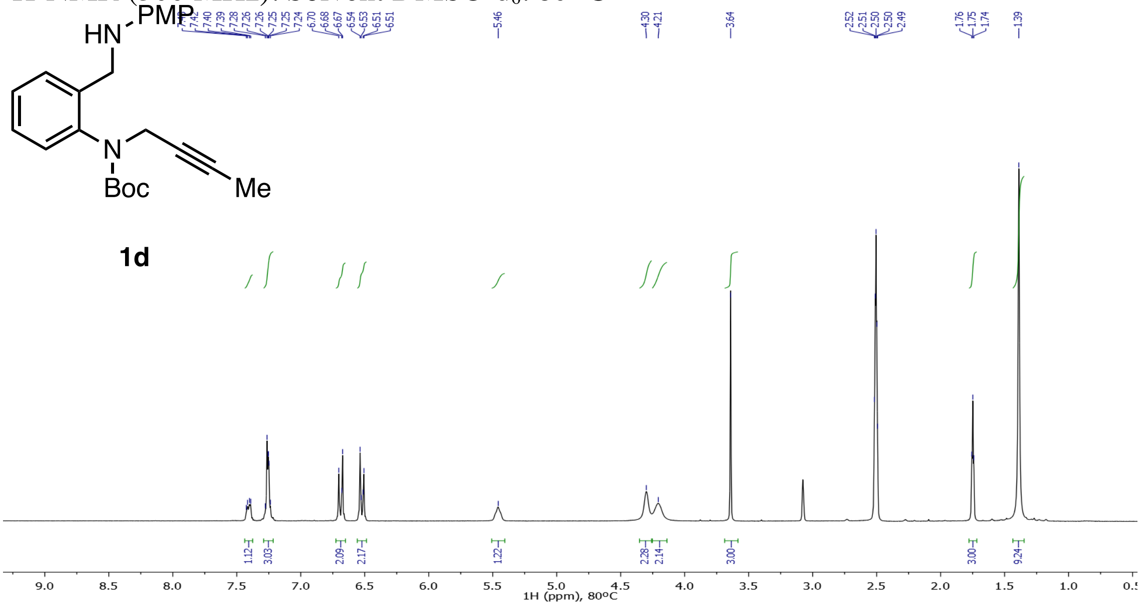

^{13}C-NMR(75MHz). Solvent CDMSO-$d_6$80 ºC

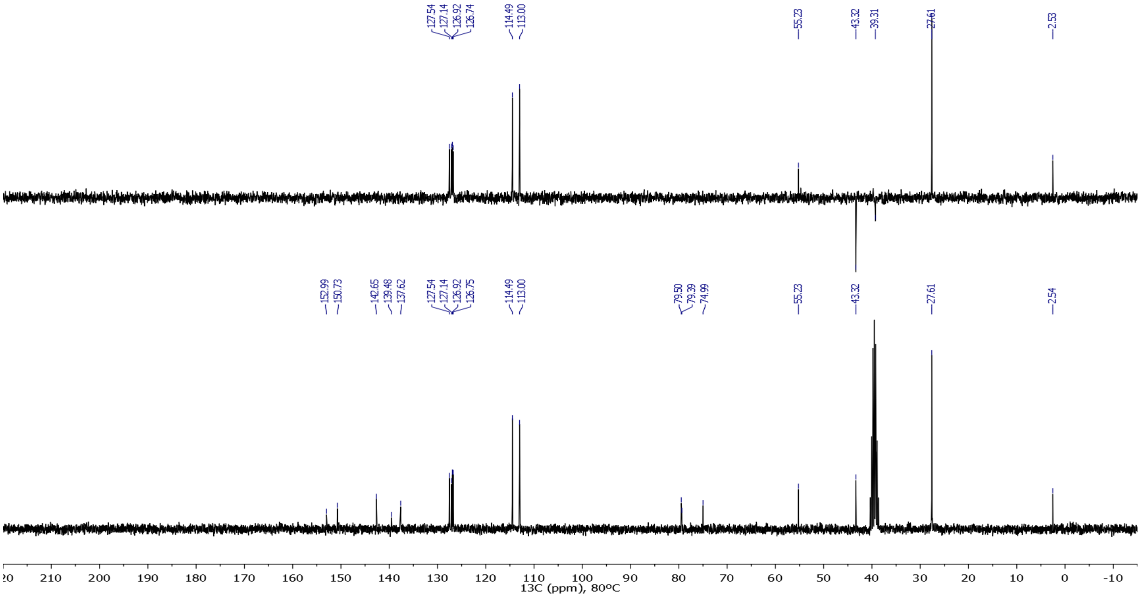

^{1}H-NMR (300 MHz). Solvent $CDCl_3$

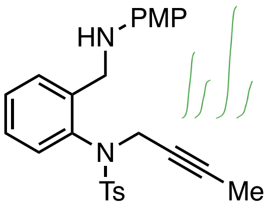

1e

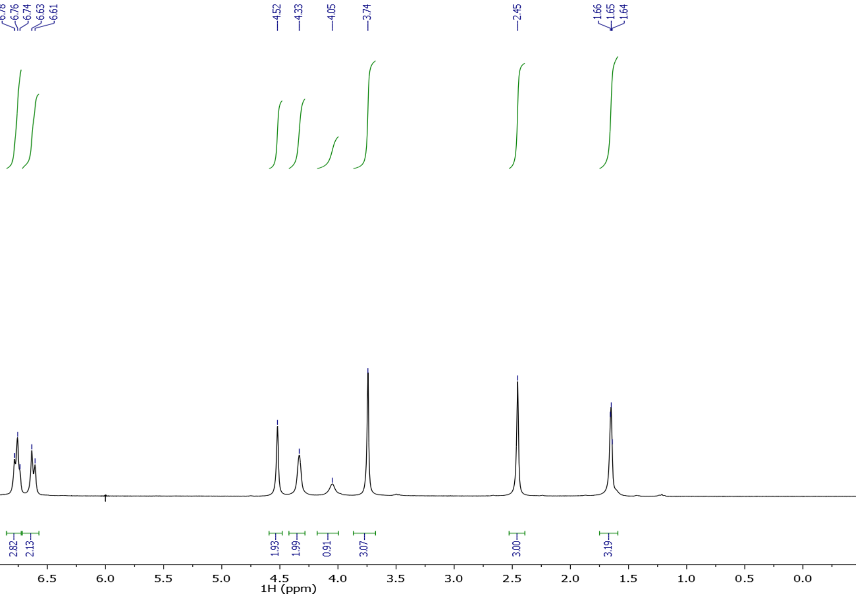

^{13}C-NMR (75 MHz). Solvent $CDCl_3$

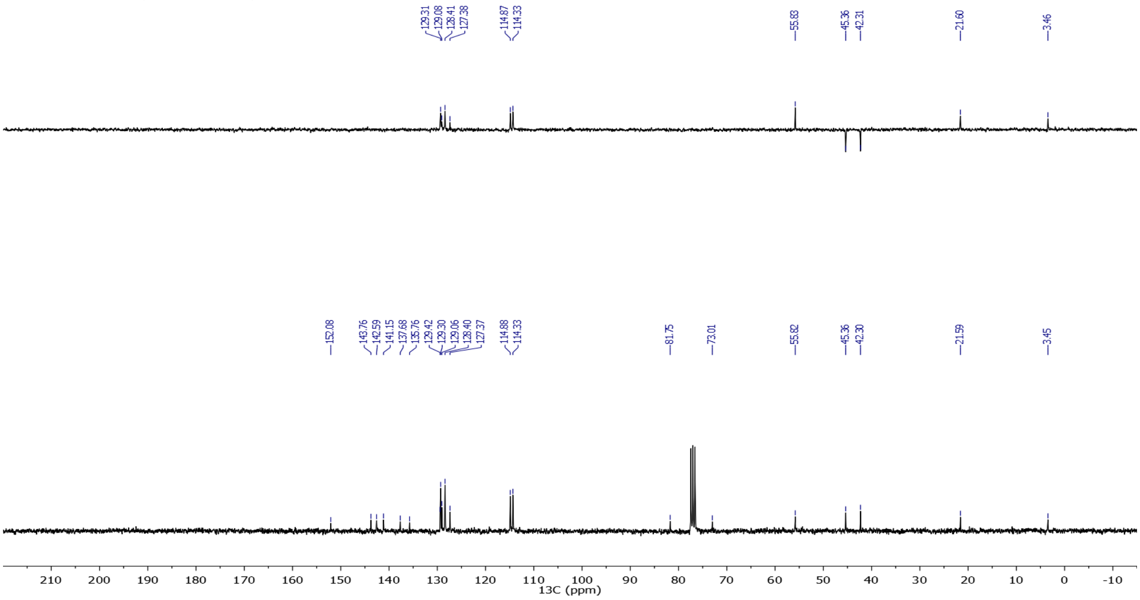

^{1}H-NMR (500 MHz). Solvent $CDCl_3$

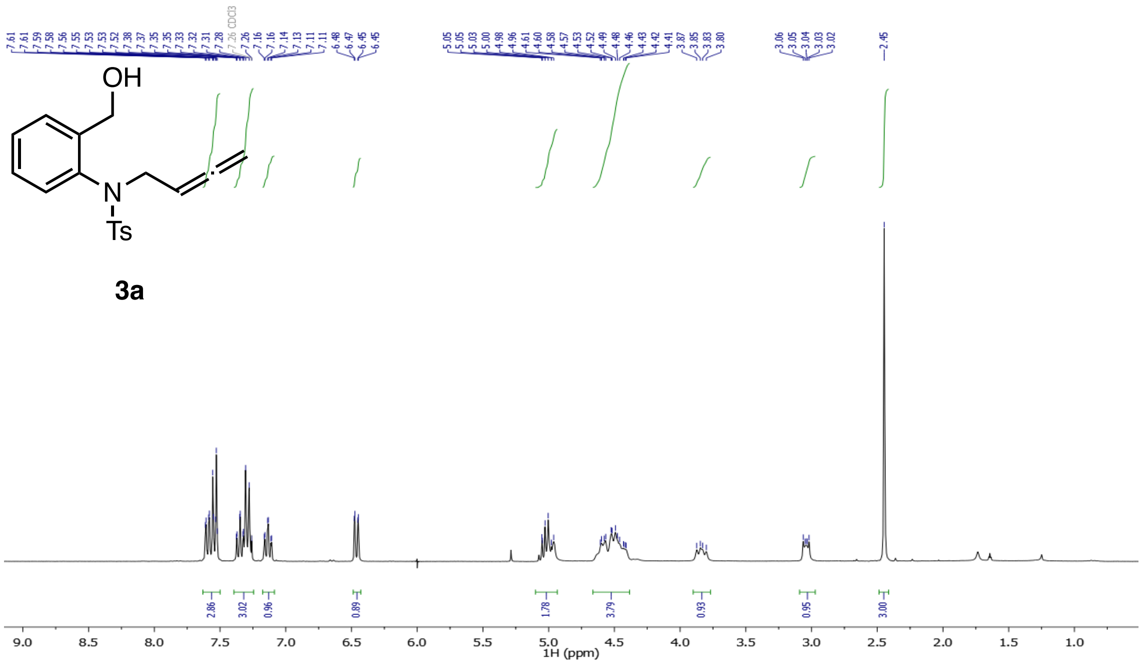

3a

^{13}C-NMR (126 MHz). Solvent $CDCl_3$

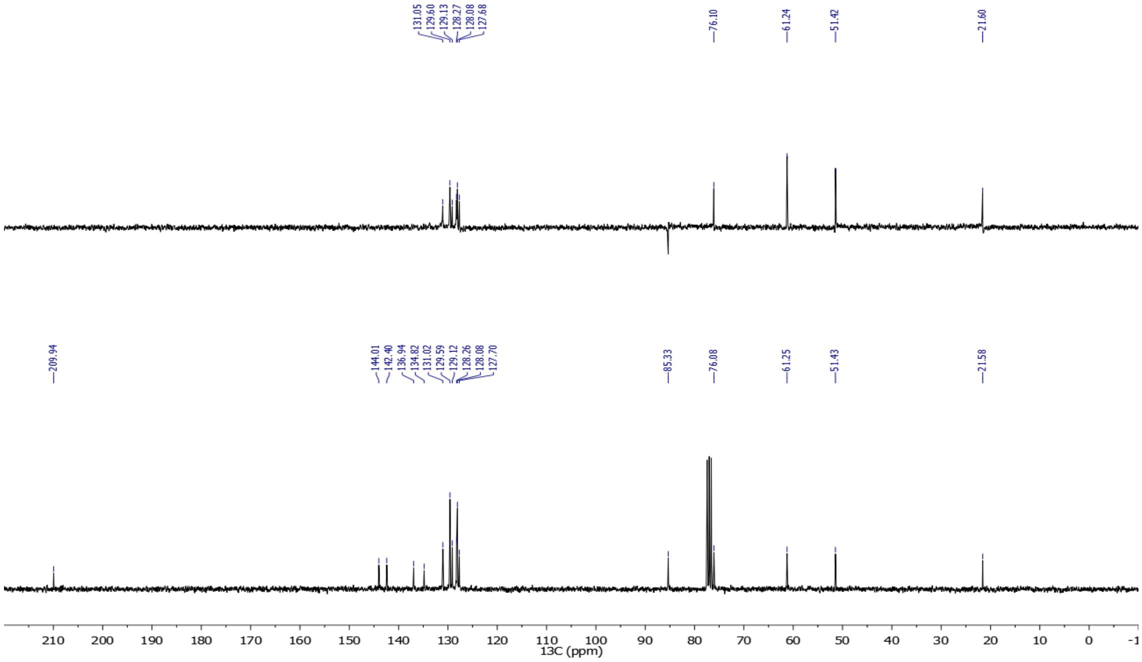

^{1}H-NMR (300 MHz). Solvent DMSO-d_6

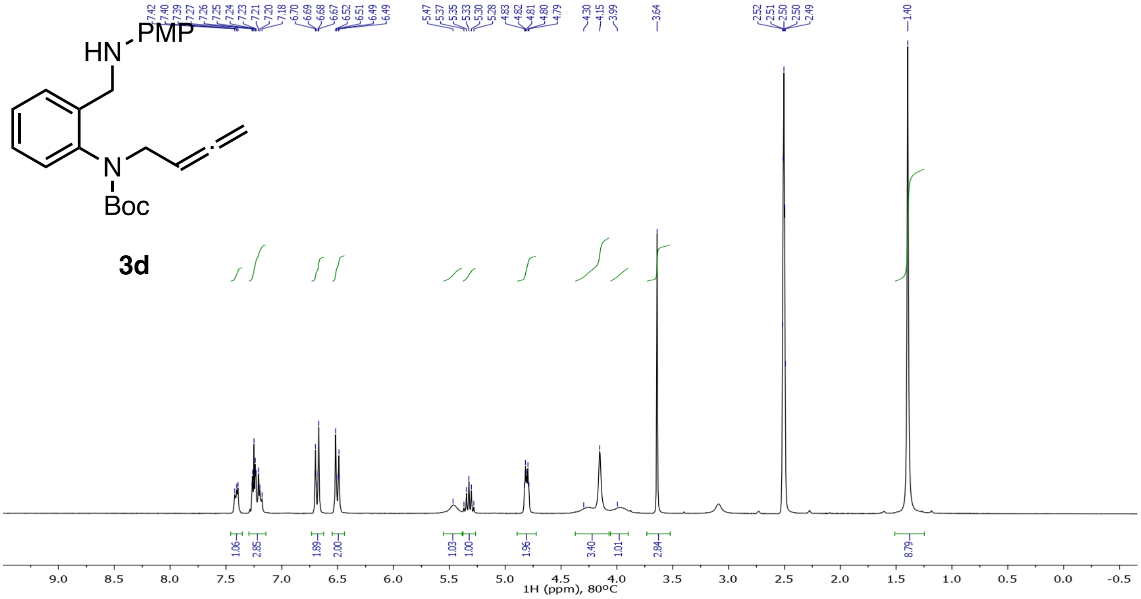

^{13}C-NMR (75 MHz). Solvent DMSO-d_6

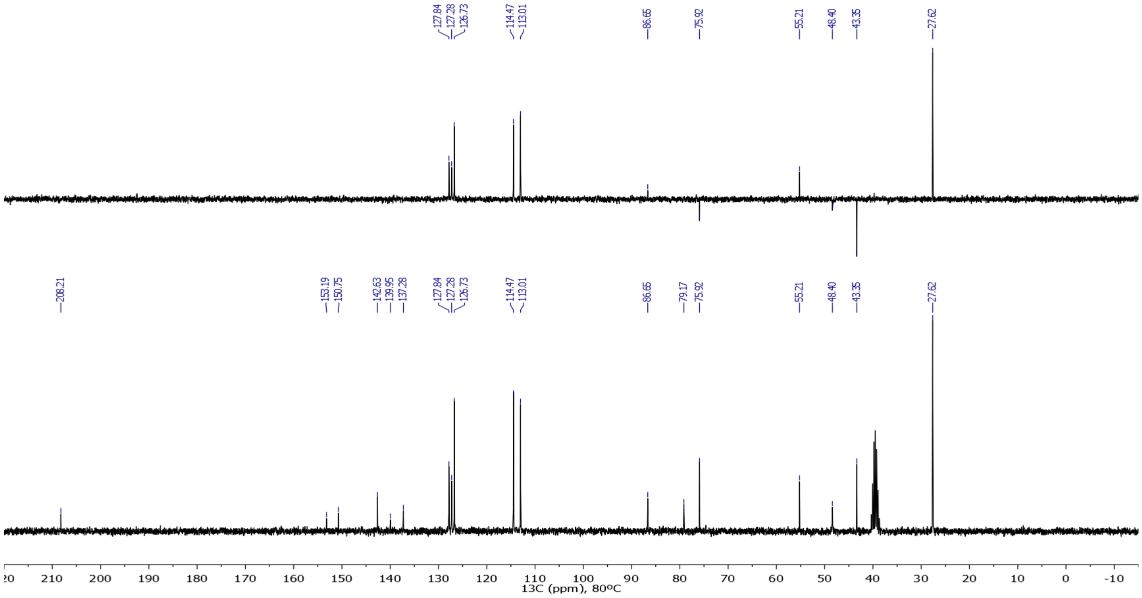

^{1}H-NMR (500 MHz). Solvent $CDCl_3$

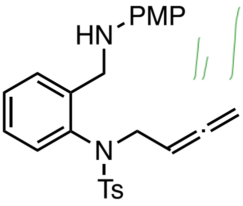

3e

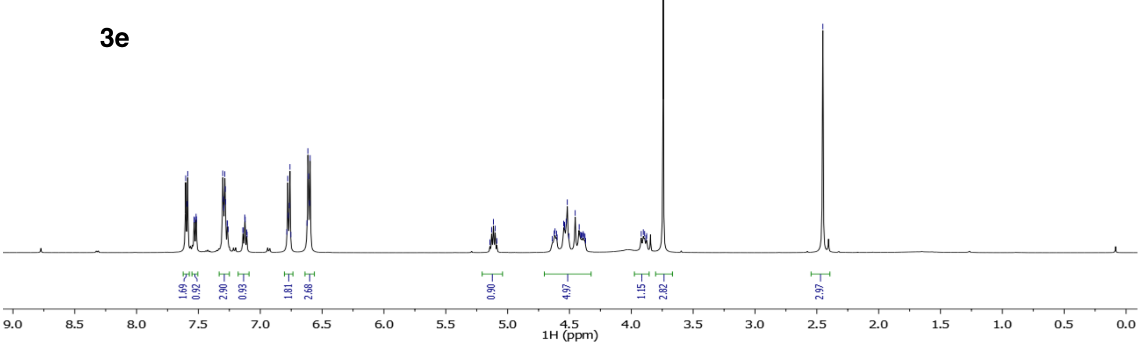

^{13}C-NMR(126 MHz). Solvent $CDCl_3$

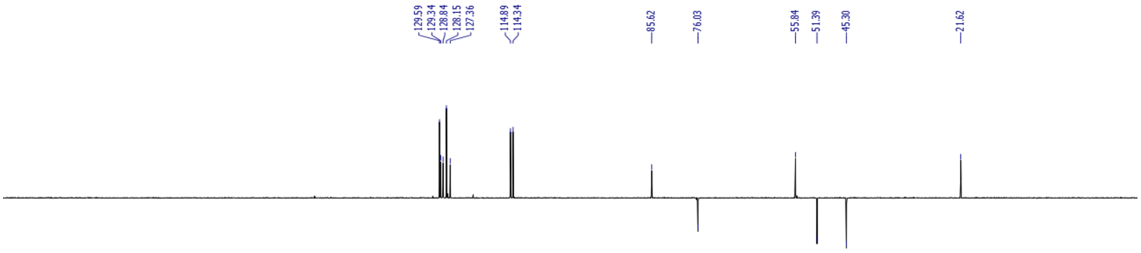

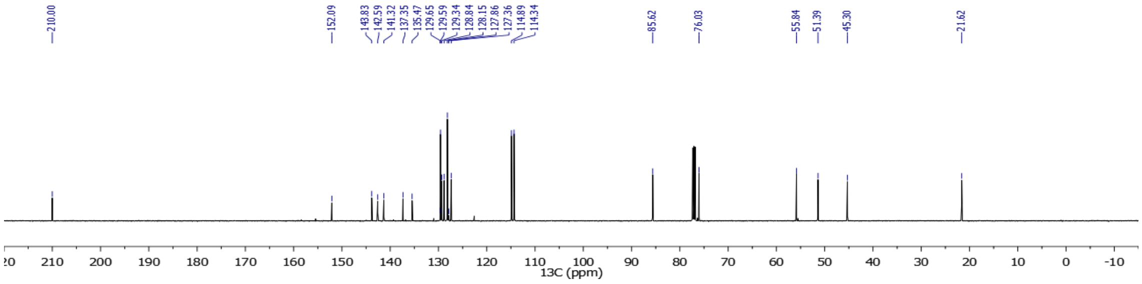

^{1}H-NMR (300 MHz). Solvent $CDCl_3$

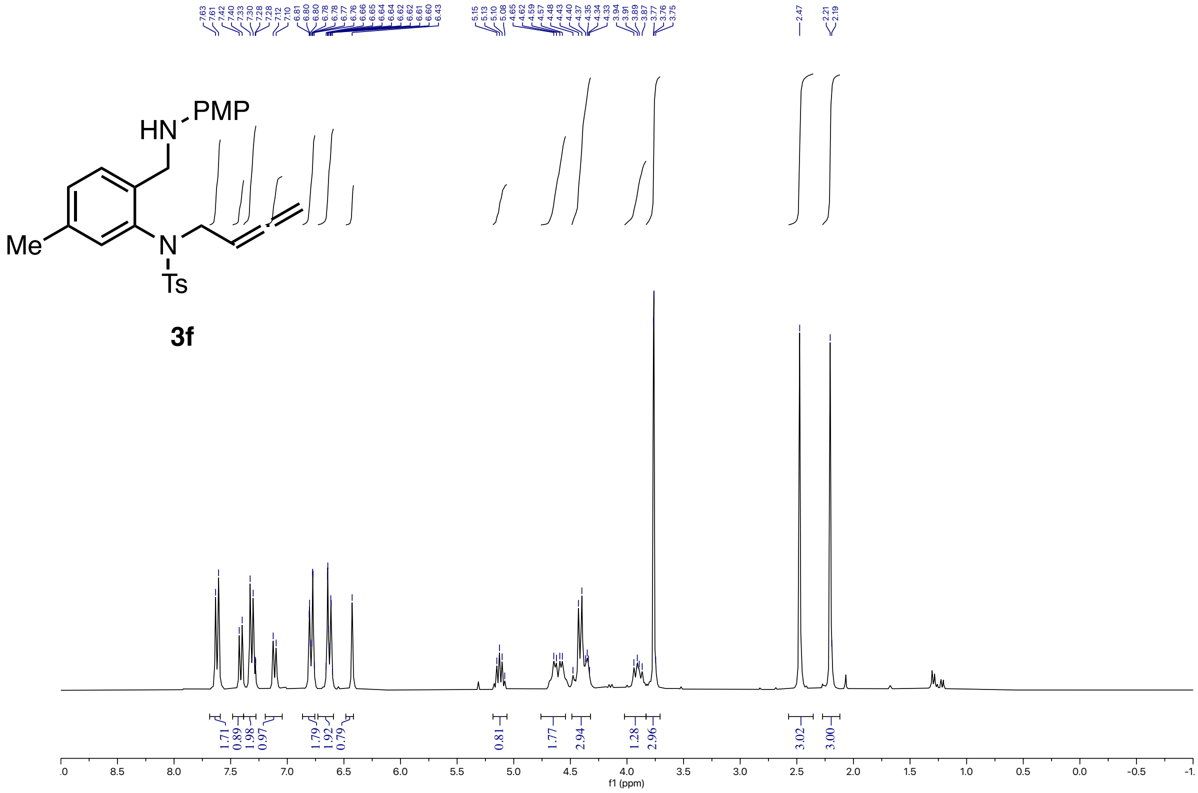

3f

^{13}C-NMR (75 MHz). Solvent $CDCl_3$

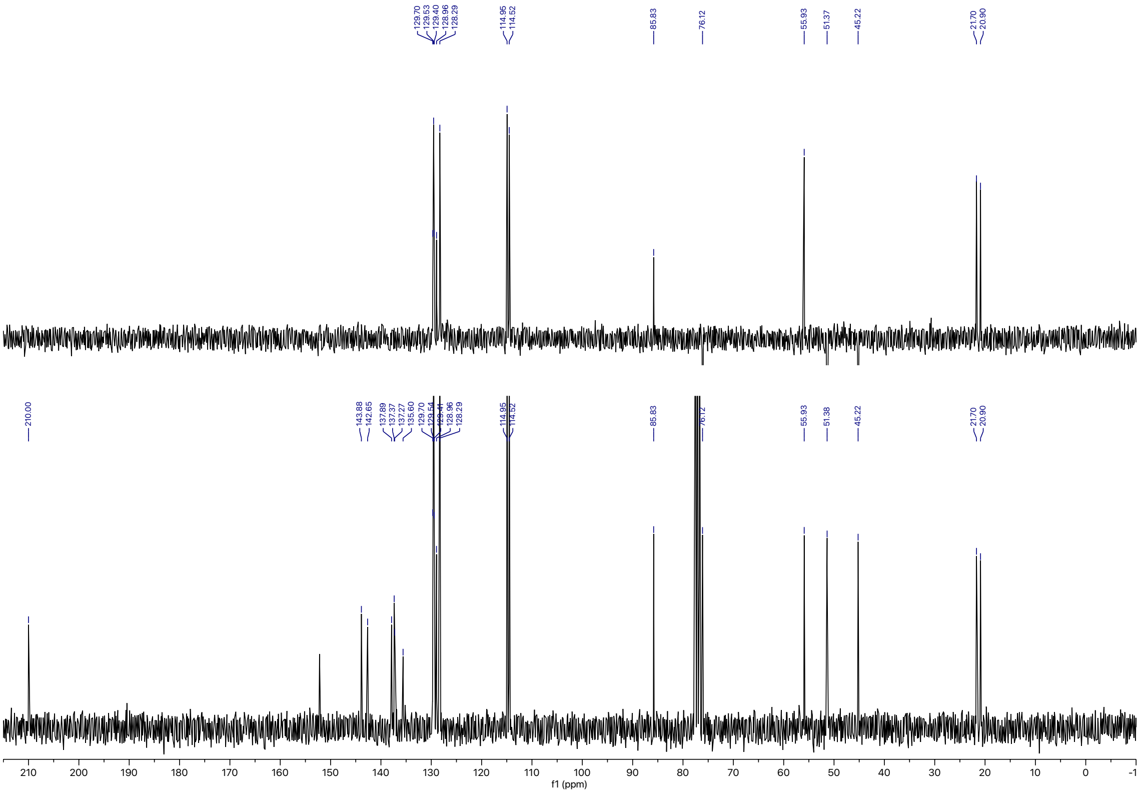

^{1}H-NMR (500 MHz). Solvent $CDCl_3$

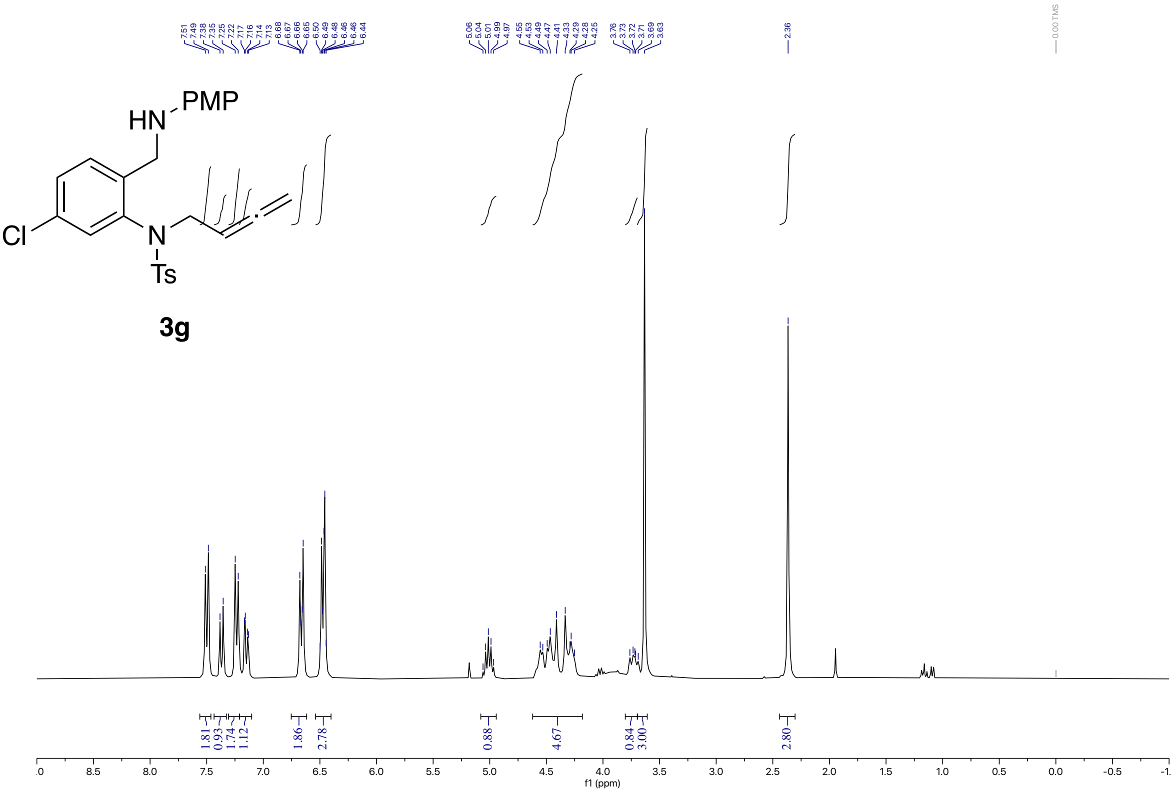

^{13}C-NMR (126 MHz). Solvent $CDCl_3$

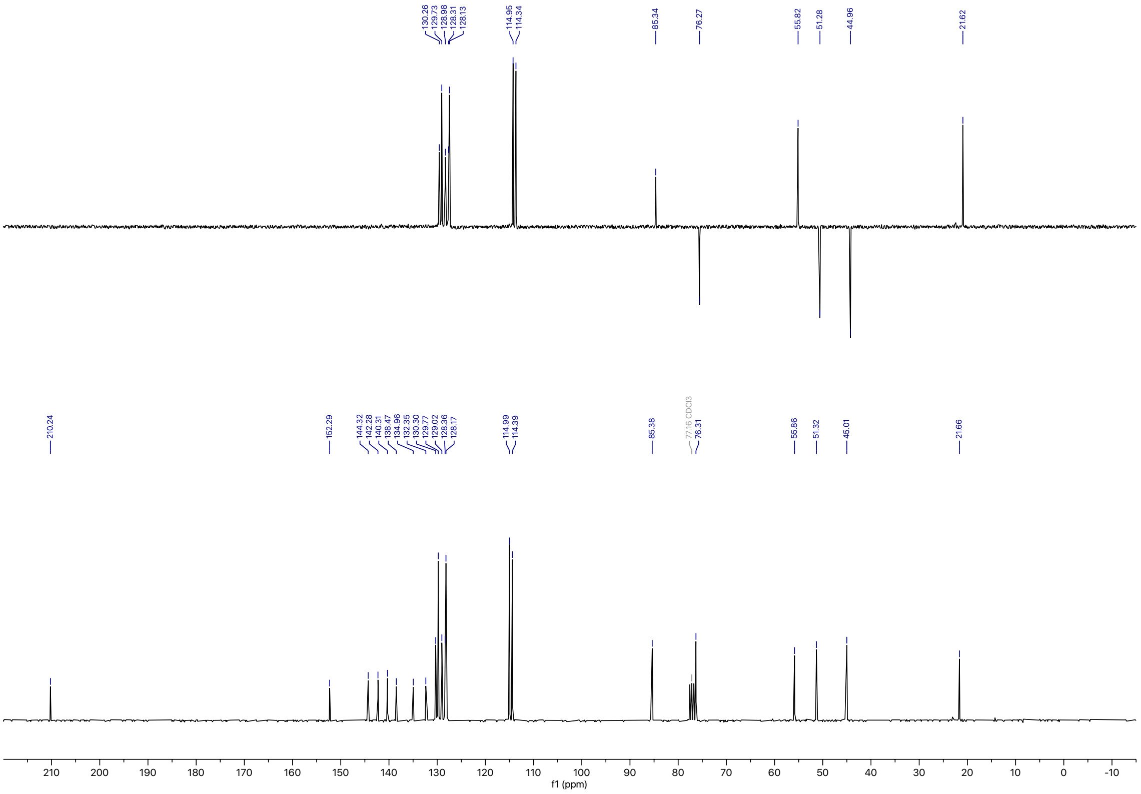

^{1}H-NMR (500 MHz). Solvent $CDCl_3$

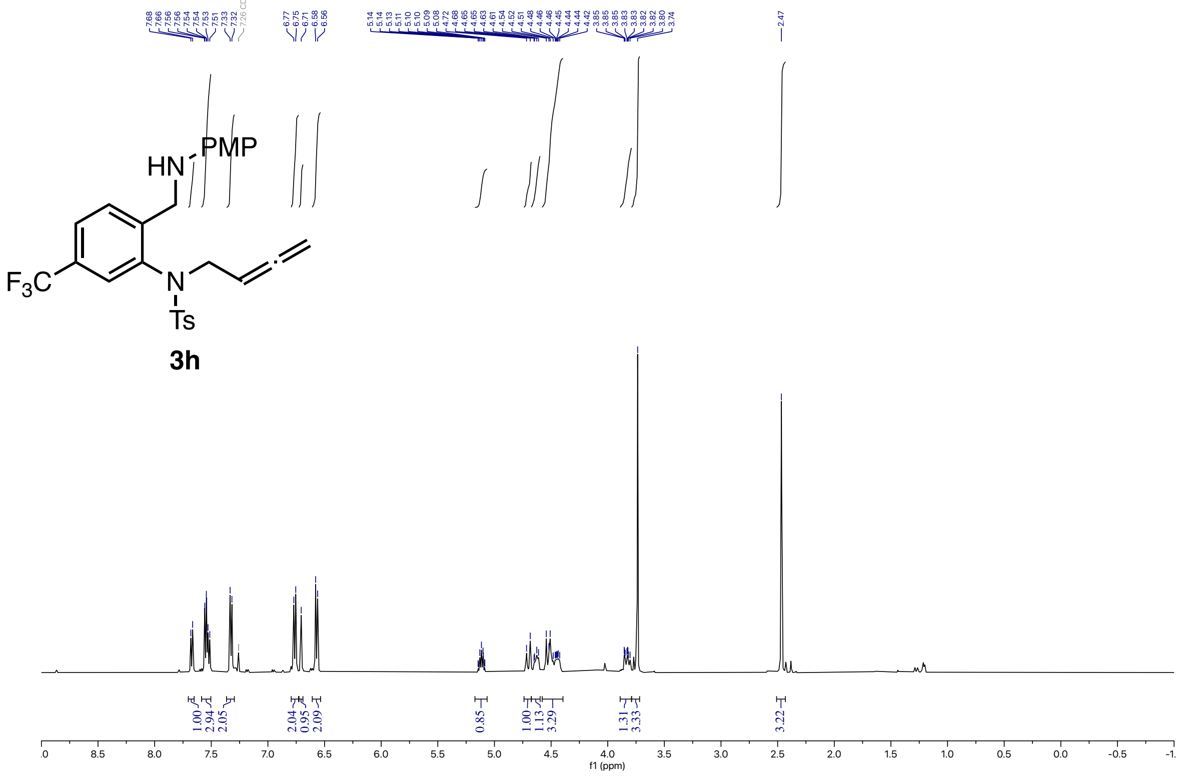

^{13}C-NMR (126 MHz). Solvent $CDCl_3$

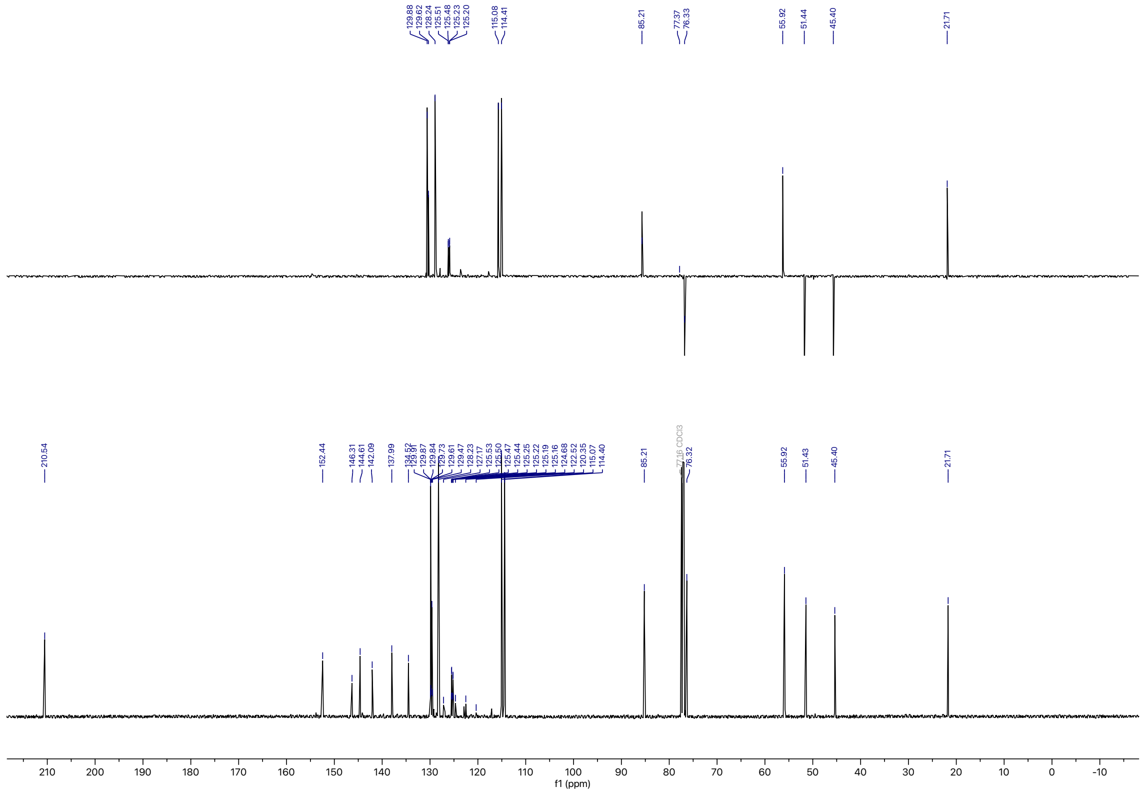

^{19}F-NMR (282 MHz). Solvent $CDCl_3$

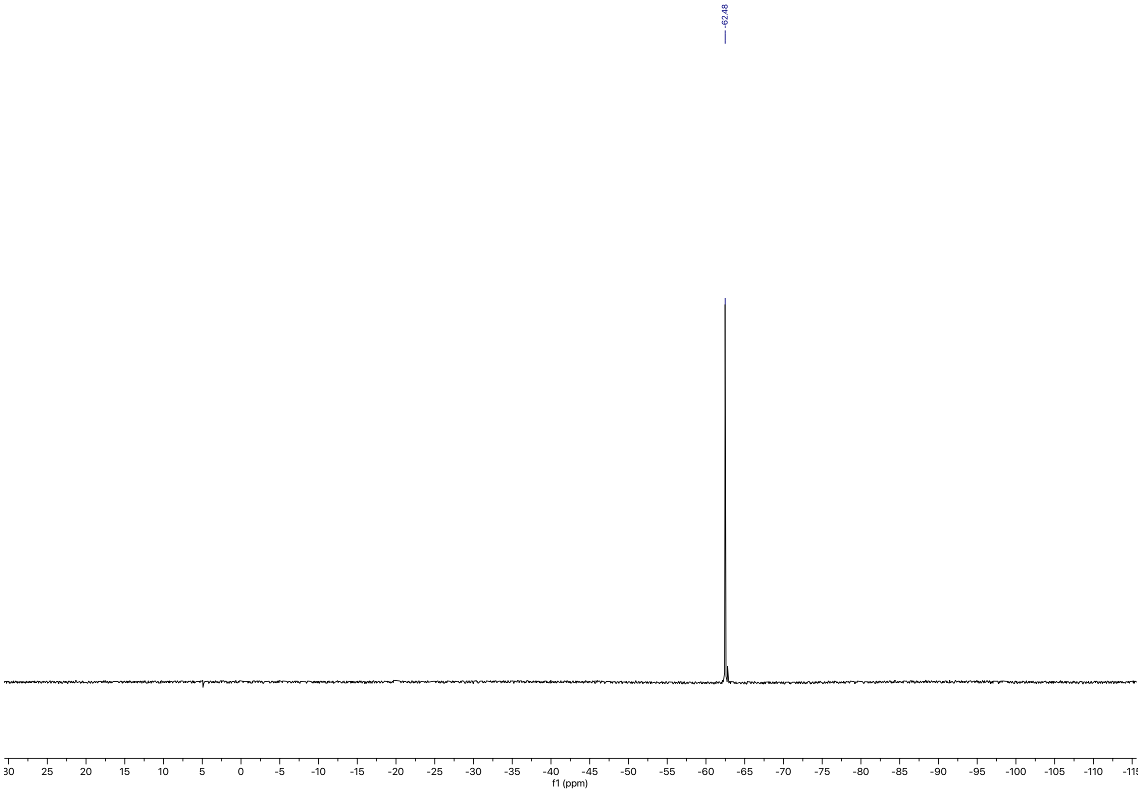

^{1}H-NMR (500 MHz). Solvent $CDCl_3$

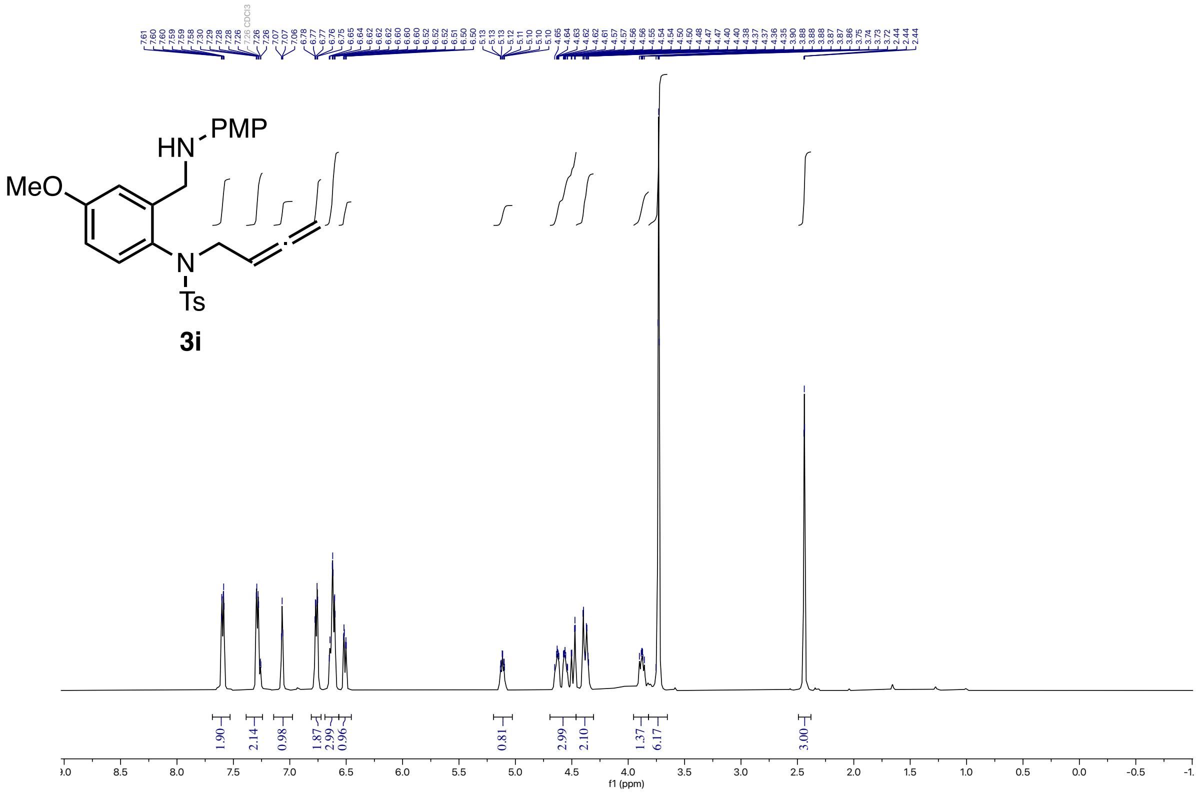

^{13}C-NMR (126 MHz). Solvent $CDCl_3$

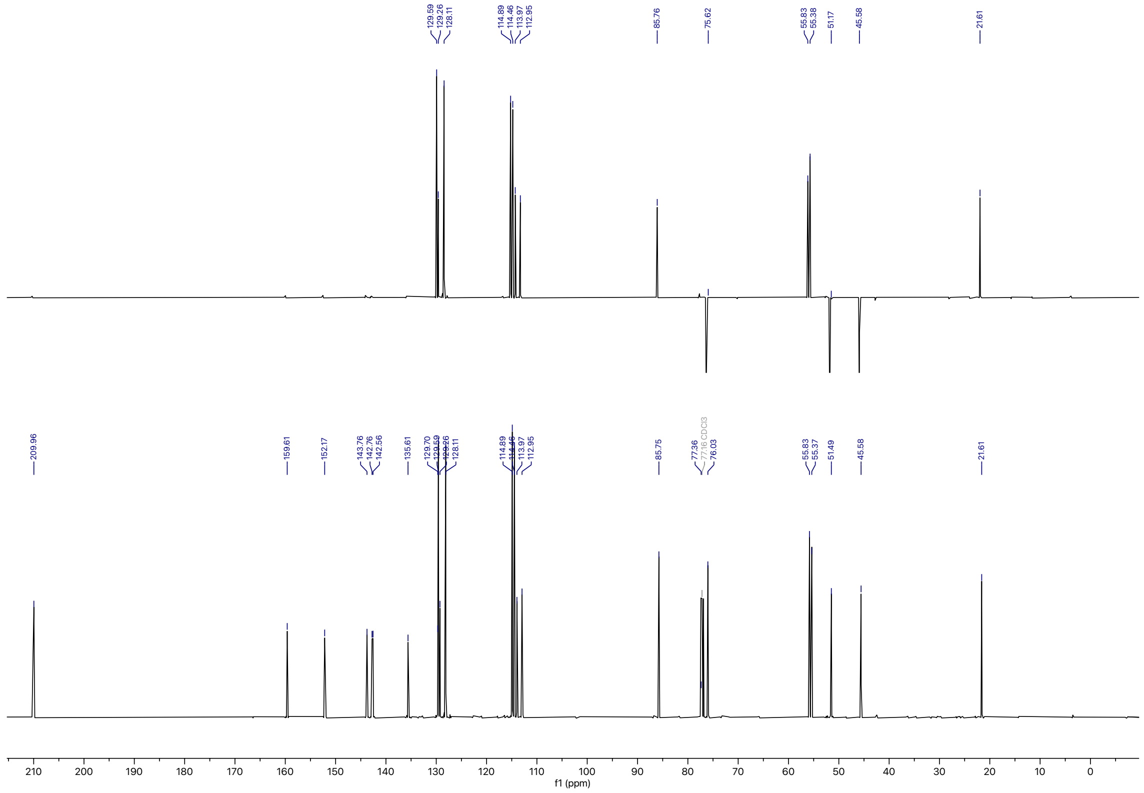

^{1}H-NMR (300 MHz). Solvent $CDCl_3$

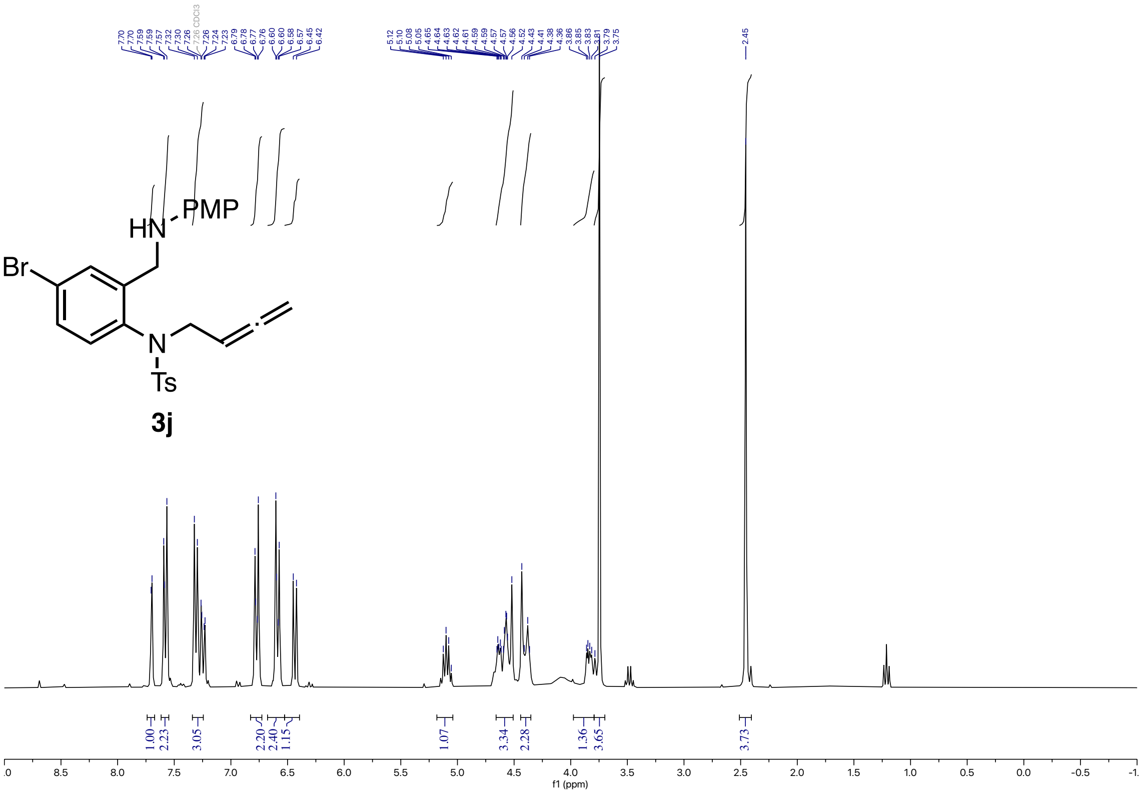

^{13}C-NMR (75 MHz). Solvent $CDCl_3$

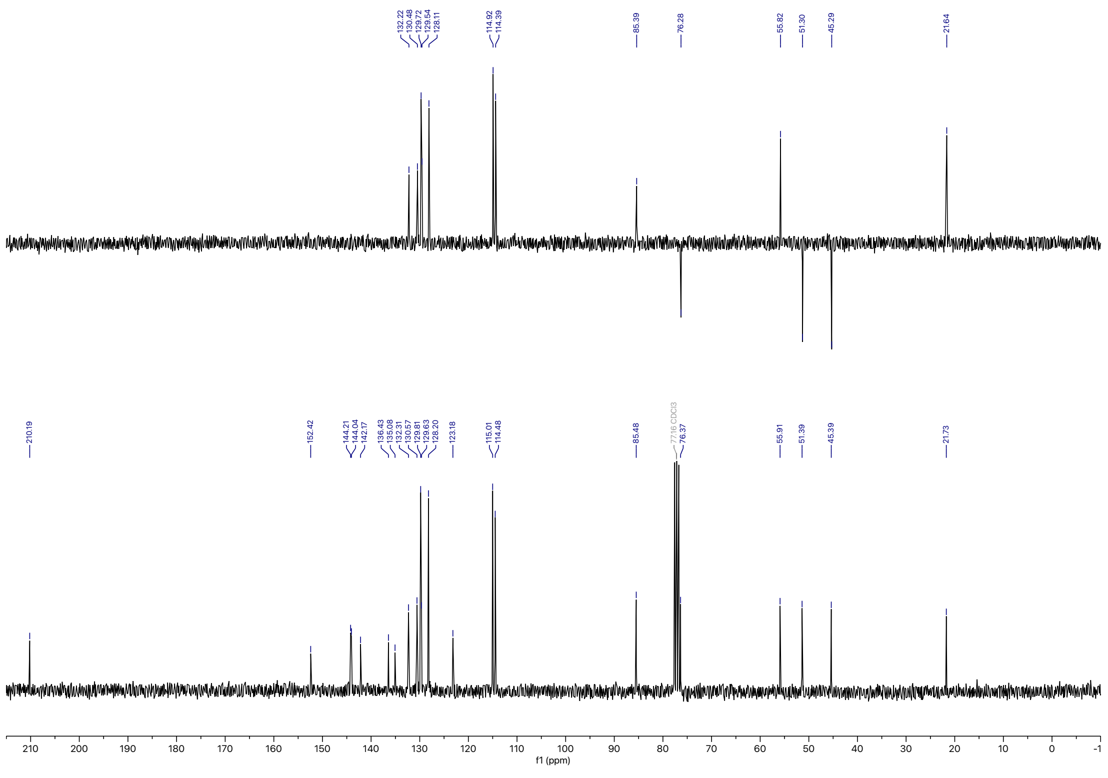

^{1}H-NMR (500 MHz). Solvent $CDCl_3$

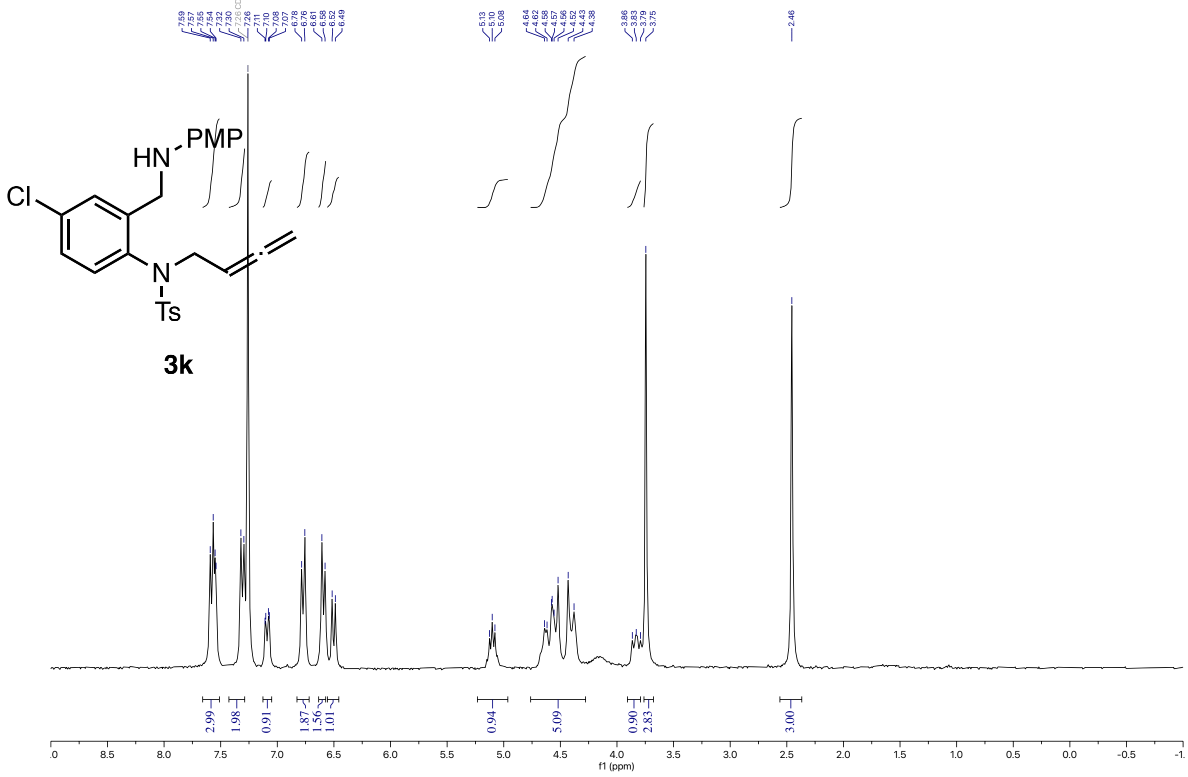

^{13}C-NMR (126 MHz). Solvent $CDCl_3$

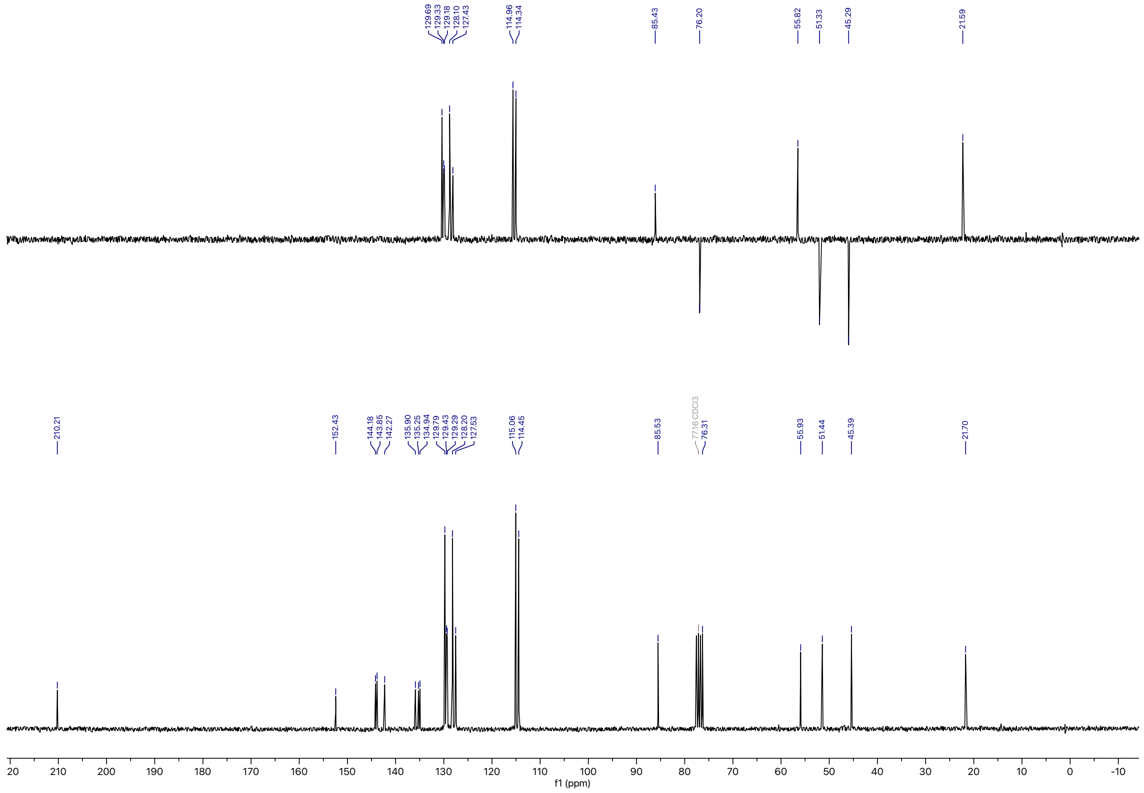

^{1}H-NMR (500 MHz). Solvent $CDCl_3$

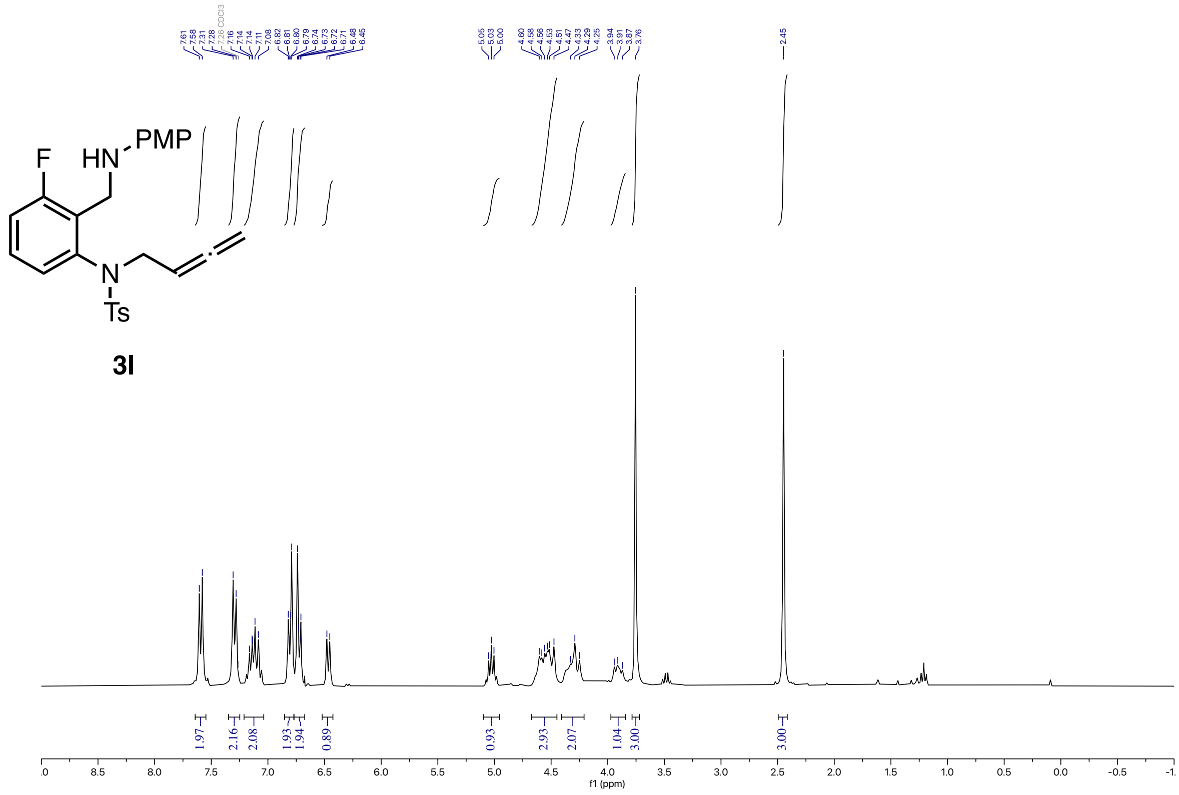

3l

^{13}C-NMR (126 MHz). Solvent $CDCl_3$

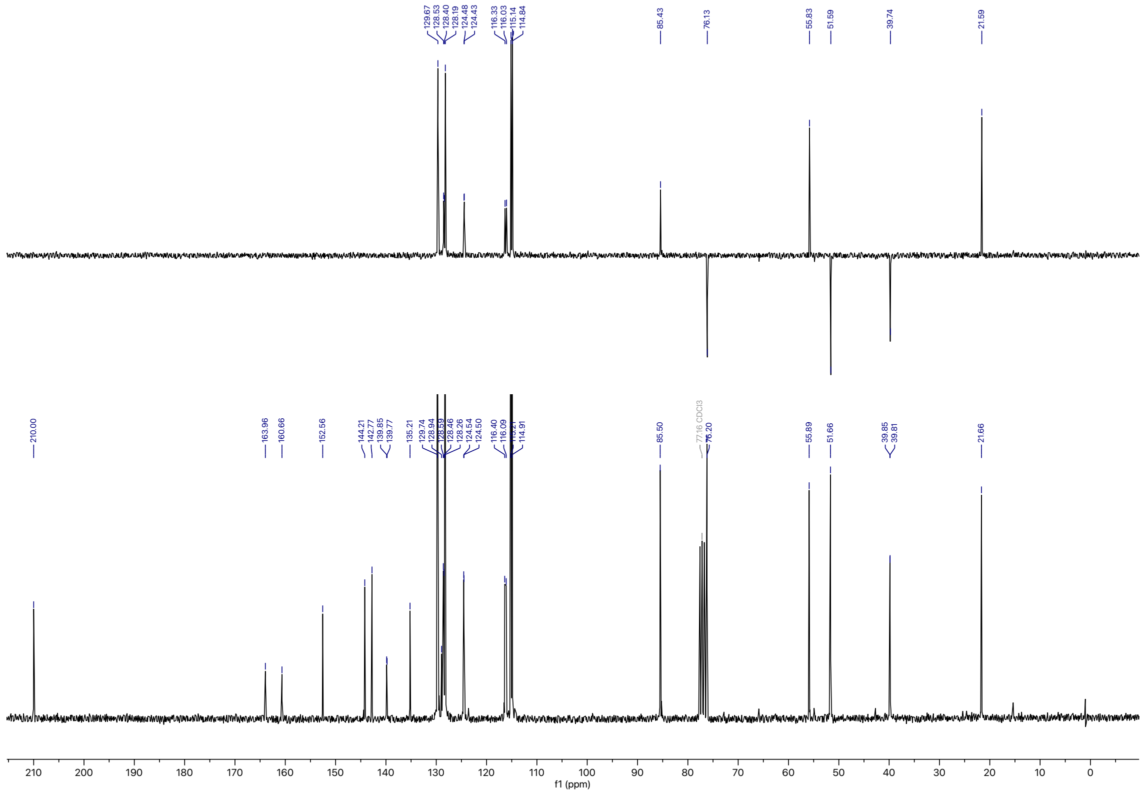

^{19}F-NMR (282 MHz). Solvent $CDCl_3$

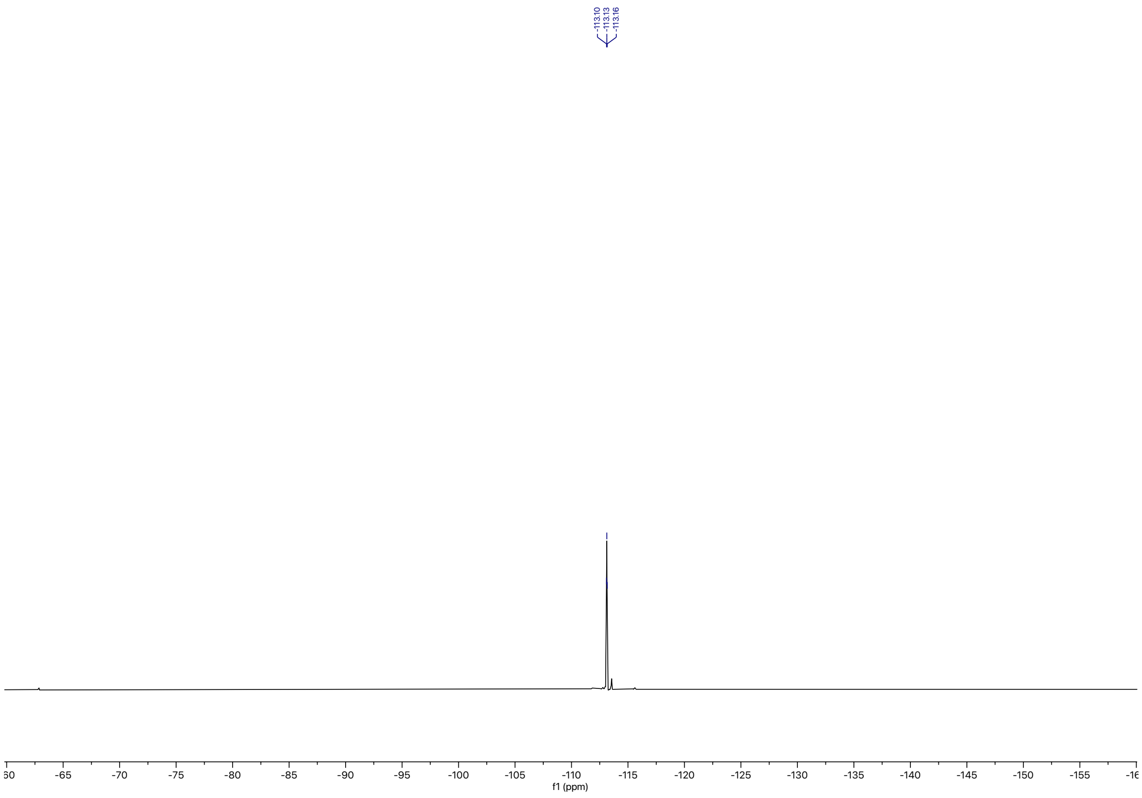

^{1}H-NMR (500 MHz). Solvent $CDCl_3$

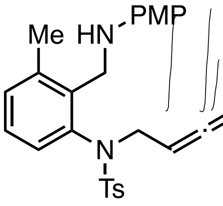

3m

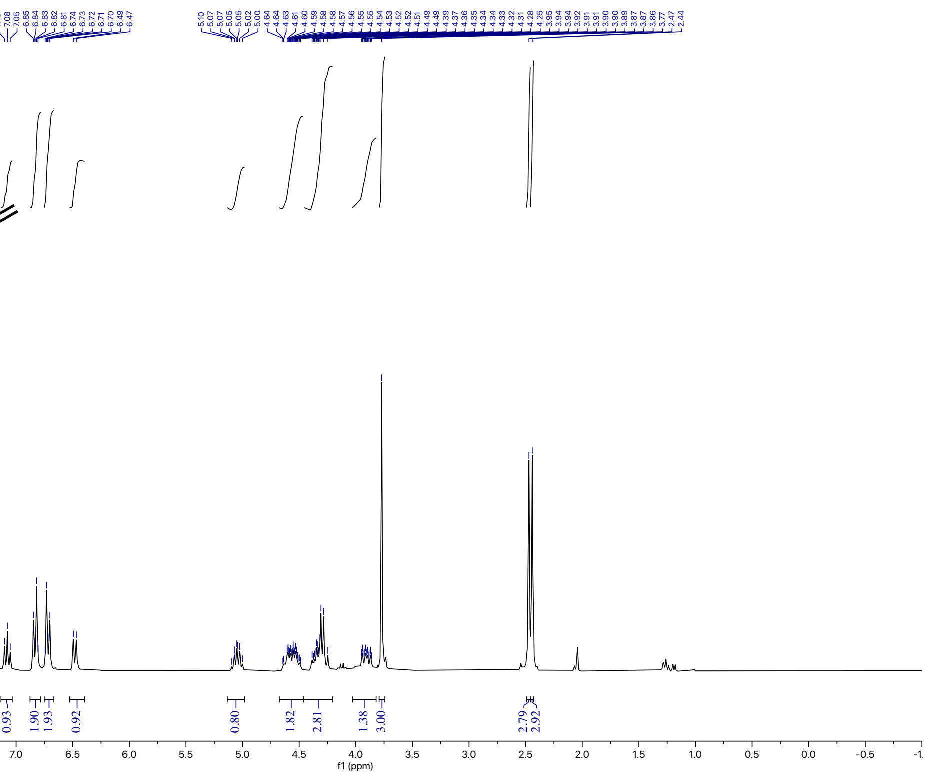

^{13}C-NMR (126 MHz). Solvent $CDCl_3$

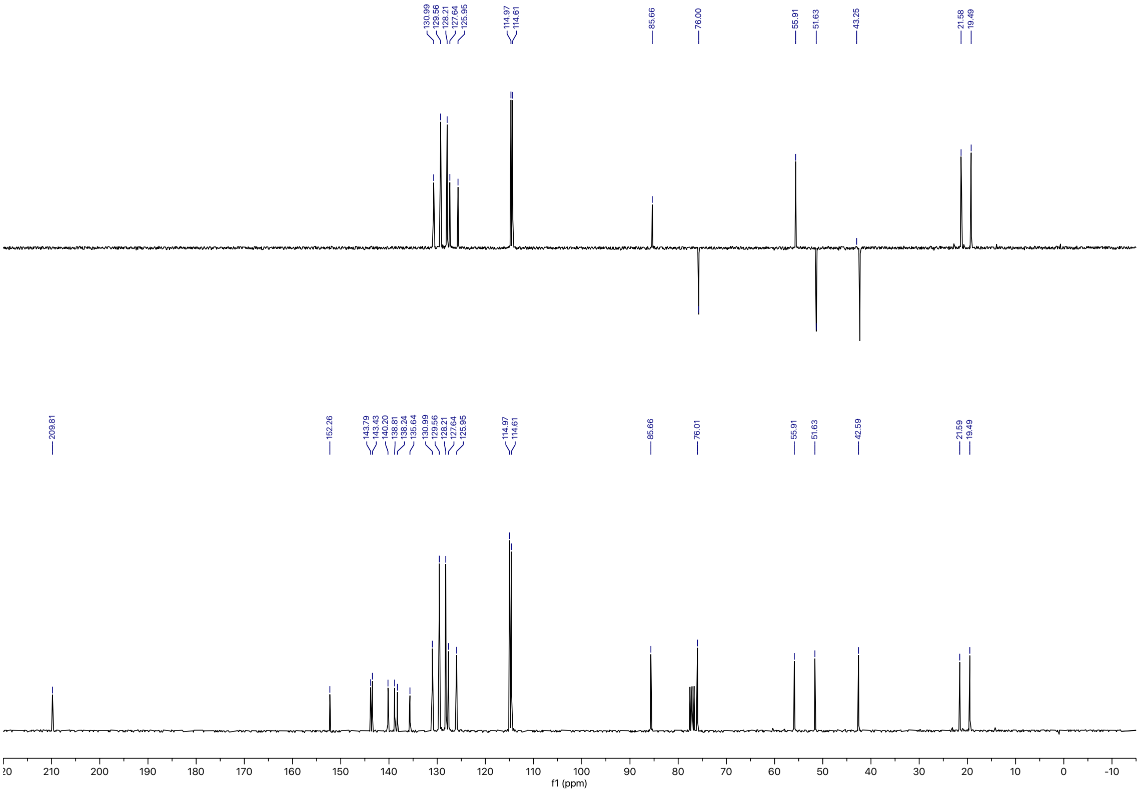

^{1}H-NMR (500 MHz). Solvent $CDCl_3$

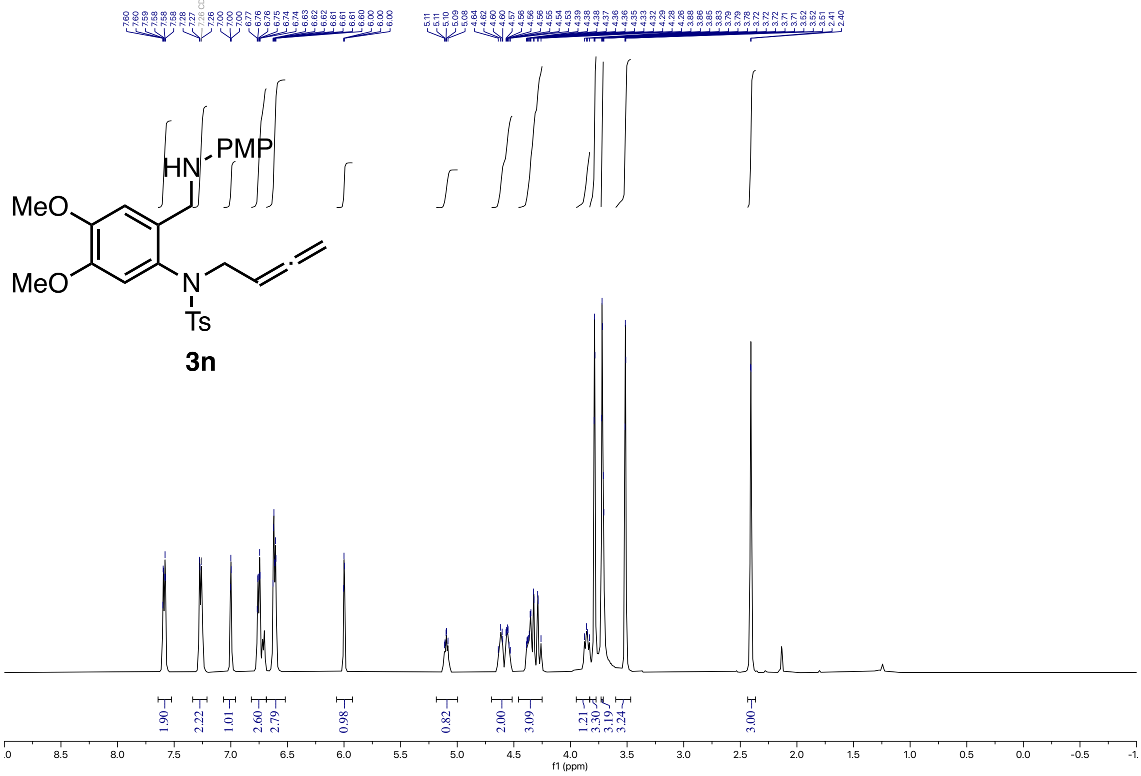

^{13}C-NMR (126 MHz). Solvent $CDCl_3$

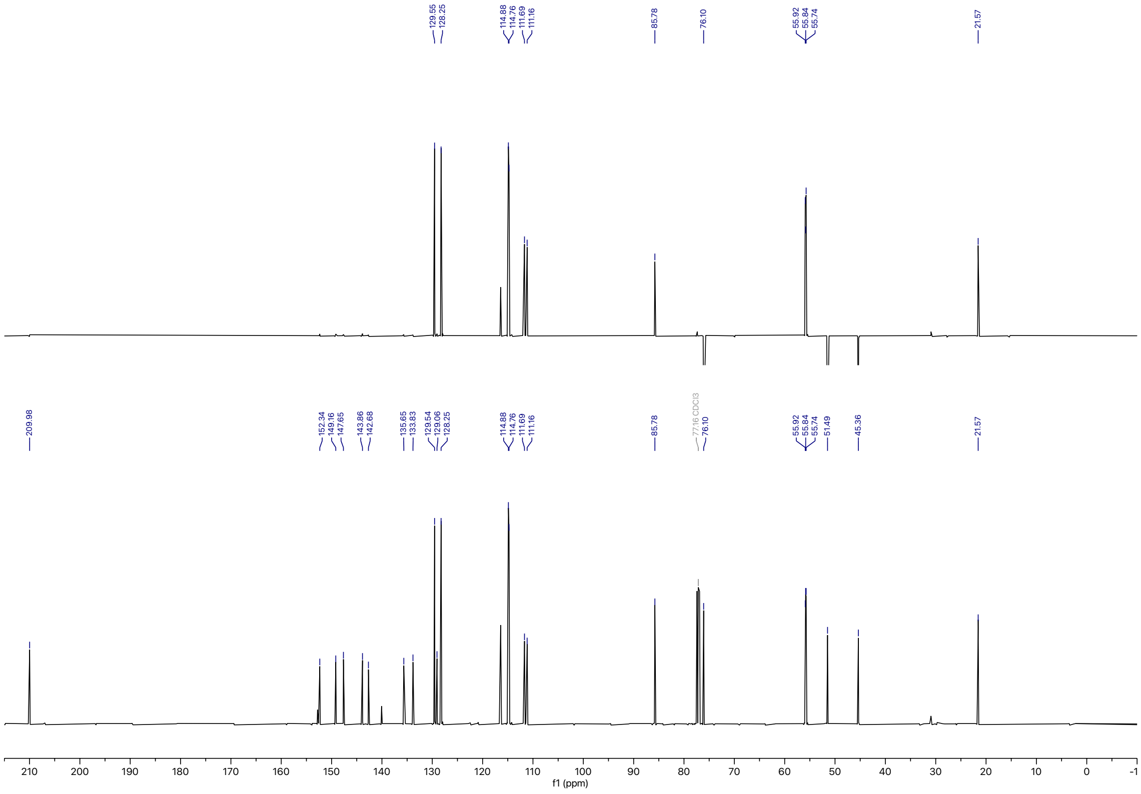

^{1}H-NMR (300 MHz). Solvent $CDCl_3$

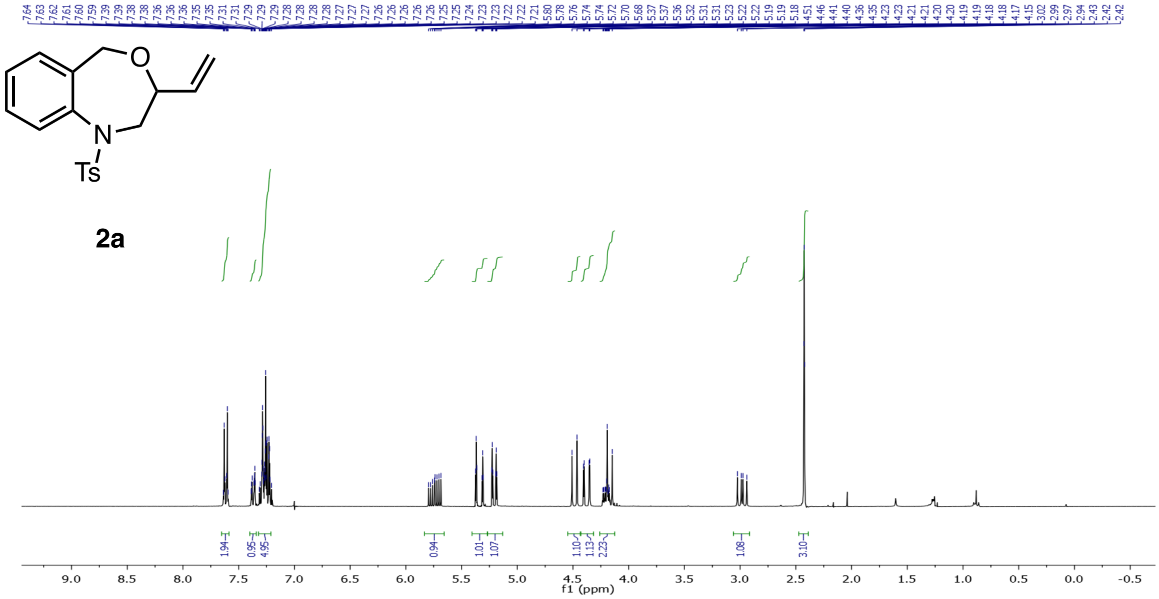

2a

^{13}C-NMR (75 MHz). Solvent $CDCl_3$

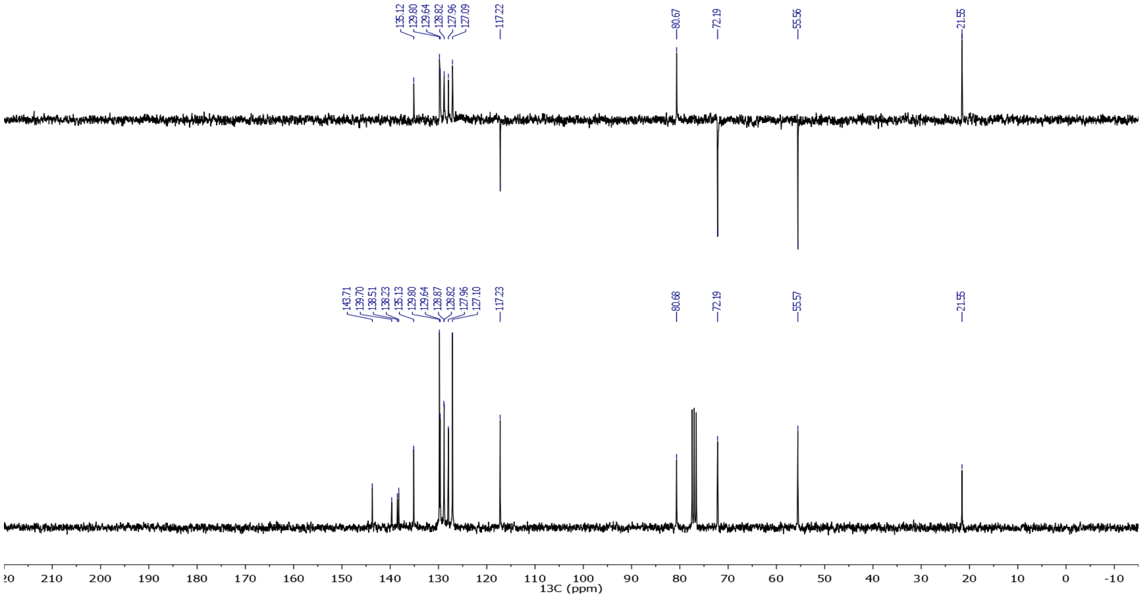

^{1}H-NMR (300 MHz). Solvent $CDCl_3$

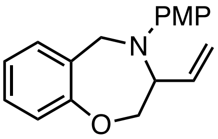

2b

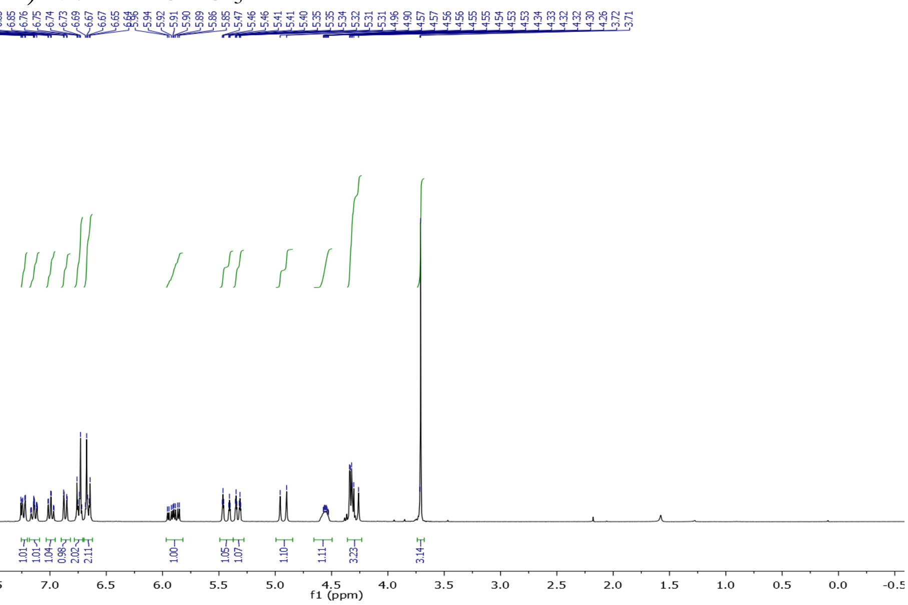

^{13}C-NMR (75 MHz). Solvent $CDCl_3$

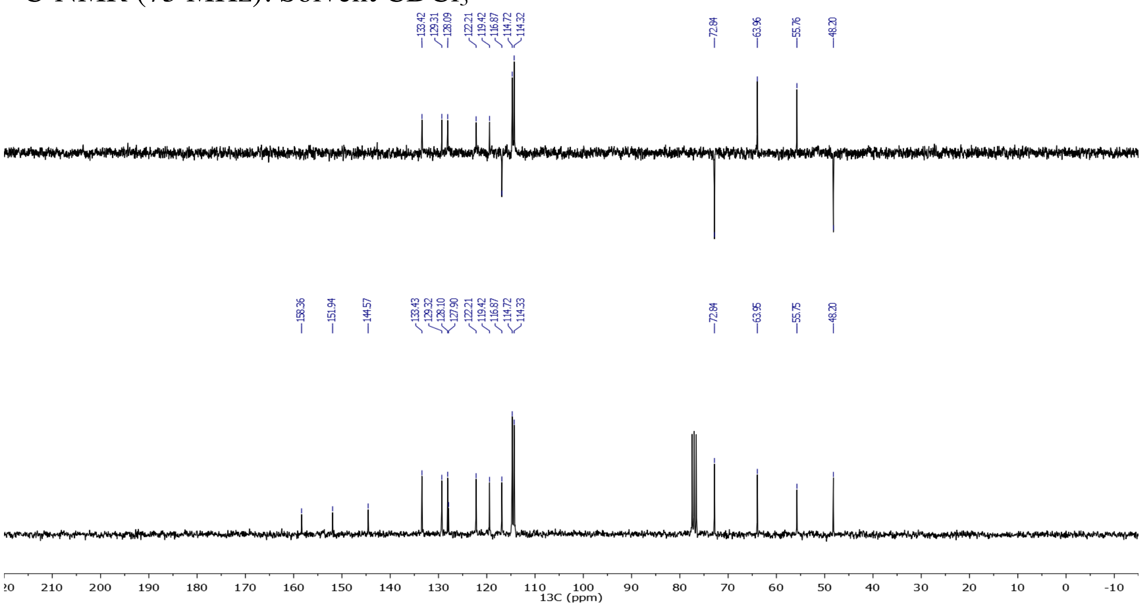

^{1}H-NMR (300 MHz). Solvent $CDCl_3$

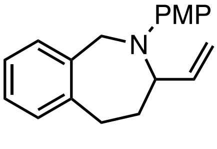

2c

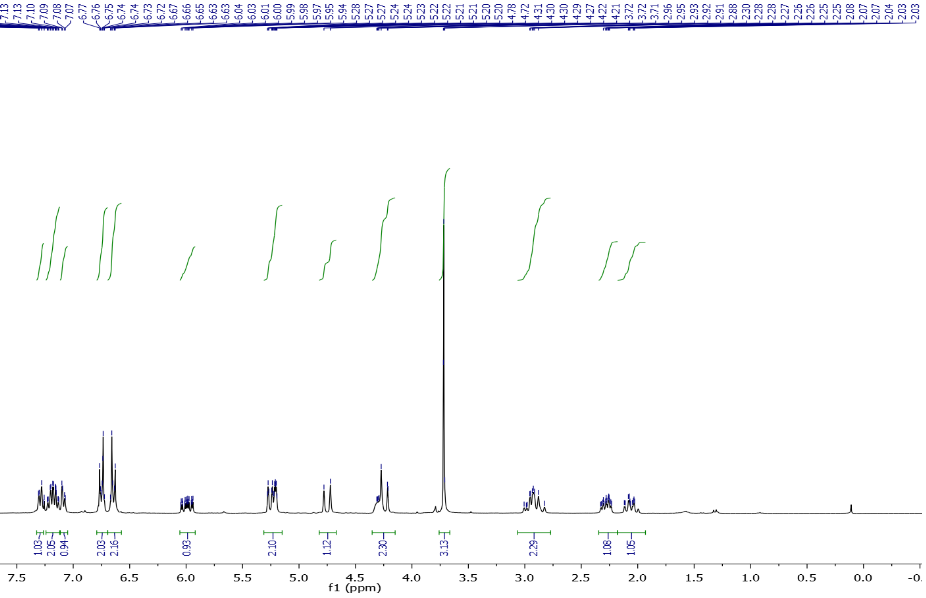

^{13}C-NMR (75 MHz). Solvent $CDCl_3$

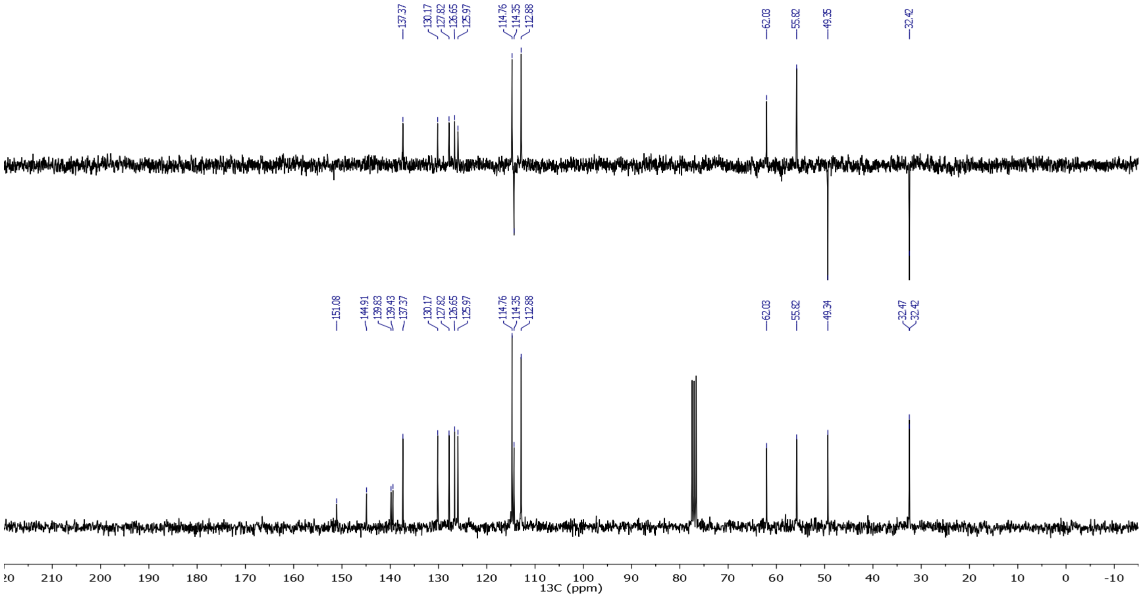

^{1}H-NMR (300 MHz). Solvent $CDCl_3$

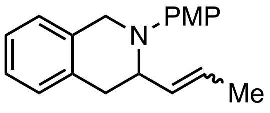

2c’

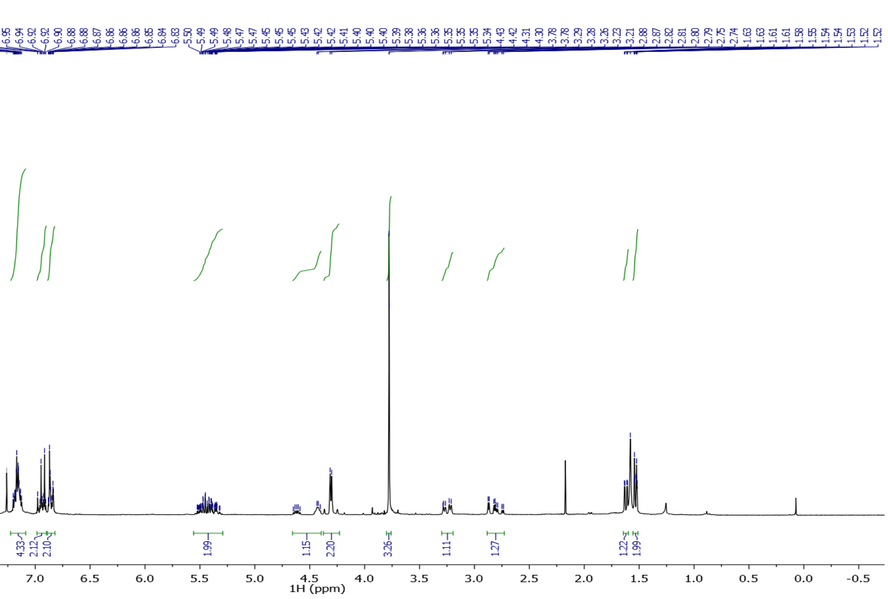

^{13}C-NMR (75 MHz). Solvent $CDCl_3$

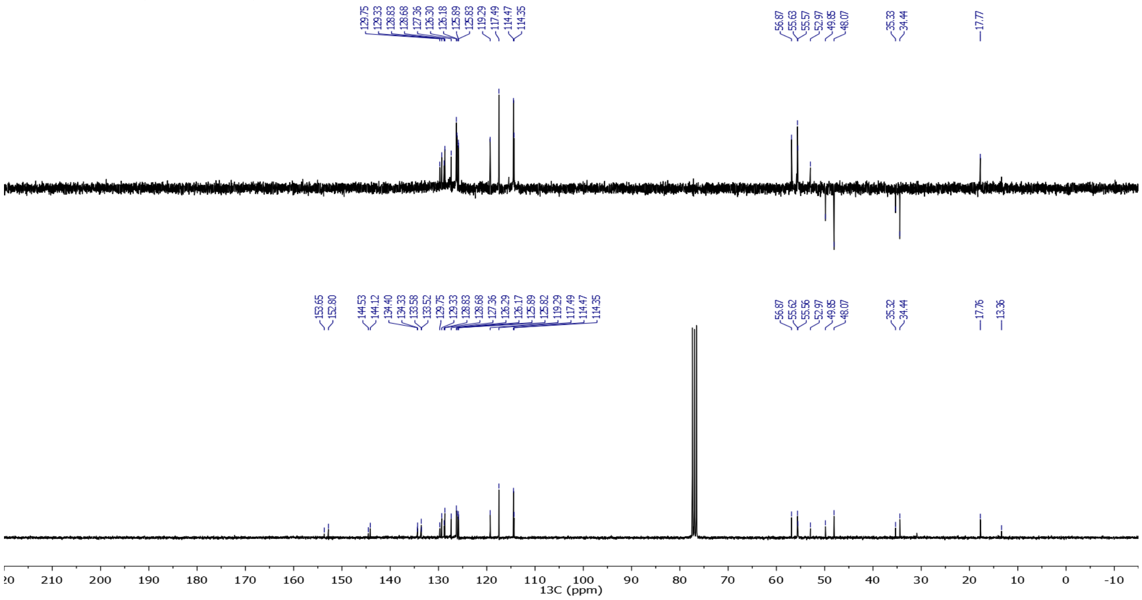

^{1}H-NMR(300 MHz). Solvent $CDCl_3$

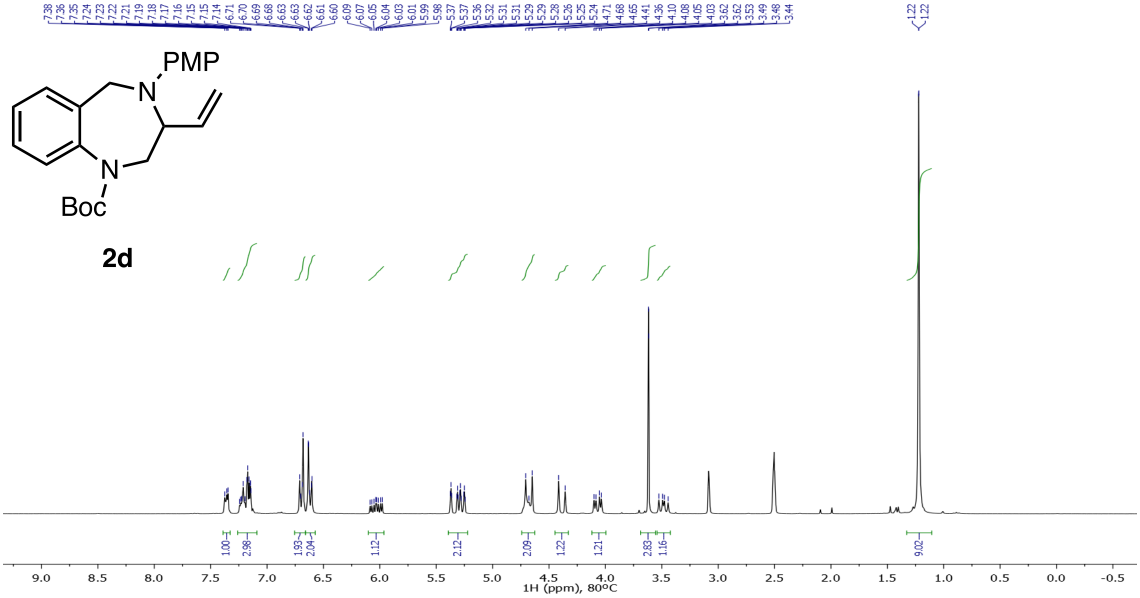

2d

^{13}C-NMR(75 MHz). Solvent $CDCl_3$

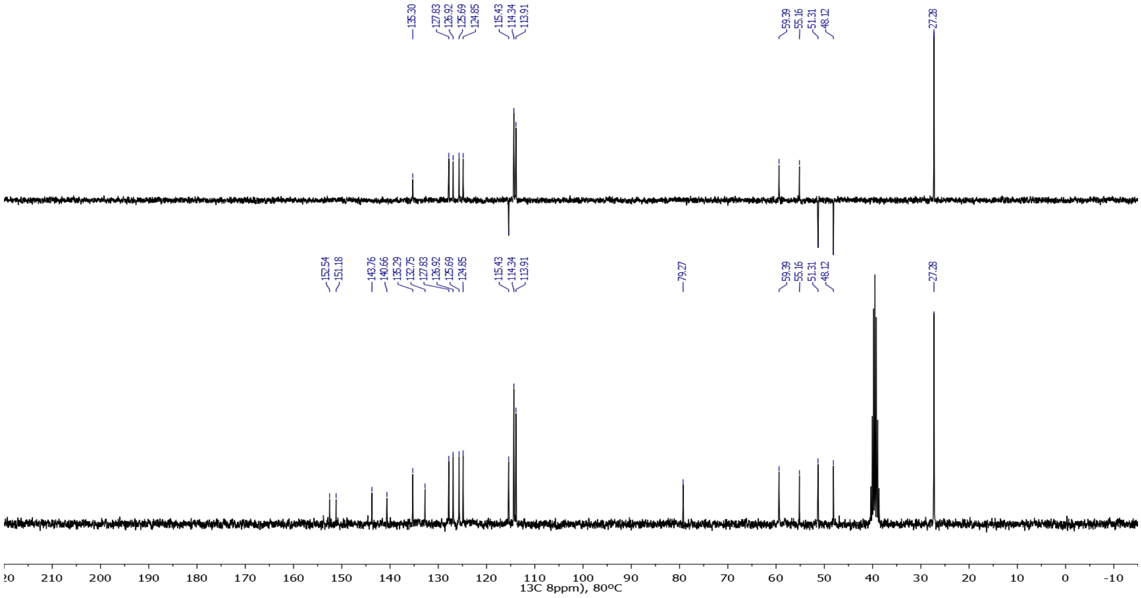

^{1}H-NMR (500 MHz). Solvent $CDCl_3$

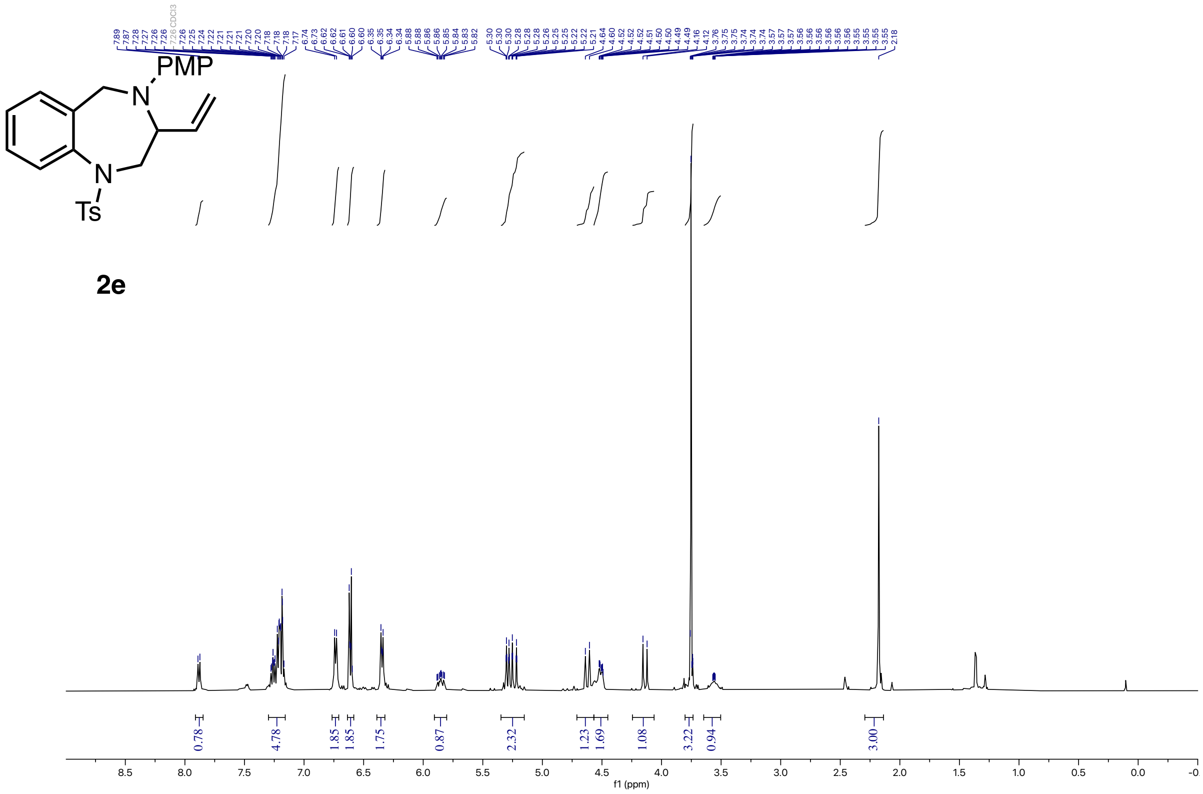

^{13}C-NMR (126 MHz). Solvent $CDCl_3$

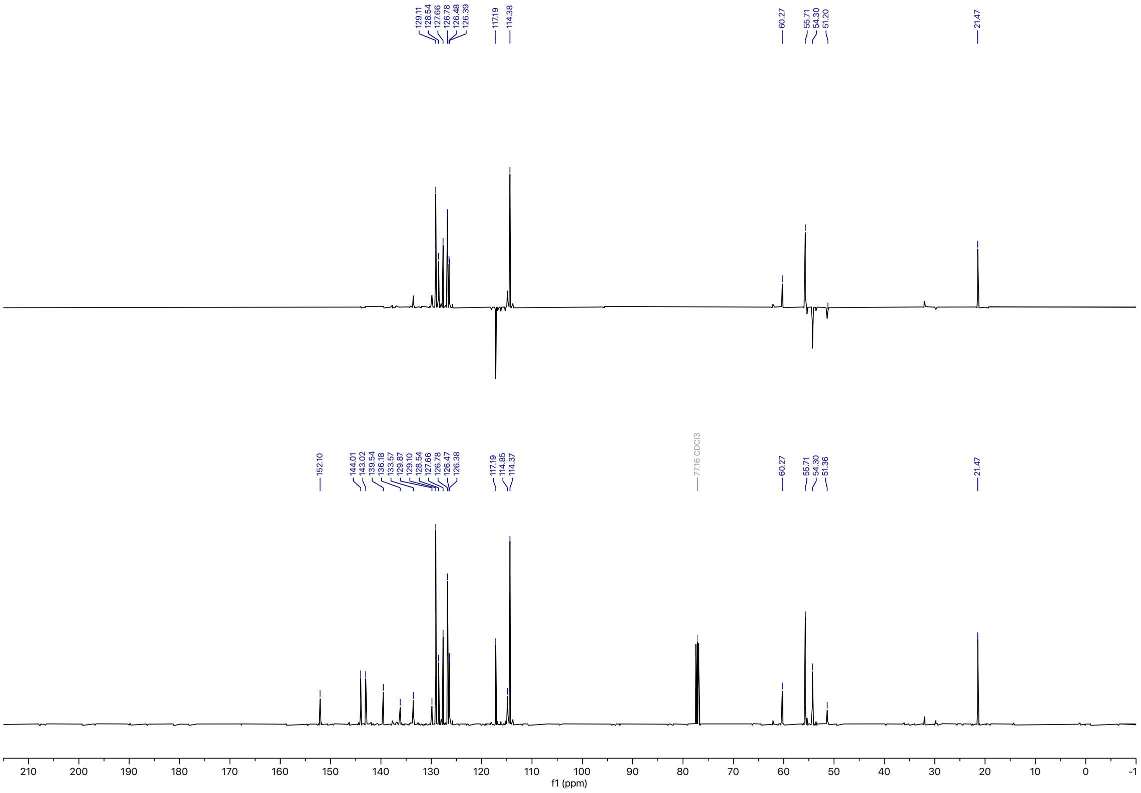

^{1}H-NMR (300 MHz). Solvent $CDCl_3$

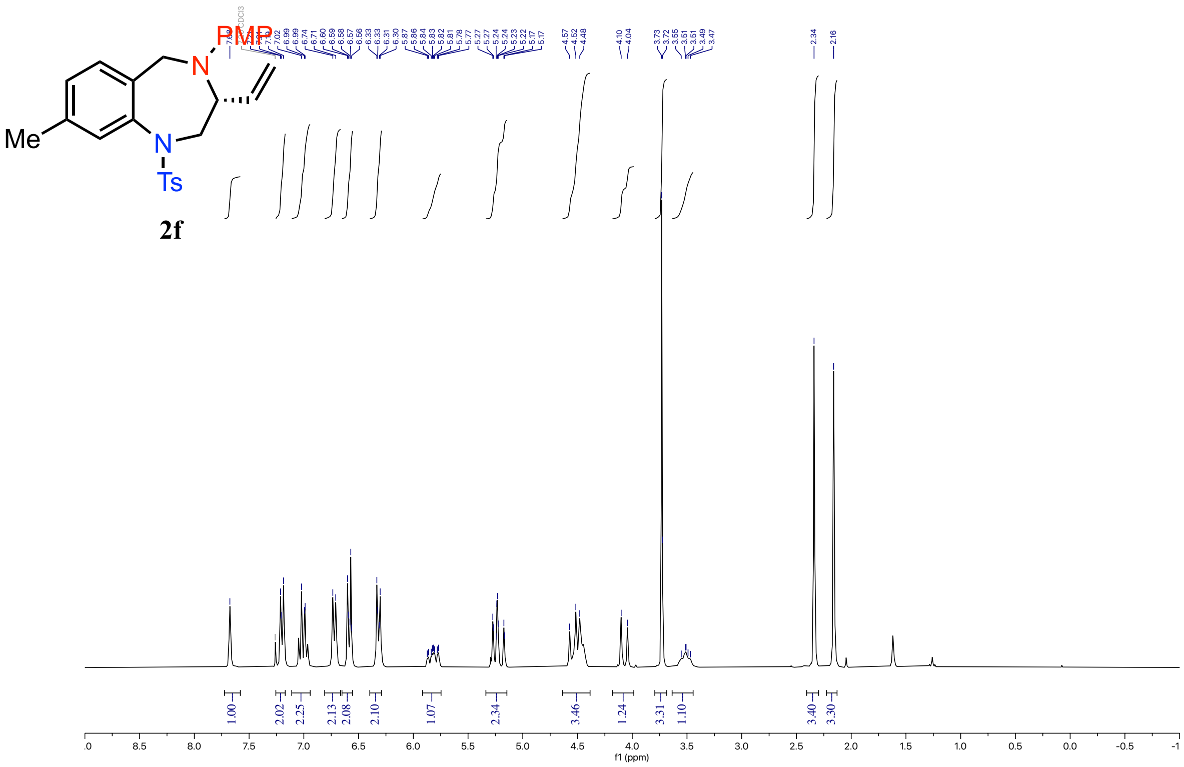

2f

^{13}C-NMR (75 MHz). Solvent $CDCl_3$

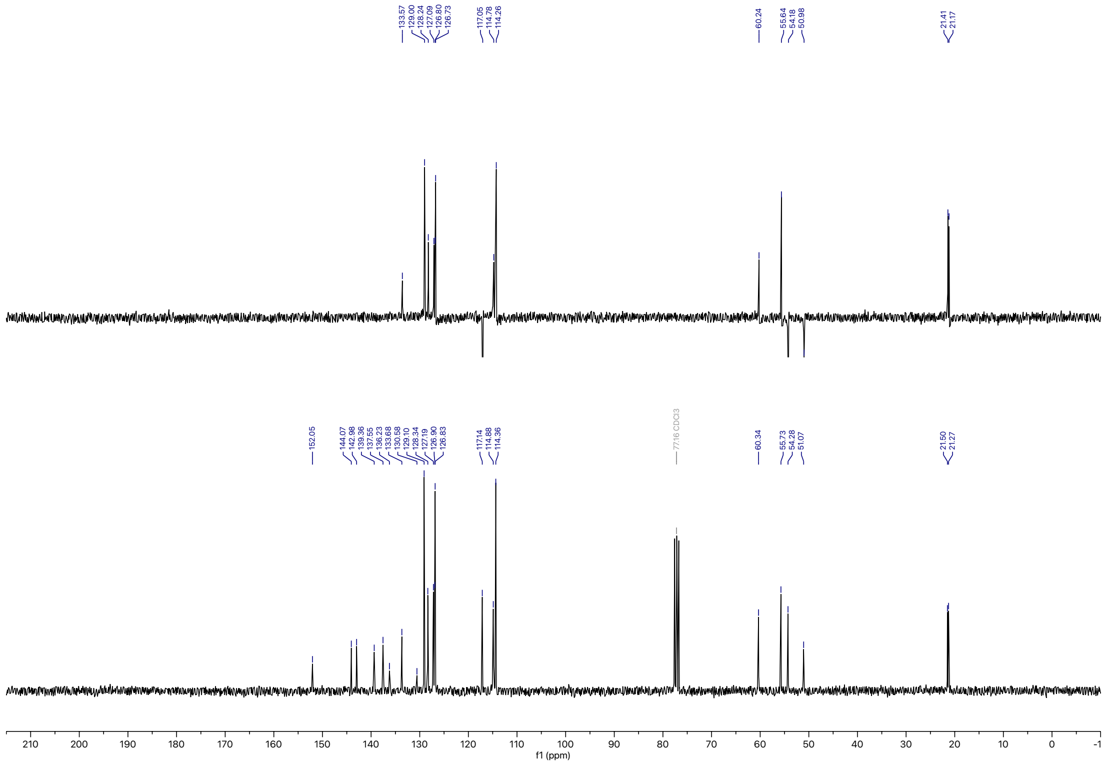

^{1}H-NMR (500 MHz). Solvent $CDCl_3$

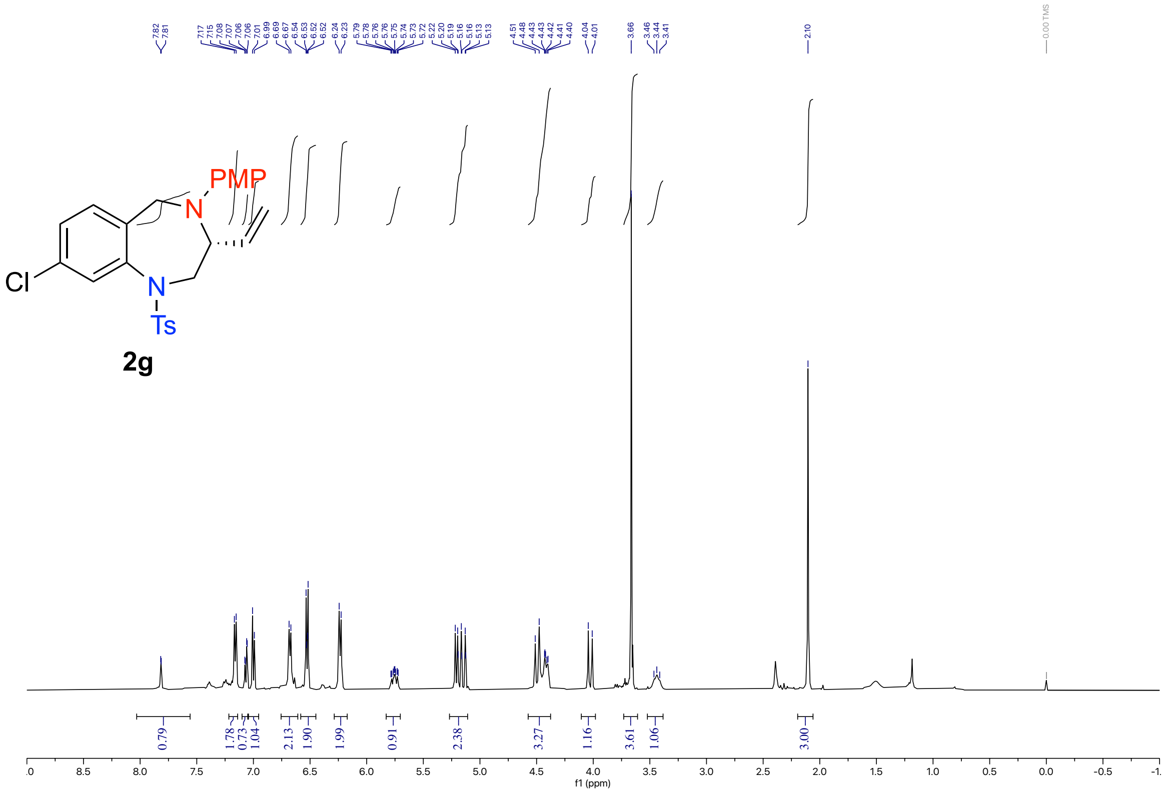

^{13}C-NMR (126 MHz). Solvent $CDCl_3$

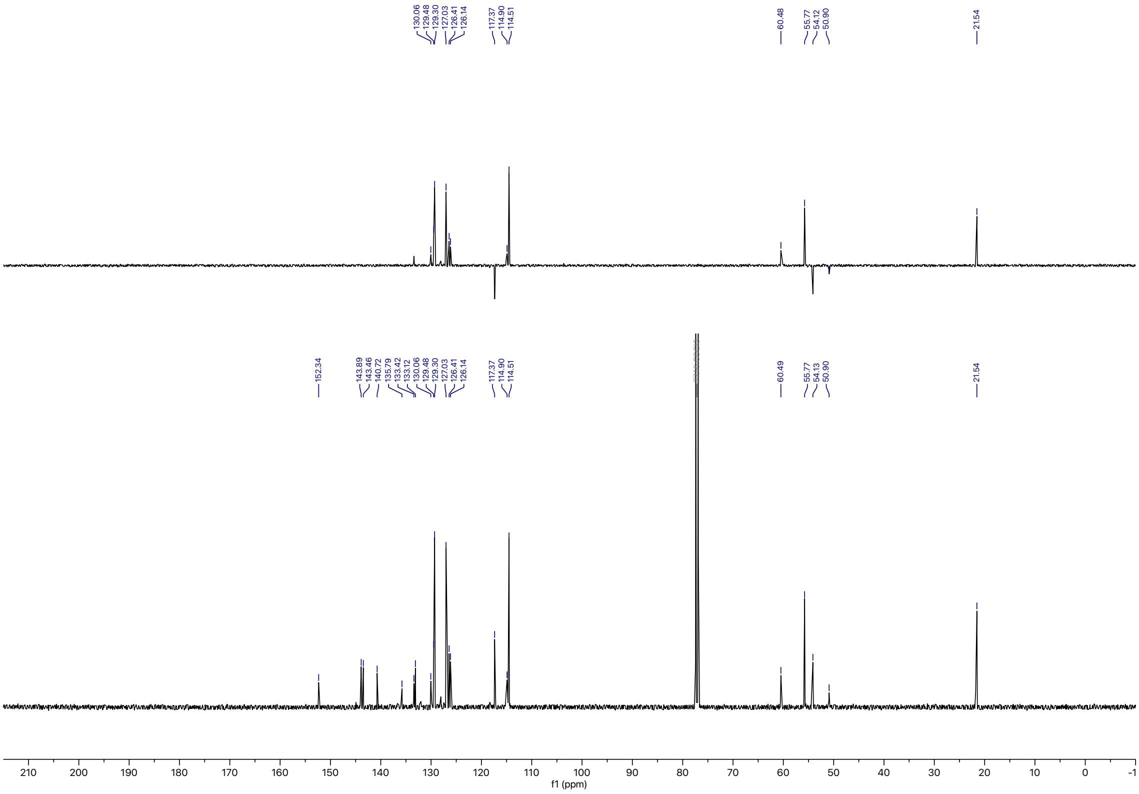

^{1}H-NMR (500 MHz). Solvent $CDCl_3$

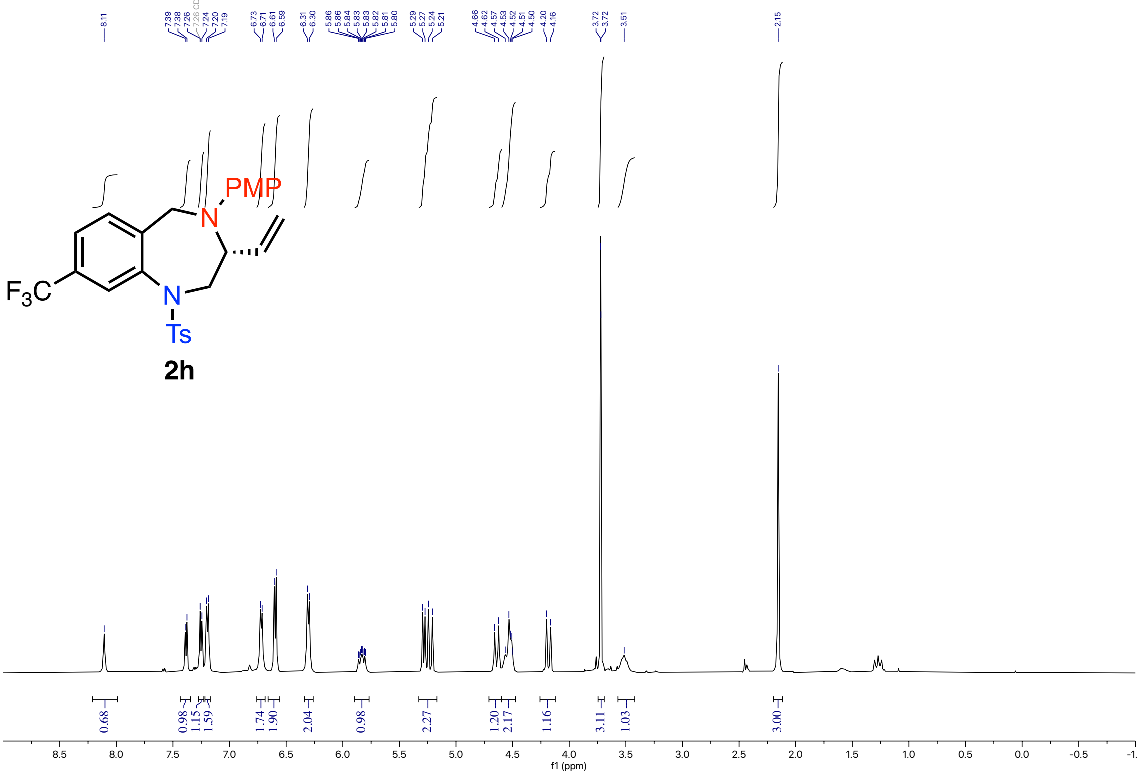

2h

^{13}C-NMR (126 MHz). Solvent $CDCl_3$

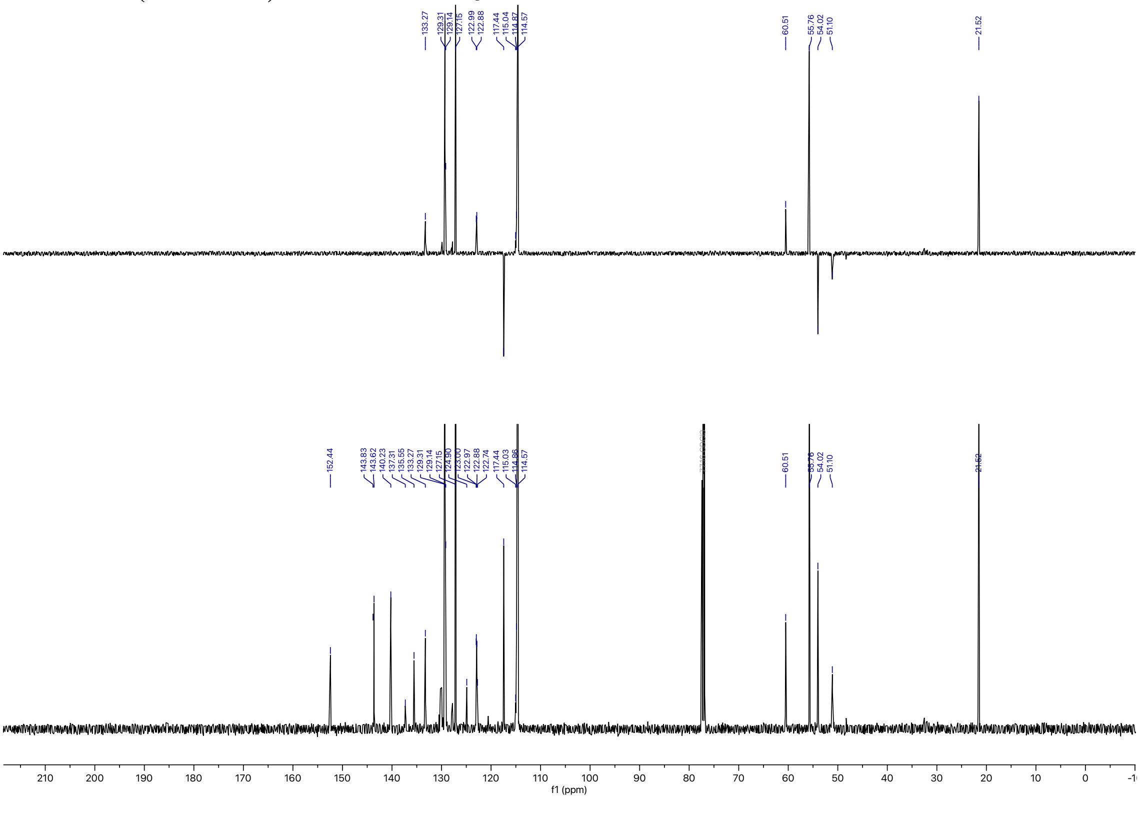

^{19}F-NMR(282 MHz). Solvent $CDCl_3$

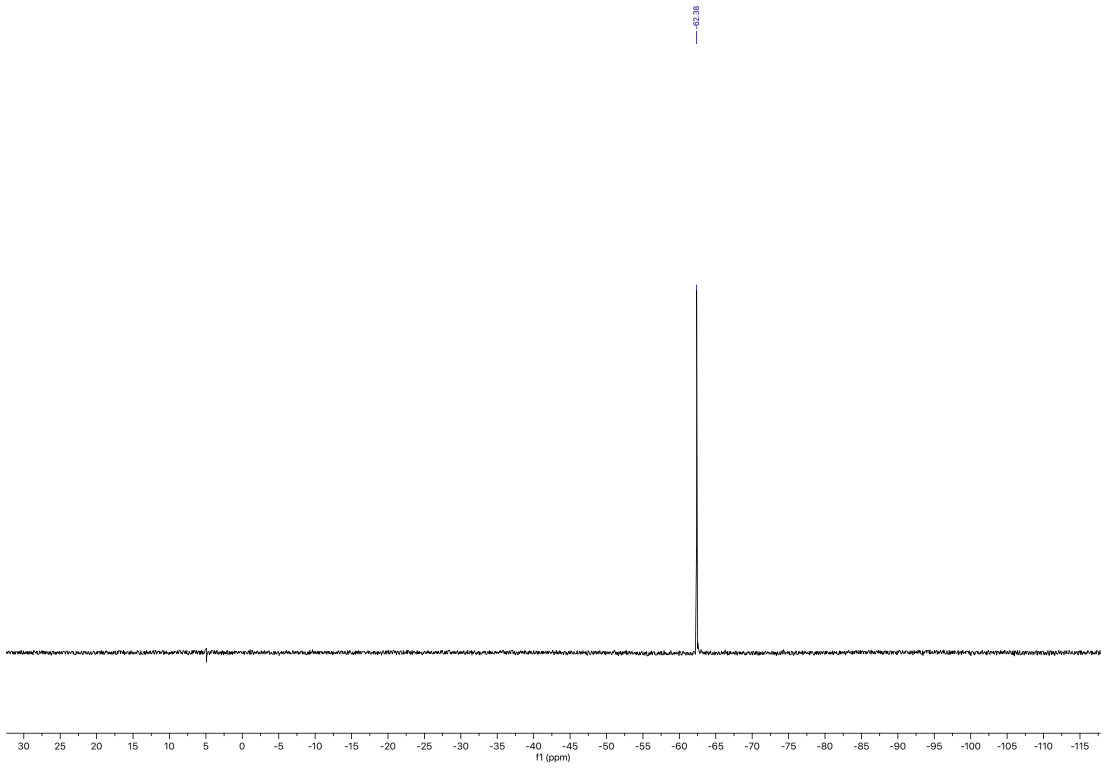

^{1}H-NMR (500 MHz). Solvent $CDCl_3$

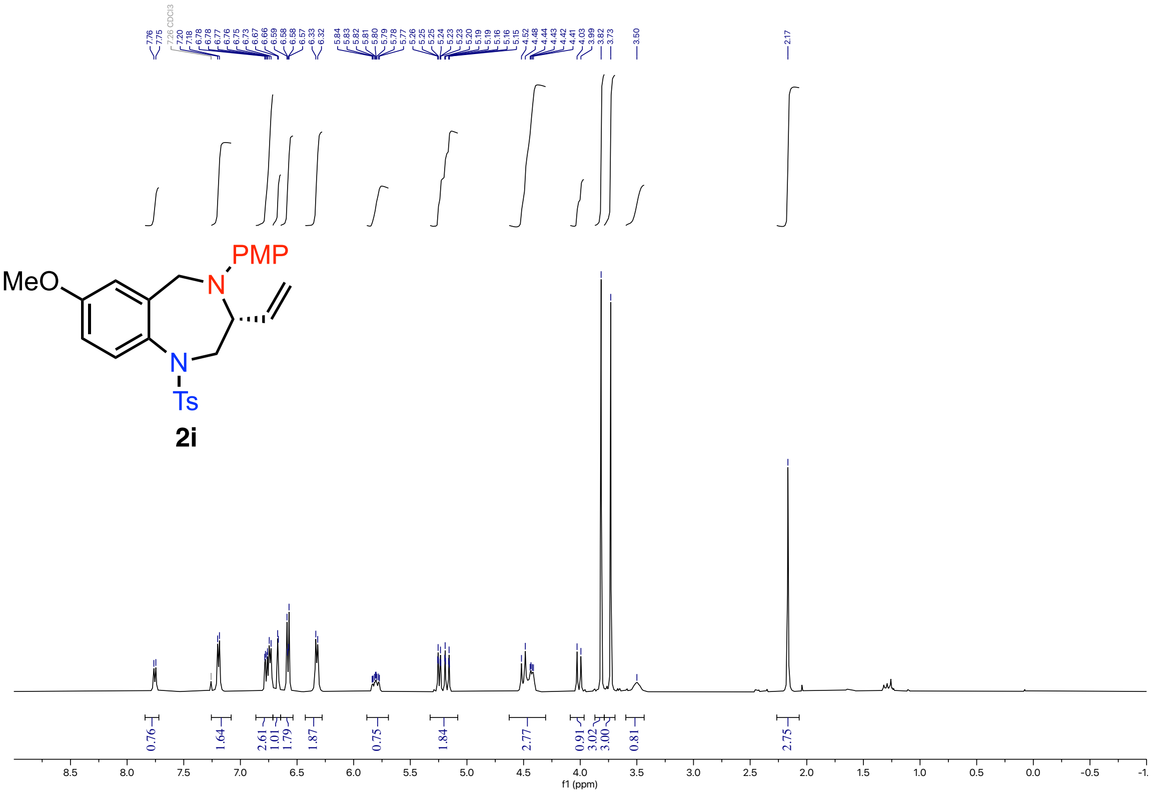

2i

^{13}C-NMR (126 MHz). Solvent $CDCl_3$

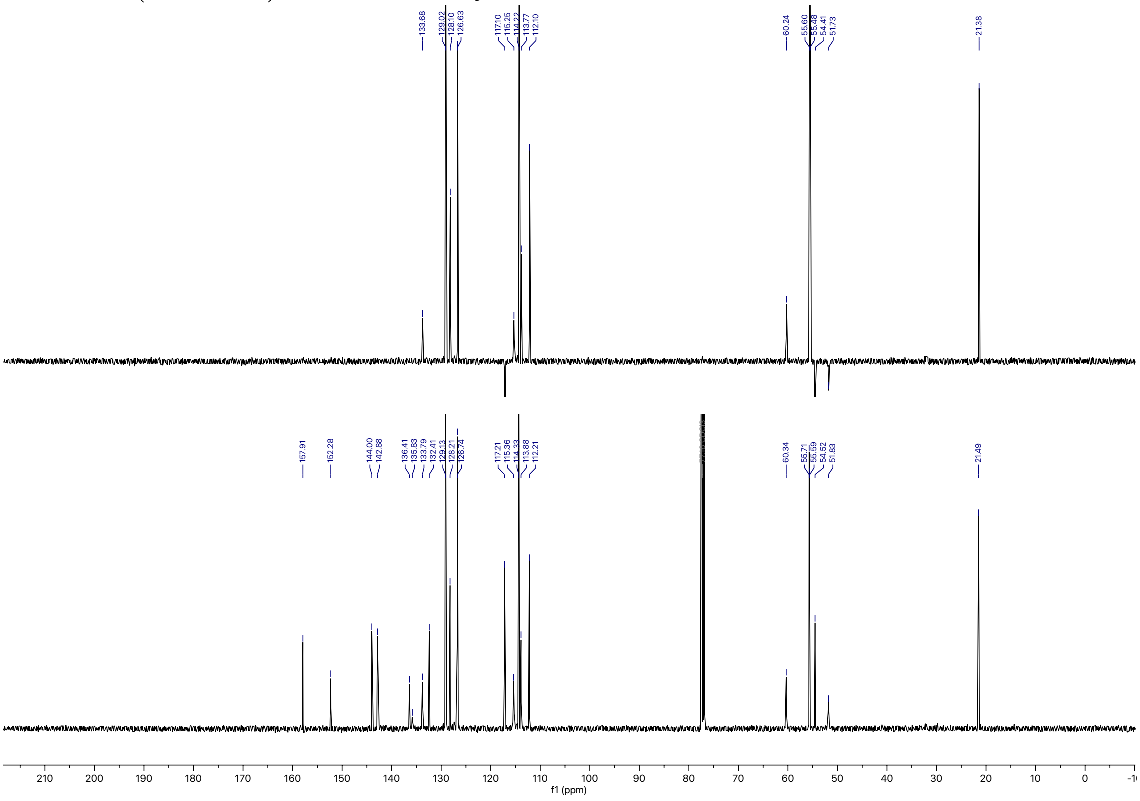

^{1}H-NMR (00 MHz). Solvent $CDCl_3$

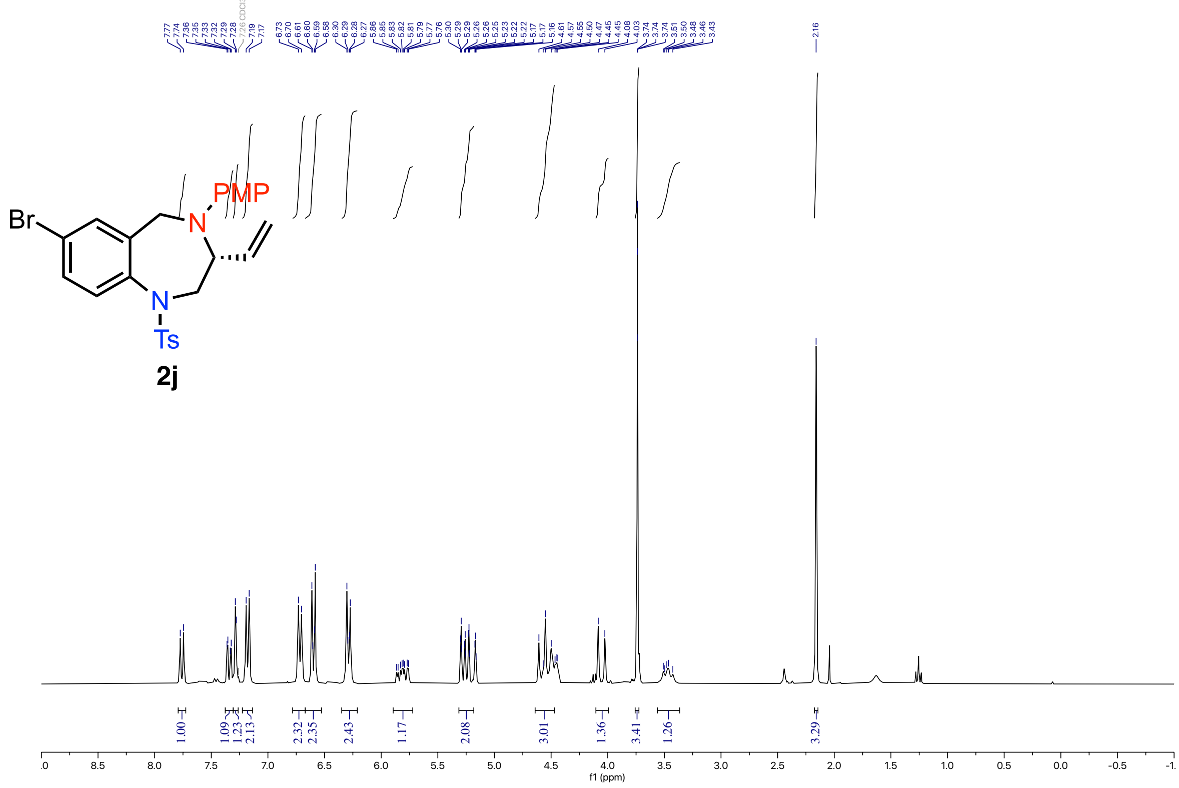

2j

^{13}C-NMR (75 MHz). Solvent $CDCl_3$

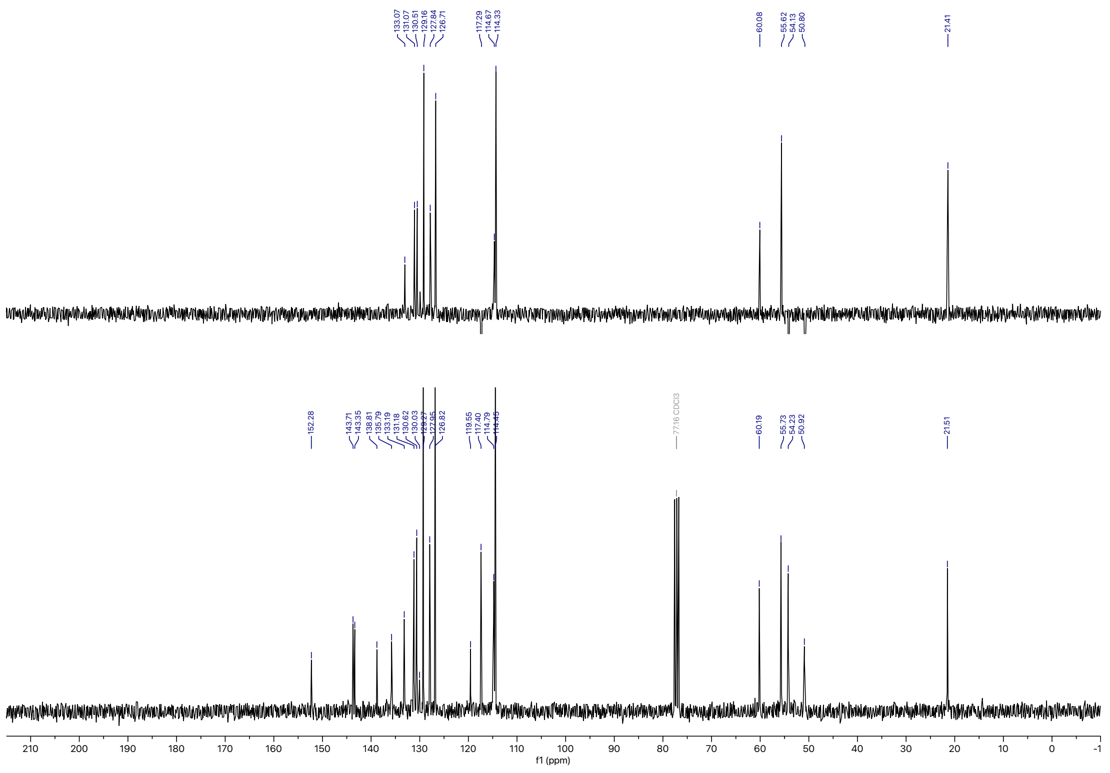

^{1}H-NMR (500 MHz). Solvent $CDCl_3$

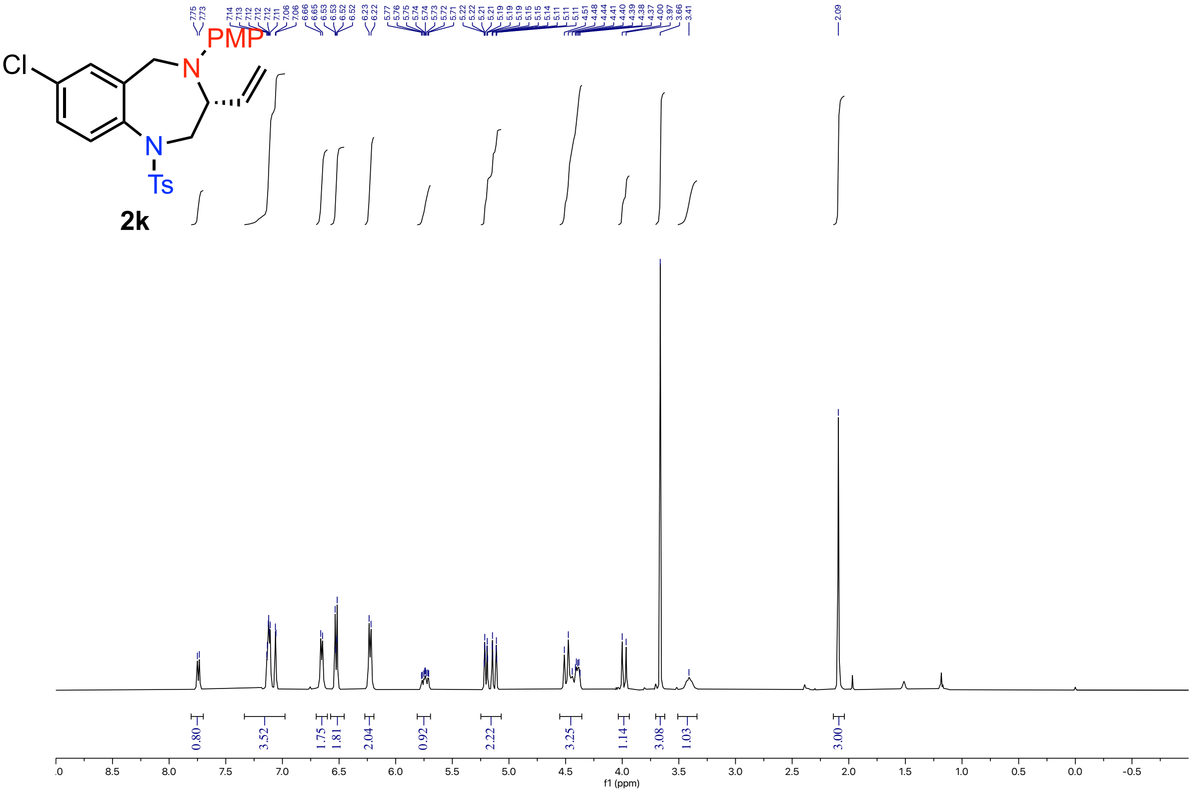

^{13}C-NMR (126 MHz). Solvent $CDCl_3$

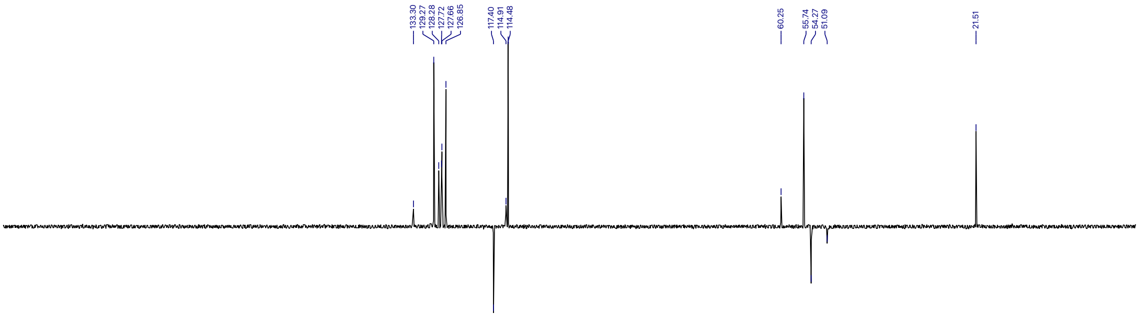

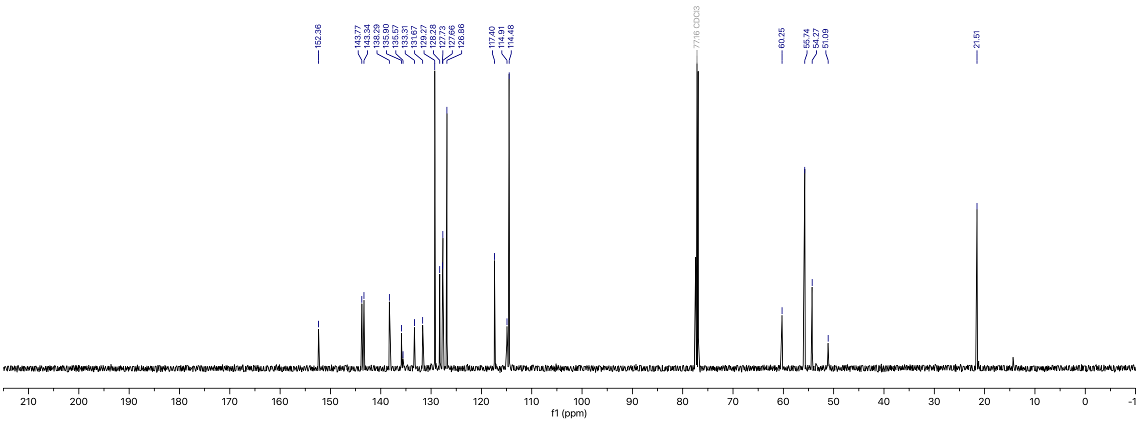

^{1}H-NMR (500 MHz). Solvent $CDCl_3$

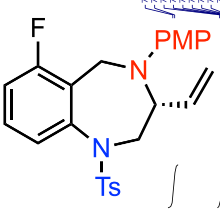

2l

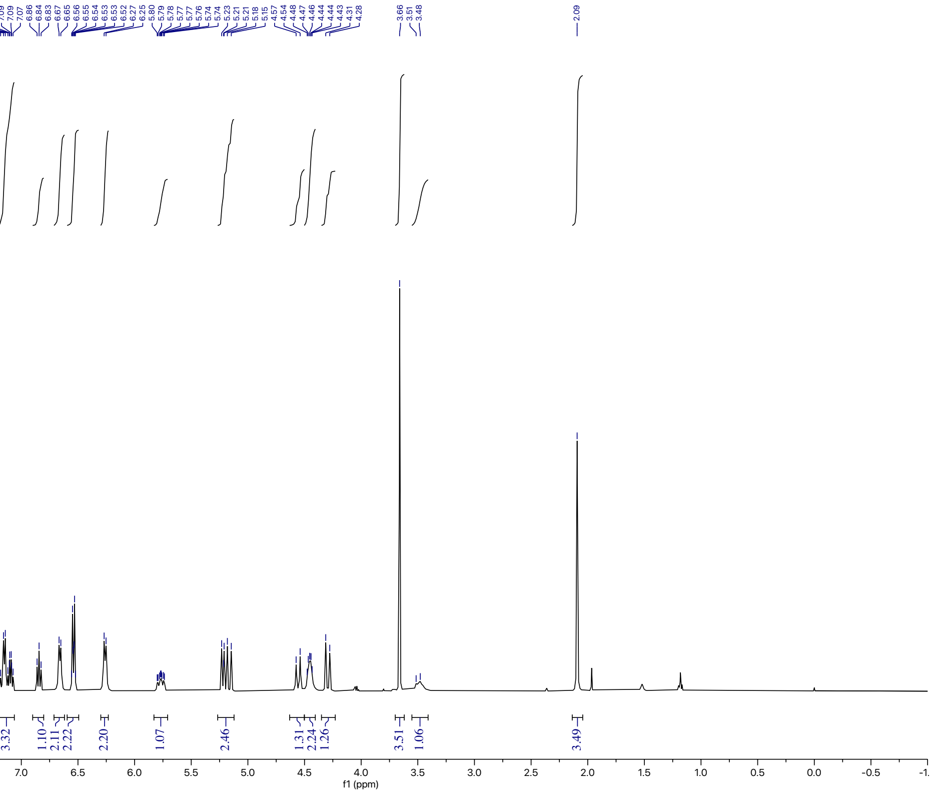

^{13}C-NMR (126 MHz). Solvent $CDCl_3$

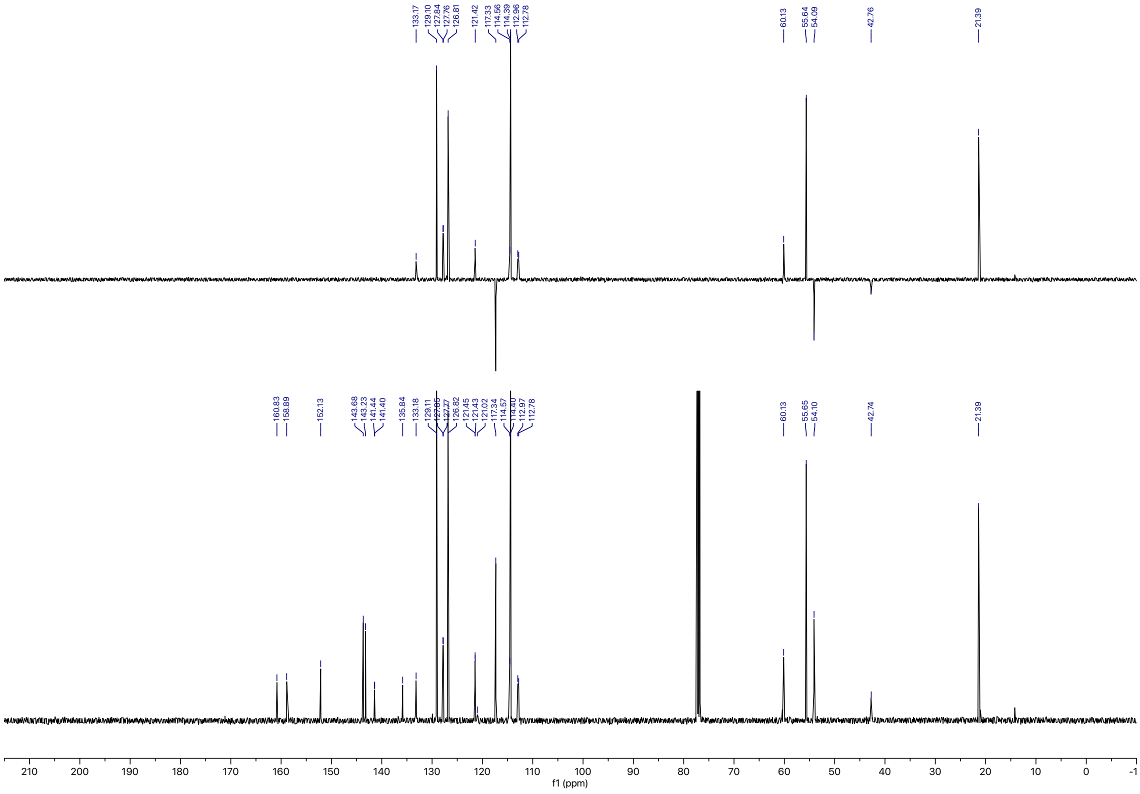

^{19}F-NMR(282 MHz). Solvent $CDCl_3$

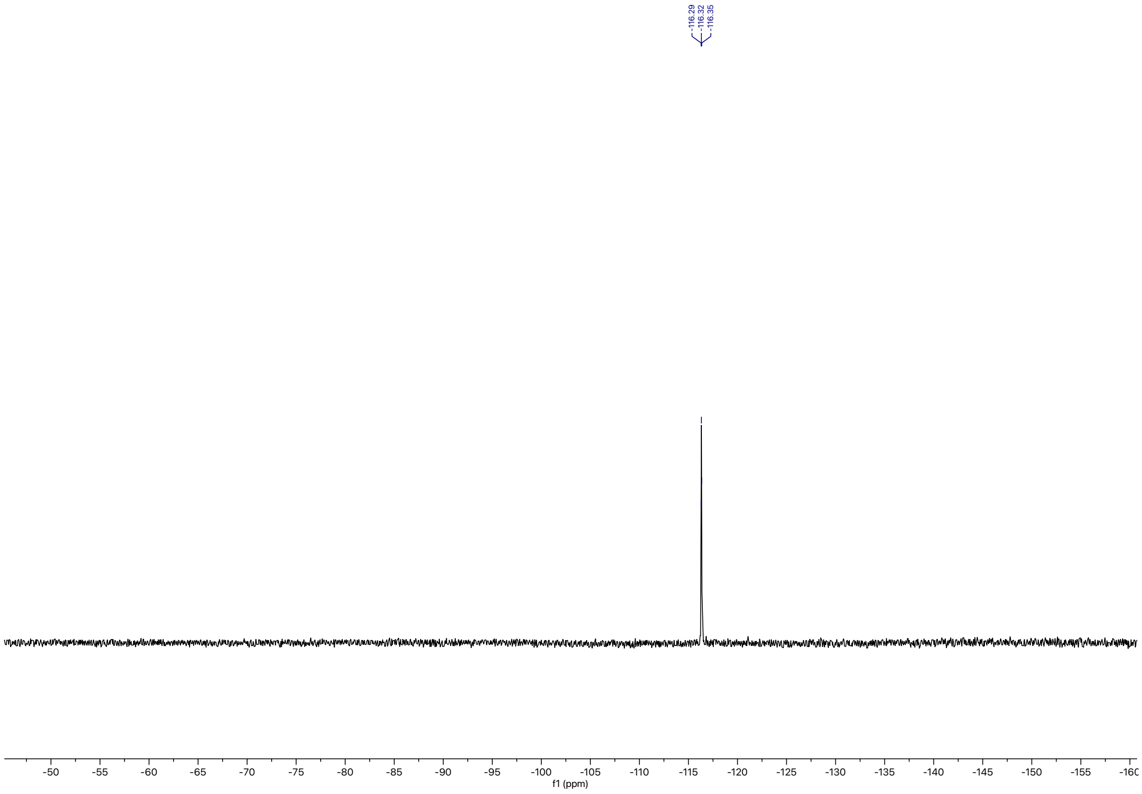

^{1}H-NMR(500 MHz). Solvent $CDCl_3$

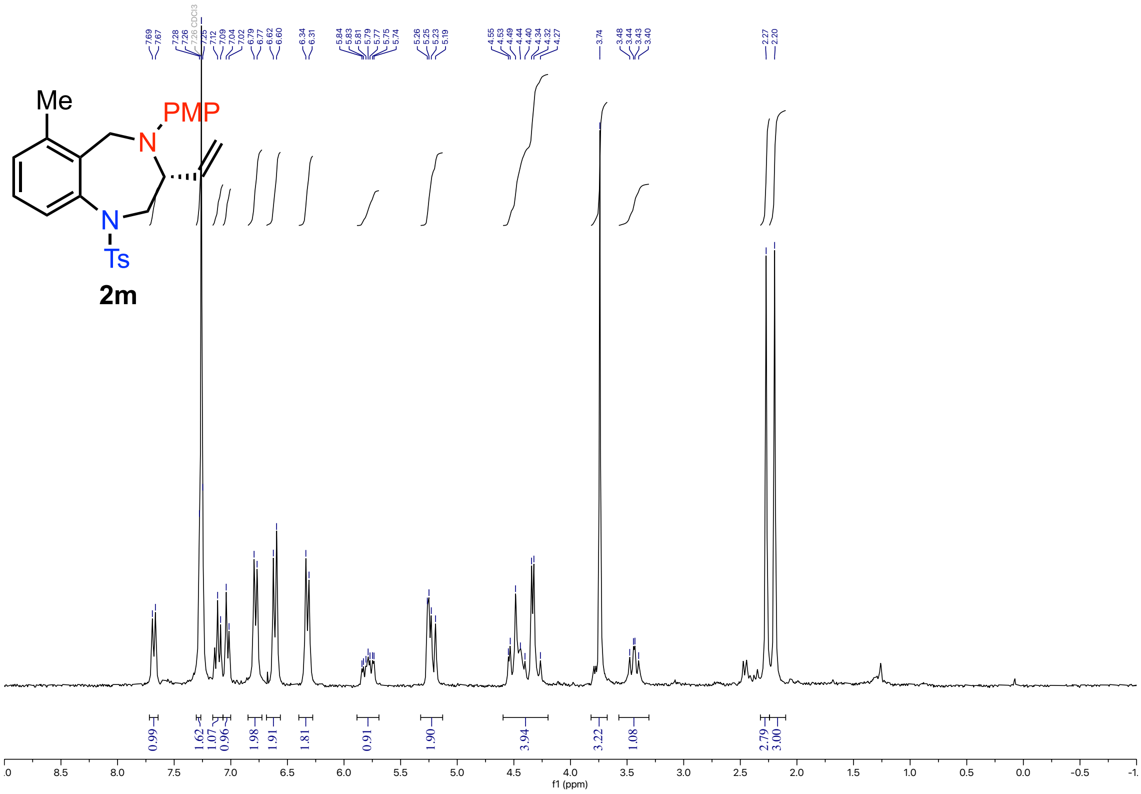

^{13}C-NMR(126 MHz). Solvent $CDCl_3$

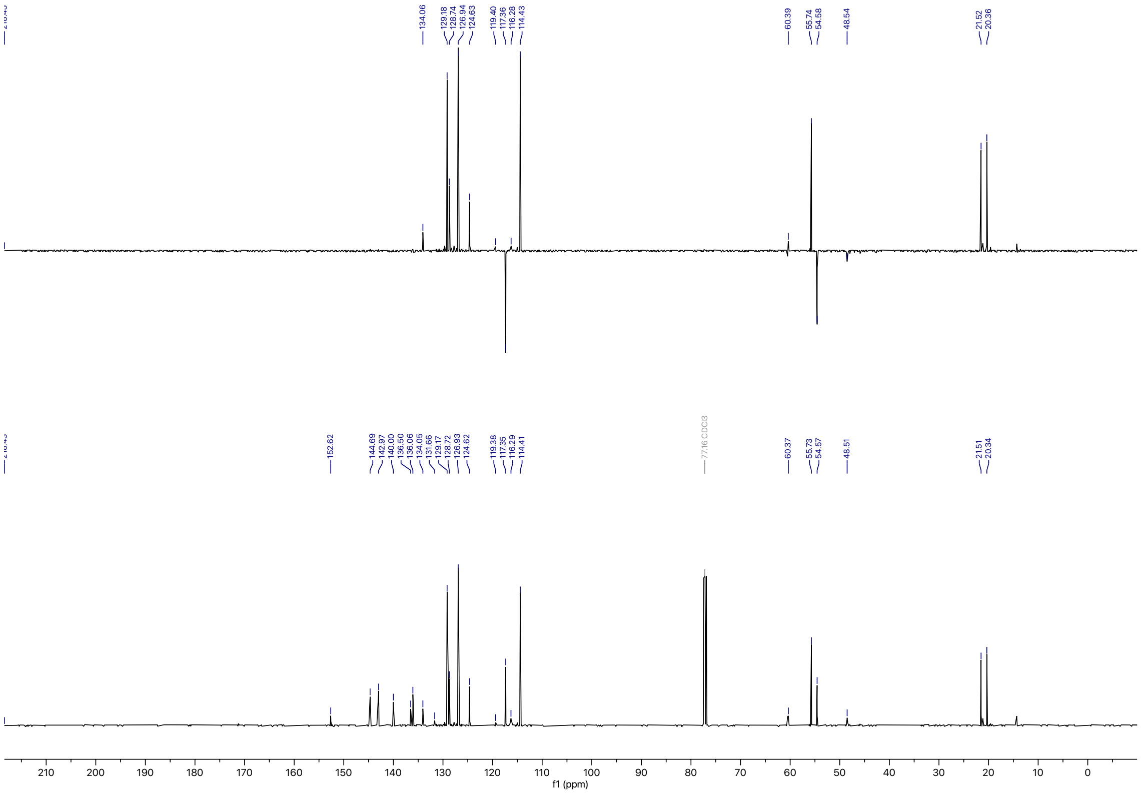

^{1}H-NMR(500 MHz). Solvent $CDCl_3$

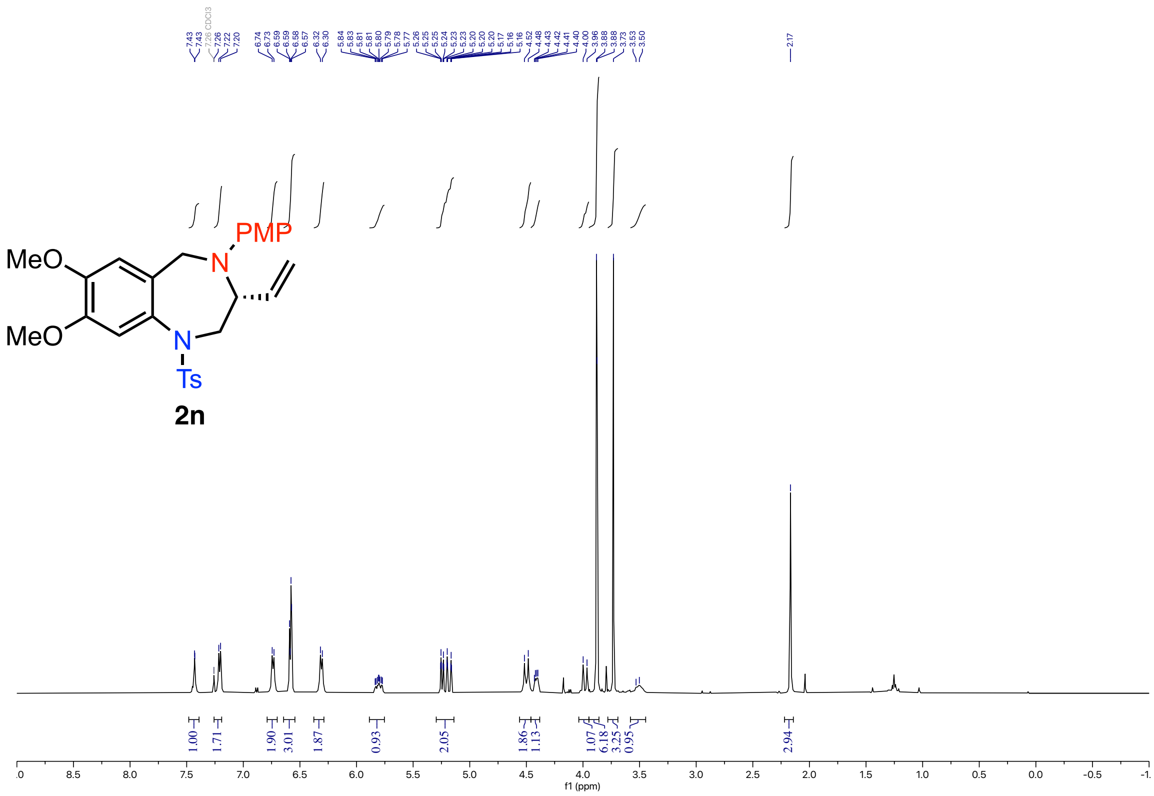

^{13}C-NMR(126 MHz). Solvent $CDCl_3$

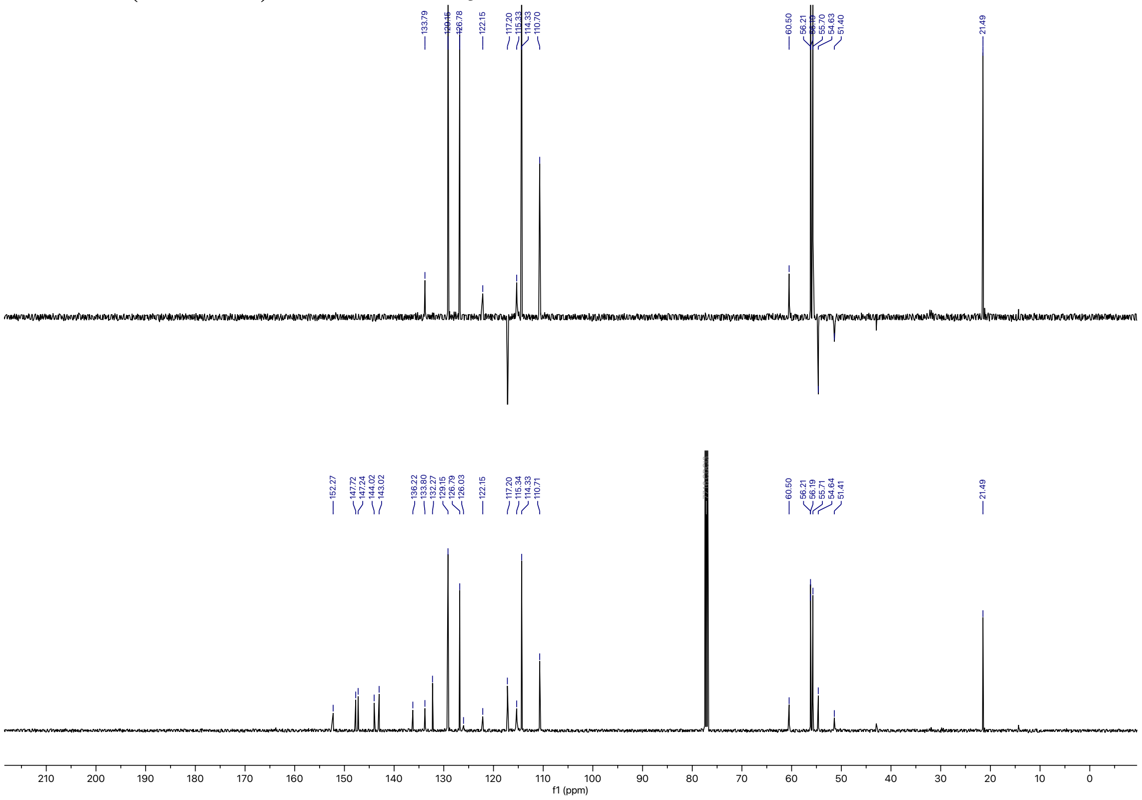

^{1}H-NMR (300 MHz). Solvent CD_3OD

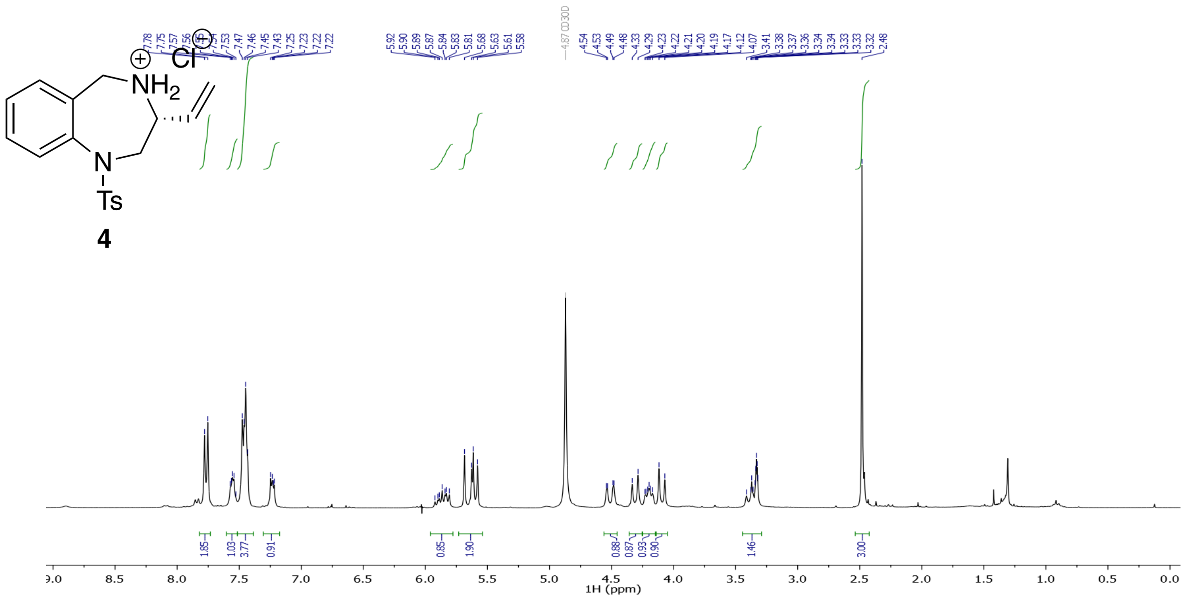

^{13}C-NMR (75 MHz). Solvent CD_3OD

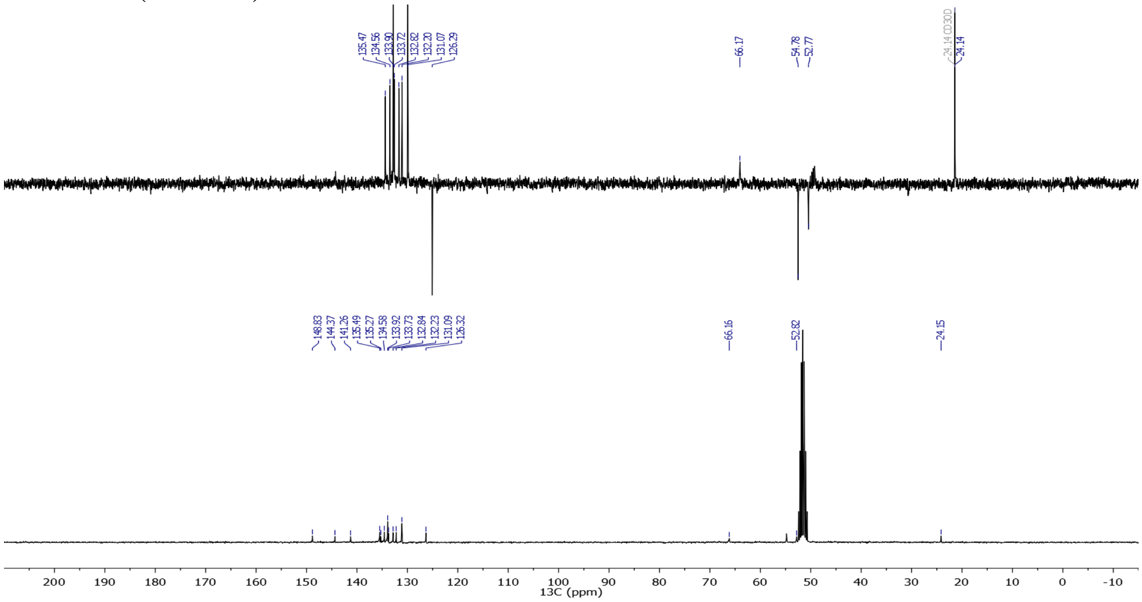

^{1}H-NMR (500 MHz). Solvent $CDCl_3$

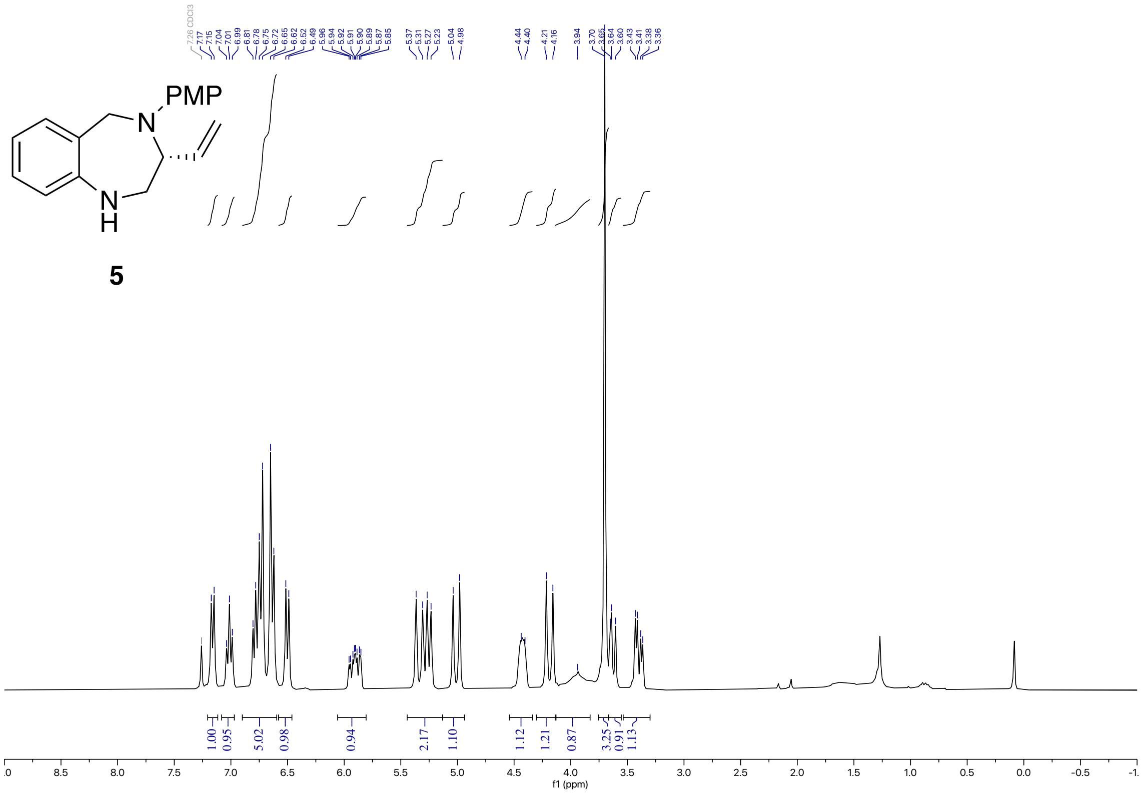

5

^{13}C-NMR (126 MHz). Solvent $CDCl_3$

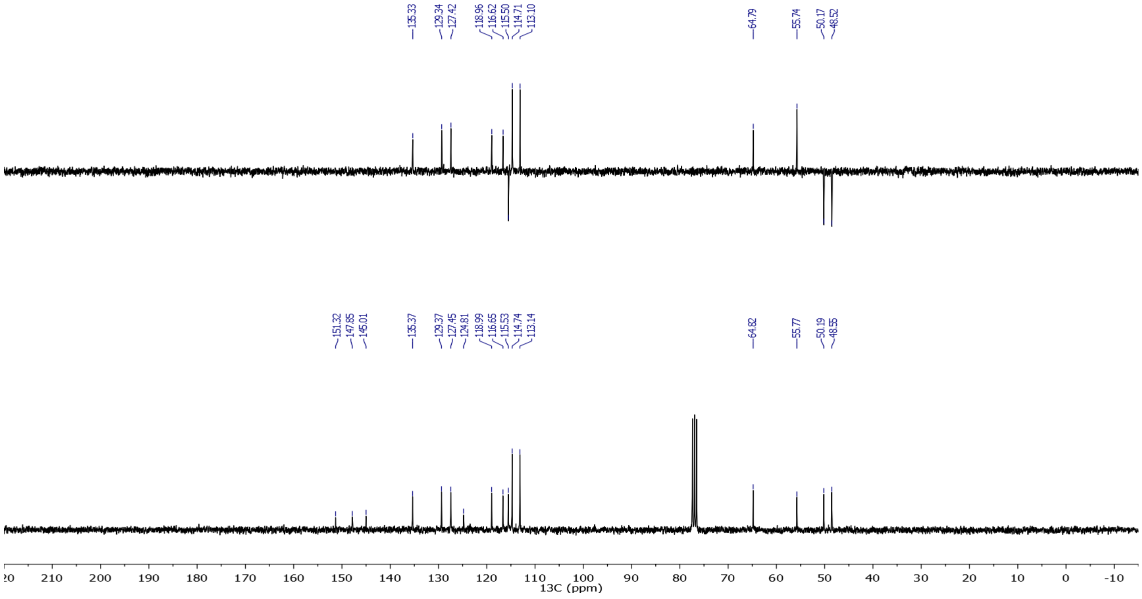

^{1}H-NMR (300 MHz). Solvent DMSO-d_6

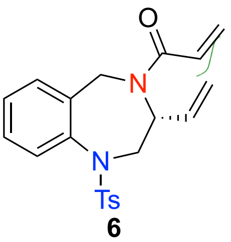

6

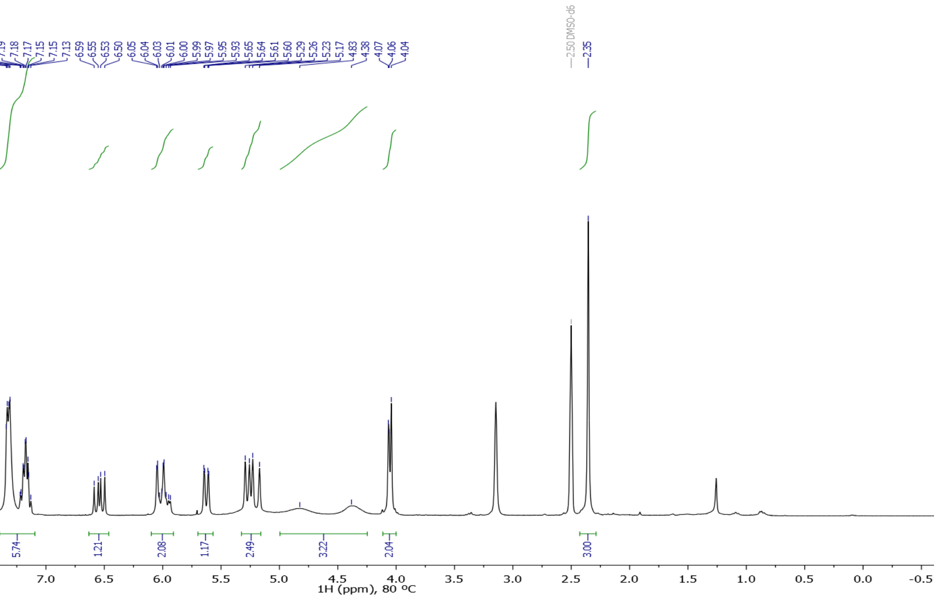

^{13}C-NMR (75 MHz). Solvent DMSO-d_6

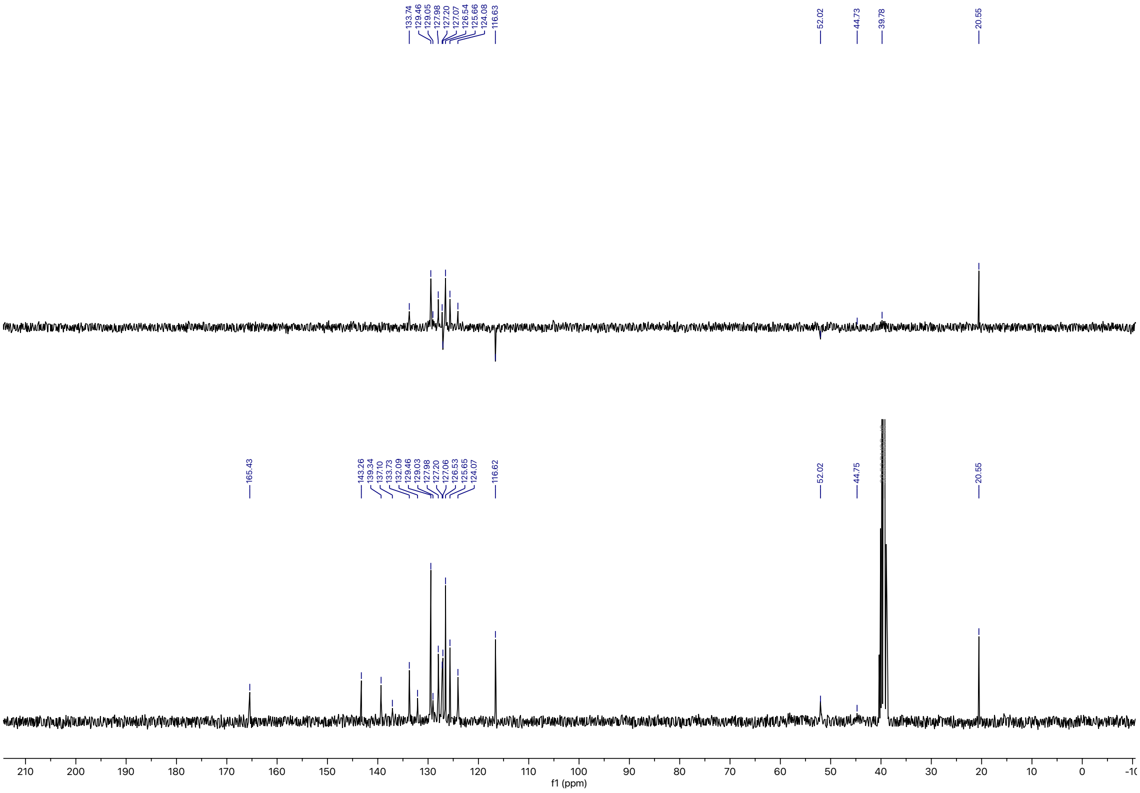

^{1}H-NMR (500 MHz). Solvent $CDCl_3$

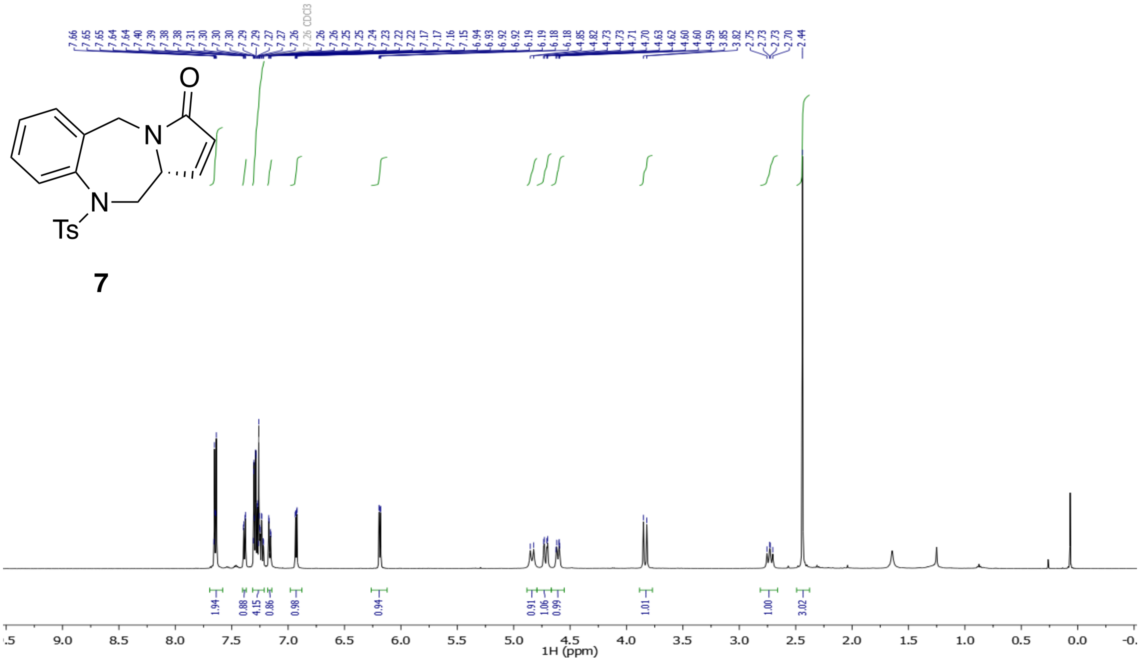

^{13}C-NMR (126 MHz). Solvent $CDCl_3$

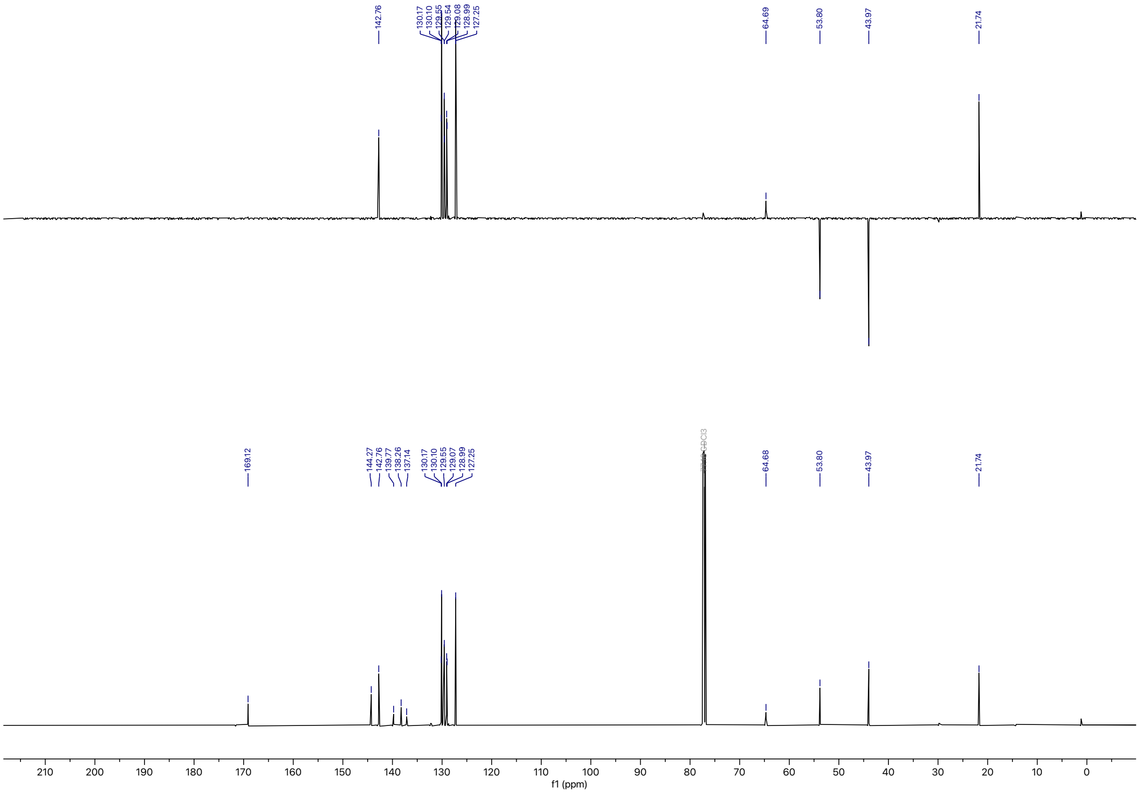

Supplement: Supplementary file 1 — jo1c01268_si_001.pdf [file jo1c01268_si_001.pdf]
